# Development of a Ferroptosis-Related lncRNA Signature to Predict the Prognosis and Immune Landscape of Bladder Cancer

**DOI:** 10.1155/2021/1031906

**Published:** 2021-06-20

**Authors:** Ranran Zhou, Jingjing Liang, Hu Tian, Qi Chen, Cheng Yang, Cundong Liu

**Affiliations:** ^1^Department of Urology, The Third Affiliated Hospital of Southern Medical University, Guangzhou 510000, China; ^2^The Third School of Clinical Medicine, Southern Medical University, Guangzhou 510000, China; ^3^Department of Cardiology, Shunde Hospital of Southern Medical University, Foshan 528000, China

## Abstract

The tight relationship between ferroptotic cell death and immune response demonstrated by recent studies enlightened us to detect the underlying roles of ferroptosis-related long noncoding RNAs (frlncRNAs) in the tumor microenvironment of bladder cancer (BCa). We collected 121 ferroptosis regulators from previous studies. Based on their expression values, 408 cases with BCa were clustered. The patients in different clusters showed diverse immune infiltration, immunotherapy response, and chemotherapy effectiveness, revalidating the tight correlation with ferroptosis and tumor immunity. Through differential, coexpression, Kaplan-Meier, Lasso, and Cox analysis, we developed a 22-lncRNA-pair signature to predict the prognosis of BCa based on gene-pair strategy, where there is no need for definite expression values. The areas under the curves are all over 0.8. The risk model also helped to predict immune infiltration, immunotherapeutic outcomes, and chemotherapy sensitivity. Totally, the prognostic assessment model indicated a promising predictive value, also providing clues for the interaction between ferroptosis and BCa immunity.

## 1. Introduction

Bladder cancer (BCa) is one of the most common malignancies worldwide, with high morbidity and mortality [1]. More than 500,000 new BCa cases and 200,000 BCa-related deaths occur worldwide annually [2]. BCa has two main subtypes: muscle-invasive BCa and nonmuscle-invasive BCa. Although the 5-year survival rate of nonmuscle-invasive BCa is about 90%, approximately 15–20% of such cases would progress to the muscle-invasive stage and even to distant metastasis, which has a dismal 5-year survival rate of 5–30% [1]. Recently, some large-scale clinical trials, such as KEYNOTE-045 and IMvigor211, demonstrated that BCa is susceptible to immune checkpoint inhibitors (ICIs), representing an important advancement in the treatment of BCa [3, 4].

Ferroptosis, a form of iron-dependent and nonapoptotic cell death, is attracting increasing attention in view of the fact that apoptosis resistance is one of the hallmarks of tumors [5]. Inducing tumor cell ferroptosis seems to be an attractive and promising therapeutic strategy, especially for drug-resistant malignancies. Recent studies have verified that ferroptotic tumor cells and the tumor microenvironment (TME) could influence each other [6, 7], suggesting that the combination of ICIs and ferroptosis inducers is a promising treatment.

Long noncoding RNAs (lncRNAs), a type of RNA molecule with transcripts of >200 nucleotides, participate in tumorigenesis and cancer development not only by altering the malignancy of cancer cells themselves but also by changing the TME, as has been reported in many studies [8]. In recent years, the interaction between lncRNAs and ferroptosis has also been investigated. For instance, the lncRNA *P53RRA* serves as a tumor suppressor by promoting *p53* maintenance in the nucleus, thus, facilitating ferroptosis [9].

Immune-related signatures for predicting the status of cancer immune infiltration are receiving increasing attention with the clinical application of ICIs, as such signatures might help predict prognosis and immunotherapy outcomes. Because lncRNAs are involved in the regulation of 70% of gene expression, signatures based on lncRNA expression have been investigated by many researchers. Meanwhile, some researchers have proposed the gene-pair strategy, which focuses on the difference between two genes regardless of the exact gene expression, to construct a prediction model, making immune-related signatures more widely applicable [10].

This study had three phases. First, unsupervised clustering based on known ferroptosis regulators was performed to determine the correlation between ferroptosis and the TME in BCa. Second, using the gene-pair strategy, we constructed a ferroptosis-related lncRNA (frlncRNA) signature to predict overall survival (OS). Lastly, the diagnostic ability of the risk score calculated by the model for immunotherapy response, chemotherapy results, and immune infiltration was also evaluated. From this analysis, we aimed to verify the close association between ferroptosis and the TME and to propose an important tool for predicting the prognosis and immune infiltration of BCa.

## 2. Materials and Methods

### 2.1. Transcriptome Data Collection and Ferroptosis-Related Genes

The transcription data (RNA-seq) and corresponding clinicopathological features were downloaded from The Cancer Genome Atlas (TCGA) up to September 3, 2020 (https://portal.gdc.cancer.gov/). A total of 121 ferroptosis regulators were identified from a previous study [5]. To select lncRNAs, the annotation file was obtained from the Ensembl database (http://asia.ensembl.org). lncRNAs with an average expression value of <0.5 were excluded from the study. Differential analysis was performed using the edgeR package [11], and false discovery rate (FDR) < 0.05 and ∣logarithmic fold change [logFC] | >1 and 2 were set as the thresholds for ferroptosis regulators and frlncRNAs, respectively. The frlncRNAs were screened through coexpression analysis, with Pearson correlation coefficients >0.5 and *p* > 0.05 as filtering criteria.

### 2.2. Unsupervised Clustering

The mRNA expression profile of 121 ferroptosis regulators was adopted for consensus clustering with the ConsensusClusterPlus R package [12] using the *K*-means method. The cumulative distribution function was implemented to detect the optimal *K* value, which represented the clustering number, and the result was validated using principal component analysis (PCA) in R software.

### 2.3. Gene-Pair Strategy

The gene-pair strategy was used for 0-or-1 matrix construction, as previously reported [10]. For instance, if the expression value of the A gene is higher than that of the B gene, then C, which means the gene pair comprising A and B, is considered as 1; otherwise, C is considered as 0. All frlncRNAs were paired, and a new 0-or-1 matrix was constructed. An frlncRNA pair was excluded from the study if its value in all samples was 0 or 1.

### 2.4. Survival Analysis

Cases with a follow-up duration of <30 days were not included in the present analysis. We implemented the survival R package to conduct Kaplan–Meier (KM) survival analysis with the log-rank test for the comparison of OS between different cohorts. To simplify the model and avoid overfitting as much as possible, Lasso–Cox regression with 10-fold cross-validation was conducted using the glmnet package, and the threshold was set at 0.05. Univariate and multivariate Cox analyses were performed using the survival package. The area under the curve (AUC) values were calculated using the survival ROC package to estimate the effectiveness of the established model.

### 2.5. Association with Clinicopathological Factors

To detect whether the risk score, which was calculated using the established model, was an independent risk factor, univariate and multivariate Cox regression analyses were performed using the survival R package. The clinicopathological features were all transformed into dichotomized variables, including risk score (high vs. low), tumor grade (high vs. low), age (≤64 vs. >64 years), sex (female vs. male), pathological *T* stage (*T* 1–2 vs. *T* 3–4), *N* stage (*N*0 vs. *N*1–3), and *M* stage (*M*0 vs. *M*1).

### 2.6. Immune Infiltration Evaluation

Different methods were used to evaluate the immune infiltration status. The ESTIMATE algorithm [13] through the estimate package of R was used to calculate the ratio of immune and stromal components of the TME, which were quantified as ImmuneScore and StromalScore, respectively. CIBERSORT [14] and TIMER [15] were used to evaluate the abundance profile of immune cells in the TME with *p* < 0.05 filtering.

### 2.7. Single-Sample GSEA (ssGSEA)

The ssGSEA method was implemented using the Gene Set Variation Analysis (GSVA) and GSEABase packages to analyze the immune and inflammatory infiltration profiles. The results were visualized using the pheatmap package.

### 2.8. Enrichment Analysis

Functional enrichment analysis of lncRNAs was conducted using LncSEA (http://www.licpathway.net/LncSEA/) with FDR < 0.05 and Bonferroni < 0.05. GSEA software (version 4.1.0) was downloaded from the GSEA website (https://www.gsea-msigdb.org/), and GSVA was performed using the GSVA R package. Hallmark gene sets v7.2 and iron metabolism-related gene sets were collected from the Molecular Signatures Database (https://www.gsea-msigdb.org/gsea/msigdb/). For GSVA, ∣logFC | >0.03 and adjusted *p* < 0.05 were considered as thresholds.

### 2.9. Transcription Factor Prediction

The JASPAR2020 [16] and TFBSTools [17] packages were used to predict transcription factors with sequences of 500 bp downstream to 2000 bp upstream from the transcription start point of lncRNAs. When the score, which was positively correlated with the possibility of transcription binding, was >0.99, the transcription factors were included in further analyses.

### 2.10. Estimating the Effectiveness of frlncRNA Signature for Clinical Treatment

The likelihood of an immunotherapy response was estimated using the TIDE algorithm [18]. In addition, the association between the risk score and some known predictors of immunotherapeutic sensitivity, such as *CXCL9* and *CXCL13* expression, was also investigated [19]. The half inhibitory concentration (IC50) values of commonly used chemotherapeutic drugs, such as cisplatin, doxorubicin, gemcitabine, methotrexate, and vinblastine, were obtained using the pRRophetic R package in patients with stage ≥ *T*2 BCa [20]. The survival information of patients who had received chemotherapy and had stage ≥ *T*2 BCa was downloaded from TCGA.

## 3. Results

### 3.1. The Tight Association between Ferroptosis and BCa Immune Infiltration

**Figure 1:**
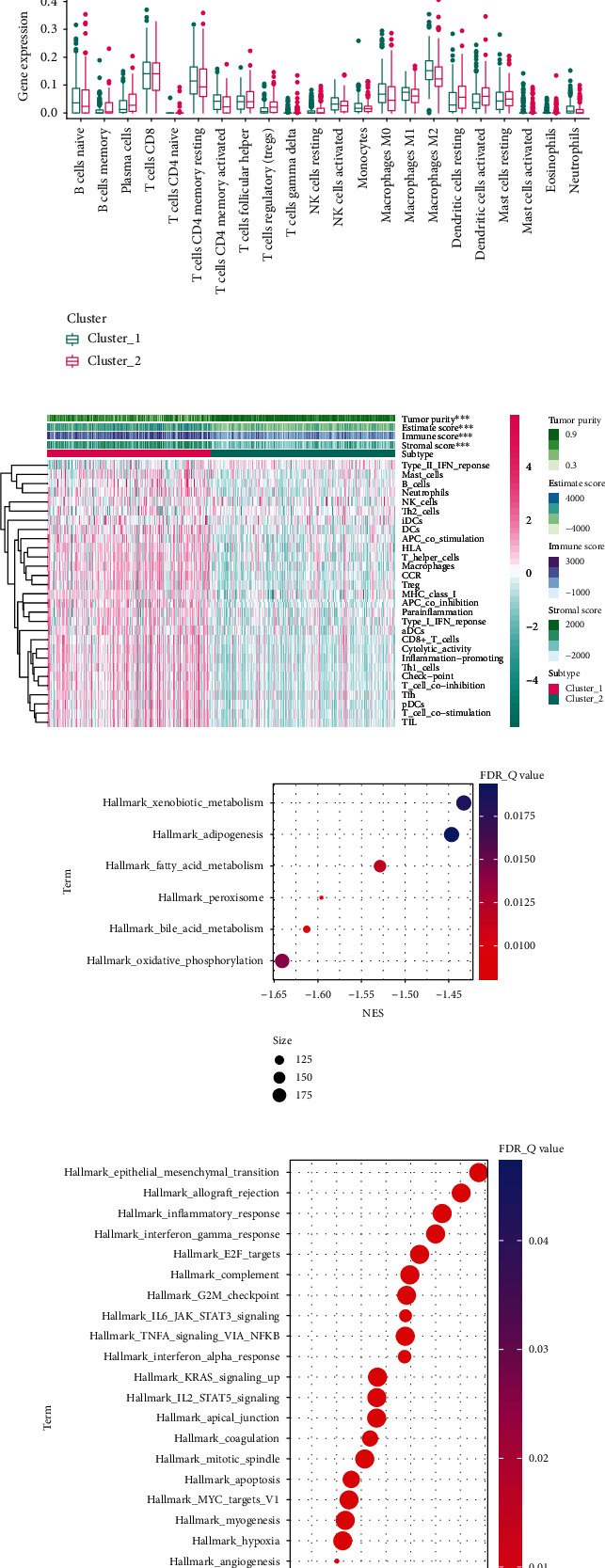
Association between ferroptosis and immune infiltration. (a) Among 121 ferroptosis regulators, 14 were upregulated and 6 were downregulated in BCa samples. (b) Consensus CDF values changed from *k* = 2 to *k* = 9. (c) Relative changes in area under CDF curve with different *k* values. (d) Consensus clustering heat map of 408 BCa cases when *k* = 2. (e) Scatter diagram displaying the PCA results of the expression of all genes when *k* = 2. (f) Kaplan–Meier survival analysis with log-rank test of BCa cases in different clusters (*p* < 0.05). (g) Box plot indicating the difference in infiltration proportion of 22 different immune cells between the two clusters. (h) Heat map of different immune-related pathways and functions. (i) GSEA for patients in cluster 1. (j) GSEA for patients in cluster 2. (k) Expression profiles of common immune checkpoints between different clusters. (l) Difference in immunotherapy response between the two clusters. (m) Patients in different clusters showed different chemotherapeutic sensitivity. BCa: bladder cancer; CDF: cumulative distribution function; PCA: principal component analysis; GSEA: gene set enrichment analysis; ∗∗∗*p* < 0.001.

From a recent review [5], 121 ferroptosis regulators were identified, and the mRNA expression of each gene was downloaded from the TCGA BCa dataset, which included 19 paracarcinoma tissues and 408 BCa samples. The edgeR package of R software (version 4.0.3) was used to detect the differentially expressed genes, and 20 ferroptosis regulatory factors were screened (Figure 1(a), Supplementary Table S1; ∣logFC | >1, FDR < 0.05). Through the consensus clustering algorithm, by which optimal grouping stability could be achieved, all enrolled cases were assigned into two groups: cluster 1 and cluster 2 (Figures 1(b)–1(d)). PCA indicated that our grouping method was reliable (Figure 1(e)). Notably, patients in cluster 1 had a significantly poorer prognosis than those in cluster 2 according to KM survival analysis (Figure 1(f)), *p* < 0.05). Mazdak et al. found that the serum iron ion level in 51 patients with BCa was significantly lower than that in 58 healthy controls, revealing the important role of iron in tumor development [21]. Subsequent studies verified that inhibition or activation of ferroptosis in BCa is a feasible measure of treatment effectiveness [22]. Generally, our analysis results again demonstrated the significant impact of ferroptosis on BCa from the perspective of big data analysis.

Some studies have reported that ferroptosis and tumor-infiltrating immune cells could influence each other [6, 7], prompting us to detect the changes in the immune landscape in different clusters. First, we estimated the infiltration of immune cells and the changes in immune-related pathways using ssGSEA based on the reported gene sets [23]. Accordingly, a different immune landscape was found in each ferroptosis cluster, which was verified by the ESTIMATE algorithm [13], a strategy used to predict the ratios of immune and stromal components of the TME (Figure 1(h)). GSEA showed that cases in cluster 1 were significantly enriched in metabolic pathways (Figure 1(i)), whereas cases in cluster 2 were mostly enriched in immune-related functions (Figure 1(j)). As ferroptosis-related clustering was immune-related, we detected the association between clustering and immune cell infiltration using the CIBERSORT algorithm [14]. We found that the different immune cells had different infiltration proportions between cluster 1 and cluster 2 according to the Wilcoxon signed-rank test (Figure 1(g)). Generally, patients in cluster 1 had a shorter survival time and relatively higher immune infiltration, indicating an immunosuppressive environment in this cluster.

Considering that immune checkpoints such as *PDL1*, *PD1*, *LAG3*, *CTLA4*, *TIM-3*, and *TIGIT* could lead to an immunosuppressive TME, we analyzed the association between ferroptosis-relevant clustering and the expression of immune checkpoints. We discovered that all of the abovementioned genes were significantly upregulated in cluster 1 (*p* < 0.001, Figure 1(k)). Immune checkpoints are also usually considered predictors of the immunotherapy response of patients with BCa, indicating that patients in cluster 1 were more likely to benefit from ICIs, which was again validated by the TIDE algorithm [18] (*p* < 0.001, Figure 1(l)). In addition to immunotherapy, we also detected the difference in chemotherapy effectiveness between the two clusters using the pRRophetic R package [19]. Patients in cluster 1 showed significantly higher chemotherapy sensitivity (Figure 1(m)). In summary, this analysis revealed the potential of ferroptosis-related genes to predict alterations in the immune infiltration of BCa.

### 3.2. Identification of Ferroptosis-Related lncRNAs (frlncRNAs)

**Figure 2:**
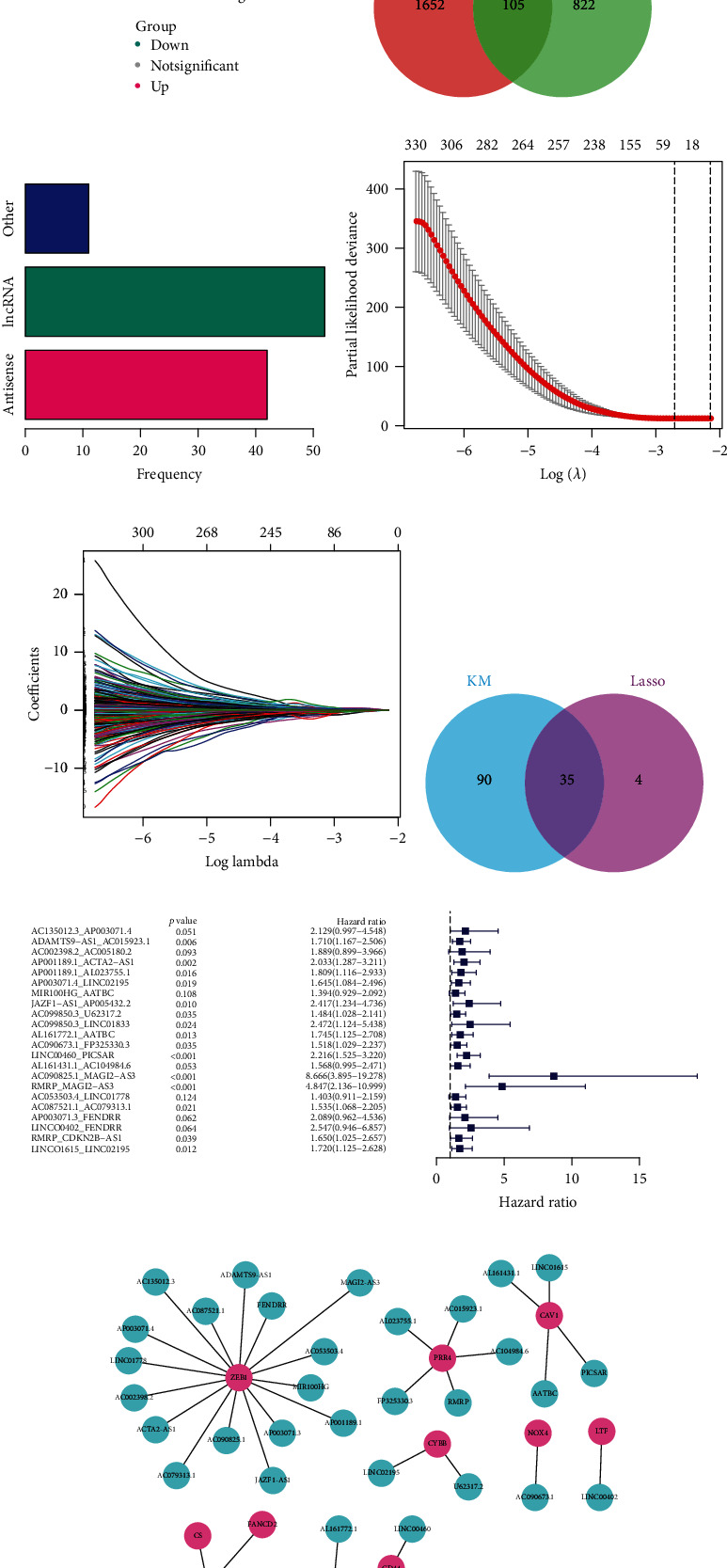
Construction of the lncRNA signature. (a) A total of 927 lncRNAs were differentially expressed between the BCa samples and paracarcinoma tissues. (b) A total of 105 overlapped lncRNAs codetermined by differential and coexpression analyses. (c) Long intervening noncoding RNAs and antisense lncRNAs accounted for most of the 105 lncRNAs. (d and e) Lasso–Cox regression with 10-fold cross-validation. (f) A total of 35 frlncRNA pairs codetermined with Kaplan–Meier survival analysis and Lasso-Cox regression. (g) A total of 22 frlncRNA pairs were included in the risk model using multivariate Cox regression with a stepwise method. (h) Network displaying the frlncRNAs and their possible target molecules, which serve as ferroptosis regulators. frlncRNA: ferroptosis-related lncRNA.

The transcriptome data of 408 BCa samples and 19 corresponding normal tissues from the TCGA website were analyzed. With the annotation file downloaded from Ensembl, all lncRNAs were selected for expression differential analysis using the edgeR package, and 927 lncRNAs were considered as differentially expressed genes with filtering criteria of FDR < 0.05 and ∣logFC | >2 (Figure 2(a)). Meanwhile, we calculated the Pearson correlation coefficients between each lncRNA and ferroptosis regulator, and the absolute values of Pearson *R* > 0.5 and *p* < 0.05 were determined to be significant (Supplementary Table S2). Ultimately, 105 lncRNAs codetermined by differential and coexpression analyses were identified as frlncRNAs (Figure 2(b)). Most of these 105 frlncRNAs were long intervening noncoding RNAs and antisense lncRNAs (Figure 2(c)).

### 3.3. Construction of frlncRNA Pairs and Prognostic Signature

Patients with a follow-up period of <30 days were excluded from the analysis. On the basis of the gene-pair strategy, 4298 frlncRNA pairs were identified. After KM survival analysis with a log-rank test, 125 gene pairs were extracted with *p* < 0.01 filtering (Supplementary Table S3), 35 of which were also determined by Lasso regression (Figures 2(d)–2(f)). Ultimately, 22 frlncRNA pairs were included in the prognostic model through multivariate Cox regression with a stepwise method (Figure 2(g)).  Figure 2(h) shows the frlncRNAs in the risk model and their possible targets.

### 3.4. Validation of the 22-frlncRNA-Pair Prognostic Model

**Figure 3:**
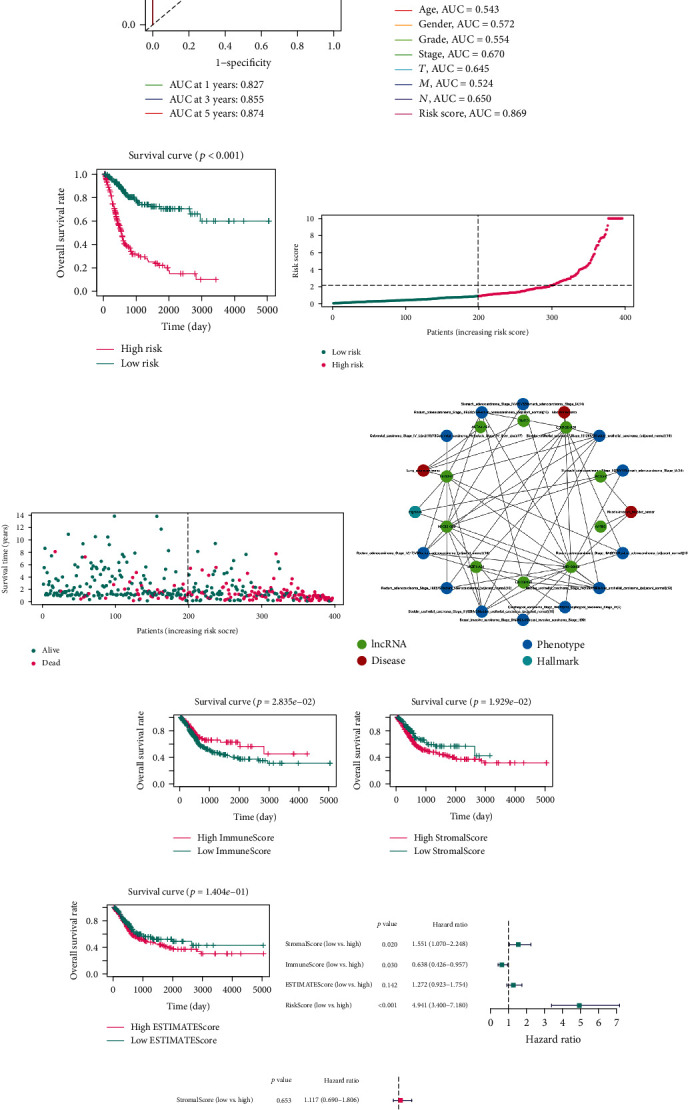
Validation of the lncRNA signature. (a) AUC of time-dependent ROC curves. (b) Comparison of 5-year ROC curves with other clinicopathological features. (c) Kaplan–Meier survival plot for OS in cases in the high- and low-risk groups (*p* < 0.001). (d and e) Scatter diagrams showing the risk scores (d) and survival status (e) of BCa cases. (f) Results of enrichment analysis using LncSEA. (g–i) Prognostic values of ImmuneScore (g, *p* < 0.05), StromalScore (h, *p* < 0.05), and ESTIMATEScore (i, *p* > 0.05). The optimal cutoffs were determined using X-tile software. (j–k) The risk score was superior to ImmuneScore, StromalScore, and ESTIMATEScore in predicting OS in univariate (j) and multivariate Cox analyses (k). AUC: area under curve; ROC: receiver operating characteristic; OS: overall survival; BCa: bladder cancer.

**Figure 4:**
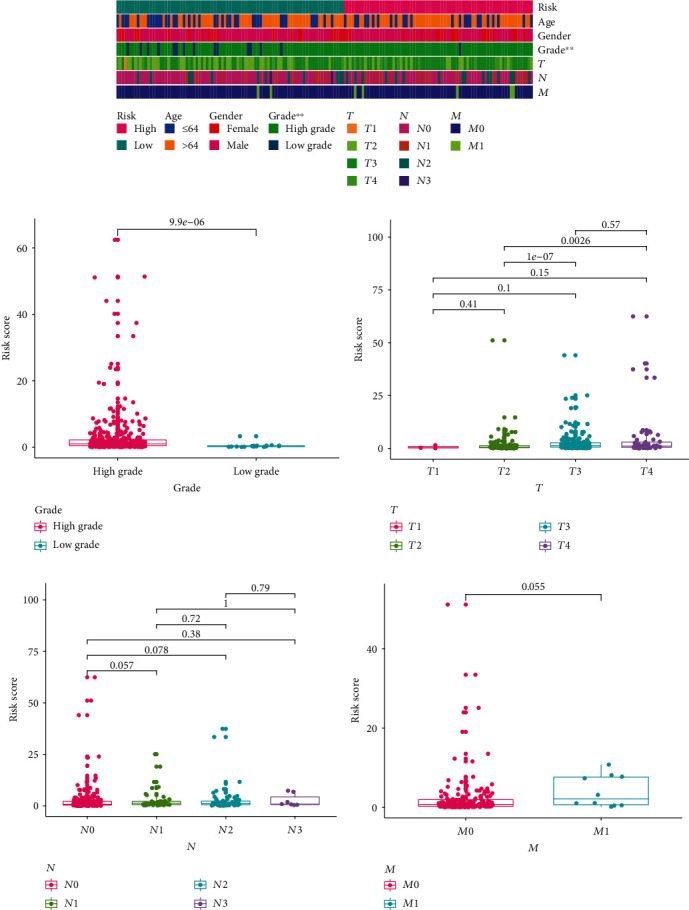
A strip chart (a) and box plots (b–f) showing the association between the risk score and tumor grades (b), pathological *T* stage (c), *N* stage (d), and *M* stage (e).

Various methods were implemented to determine the prognostic value of the established model. First, we generated the 1-, 3-, and 5-year receiver operating curves (ROCs) of the gene-pair signature and found that the AUCs were all >0.8 (Figure 3(a)). After excluding cases without definite TNM stages, we compared the AUCs for the 5-year ROC curves of different clinicopathological parameters and risk scores (Figure 3(b)). According to the median value of the risk score, 197 cases were categorized into the high-risk group and 199 cases were classified as the low-risk group. The high-risk subgroup had a significantly poorer clinical outcome than the low-risk subgroup (*p* < 0.001, Figure 3(c)). Higher risk scores were associated with more deaths and shorter survival time (Figures 3(d) and 3(e)). Furthermore, univariate and multivariate Cox regression analyses were conducted, and the results revealed that the risk score was an independent prognostic predictor of OS (Table 1). In addition, we found that the established model was superior to ImmuneScore, StromalScore, and ESTIMATEScore in predicting prognosis in BCa, which could partly reflect the level of tumor immune infiltration (Figures 3(g)–3(k)). We also randomly resampled 30% of cases from the training set as the internal validation dataset, and the risk model also showed high efficacy in distinguishing cases with a low risk from those with a high risk (Supplementary Figure S1). To validate the robustness of the frlncRNA signature, we conducted KM survival estimations in multiple subgroups according to diverse clinicopathological features (Supplementary Figure S2). In addition, we evaluated the clinical associations of the risk score. We compared the risk scores between various tumor grades and TNM stages using the chi-square test (Figure 4(a)) and Wilcoxon signed-rank test (Figures 4(b)–4(e)).

### 3.5. Functional Enrichment of the 22-frlncRNA Pair

We implemented LncSEA [24], an online tool developed for lncRNA enrichment analysis, to primarily assess the related functions (Figure 3(f) and Supplementary Table S4). In the risk model, 36 frlncRNAs were mostly enriched in BCa-related functions, indicating that the signature had a degree of BCa specificity.

### 3.6. The Immune Infiltration Landscape and Immunotherapy Results between High- and Low-Risk Groups

**Figure 5:**
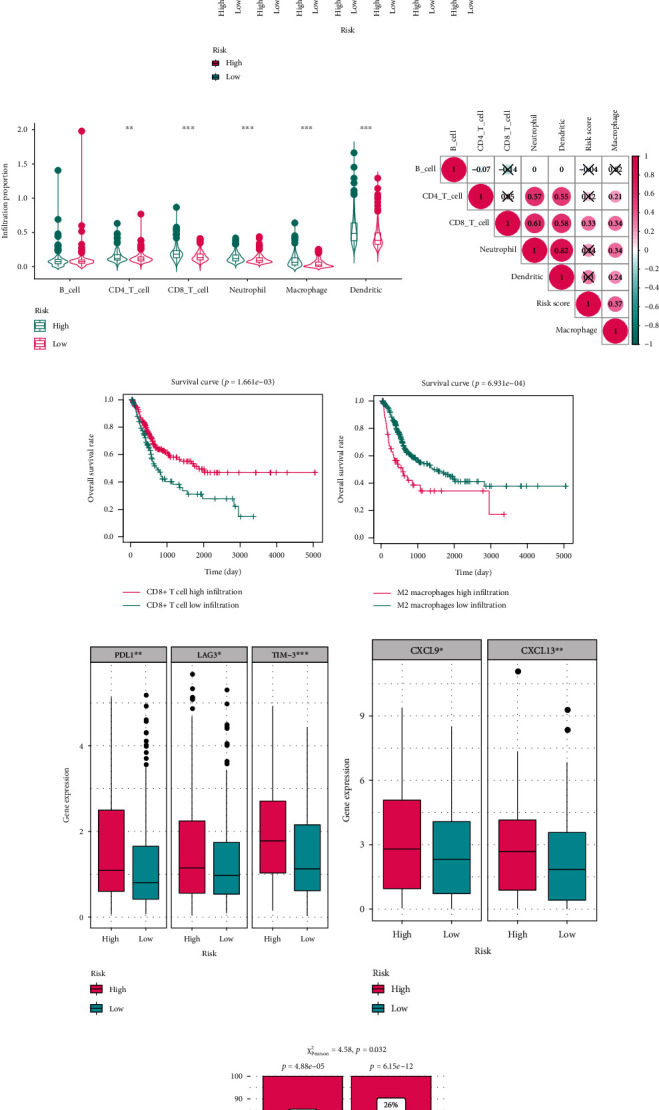
Evaluation of the predictive ability for immune infiltration and immunotherapy response of the risk model. (a) Cases in the high-risk group had a higher StromalScore, ImmuneScore, and ESTIMATEScore. (b) Heat map showing different immune and inflammatory profiles in the high- and low-risk group. (c) GSVA scores of seven clusters of immune and inflammatory genes in the high- and low-risk groups. (d) Infiltration ratios of six immune cells in different risk groups. (e) The risk score was positively correlated with the infiltration levels of CD8+ T cells and macrophages. (f) Patients with BCa with a low infiltration level of CD8+ T cells had unfavorable clinical outcomes (*p* < 0.01). (g) Patients with BCa with a high infiltration level of M2 macrophages had poorer survival (*p* < 0.001). (h) *PDL-1*, *LAG3*, and *TIM-3* were differentially expressed between the high- and low-risk groups. (i) The expression levels of *CXCL9* and *CXCL13* in the high-risk group were significantly higher than those in the low-risk group according to the Wilcoxon test. (j) The chi-square test indicated that cases in the high-risk group might more likely benefit from immunotherapy. GSVA: gene set variation analysis.

Various methods were implemented to estimate the immune profiles of cases in the low- and high-risk subgroups. On the one hand, the Wilcoxon signed-rank test indicated that the StromalScore, ImmuneScore, and ESTIMATEScore were significantly higher in the high-risk cases according to the ESTIMATE algorithm (Figure 5(a)), implying that high-risk patients might have a more active TME. On the other hand, seven clusters of immune and inflammatory genes were collected from a previous study [25], and GSVA was implemented to evaluate the immune and inflammatory response enrichment status (Figure 5(b)). Except for interferon (*IFN*) and *MHC-I*, *IgG*, *HCK*, *MHC-II*, *LCK*, and *STAT1* were all positively correlated with the risk score according to the Wilcoxon test (Figure 5(c), *p* < 0.05). Tumor immune heterogeneity might account for the disconnected relationship between *IFN* and *MHC-I* and the risk score.

Considering that the risk score was significantly correlated with tumor immune activity, we analyzed the infiltration proportion of immune cells. The TIMER algorithm [15], which is similar to CIBERSORT, was developed to estimate the infiltration levels of different immune cells, including B cells, CD4 T cells, CD8 T cells, neutrophils, dendritic cells, and macrophages. The Wilcoxon signed-rank test indicated that only B cells showed no significant association with risk (Figure 5(d), *p* < 0.05), whereas the Spearman correlation test revealed that CD8 T cells and macrophages were positively correlated with the risk score (Figure 5(e), *p* < 0.05). We also evaluated the prognostic values of the infiltration levels of CD8+ T cells and M2 macrophages, which have been reported to be closely associated with ferroptosis in many other malignant tumors [6, 7], via CIBERSORT algorithm and KM survival analysis with log-rank test in BCa, and X-tile was used to determine the optimal cut-off. The analyses results showed the patients exhibiting worse OS carried high CD8+ T cell infiltration (Figure 5(f), *p* < 0.01) and low M2 macrophage infiltration (Figure 5(g), *p* < 0.001).

The strong linkage between risk score and immune infiltration prompted us to investigate whether the risk score could serve as an indicator of the response to ICIs. First, we examined the expression difference of common immune checkpoint molecules and discovered that *PDL1*, *LAG3*, and *TIM-3* were upregulated in the high-risk cohort according to the Wilcoxon test (Figure 5(h), *p* < 0.05). We also found the expression level of *CXCL9* and *CXCL13* was significantly associated with the risk score (Figure 5(i), *p* < 0.05). In addition, using the TIDE algorithm, we predicted the immunotherapy clinical outcomes of the low- and high-risk cohorts. The chi-square test revealed a significant difference (Figure 5(j), *p* < 0.05). Overall, we believe that the prognosis model could also be a potential indicator of the effectiveness of immunotherapy.

### 3.7. The Linkage between Chemotherapy and Risk Score

**Figure 6:**
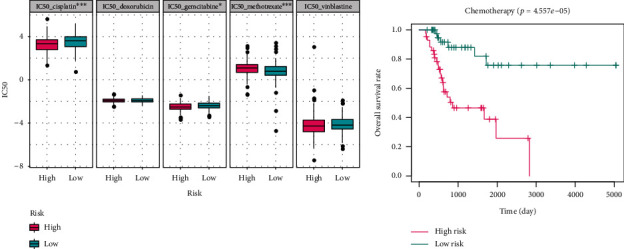
Evaluation of the predictive ability for chemotherapy effectiveness of the risk model. (a) Correlation analysis revealed that the risk score was significantly correlated with the IC50 values of cisplatin, gemcitabine, and methotrexate. (b) Patients with a high-risk score showed worse prognosis among those who received chemotherapy (*p* < 0.001). IC50: half inhibitory concentration.

Emerging evidence suggests that some types of chemotherapeutic drugs could induce malignant cancer cells to express damage-associated molecular patterns that bind to antigen-presenting cells, trigger a tumor-specific immune response, and enhance the immunotherapeutic sensitivity of a malignant tumor [26]. Considering the above findings, we analyzed the association between risk and chemotherapy sensitivity and found that the IC50 values of cisplatin and gemcitabine were negatively correlated with risk (Figure 6(a)), according to the Wilcoxon test, whereas the IC50 value of methotrexate had a positive association (Figure 6(a)), implying that the risk model has a potential to predict the efficacy of chemotherapy for BCa. The evaluated risk score could also serve as a prognostic biomarker for patients who had received chemotherapy (*p* < 0.001, Figure 6(b)).

### 3.8. Analysis of Iron Metabolic Pathways

**Figure 7:**
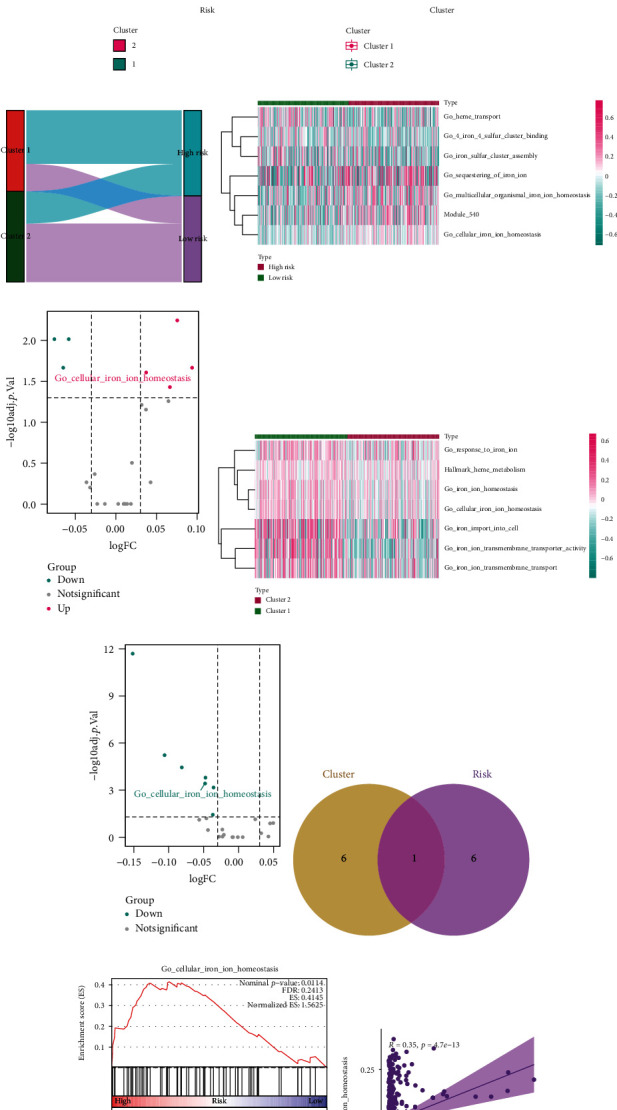
Enrichment analysis of iron metabolic pathways. (a) Chi-square test indicated that ferroptotic clustering and risk grouping were highly correlated. (b) Patients in cluster 1 had higher risk scores. (d) Sankey diagram showing the close association between ferroptotic clustering and risk grouping. (d and e) Heat map (d) and volcano diagram (e) indicating that seven iron metabolic pathways were changed with risk. (f and g) Heat map (f) and volcano diagram (g) showing that seven iron metabolic pathways were differentially enriched in cluster 1 and cluster 2. (h) Venn plot showing overlapping of cellular iron ion homeostasis. (i) GSEA of cellular iron ion homeostasis in the high-risk group. (j) The risk score was positively correlated with the GSVA score of cellular iron ion homeostasis. GSEA: gene set enrichment analysis; GSVA: gene set variation analysis.

Under normal conditions, intracellular iron balance is maintained through the iron transport system. Increasing the uptake of iron ions or decreasing their efflux can enhance the sensitivity of cancer cells to oxidative damage and ferroptosis [27]. Hence, we analyzed the changes in iron metabolism-related pathways and functions. Although the chi-square test and Wilcoxon signed-rank test revealed that ferroptosis-related clustering and frlncRNA-based risk grouping were highly associated (Figures 7(a)–7(c)), the changes in iron metabolism-related pathways were mostly different according to GSVA (Figures 7(d)–7(g), Supplementary Tables S5 and S6). It is noteworthy that the GSVA scores of cellular iron ion homeostasis were significantly altered in both risk grouping and ferroptosis clustering (Figure 7(h)), and the results were validated using GSEA (Figure 7(i)). The significantly positive Spearman correlation between the GSVA score and the risk score verified the importance of maintaining cellular iron homeostasis for the occurrence of ferroptosis (Figure 7(j)), also validating our risk model as ferroptosis related.

### 3.9. The Functional Prediction of the Crucial frlncRNA Pair

The gene-pair strategy focuses on the relative expression levels of two genes, and high prognostic and diagnostic values of the relative expression level imply that the two genes might be involved in the same molecular mechanism through a mutual interaction. The present study investigated the mechanisms using *AC090825.1* and *MAGI2-AS3*, which comprised the frlncRNA pair with the highest hazard ratio, as examples. Despite the relatively lower expression of *AC090825.1* and *MAGI2-AS3* in BCa samples (Supplementary Figure 3a, b), the difference was significantly higher than that in paracarcinoma tissues (Supplementary Figure 3c). In addition, we found that patients had poorer survival when *AC090825.1* was more highly expressed than *MAGI2-AS3* (Supplementary Figure 3d). Moreover, *AC090825.1* and *MAGI2-AS3* expression had a significant association in both paracarcinoma tissues and BCa samples (Supplementary Figure 3e, *p* < 0.05). Recent studies have postulated that lncRNAs regulate their target genes by interacting with transcription factors. Accordingly, we predicted the transcription factor of frlncRNAs and their target genes (Supplementary Tables S7–S10) and constructed a regulatory network (Supplementary Figure 3f). *HOXD4*, *ZNF354C*, *GSX2*, *NR2C2*, *MEIS1*, and *HOXC4* were codetermined by the transcription factor prediction (Supplementary Figure 3g) and coexpressed with two frlncRNAs (Supplementary Figure 3h, *R* > 0.3, *p* < 0.05), implying the possible biological functions of *AC090825.1* and *MAGI2-AS3*.

## 4. Discussion

In recent years, several models have been proposed to predict the prognosis and immune infiltration of malignancies [28]. However, few studies have focused on gene-pair modeling methods, which do not require exact gene expression levels. In addition, most studies chose to screen immune-related genes to predict the immune infiltration of malignancies, whereas few studies used ferroptosis-related genes to predict the immune infiltration status despite several reports indicating that ferroptosis and the TME could affect each other. In this study, frlncRNAs were detected to construct a rational model based on the gene-pair strategy to predict the prognosis and tumor immune reaction of BCa for the first time.

First, we initially identified 121 ferroptosis regulators from a previous study [5] and extracted transcriptome data from the TCGA BCa dataset. After unsupervised clustering, all enrolled cases were divided into two subgroups. We found that the subgroups had different prognoses, immune infiltration, immunotherapy response, and chemotherapy outcomes, implying that ferroptosis was closely correlated with the TME in BCa, thus, supporting the relevance of constructing an frlncRNA signature to predict tumor immune infiltration in future studies. Furthermore, all lncRNAs were extracted, and frlncRNAs were determined through genomic difference analysis and Pearson coefficient calculation. After pairing and differential analysis of frlncRNAs, we ultimately built a 0-or-1 matrix. Using KM survival analysis, the Lasso algorithm, and multivariate Cox regression, 22 frlncRNA pairs were identified. KM plot analysis, ROC curve analysis, random sampling validation, and subgroup analysis were performed for model verification and showed that the novel signature is a powerful tool for BCa prognosis prediction. Moreover, it was found that the risk score calculated using the frlncRNA signature was significantly correlated with immune infiltration, immunotherapeutic effectiveness, and chemotherapeutic results. GSVA showed that cellular iron homeostasis imbalance, which is routinely described as one of the key mechanisms of ferroptosis, was the crucial iron metabolism-related pathway in different subgroups, validating the clustering strategy and that the constructed risk model is ferroptosis related.

**Figure 8:**
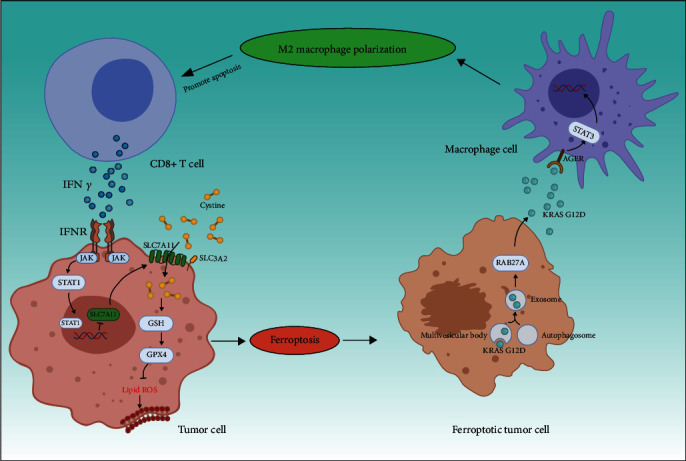
Dual role of ferroptosis in tumor immunity.

Some researchers have contributed to the construction of lncRNA signatures for predicting the immune infiltration profiles of BCa. Wu et al. identified eight immune-related lncRNAs and found that the risk score was positively correlated with poor clinical outcomes and high immune infiltration [29]. By using the ESTIMATE algorithm and correlation analysis, Yuan et al. constructed an immune-related lncRNA signature to evaluate immune cell infiltration in the TME [30]. The use of lncRNAs to predict tumor immune infiltration is an effective strategy because of the wide participation of lncRNAs in biological processes [31]. Most studies have constructed lncRNA diagnosis models based on immune-related genes or algorithms, which is a reasonable and logical method. However, emerging evidence has revealed a strong association between ferroptosis and tumor immune reactions. For instance, Wang et al. found that IFN*γ* released from CD8+ T cells could suppress the expression of *SLC7A11*, thereby promoting lipid peroxidation in cancer cells and inducing ferroptosis [6] (Figure 8). In addition, it was reported that extracellular KRAS protein released by ferroptotic tumor cells and taken up by macrophages contributes to M2 macrophage polarization, thus, resulting in the development of pancreatic tumor cells [7] (Figure 8). Similarly, a low infiltration level of CD8+ T cells and a high infiltration level of M2 macrophages were found to be associated with poor prognosis in patients with BCa (Figures 5(f) and 5(g)), and the infiltration ratio was significantly different between the high- and low-risk groups (Figure 5(d)), suggesting that the same biological mechanisms might exist in BCa. The close relationship between ferroptotic cell death and the TME prompted us to develop a ferroptosis-linked lncRNA model for predicting prognosis and diagnosing immune infiltration of BCa.

The frlncRNA signature is an effective and practical tool for predicting the prognosis of patients with BCa. Compared with other clinical features, the novel model was able to distinguish cases with a high or low risk with higher efficacy. Univariate and multivariate analyses revealed that the risk score was an independent prognostic predictor. Random resampling verification, clinical parameter association analysis, and subgroup analysis validated the robustness of the model. Notably, we used a gene-pair strategy instead of detecting the exact expression level to establish the predictive model, which only required examining which gene in the pair had a higher expression, thus, extending the application of the risk model.

Among the lncRNAs in the frlncRNA signature, several have been demonstrated to be related to immunity, ferroptosis, and malignancy. For instance, ADAMTS9-AS1, which was reported to suppress the malignant phenotypes of breast cancer cells [32], could also regulate colorectal cancer cell proliferation and migration [33]. The gene-pair strategy also helped disclose the underlying association between the paired lncRNAs. AC090825.1 and MAGI2-AS3, comprising the gene pair with the highest hazard ratio in the model, were strongly correlated based on RNA expression levels in both paracarcinoma tissues and tumor samples (Supplementary Figure 3e). Although it has been reported that MAGI2-AS3 could inhibit the development of BCa cells [34], the interplay between AC090825.1 and MAGI2-AS3 is still unknown. Functional prediction analysis indicated that AC090825.1 and MAGI2-AS3 might regulate their targets, *NOX4* and *ZEB1*, by interacting with the transcription factors (Supplementary Figure 3f). Overall, our model helped in identifying new biomarkers and in proposing novel mechanisms for BCa.

To our knowledge, this is the first study to use frlncRNAs to predict the immune landscape in BCa. In addition, this study is the first to use the gene-pair strategy to construct a lncRNA signature for predicting the clinical outcomes of BCa. From the perspective of model performance, the established model had the highest AUC values among the known prognostic models for BCa. We also identified dozens of novel lncRNA biomarkers, such as AC090825.1, which may be useful in further studies.

However, the present study had several limitations. The analyzed raw data were all downloaded from TCGA, and the external validation of the established model was inadequate. As a general rule, validation in a different cohort is required for a diagnosis or prognosis model because of the individual variations in gene expression. To reduce errors caused by gene expression differences, we innovatively used the gene-pair modeling method to construct an frlncRNA signature. Random resampling, subgroup analysis, and correlation analysis of clinical risk parameters were conducted to validate the robustness of our model. Although external validation was insufficient, these methods and the evidence suggested that the novel model was acceptable. However, external validation in other BCa cohorts is warranted.

## 5. Conclusions

We developed a novel lncRNA signature to predict the prognosis and immune landscape of BCa based on the gene-pair strategy and ferroptosis-related genes. This lncRNA signature provides new clues for identifying the regulatory relationship between ferroptosis and the TME and can help clinicians estimate the prognosis, immunotherapy outcomes, and chemotherapy response of patients with BCa.

## Figures and Tables

**Table 1:** Univariate and multivariate Cox analysis of the risk score.

Covariates	Univariate analysis		Multivariate analysis	
	HR (95% CI)	*p* value	HR (95% CI)	*p* value
Age (≤64 vs. >64)	1.81 (0.97-3.37)	0.06	2.04 (1.07-3.89)	0.03
Gender (female vs. male)	1.62 (0.93-2.84)	0.09	1.40 (0.79-2.47)	0.24
Grade (low vs. high)	2.81e+07 (0-∞)	1.00	5.16e+06 (0-∞)	1.00
*T* (*T* 1-2 vs. *T* 3-4)	2.61 (1.28-5.33)	0.01	1.66 (0.79-3.50)	0.18
*N* (*N*0 vs. *N*1-3)	2.31 (1.37-3.89)	<0.01	2.41 (1.36-4.28)	<0.01
*M* (*M*0 vs. *M*1)	2.17 (0.78-6.03)	0.14	0.87 (0.30-2.55)	0.80
Risk score (low vs. high)	6.47 (3.40-12.32)	<0.01	6.41 (3.31-12.42)	<0.01

HR: hazard ratio; CI: confidence interval.

## Data Availability

The data used to support the findings of this study are available from The Cancer Genome Atlas (TCGA, https://portal.gdc.cancer.gov/), and the code would be supplied from the corresponding author upon request.

## Supplementary Materials

Supplementary Materials Supplementary Figure S1: validation of the risk model with resampling. Note: (a) Kaplan-Meier survival analysis of cases in high-risk group and low-risk group (*p* < 0.001). (b) ROC curves of the risk score in internal validation dataset. Supplementary Figure S2: Kaplan-Meier subgroup survival analysis in TCGA-BLCA patients. Note: (a) age ≤ 64 (*p* < 0.001). (b) Age > 64 (*p* < 0.001). (c) Male (*p* < 0.001). (d) Female (*p* < 0.001). (e) Pathological *T* 1-2 stages (*p* < 0.001). (f) Pathological *T* 3-4 stages (*p* < 0.001). (g) *N*0 stages (*p* < 0.001). (h) *N*1-*N*3 stages (*p* < 0.001). (i) *M*0 stage (*p* < 0.001). (j) *M*1 stage (*p* < 0.05). Supplementary Figure S3: functional prediction of AC090825.1 and MAGI2-AS3. Note: (a) the expression difference of AC090825.1 in BCa samples and paracarcinoma tissue via Wilcoxon signed-rank test. (b) The expression difference of MAGI2-AS3 in BCa samples and paracarcinoma tissue via Wilcoxon signed-rank test. (c) The difference values between the expression of AC090825.1 and MAGI2-AS3 in BCa samples and paracarcinoma samples. (d) The cases suffered poorer survival when the expression value of AC090825.1 was higher than that of MAGI2-AS3 (*p* < 0.01). (e) The expression levels of AC090825.1 and MAGI2-AS3 were positively correlated both in BCa samples and paracarcinoma samples. (f) Network of lncRNAs, the target genes and their possible transcription factors. (g) UpSet diagram showing 6 overlapped transcription factors. (h) The correlation network of lncRNAs, the target genes and transcription factors. BCa: bladder cancer. Supplementary Table S1: 20 ferroptosis regulators were differentially expressed between BCa and paracarinoma samples. Supplementary Table S2: the Pearson correlation analysis of lncRNAs and ferroptosis regulators. Supplementary Table S3: Kaplan-Meier survival analysis of the lncRNA pairs. Supplementary Table S4: enrichment results from LncSEA website. Supplementary Table S5: GSVA results of different clusters based on ferroptosis regulators. Supplementary Table S6: GSVA results of different risk subgroups based on frlncRNA signature. Supplementary Table S7: the prediction of the transcription factors of AC090825.1. Supplementary Table S8: the prediction of the transcription factors of MAGI2-AS3. Supplementary Table S9: the prediction of the transcription factors of NOX4. Supplementary Table S10: the prediction of the transcription factors of ZEB1.
