# Supplementary material for: Development of a Ferroptosis-Related lncRNA Signature to Predict the Prognosis and Immune Landscape of Bladder Cancer
Source: Dis Markers. 2021 Jun 20;2021:1031906. doi: 10.1155/2021/1031906 (PMC8238626; doi:10.1155/2021/1031906)
Supplement: Supplementary Materials — Supplementary Figure S1: validation of the risk model with resampling. Note: (a) Kaplan-Meier survival analysis of cases in high-risk group and low-risk group (p < 0.001). (b) ROC curves of the risk score in internal validation dataset. Supplementary Figure S2: Kaplan-Meier subgroup survival analysis in TCGA-BLCA patients. Note: (a) age ≤ 64 (p < 0.001). (b) Age > 64 (p < 0.001). (c) Male (p < 0.001). (d) Female (p < 0.001). (e) Pathological T 1-2 stages (p < 0.001). (f) Pathological T 3-4 stages (p < 0.001). (g) N0 stages (p < 0.001). (h) N1-N3 stages (p < 0.001). (i) M0 stage (p < 0.001). (j) M1 stage (p < 0.05). Supplementary Figure S3: functional prediction of AC090825.1 and MAGI2-AS3. Note: (a) the expression difference of AC090825.1 in BCa samples and paracarcinoma tissue via Wilcoxon signed-rank test. (b) The expression difference of MAGI2-AS3 in BCa samples and paracarcinoma tissue via Wilcoxon signed-rank test. (c) The difference values between the expression of AC090825.1 and MAGI2-AS3 in BCa samples and paracarcinoma samples. (d) The cases suffered poorer survival when the expression value of AC090825.1 was higher than that of MAGI2-AS3 (p < 0.01). (e) The expression levels of AC090825.1 and MAGI2-AS3 were positively correlated both in BCa samples and paracarcinoma samples. (f) Network of lncRNAs, the target genes and their possible transcription factors. (g) UpSet diagram showing 6 overlapped transcription factors. (h) The correlation network of lncRNAs, the target genes and transcription factors. BCa: bladder cancer. Supplementary Table S1: 20 ferroptosis regulators were differentially expressed between BCa and paracarinoma samples. Supplementary Table S2: the Pearson correlation analysis of lncRNAs and ferroptosis regulators. Supplementary Table S3: Kaplan-Meier survival analysis of the lncRNA pairs. Supplementary Table S4: enrichment results from LncSEA website. Supplementary Table S5: GSVA results of different clusters based on ferroptosis regulators [file 1031906.f1.zip › Supplementary_Table_S1.PDF]

Supplementary Table S1. 20 ferroptosis regulators were differentially expressed between BCa and paracarinoma samples.

gene	logFC	logCPM	PValue	FDR
ORAI1	0.344829075	4.910796787	6.29E-02	1.04E-01
ATF3	-3.066643529	6.824484612	3.40E-33	2.82E-31
BAP1	0.11734232	6.406181595	3.28E-01	4.16E-01
CDO1	-1.864375412	0.766890655	1.15E-05	5.84E-05
TP53	0.413248331	6.357973428	6.40E-02	1.06E-01
ATM	-0.481319485	5.139139396	2.21E-03	5.92E-03
ZEB1	-2.849320493	4.506179225	2.62E-40	3.50E-38
DDIT3	0.384344005	4.574685679	9.65E-02	1.50E-01
GLS2	0.17175121	-0.407600465	6.37E-01	7.09E-01
GOT1	0.02510147	5.530909769	8.96E-01	9.24E-01
SLC38A1	0.968100717	7.698942811	2.12E-06	1.28E-05
ABCC1	0.089161974	6.558697752	7.34E-01	7.93E-01
HMOX1	1.011111745	6.184530634	1.11E-02	2.37E-02
IREB2	0.14527421	5.563721158	2.81E-01	3.65E-01
TF	1.774311513	4.319840577	5.70E-02	9.59E-02
TFRC	1.210513669	7.925259245	4.24E-05	1.87E-04
LTF	1.868429934	6.687419556	6.50E-02	1.07E-01
BRAF	-0.032863272	5.286957098	8.23E-01	8.66E-01
KRAS	0.090580896	5.835047139	6.40E-01	7.12E-01
ACSL4	-0.138645128	5.843206003	5.08E-01	5.92E-01
ALOX12	-1.991909239	2.949184475	1.06E-09	1.19E-08
ALOX15B	-2.2071967	2.752588354	7.80E-09	7.59E-08
ALOXE3	1.58665713	0.973107149	5.17E-03	1.23E-02
CS	0.070748361	7.276540997	5.45E-01	6.26E-01
CSF2	2.800483241	0.318208061	1.31E-04	5.07E-04
EPAS1	-0.232395365	8.157678408	2.78E-01	3.62E-01
HILPDA	1.775148938	5.773826049	1.02E-06	6.55E-06
LPCAT3	0.389449541	4.617519953	1.03E-01	1.58E-01
MDM2	0.89378648	7.410517515	1.45E-02	2.99E-02
PEBP1	0.351464135	8.799887905	1.02E-01	1.58E-01
POR	0.543167605	7.253421256	1.16E-03	3.39E-03
SAT1	0.41021205	8.248085639	1.02E-01	1.58E-01
SQLE	1.533239027	6.48444592	2.31E-08	2.06E-07
YAP1	-0.588496473	7.104576134	7.77E-03	1.75E-02
ATG3	0.259920908	5.609441001	2.48E-02	4.72E-02
ATG5	0.125137345	5.320247659	3.64E-01	4.54E-01
ATG7	0.49126391	5.103292891	1.39E-03	3.99E-03
ATG13	0.080020395	6.169236597	5.54E-01	6.35E-01
ATG16L1	-0.218495942	4.803312174	4.34E-02	7.59E-02
BECN1	-0.1438908	5.808788644	2.39E-01	3.20E-01
ELAVL1	0.40337972	6.692891387	7.46E-06	3.96E-05

MAP1LC3B	-0.372122753	6.217826173	1.14E-03	3.35E-03
NCOA4	-0.505110718	7.267148811	1.98E-04	7.25E-04
PINK1	-0.562152811	4.742207667	9.76E-05	3.90E-04
RAB7A	0.326890016	8.256005688	3.07E-03	7.83E-03
SQSTM1	-0.530185823	8.071621249	1.25E-03	3.63E-03
STAT3	-0.483006618	7.646691398	4.00E-04	1.35E-03
CTSB	0.469897095	10.14758604	7.69E-02	1.24E-01
ULK1	0.353613689	6.327455695	2.73E-02	5.12E-02
ULK2	-0.469777628	4.396511561	1.01E-02	2.19E-02
BBC3	1.204867525	4.178921265	3.44E-07	2.45E-06
BID	1.300843404	5.9546214	1.43E-11	2.10E-10
VDAC2	-0.487962115	6.144715047	5.38E-04	1.74E-03
VDAC3	0.051108257	6.65167087	7.43E-01	8.01E-01
ACVR1B	0.427002371	5.638434381	2.04E-02	4.01E-02
CDKN2A	4.097475476	5.999591235	3.40E-07	2.42E-06
EIF2AK2	0.495833552	6.415571846	2.31E-03	6.16E-03
KEAP1	0.323071415	6.717295166	1.19E-02	2.52E-02
TGFBR1	-0.310619308	5.81931071	1.40E-01	2.05E-01
CYBB	0.208974807	5.128160971	6.79E-01	7.47E-01
DPP4	1.048488916	3.373443059	5.74E-02	9.64E-02
HDDC3	0.587503621	3.488138825	5.15E-04	1.68E-03
NOX1	0.956935067	1.843197336	2.62E-02	4.94E-02
NOX4	0.728661904	1.804678011	4.20E-02	7.39E-02
AKR1C1	0.403930711	6.79297729	5.37E-01	6.20E-01
AKR1C2	0.098618306	7.943417552	9.38E-01	9.57E-01
AKR1C3	0.221454211	7.468457069	7.14E-01	7.77E-01
GCH1	-0.436451265	4.359561068	1.53E-02	3.14E-02
CDH1	0.878099842	9.037407125	6.51E-03	1.50E-02
ITGA6	-0.055414001	7.805947338	8.09E-01	8.55E-01
ITGB4	1.065002281	9.280348345	1.03E-03	3.08E-03
NF2	0.163120195	5.588805953	3.20E-01	4.07E-01
AIFM2	0.267554053	4.840548034	2.04E-01	2.81E-01
COQ2	0.407268198	3.62480621	3.07E-02	5.66E-02
CD44	0.020261852	8.775876049	9.90E-01	9.95E-01
MUC1	1.590159678	8.176358799	1.86E-03	5.11E-03
OTUB1	0.680312379	7.091222928	8.31E-07	5.45E-06
SLC3A2	0.984103014	7.849670098	1.93E-05	9.31E-05
SLC7A11	0.638974341	5.166697078	3.22E-01	4.09E-01
FANCD2	1.770481985	4.997335953	2.21E-15	5.10E-14
TFAM	0.443463634	5.400538393	1.54E-03	4.36E-03
ATF4	-0.375262344	8.287404312	4.77E-03	1.15E-02
HSPA5	0.695661912	9.480086965	7.09E-06	3.79E-05
GCLC	-0.153649106	6.748551279	5.64E-01	6.44E-01
CISD1	-0.288014783	4.435463628	6.60E-02	1.09E-01

CISD2	0.687242671	5.035506183	3.94E-07	2.77E-06
FTH1	0.173674902	8.997736978	3.73E-01	4.62E-01
FTMT	2.735921281	-3.6376038	7.59E-02	1.22E-01
HSPB1	-0.116917774	10.56296462	6.07E-01	6.83E-01
ISCU	-0.433059183	6.24924552	7.95E-05	3.25E-04
PROM2	0.74213003	8.037098764	5.18E-02	8.84E-02
SLC40A1	-0.398576248	5.957651451	1.78E-01	2.50E-01
ACSL3	0.047527883	6.552969779	8.01E-01	8.47E-01
FDFT1	0.503808547	7.512183198	2.12E-02	4.14E-02
GPX4	0.373964276	7.886363424	3.57E-02	6.43E-02
HIF1A	0.15877897	7.64067462	5.15E-01	5.99E-01
PLIN2	0.04990445	6.595999451	9.06E-01	9.32E-01
SCD	1.90196809	8.898506114	2.89E-06	1.69E-05
TPD52	0.447819776	6.213272433	3.09E-02	5.69E-02
VHL	0.977137513	5.854479495	1.23E-07	9.55E-07
ARNTL	-0.168133358	2.953944362	3.44E-01	4.33E-01
CHMP5	0.240941238	6.379238354	8.65E-02	1.36E-01
CHMP6	0.18559512	4.772836803	2.36E-01	3.16E-01
NEDD4	-0.188997308	4.374552416	3.27E-01	4.15E-01
MT1G	0.831900305	2.561283376	1.42E-01	2.07E-01
NFE2L2	-0.826287265	7.231005935	2.10E-08	1.89E-07
NQO1	0.468613655	7.742211889	1.99E-01	2.75E-01
PRDX1	0.341622706	8.640331089	5.59E-02	9.42E-02
PRDX5	0.377755427	8.137043927	4.89E-02	8.42E-02
PRDX6	0.294331765	7.978344354	8.31E-02	1.32E-01
CAV1	-1.363577928	7.593696333	3.39E-05	1.54E-04
NOS2	0.093792728	2.0369378	9.49E-01	9.65E-01
CBS	2.09780915	0.452457033	4.58E-04	1.52E-03
TXN	0.413708553	8.042888865	7.72E-02	1.24E-01
MDM4	0.160288758	5.265727691	0.366492012	0.456142061
PRKAA1	0.044244156	6.150973556	0.787388847	0.836620817
HSP90AA1	0.329770164	10.39809214	0.03509363	0.063374557
TMEM173	-0.28100113	5.583237378	0.197960676	0.273652853
CARS	0.255101923	6.042863828	0.044667129	0.077821227
PRR4	0.693825849	-0.415713606	0.010484778	0.022594186
