# Supplementary material for: Development of a Ferroptosis-Related lncRNA Signature to Predict the Prognosis and Immune Landscape of Bladder Cancer
Source: Dis Markers. 2021 Jun 20;2021:1031906. doi: 10.1155/2021/1031906 (PMC8238626; doi:10.1155/2021/1031906)
Supplement: Supplementary Materials — Supplementary Figure S1: validation of the risk model with resampling. Note: (a) Kaplan-Meier survival analysis of cases in high-risk group and low-risk group (p < 0.001). (b) ROC curves of the risk score in internal validation dataset. Supplementary Figure S2: Kaplan-Meier subgroup survival analysis in TCGA-BLCA patients. Note: (a) age ≤ 64 (p < 0.001). (b) Age > 64 (p < 0.001). (c) Male (p < 0.001). (d) Female (p < 0.001). (e) Pathological T 1-2 stages (p < 0.001). (f) Pathological T 3-4 stages (p < 0.001). (g) N0 stages (p < 0.001). (h) N1-N3 stages (p < 0.001). (i) M0 stage (p < 0.001). (j) M1 stage (p < 0.05). Supplementary Figure S3: functional prediction of AC090825.1 and MAGI2-AS3. Note: (a) the expression difference of AC090825.1 in BCa samples and paracarcinoma tissue via Wilcoxon signed-rank test. (b) The expression difference of MAGI2-AS3 in BCa samples and paracarcinoma tissue via Wilcoxon signed-rank test. (c) The difference values between the expression of AC090825.1 and MAGI2-AS3 in BCa samples and paracarcinoma samples. (d) The cases suffered poorer survival when the expression value of AC090825.1 was higher than that of MAGI2-AS3 (p < 0.01). (e) The expression levels of AC090825.1 and MAGI2-AS3 were positively correlated both in BCa samples and paracarcinoma samples. (f) Network of lncRNAs, the target genes and their possible transcription factors. (g) UpSet diagram showing 6 overlapped transcription factors. (h) The correlation network of lncRNAs, the target genes and transcription factors. BCa: bladder cancer. Supplementary Table S1: 20 ferroptosis regulators were differentially expressed between BCa and paracarinoma samples. Supplementary Table S2: the Pearson correlation analysis of lncRNAs and ferroptosis regulators. Supplementary Table S3: Kaplan-Meier survival analysis of the lncRNA pairs. Supplementary Table S4: enrichment results from LncSEA website. Supplementary Table S5: GSVA results of different clusters based on ferroptosis regulators [file 1031906.f1.zip › Supplementary_Table_S10.PDF]

Supplementary Table S10. The prediction of the transcription factors of ZEB1.

seqname	source	feature	start	end	score	strand	frame	attributes
ZEB1|NM_001323647	TFBS	TFBS	2300	2305	1	+	.	TF=ZNF354C;class=C2H2 zinc finger factors;sequence=ATCCAC
ZEB1|NM_001323647	TFBS	TFBS	1044	1049	1	-	.	TF=ZNF354C;class=C2H2 zinc finger factors;sequence=ATCCAC
ZEB1|NM_001323647	TFBS	TFBS	276	283	1	-	.	TF=HOXB4;class=Homeo domain factors;sequence=ATCATTAG
ZEB1|NM_001323647	TFBS	TFBS	276	283	1	-	.	TF=HOXD4;class=Homeo domain factors;sequence=ATCATTAG
ZEB1|NM_001323647	TFBS	TFBS	213	221	1	-	.	TF=IRF6;class=Tryptophan cluster factors;sequence=ACCGAAACT
ZEB1|NM_001323647	TFBS	TFBS	616	625	1	-	.	TF=ZKSCAN1;class=C2H2 zinc finger factors;sequence=ATAGTAGGTG
ZEB1|NM_001128128	TFBS	TFBS	2300	2305	1	+	.	TF=ZNF354C;class=C2H2 zinc finger factors;sequence=ATCCAC
ZEB1|NM_001128128	TFBS	TFBS	1477	1483	1	+	.	TF=NFATC2;class=Rel homology region (RHR) factors;sequence=TTTTCCA
ZEB1|NM_001128128	TFBS	TFBS	844	851	1	-	.	TF=PITX1;class=Homeo domain factors;sequence=CTAATCCC
ZEB1|NM_001323641	TFBS	TFBS	2300	2305	1	+	.	TF=ZNF354C;class=C2H2 zinc finger factors;sequence=ATC

								CAC
ZEB1|NM_001323641	TFBS	TFBS	1442	1447	1	-	.	TF=ZNF354C;class=C2H2 zinc finger factors;sequence=ATCCAC
ZEB1|NM_001323641	TFBS	TFBS	75	81	1	-	.	TF=MEIS1;class=Homeo domain factors;sequence=TTGACAG
ZEB1|NM_001323641	TFBS	TFBS	674	681	1	-	.	TF=HOXB4;class=Homeo domain factors;sequence=ATCATTAG
ZEB1|NM_001323641	TFBS	TFBS	674	681	1	-	.	TF=HOXD4;class=Homeo domain factors;sequence=ATCATTAG
ZEB1|NM_001323641	TFBS	TFBS	611	619	1	-	.	TF=IRF6;class=Tryptophan cluster factors;sequence=ACCGAAACT
ZEB1|NM_001323641	TFBS	TFBS	1014	1023	1	-	.	TF=ZKSCAN1;class=C2H2 zinc finger factors;sequence=ATAGTAGGTG
ZEB1|NM_001323638	TFBS	TFBS	2300	2305	1	+	.	TF=ZNF354C;class=C2H2 zinc finger factors;sequence=ATCCAC
ZEB1|NM_001323638	TFBS	TFBS	388	393	1	-	.	TF=ZNF354C;class=C2H2 zinc finger factors;sequence=ATCCAC
ZEB1|NM_001323638	TFBS	TFBS	1843	1848	1	-	.	TF=ZNF354C;class=C2H2 zinc finger factors;sequence=ATCCAC
ZEB1|NM_001323638	TFBS	TFBS	476	482	1	-	.	TF=MEIS1;class=Homeo domain factors;sequence=TTGACAG
ZEB1|NM_001323638	TFBS	TFBS	310	321	1	+	.	TF=ZNF384;class=C2H2 zinc finger factors;sequence=TTTA

								AAAAAAAA
ZEB1|NM_001323638	TFBS	TFBS	85	92	1	+	.	TF=GATA3;class=Other C4 zinc finger-type factors;sequence=AGATAAGA
ZEB1|NM_001323638	TFBS	TFBS	1075	1082	1	-	.	TF=HOXB4;class=Homeo domain factors;sequence=ATCATTAG
ZEB1|NM_001323638	TFBS	TFBS	395	402	1	-	.	TF=HOXC4;class=Homeo domain factors;sequence=GTCATTAA
ZEB1|NM_001323638	TFBS	TFBS	1075	1082	1	-	.	TF=HOXD4;class=Homeo domain factors;sequence=ATCATTAG
ZEB1|NM_001323638	TFBS	TFBS	1012	1020	1	-	.	TF=IRF6;class=Tryptophan cluster factors;sequence=ACCGAAACT
ZEB1|NM_001323638	TFBS	TFBS	1415	1424	1	-	.	TF=ZKSCAN1;class=C2H2 zinc finger factors;sequence=ATAGTAGGTG
ZEB1|NM_001323644	TFBS	TFBS	2300	2305	1	+	.	TF=ZNF354C;class=C2H2 zinc finger factors;sequence=ATCCAC
ZEB1|NM_001323644	TFBS	TFBS	1442	1447	1	-	.	TF=ZNF354C;class=C2H2 zinc finger factors;sequence=ATCCAC
ZEB1|NM_001323644	TFBS	TFBS	75	81	1	-	.	TF=MEIS1;class=Homeo domain factors;sequence=TTGACAG
ZEB1|NM_001323644	TFBS	TFBS	674	681	1	-	.	TF=HOXB4;class=Homeo domain factors;sequence=ATCATTAG
ZEB1|NM_001323644	TFBS	TFBS	674	681	1	-	.	TF=HOXD4;class=Homeo domain factors;sequence=ATC

								ATTAG
ZEB1|NM_001323644	TF BS	TF BS	611	619	1	-	.	TF=IRF6;class=Tryptophan cluster factors;sequence=ACCGAAACT
ZEB1|NM_001323644	TF BS	TF BS	1014	1023	1	-	.	TF=ZKSCAN1;class=C2H2 zinc finger factors;sequence=ATAGTAGGTG
ZEB1|NM_001323643	TF BS	TF BS	2300	2305	1	+	.	TF=ZNF354C;class=C2H2 zinc finger factors;sequence=ATCCAC
ZEB1|NM_001323643	TF BS	TF BS	388	393	1	-	.	TF=ZNF354C;class=C2H2 zinc finger factors;sequence=ATCCAC
ZEB1|NM_001323643	TF BS	TF BS	1843	1848	1	-	.	TF=ZNF354C;class=C2H2 zinc finger factors;sequence=ATCCAC
ZEB1|NM_001323643	TF BS	TF BS	476	482	1	-	.	TF=MEIS1;class=Homeo domain factors;sequence=TTGACAG
ZEB1|NM_001323643	TF BS	TF BS	310	321	1	+	.	TF=ZNF384;class=C2H2 zinc finger factors;sequence=TTTAAAAAAAAA
ZEB1|NM_001323643	TF BS	TF BS	85	92	1	+	.	TF=GATA3;class=Other C4 zinc finger-type factors;sequence=AGATAAGA
ZEB1|NM_001323643	TF BS	TF BS	1075	1082	1	-	.	TF=HOXB4;class=Homeo domain factors;sequence=ATCATTAG
ZEB1|NM_001323643	TF BS	TF BS	395	402	1	-	.	TF=HOXC4;class=Homeo domain factors;sequence=GTCATTAA
ZEB1|NM_001323643	TF BS	TF BS	1075	1082	1	-	.	TF=HOXD4;class=Homeo domain factors;sequence=ATC

								ATTAG
ZEB1|NM_001323643	TFBS	TFBS	1012	1020	1	-	.	TF=IRF6;class=Tryptophan cluster factors;sequence=ACCGAAACT
ZEB1|NM_001323643	TFBS	TFBS	1415	1424	1	-	.	TF=ZKSCAN1;class=C2H2 zinc finger factors;sequence=ATAGTAGGTG
ZEB1|NM_001323674	TFBS	TFBS	2300	2305	1	+	.	TF=ZNF354C;class=C2H2 zinc finger factors;sequence=ATCCAC
ZEB1|NM_001323674	TFBS	TFBS	1044	1049	1	-	.	TF=ZNF354C;class=C2H2 zinc finger factors;sequence=ATCCAC
ZEB1|NM_001323674	TFBS	TFBS	276	283	1	-	.	TF=HOXB4;class=Homeo domain factors;sequence=ATCATTAG
ZEB1|NM_001323674	TFBS	TFBS	276	283	1	-	.	TF=HOXD4;class=Homeo domain factors;sequence=ATCATTAG
ZEB1|NM_001323674	TFBS	TFBS	213	221	1	-	.	TF=IRF6;class=Tryptophan cluster factors;sequence=ACCGAAACT
ZEB1|NM_001323674	TFBS	TFBS	616	625	1	-	.	TF=ZKSCAN1;class=C2H2 zinc finger factors;sequence=ATAGTAGGTG
ZEB1|NM_001323673	TFBS	TFBS	2300	2305	1	+	.	TF=ZNF354C;class=C2H2 zinc finger factors;sequence=ATCCAC
ZEB1|NM_001323673	TFBS	TFBS	1018	1024	1	+	.	TF=NFATC2;class=Rel homology region (RHR) factors;sequence=TTTTCCA
ZEB1|NM_001323673	TFBS	TFBS	385	392	1	-	.	TF=PITX1;class=Homeo domain

								factors;sequence=CTA ATCCC
ZEB1|NM_0 01323658	TF BS	TF BS	23 00	23 05	1	+	.	TF=ZNF354C;class=C2 H2 zinc finger factors;sequence=ATC CAC
ZEB1|NM_0 01323658	TF BS	TF BS	49 2	49 7	1	-	.	TF=ZNF354C;class=C2 H2 zinc finger factors;sequence=ATC CAC
ZEB1|NM_0 01323658	TF BS	TF BS	64	73	1	-	.	TF=ZKSCAN1;class=C 2H2 zinc finger factors;sequence=ATA GTAGGTG
ZEB1|NM_0 01323657	TF BS	TF BS	23 00	23 05	1	+	.	TF=ZNF354C;class=C2 H2 zinc finger factors;sequence=ATC CAC
ZEB1|NM_0 01323657	TF BS	TF BS	49 2	49 7	1	-	.	TF=ZNF354C;class=C2 H2 zinc finger factors;sequence=ATC CAC
ZEB1|NM_0 01323657	TF BS	TF BS	64	73	1	-	.	TF=ZKSCAN1;class=C 2H2 zinc finger factors;sequence=ATA GTAGGTG
ZEB1|NM_0 01323650	TF BS	TF BS	23 00	23 05	1	+	.	TF=ZNF354C;class=C2 H2 zinc finger factors;sequence=ATC CAC
ZEB1|NM_0 01323650	TF BS	TF BS	10 44	10 49	1	-	.	TF=ZNF354C;class=C2 H2 zinc finger factors;sequence=ATC CAC
ZEB1|NM_0 01323650	TF BS	TF BS	27 6	28 3	1	-	.	TF=HOXB4;class=Ho meo domain factors;sequence=ATC ATTAG
ZEB1|NM_0 01323650	TF BS	TF BS	27 6	28 3	1	-	.	TF=HOXD4;class=Ho meo domain factors;sequence=ATC ATTAG
ZEB1|NM_0 01323650	TF BS	TF BS	21 3	22 1	1	-	.	TF=IRF6;class=Tryptop han cluster

								factors;sequence=ACCGAAACT
ZEB1|NM_001323650	TFBS	TFBS	616	625	1	-	.	TF=ZKSCAN1;class=C2H2 zinc finger factors;sequence=ATAGTAGGTG
ZEB1|NM_001323648	TFBS	TFBS	2300	2305	1	+	.	TF=ZNF354C;class=C2H2 zinc finger factors;sequence=ATCCAC
ZEB1|NM_001323648	TFBS	TFBS	1044	1049	1	-	.	TF=ZNF354C;class=C2H2 zinc finger factors;sequence=ATCCAC
ZEB1|NM_001323648	TFBS	TFBS	276	283	1	-	.	TF=HOXB4;class=Homeo domain factors;sequence=ATCATTAG
ZEB1|NM_001323648	TFBS	TFBS	276	283	1	-	.	TF=HOXD4;class=Homeo domain factors;sequence=ATCATTAG
ZEB1|NM_001323648	TFBS	TFBS	213	221	1	-	.	TF=IRF6;class=Tryptophan cluster factors;sequence=ACCGAAACT
ZEB1|NM_001323648	TFBS	TFBS	616	625	1	-	.	TF=ZKSCAN1;class=C2H2 zinc finger factors;sequence=ATAGTAGGTG
ZEB1|NM_001174095	TFBS	TFBS	2300	2305	1	+	.	TF=ZNF354C;class=C2H2 zinc finger factors;sequence=ATCCAC
ZEB1|NM_001174095	TFBS	TFBS	1044	1049	1	-	.	TF=ZNF354C;class=C2H2 zinc finger factors;sequence=ATCCAC
ZEB1|NM_001174095	TFBS	TFBS	276	283	1	-	.	TF=HOXB4;class=Homeo domain factors;sequence=ATCATTAG
ZEB1|NM_001174095	TFBS	TFBS	276	283	1	-	.	TF=HOXD4;class=Homeo domain

								factors;sequence=ATCATTAG
ZEB1|NM_001174095	TFBS	TFBS	213	221	1	-	.	TF=IRF6;class=Tryptophan cluster factors;sequence=ACCGAAACT
ZEB1|NM_001174095	TFBS	TFBS	616	625	1	-	.	TF=ZKSCAN1;class=C2H2 zinc finger factors;sequence=ATAGTAGGTG
ZEB1|NM_001323646	TFBS	TFBS	2300	2305	1	+	.	TF=ZNF354C;class=C2H2 zinc finger factors;sequence=ATCCAC
ZEB1|NM_001323646	TFBS	TFBS	1442	1447	1	-	.	TF=ZNF354C;class=C2H2 zinc finger factors;sequence=ATCCAC
ZEB1|NM_001323646	TFBS	TFBS	75	81	1	-	.	TF=MEIS1;class=Homeo domain factors;sequence=TTGACAG
ZEB1|NM_001323646	TFBS	TFBS	674	681	1	-	.	TF=HOXB4;class=Homeo domain factors;sequence=ATCATTAG
ZEB1|NM_001323646	TFBS	TFBS	674	681	1	-	.	TF=HOXD4;class=Homeo domain factors;sequence=ATCATTAG
ZEB1|NM_001323646	TFBS	TFBS	611	619	1	-	.	TF=IRF6;class=Tryptophan cluster factors;sequence=ACCGAAACT
ZEB1|NM_001323646	TFBS	TFBS	1014	1023	1	-	.	TF=ZKSCAN1;class=C2H2 zinc finger factors;sequence=ATAGTAGGTG
ZEB1|NM_001323645	TFBS	TFBS	2300	2305	1	+	.	TF=ZNF354C;class=C2H2 zinc finger factors;sequence=ATCCAC
ZEB1|NM_001323645	TFBS	TFBS	1442	1447	1	-	.	TF=ZNF354C;class=C2H2 zinc finger

								factors;sequence=ATC CAC
ZEB1|NM_0 01323645	TF BS	TF BS	75	81	1	-	.	TF=MEIS1;class=Hom eo domain factors;sequence=TTG ACAG
ZEB1|NM_0 01323645	TF BS	TF BS	67 4	68 1	1	-	.	TF=HOXB4;class=Ho meo domain factors;sequence=ATC ATTAG
ZEB1|NM_0 01323645	TF BS	TF BS	67 4	68 1	1	-	.	TF=HOXD4;class=Ho meo domain factors;sequence=ATC ATTAG
ZEB1|NM_0 01323645	TF BS	TF BS	61 1	61 9	1	-	.	TF=IRF6;class=Tryptop han cluster factors;sequence=ACC GAAACT
ZEB1|NM_0 01323645	TF BS	TF BS	10 14	10 23	1	-	.	TF=ZKSCAN1;class=C 2H2 zinc finger factors;sequence=ATA GTAGGTG
ZEB1|NM_0 01174093	TF BS	TF BS	23 00	23 05	1	+	.	TF=ZNF354C;class=C2 H2 zinc finger factors;sequence=ATC CAC
ZEB1|NM_0 01174093	TF BS	TF BS	10 44	10 49	1	-	.	TF=ZNF354C;class=C2 H2 zinc finger factors;sequence=ATC CAC
ZEB1|NM_0 01174093	TF BS	TF BS	27 6	28 3	1	-	.	TF=HOXB4;class=Ho meo domain factors;sequence=ATC ATTAG
ZEB1|NM_0 01174093	TF BS	TF BS	27 6	28 3	1	-	.	TF=HOXD4;class=Ho meo domain factors;sequence=ATC ATTAG
ZEB1|NM_0 01174093	TF BS	TF BS	21 3	22 1	1	-	.	TF=IRF6;class=Tryptop han cluster factors;sequence=ACC GAAACT
ZEB1|NM_0 01174093	TF BS	TF BS	61 6	62 5	1	-	.	TF=ZKSCAN1;class=C 2H2 zinc finger

								factors;sequence=ATAGTAGGTG
ZEB1|NM_001323656	TFBS	TFBS	2300	2305	1	+	.	TF=ZNF354C;class=C2H2 zinc finger factors;sequence=ATCCAC
ZEB1|NM_001323656	TFBS	TFBS	492	497	1	-	.	TF=ZNF354C;class=C2H2 zinc finger factors;sequence=ATCCAC
ZEB1|NM_001323656	TFBS	TFBS	64	73	1	-	.	TF=ZKSCAN1;class=C2H2 zinc finger factors;sequence=ATAGTAGGTG
ZEB1|NM_001323655	TFBS	TFBS	2300	2305	1	+	.	TF=ZNF354C;class=C2H2 zinc finger factors;sequence=ATCCAC
ZEB1|NM_001323655	TFBS	TFBS	1044	1049	1	-	.	TF=ZNF354C;class=C2H2 zinc finger factors;sequence=ATCCAC
ZEB1|NM_001323655	TFBS	TFBS	276	283	1	-	.	TF=HOXB4;class=Homeo domain factors;sequence=ATCATTAG
ZEB1|NM_001323655	TFBS	TFBS	276	283	1	-	.	TF=HOXD4;class=Homeo domain factors;sequence=ATCATTAG
ZEB1|NM_001323655	TFBS	TFBS	213	221	1	-	.	TF=IRF6;class=Tryptophan cluster factors;sequence=ACCGAAACT
ZEB1|NM_001323655	TFBS	TFBS	616	625	1	-	.	TF=ZKSCAN1;class=C2H2 zinc finger factors;sequence=ATAGTAGGTG
ZEB1|NM_001323654	TFBS	TFBS	2300	2305	1	+	.	TF=ZNF354C;class=C2H2 zinc finger factors;sequence=ATCCAC
ZEB1|NM_001323654	TFBS	TFBS	1044	1049	1	-	.	TF=ZNF354C;class=C2H2 zinc finger

								factors;sequence=ATCCAC
ZEB1|NM_001323654	TFBS	TFBS	276	283	1	-	.	TF=HOXB4;class=Homeo domain factors;sequence=ATCATTAG
ZEB1|NM_001323654	TFBS	TFBS	276	283	1	-	.	TF=HOXD4;class=Homeo domain factors;sequence=ATCATTAG
ZEB1|NM_001323654	TFBS	TFBS	213	221	1	-	.	TF=IRF6;class=Tryptophan cluster factors;sequence=ACCGAAACT
ZEB1|NM_001323654	TFBS	TFBS	616	625	1	-	.	TF=ZKSCAN1;class=C2H2 zinc finger factors;sequence=ATAGTAGGTG
ZEB1|NM_001323653	TFBS	TFBS	2300	2305	1	+	.	TF=ZNF354C;class=C2H2 zinc finger factors;sequence=ATCCAC
ZEB1|NM_001323653	TFBS	TFBS	1044	1049	1	-	.	TF=ZNF354C;class=C2H2 zinc finger factors;sequence=ATCCAC
ZEB1|NM_001323653	TFBS	TFBS	276	283	1	-	.	TF=HOXB4;class=Homeo domain factors;sequence=ATCATTAG
ZEB1|NM_001323653	TFBS	TFBS	276	283	1	-	.	TF=HOXD4;class=Homeo domain factors;sequence=ATCATTAG
ZEB1|NM_001323653	TFBS	TFBS	213	221	1	-	.	TF=IRF6;class=Tryptophan cluster factors;sequence=ACCGAAACT
ZEB1|NM_001323653	TFBS	TFBS	616	625	1	-	.	TF=ZKSCAN1;class=C2H2 zinc finger factors;sequence=ATAGTAGGTG
ZEB1|NM_001323652	TFBS	TFBS	2300	2305	1	+	.	TF=ZNF354C;class=C2H2 zinc finger

								factors;sequence=ATCCAC
ZEB1|NM_001323652	TFBS	TFBS	1044	1049	1	-	.	TF=ZNF354C;class=C2H2 zinc finger factors;sequence=ATCCAC
ZEB1|NM_001323652	TFBS	TFBS	276	283	1	-	.	TF=HOXB4;class=Homeo domain factors;sequence=ATCATTAG
ZEB1|NM_001323652	TFBS	TFBS	276	283	1	-	.	TF=HOXD4;class=Homeo domain factors;sequence=ATCATTAG
ZEB1|NM_001323652	TFBS	TFBS	213	221	1	-	.	TF=IRF6;class=Tryptophan cluster factors;sequence=ACCGAAACT
ZEB1|NM_001323652	TFBS	TFBS	616	625	1	-	.	TF=ZKSCAN1;class=C2H2 zinc finger factors;sequence=ATAGTAGGTG
ZEB1|NM_001323651	TFBS	TFBS	2300	2305	1	+	.	TF=ZNF354C;class=C2H2 zinc finger factors;sequence=ATCCAC
ZEB1|NM_001323651	TFBS	TFBS	1044	1049	1	-	.	TF=ZNF354C;class=C2H2 zinc finger factors;sequence=ATCCAC
ZEB1|NM_001323651	TFBS	TFBS	276	283	1	-	.	TF=HOXB4;class=Homeo domain factors;sequence=ATCATTAG
ZEB1|NM_001323651	TFBS	TFBS	276	283	1	-	.	TF=HOXD4;class=Homeo domain factors;sequence=ATCATTAG
ZEB1|NM_001323651	TFBS	TFBS	213	221	1	-	.	TF=IRF6;class=Tryptophan cluster factors;sequence=ACCGAAACT
ZEB1|NM_001323651	TFBS	TFBS	616	625	1	-	.	TF=ZKSCAN1;class=C2H2 zinc finger

								factors;sequence=ATA GTAGGTG
ZEB1|NM_0 01323678	TF BS	TF BS	23 00	23 05	1	+	.	TF=ZNF354C;class=C2 H2 zinc finger factors;sequence=ATC CAC
ZEB1|NM_0 01323678	TF BS	TF BS	14 77	14 83	1	+	.	TF=NFATC2;class=Rel homology region (RHR) factors;sequence=TTTT CCA
ZEB1|NM_0 01323678	TF BS	TF BS	84 4	85 1	1	-	.	TF=PITX1;class=Home o domain factors;sequence=CTA ATCCC
ZEB1|NM_0 01323677	TF BS	TF BS	23 00	23 05	1	+	.	TF=ZNF354C;class=C2 H2 zinc finger factors;sequence=ATC CAC
ZEB1|NM_0 01323677	TF BS	TF BS	10 18	10 24	1	+	.	TF=NFATC2;class=Rel homology region (RHR) factors;sequence=TTTT CCA
ZEB1|NM_0 01323677	TF BS	TF BS	38 5	39 2	1	-	.	TF=PITX1;class=Home o domain factors;sequence=CTA ATCCC
ZEB1|NM_0 01323676	TF BS	TF BS	23 00	23 05	1	+	.	TF=ZNF354C;class=C2 H2 zinc finger factors;sequence=ATC CAC
ZEB1|NM_0 01323676	TF BS	TF BS	10 18	10 24	1	+	.	TF=NFATC2;class=Rel homology region (RHR) factors;sequence=TTTT CCA
ZEB1|NM_0 01323676	TF BS	TF BS	38 5	39 2	1	-	.	TF=PITX1;class=Home o domain factors;sequence=CTA ATCCC
ZEB1|NM_0 01323649	TF BS	TF BS	23 00	23 05	1	+	.	TF=ZNF354C;class=C2 H2 zinc finger factors;sequence=ATC

								CAC
ZEB1|NM_0 01323649	TF BS	TF BS	10 44	10 49	1	-	.	TF=ZNF354C;class=C2 H2 zinc finger factors;sequence=ATC CAC
ZEB1|NM_0 01323649	TF BS	TF BS	27 6	28 3	1	-	.	TF=HOXB4;class=Ho meo domain factors;sequence=ATC ATTAG
ZEB1|NM_0 01323649	TF BS	TF BS	27 6	28 3	1	-	.	TF=HOXD4;class=Ho meo domain factors;sequence=ATC ATTAG
ZEB1|NM_0 01323649	TF BS	TF BS	21 3	22 1	1	-	.	TF=IRF6;class=Tryptop han cluster factors;sequence=ACC GAAACT
ZEB1|NM_0 01323649	TF BS	TF BS	61 6	62 5	1	-	.	TF=ZKSCAN1;class=C 2H2 zinc finger factors;sequence=ATA GTAGGTG
ZEB1|NM_0 01323675	TF BS	TF BS	23 00	23 05	1	+	.	TF=ZNF354C;class=C2 H2 zinc finger factors;sequence=ATC CAC
ZEB1|NM_0 01323675	TF BS	TF BS	10 18	10 24	1	+	.	TF=NFATC2;class=Rel homology region (RHR) factors;sequence=TTTT CCA
ZEB1|NM_0 01323675	TF BS	TF BS	38 5	39 2	1	-	.	TF=PITX1;class=Home o domain factors;sequence=CTA ATCCC
ZEB1|NM_0 01323672	TF BS	TF BS	23 00	23 05	1	+	.	TF=ZNF354C;class=C2 H2 zinc finger factors;sequence=ATC CAC
ZEB1|NM_0 01323672	TF BS	TF BS	10 18	10 24	1	+	.	TF=NFATC2;class=Rel homology region (RHR) factors;sequence=TTTT CCA
ZEB1|NM_0	TF	TF	38	39	1	-	.	TF=PITX1;class=Home

01323672	BS	BS	5	2				o domain factors;sequence=CTAATCCC
ZEB1|NM_001323671	TFBS	TFBS	2300	2305	1	+	.	TF=ZNF354C;class=C2H2 zinc finger factors;sequence=ATCCAC
ZEB1|NM_001323671	TFBS	TFBS	1018	1024	1	+	.	TF=NFATC2;class=Rel homology region (RHR) factors;sequence=TTTTCCA
ZEB1|NM_001323671	TFBS	TFBS	385	392	1	-	.	TF=PITX1;class=Homeo domain factors;sequence=CTAATCCC
ZEB1|NM_001323666	TFBS	TFBS	2300	2305	1	+	.	TF=ZNF354C;class=C2H2 zinc finger factors;sequence=ATCCAC
ZEB1|NM_001323666	TFBS	TFBS	1477	1483	1	+	.	TF=NFATC2;class=Rel homology region (RHR) factors;sequence=TTTTCCA
ZEB1|NM_001323666	TFBS	TFBS	844	851	1	-	.	TF=PITX1;class=Homeo domain factors;sequence=CTAATCCC
ZEB1|NM_001323642	TFBS	TFBS	2300	2305	1	+	.	TF=ZNF354C;class=C2H2 zinc finger factors;sequence=ATCCAC
ZEB1|NM_001323642	TFBS	TFBS	1442	1447	1	-	.	TF=ZNF354C;class=C2H2 zinc finger factors;sequence=ATCCAC
ZEB1|NM_001323642	TFBS	TFBS	75	81	1	-	.	TF=MEIS1;class=Homeo domain factors;sequence=TTGACAG
ZEB1|NM_001323642	TFBS	TFBS	674	681	1	-	.	TF=HOXB4;class=Homeo domain factors;sequence=ATC

								ATTAG
ZEB1|NM_001323642	TFBS	TFBS	674	681	1	-	.	TF=HOXD4;class=Homeo domain factors;sequence=ATCATTAG
ZEB1|NM_001323642	TFBS	TFBS	611	619	1	-	.	TF=IRF6;class=Tryptophan cluster factors;sequence=ACCGAAACT
ZEB1|NM_001323642	TFBS	TFBS	1014	1023	1	-	.	TF=ZKSCAN1;class=C2H2 zinc finger factors;sequence=ATAGTAGGTG
ZEB1|NM_001174096	TFBS	TFBS	2300	2305	1	+	.	TF=ZNF354C;class=C2H2 zinc finger factors;sequence=ATCCAC
ZEB1|NM_001174096	TFBS	TFBS	1044	1049	1	-	.	TF=ZNF354C;class=C2H2 zinc finger factors;sequence=ATCCAC
ZEB1|NM_001174096	TFBS	TFBS	276	283	1	-	.	TF=HOXB4;class=Homeo domain factors;sequence=ATCATTAG
ZEB1|NM_001174096	TFBS	TFBS	276	283	1	-	.	TF=HOXD4;class=Homeo domain factors;sequence=ATCATTAG
ZEB1|NM_001174096	TFBS	TFBS	213	221	1	-	.	TF=IRF6;class=Tryptophan cluster factors;sequence=ACCGAAACT
ZEB1|NM_001174096	TFBS	TFBS	616	625	1	-	.	TF=ZKSCAN1;class=C2H2 zinc finger factors;sequence=ATAGTAGGTG
ZEB1|NM_030751	TFBS	TFBS	2300	2305	1	+	.	TF=ZNF354C;class=C2H2 zinc finger factors;sequence=ATCCAC
ZEB1|NM_030751	TFBS	TFBS	1044	1049	1	-	.	TF=ZNF354C;class=C2H2 zinc finger factors;sequence=ATC

								CAC
ZEB1|NM_030751	TFBS	TFBS	276	283	1	-	.	TF=HOXB4;class=Homeo domain factors;sequence=ATCATTAG
ZEB1|NM_030751	TFBS	TFBS	276	283	1	-	.	TF=HOXD4;class=Homeo domain factors;sequence=ATCATTAG
ZEB1|NM_030751	TFBS	TFBS	213	221	1	-	.	TF=IRF6;class=Tryptophan cluster factors;sequence=ACCGAAACT
ZEB1|NM_030751	TFBS	TFBS	616	625	1	-	.	TF=ZKSCAN1;class=C2H2 zinc finger factors;sequence=ATAGTAGGTG
ZEB1|NM_001323665	TFBS	TFBS	2300	2305	1	+	.	TF=ZNF354C;class=C2H2 zinc finger factors;sequence=ATCCAC
ZEB1|NM_001323665	TFBS	TFBS	1477	1483	1	+	.	TF=NFATC2;class=Rel homology region (RHR) factors;sequence=TTTTCCA
ZEB1|NM_001323665	TFBS	TFBS	844	851	1	-	.	TF=PITX1;class=Homeo domain factors;sequence=CTAATCCC
ZEB1|NM_001323664	TFBS	TFBS	2300	2305	1	+	.	TF=ZNF354C;class=C2H2 zinc finger factors;sequence=ATCCAC
ZEB1|NM_001323664	TFBS	TFBS	1477	1483	1	+	.	TF=NFATC2;class=Rel homology region (RHR) factors;sequence=TTTTCCA
ZEB1|NM_001323664	TFBS	TFBS	844	851	1	-	.	TF=PITX1;class=Homeo domain factors;sequence=CTAATCCC
ZEB1|NM_0	TF	TF	23	23	1	+	.	TF=ZNF354C;class=C2

01323663	BS	BS	00	05				H2 zinc finger factors;sequence=ATC CAC
ZEB1|NM_0 01323663	TF BS	TF BS	14 77	14 83	1	+	.	TF=NFATC2;class=Rel homology region (RHR) factors;sequence=TTTT CCA
ZEB1|NM_0 01323663	TF BS	TF BS	84 4	85 1	1	-	.	TF=PITX1;class=Home o domain factors;sequence=CTA ATCCC
ZEB1|NM_0 01323662	TF BS	TF BS	23 00	23 05	1	+	.	TF=ZNF354C;class=C2 H2 zinc finger factors;sequence=ATC CAC
ZEB1|NM_0 01323662	TF BS	TF BS	14 77	14 83	1	+	.	TF=NFATC2;class=Rel homology region (RHR) factors;sequence=TTTT CCA
ZEB1|NM_0 01323662	TF BS	TF BS	84 4	85 1	1	-	.	TF=PITX1;class=Home o domain factors;sequence=CTA ATCCC
ZEB1|NM_0 01323661	TF BS	TF BS	23 00	23 05	1	+	.	TF=ZNF354C;class=C2 H2 zinc finger factors;sequence=ATC CAC
ZEB1|NM_0 01323661	TF BS	TF BS	19 97	20 07	1	-	.	TF=CDX4;class=Home o domain factors;sequence=GGC AATAAAAC
ZEB1|NM_0 01323661	TF BS	TF BS	19 97	20 06	1	-	.	TF=HOXD9;class=Ho meo domain factors;sequence=GCA ATAAAAC
ZEB1|NM_0 01323661	TF BS	TF BS	14 91	14 98	1	-	.	TF=PITX1;class=Home o domain factors;sequence=CTA ATCCC
ZEB1|NM_0 01323660	TF BS	TF BS	23 00	23 05	1	+	.	TF=ZNF354C;class=C2 H2 zinc finger factors;sequence=ATC

								CAC
ZEB1|NM_001323660	TFBS	TFBS	1997	2007	1	-	.	TF=CDX4;class=Homeo domain factors;sequence=GGCAATAAAAC
ZEB1|NM_001323660	TFBS	TFBS	1997	2006	1	-	.	TF=HOXD9;class=Homeo domain factors;sequence=GCAATAAAAC
ZEB1|NM_001323660	TFBS	TFBS	1491	1498	1	-	.	TF=PITX1;class=Homeo domain factors;sequence=CTAATCCC
ZEB1|NM_001323659	TFBS	TFBS	2300	2305	1	+	.	TF=ZNF354C;class=C2H2 zinc finger factors;sequence=ATCCAC
ZEB1|NM_001323659	TFBS	TFBS	1997	2007	1	-	.	TF=CDX4;class=Homeo domain factors;sequence=GGCAATAAAAC
ZEB1|NM_001323659	TFBS	TFBS	1997	2006	1	-	.	TF=HOXD9;class=Homeo domain factors;sequence=GCAATAAAAC
ZEB1|NM_001323659	TFBS	TFBS	1491	1498	1	-	.	TF=PITX1;class=Homeo domain factors;sequence=CTAATCCC
ZEB1|NM_001174094	TFBS	TFBS	2300	2305	1	+	.	TF=ZNF354C;class=C2H2 zinc finger factors;sequence=ATCCAC
ZEB1|NM_001174094	TFBS	TFBS	1477	1483	1	+	.	TF=NFATC2;class=Rel homology region (RHR) factors;sequence=TTTTCCA
ZEB1|NM_001174094	TFBS	TFBS	844	851	1	-	.	TF=PITX1;class=Homeo domain factors;sequence=CTAATCCC
ZEB1|NM_001128128	TFBS	TFBS	1909	1918	0.999742203	+	.	TF=KLF5;class=C2H2 zinc finger

								factors;sequence=GCCCCACCCC
ZEB1|NM_001323673	TFBS	TFBS	1450	1459	0.999742203	+	.	TF=KLF5;class=C2H2 zinc finger factors;sequence=GCCCCACCCC
ZEB1|NM_001323678	TFBS	TFBS	1909	1918	0.999742203	+	.	TF=KLF5;class=C2H2 zinc finger factors;sequence=GCCCCACCCC
ZEB1|NM_001323677	TFBS	TFBS	1450	1459	0.999742203	+	.	TF=KLF5;class=C2H2 zinc finger factors;sequence=GCCCCACCCC
ZEB1|NM_001323676	TFBS	TFBS	1450	1459	0.999742203	+	.	TF=KLF5;class=C2H2 zinc finger factors;sequence=GCCCCACCCC
ZEB1|NM_001323675	TFBS	TFBS	1450	1459	0.999742203	+	.	TF=KLF5;class=C2H2 zinc finger factors;sequence=GCCCCACCCC
ZEB1|NM_001323672	TFBS	TFBS	1450	1459	0.999742203	+	.	TF=KLF5;class=C2H2 zinc finger factors;sequence=GCCCCACCCC
ZEB1|NM_001323671	TFBS	TFBS	1450	1459	0.999742203	+	.	TF=KLF5;class=C2H2 zinc finger factors;sequence=GCCCCACCCC
ZEB1|NM_001323666	TFBS	TFBS	1909	1918	0.999742203	+	.	TF=KLF5;class=C2H2 zinc finger factors;sequence=GCCCCACCCC
ZEB1|NM_001323665	TFBS	TFBS	1909	1918	0.999742203	+	.	TF=KLF5;class=C2H2 zinc finger factors;sequence=GCCCCACCCC
ZEB1|NM_001323664	TFBS	TFBS	1909	1918	0.999742203	+	.	TF=KLF5;class=C2H2 zinc finger factors;sequence=GCCCCACCCC
ZEB1|NM_001323663	TFBS	TFBS	1909	1918	0.999742203	+	.	TF=KLF5;class=C2H2 zinc finger

								factors;sequence=GCCCCACCCC
ZEB1|NM_001323662	TFBS	TFBS	1909	1918	0.999742203	+	.	TF=KLF5;class=C2H2 zinc finger factors;sequence=GCCCCACCCC
ZEB1|NM_001174094	TFBS	TFBS	1909	1918	0.999742203	+	.	TF=KLF5;class=C2H2 zinc finger factors;sequence=GCCCCACCCC
ZEB1|NM_001323647	TFBS	TFBS	1926	1936	0.999738076	-	.	TF=MAZ;class=C2H2 zinc finger factors;sequence=CCCCCCTCCCC
ZEB1|NM_001323674	TFBS	TFBS	1926	1936	0.999738076	-	.	TF=MAZ;class=C2H2 zinc finger factors;sequence=CCCCCCTCCCC
ZEB1|NM_001323658	TFBS	TFBS	1374	1384	0.999738076	-	.	TF=MAZ;class=C2H2 zinc finger factors;sequence=CCCCCCTCCCC
ZEB1|NM_001323657	TFBS	TFBS	1374	1384	0.999738076	-	.	TF=MAZ;class=C2H2 zinc finger factors;sequence=CCCCCCTCCCC
ZEB1|NM_001323650	TFBS	TFBS	1926	1936	0.999738076	-	.	TF=MAZ;class=C2H2 zinc finger factors;sequence=CCCCCCTCCCC
ZEB1|NM_001323648	TFBS	TFBS	1926	1936	0.999738076	-	.	TF=MAZ;class=C2H2 zinc finger factors;sequence=CCCCCCTCCCC
ZEB1|NM_001174095	TFBS	TFBS	1926	1936	0.999738076	-	.	TF=MAZ;class=C2H2 zinc finger factors;sequence=CCCCCCTCCCC
ZEB1|NM_001174093	TFBS	TFBS	1926	1936	0.999738076	-	.	TF=MAZ;class=C2H2 zinc finger factors;sequence=CCCCCCTCCCC
ZEB1|NM_001323656	TFBS	TFBS	1374	1384	0.999738076	-	.	TF=MAZ;class=C2H2 zinc finger

								factors;sequence=CCCCCCTCCCC
ZEB1|NM_001323655	TF BS	TF BS	1926	1936	0.999738076	-	.	TF=MAZ;class=C2H2 zinc finger factors;sequence=CCCCCCTCCCC
ZEB1|NM_001323654	TF BS	TF BS	1926	1936	0.999738076	-	.	TF=MAZ;class=C2H2 zinc finger factors;sequence=CCCCCCTCCCC
ZEB1|NM_001323653	TF BS	TF BS	1926	1936	0.999738076	-	.	TF=MAZ;class=C2H2 zinc finger factors;sequence=CCCCCCTCCCC
ZEB1|NM_001323652	TF BS	TF BS	1926	1936	0.999738076	-	.	TF=MAZ;class=C2H2 zinc finger factors;sequence=CCCCCCTCCCC
ZEB1|NM_001323651	TF BS	TF BS	1926	1936	0.999738076	-	.	TF=MAZ;class=C2H2 zinc finger factors;sequence=CCCCCCTCCCC
ZEB1|NM_001323649	TF BS	TF BS	1926	1936	0.999738076	-	.	TF=MAZ;class=C2H2 zinc finger factors;sequence=CCCCCCTCCCC
ZEB1|NM_001174096	TF BS	TF BS	1926	1936	0.999738076	-	.	TF=MAZ;class=C2H2 zinc finger factors;sequence=CCCCCCTCCCC
ZEB1|NM_030751	TF BS	TF BS	1926	1936	0.999738076	-	.	TF=MAZ;class=C2H2 zinc finger factors;sequence=CCCCCCTCCCC
ZEB1|NM_001323661	TF BS	TF BS	588	598	0.999738076	-	.	TF=MAZ;class=C2H2 zinc finger factors;sequence=CCCCCCTCCCC
ZEB1|NM_001323660	TF BS	TF BS	588	598	0.999738076	-	.	TF=MAZ;class=C2H2 zinc finger factors;sequence=CCCCCCTCCCC
ZEB1|NM_001323659	TF BS	TF BS	588	598	0.999738076	-	.	TF=MAZ;class=C2H2 zinc finger

								factors;sequence=CCCCCCTCCCC
ZEB1|NM_001323647	TF BS	TF BS	266	273	0.999565985	+	.	TF=HOXC4;class=Homeo domain factors;sequence=ATCATTAA
ZEB1|NM_001323641	TF BS	TF BS	664	671	0.999565985	+	.	TF=HOXC4;class=Homeo domain factors;sequence=ATCATTAA
ZEB1|NM_001323638	TF BS	TF BS	1065	1072	0.999565985	+	.	TF=HOXC4;class=Homeo domain factors;sequence=ATCATTAA
ZEB1|NM_001323644	TF BS	TF BS	664	671	0.999565985	+	.	TF=HOXC4;class=Homeo domain factors;sequence=ATCATTAA
ZEB1|NM_001323643	TF BS	TF BS	1065	1072	0.999565985	+	.	TF=HOXC4;class=Homeo domain factors;sequence=ATCATTAA
ZEB1|NM_001323674	TF BS	TF BS	266	273	0.999565985	+	.	TF=HOXC4;class=Homeo domain factors;sequence=ATCATTAA
ZEB1|NM_001323673	TF BS	TF BS	1640	1647	0.999565985	+	.	TF=HOXC4;class=Homeo domain factors;sequence=ATCATTAA
ZEB1|NM_001323650	TF BS	TF BS	266	273	0.999565985	+	.	TF=HOXC4;class=Homeo domain factors;sequence=ATCATTAA
ZEB1|NM_001323648	TF BS	TF BS	266	273	0.999565985	+	.	TF=HOXC4;class=Homeo domain factors;sequence=ATCATTAA
ZEB1|NM_001174095	TF BS	TF BS	266	273	0.999565985	+	.	TF=HOXC4;class=Homeo domain factors;sequence=ATCATTAA
ZEB1|NM_001323646	TF BS	TF BS	664	671	0.999565985	+	.	TF=HOXC4;class=Homeo domain

								factors;sequence=ATC ATTAA
ZEB1|NM_0 01323645	TF BS	TF BS	66 4	67 1	0.9995 65985	+	.	TF=HOXC4;class=Ho meo domain factors;sequence=ATC ATTAA
ZEB1|NM_0 01174093	TF BS	TF BS	26 6	27 3	0.9995 65985	+	.	TF=HOXC4;class=Ho meo domain factors;sequence=ATC ATTAA
ZEB1|NM_0 01323655	TF BS	TF BS	26 6	27 3	0.9995 65985	+	.	TF=HOXC4;class=Ho meo domain factors;sequence=ATC ATTAA
ZEB1|NM_0 01323654	TF BS	TF BS	26 6	27 3	0.9995 65985	+	.	TF=HOXC4;class=Ho meo domain factors;sequence=ATC ATTAA
ZEB1|NM_0 01323653	TF BS	TF BS	26 6	27 3	0.9995 65985	+	.	TF=HOXC4;class=Ho meo domain factors;sequence=ATC ATTAA
ZEB1|NM_0 01323652	TF BS	TF BS	26 6	27 3	0.9995 65985	+	.	TF=HOXC4;class=Ho meo domain factors;sequence=ATC ATTAA
ZEB1|NM_0 01323651	TF BS	TF BS	26 6	27 3	0.9995 65985	+	.	TF=HOXC4;class=Ho meo domain factors;sequence=ATC ATTAA
ZEB1|NM_0 01323677	TF BS	TF BS	16 40	16 47	0.9995 65985	+	.	TF=HOXC4;class=Ho meo domain factors;sequence=ATC ATTAA
ZEB1|NM_0 01323676	TF BS	TF BS	16 40	16 47	0.9995 65985	+	.	TF=HOXC4;class=Ho meo domain factors;sequence=ATC ATTAA
ZEB1|NM_0 01323649	TF BS	TF BS	26 6	27 3	0.9995 65985	+	.	TF=HOXC4;class=Ho meo domain factors;sequence=ATC ATTAA
ZEB1|NM_0 01323675	TF BS	TF BS	16 40	16 47	0.9995 65985	+	.	TF=HOXC4;class=Ho meo domain

								factors;sequence=ATC ATTAA
ZEB1|NM_0 01323672	TF BS	TF BS	16 40	16 47	0.9995 65985	+	.	TF=HOXC4;class=Ho meo domain factors;sequence=ATC ATTAA
ZEB1|NM_0 01323671	TF BS	TF BS	16 40	16 47	0.9995 65985	+	.	TF=HOXC4;class=Ho meo domain factors;sequence=ATC ATTAA
ZEB1|NM_0 01323642	TF BS	TF BS	66 4	67 1	0.9995 65985	+	.	TF=HOXC4;class=Ho meo domain factors;sequence=ATC ATTAA
ZEB1|NM_0 01174096	TF BS	TF BS	26 6	27 3	0.9995 65985	+	.	TF=HOXC4;class=Ho meo domain factors;sequence=ATC ATTAA
ZEB1|NM_0 30751	TF BS	TF BS	26 6	27 3	0.9995 65985	+	.	TF=HOXC4;class=Ho meo domain factors;sequence=ATC ATTAA
ZEB1|NM_0 01323641	TF BS	TF BS	96	10 3	0.9994 17997	+	.	TF=NR2C2(var.2);class =Nuclear receptors with C4 zinc fingers;sequence=AAG GTCAA
ZEB1|NM_0 01323638	TF BS	TF BS	49 7	50 4	0.9994 17997	+	.	TF=NR2C2(var.2);class =Nuclear receptors with C4 zinc fingers;sequence=AAG GTCAA
ZEB1|NM_0 01323644	TF BS	TF BS	96	10 3	0.9994 17997	+	.	TF=NR2C2(var.2);class =Nuclear receptors with C4 zinc fingers;sequence=AAG GTCAA
ZEB1|NM_0 01323643	TF BS	TF BS	49 7	50 4	0.9994 17997	+	.	TF=NR2C2(var.2);class =Nuclear receptors with C4 zinc fingers;sequence=AAG GTCAA
ZEB1|NM_0 01323646	TF BS	TF BS	96	10 3	0.9994 17997	+	.	TF=NR2C2(var.2);class =Nuclear receptors with

								C4 zinc fingers;sequence=AAGGTCAA
ZEB1|NM_001323645	TFBS	TFBS	96	103	0.999417997	+	.	TF=NR2C2(var.2);class=Nuclear receptors with C4 zinc fingers;sequence=AAGGTCAA
ZEB1|NM_001323642	TFBS	TFBS	96	103	0.999417997	+	.	TF=NR2C2(var.2);class=Nuclear receptors with C4 zinc fingers;sequence=AAGGTCAA
ZEB1|NM_001323647	TFBS	TFBS	266	273	0.999331142	+	.	TF=HOXD4;class=Homeo domain factors;sequence=ATCATTAA
ZEB1|NM_001323641	TFBS	TFBS	664	671	0.999331142	+	.	TF=HOXD4;class=Homeo domain factors;sequence=ATCATTAA
ZEB1|NM_001323638	TFBS	TFBS	1065	1072	0.999331142	+	.	TF=HOXD4;class=Homeo domain factors;sequence=ATCATTAA
ZEB1|NM_001323644	TFBS	TFBS	664	671	0.999331142	+	.	TF=HOXD4;class=Homeo domain factors;sequence=ATCATTAA
ZEB1|NM_001323643	TFBS	TFBS	1065	1072	0.999331142	+	.	TF=HOXD4;class=Homeo domain factors;sequence=ATCATTAA
ZEB1|NM_001323674	TFBS	TFBS	266	273	0.999331142	+	.	TF=HOXD4;class=Homeo domain factors;sequence=ATCATTAA
ZEB1|NM_001323673	TFBS	TFBS	1640	1647	0.999331142	+	.	TF=HOXD4;class=Homeo domain factors;sequence=ATCATTAA
ZEB1|NM_001323650	TFBS	TFBS	266	273	0.999331142	+	.	TF=HOXD4;class=Homeo domain factors;sequence=ATC

								ATTAA
ZEB1|NM_001323648	TFBS	TFBS	266	273	0.999331142	+	.	TF=HOXD4;class=Homeo domain factors;sequence=ATCATTAA
ZEB1|NM_001174095	TFBS	TFBS	266	273	0.999331142	+	.	TF=HOXD4;class=Homeo domain factors;sequence=ATCATTAA
ZEB1|NM_001323646	TFBS	TFBS	664	671	0.999331142	+	.	TF=HOXD4;class=Homeo domain factors;sequence=ATCATTAA
ZEB1|NM_001323645	TFBS	TFBS	664	671	0.999331142	+	.	TF=HOXD4;class=Homeo domain factors;sequence=ATCATTAA
ZEB1|NM_001174093	TFBS	TFBS	266	273	0.999331142	+	.	TF=HOXD4;class=Homeo domain factors;sequence=ATCATTAA
ZEB1|NM_001323655	TFBS	TFBS	266	273	0.999331142	+	.	TF=HOXD4;class=Homeo domain factors;sequence=ATCATTAA
ZEB1|NM_001323654	TFBS	TFBS	266	273	0.999331142	+	.	TF=HOXD4;class=Homeo domain factors;sequence=ATCATTAA
ZEB1|NM_001323653	TFBS	TFBS	266	273	0.999331142	+	.	TF=HOXD4;class=Homeo domain factors;sequence=ATCATTAA
ZEB1|NM_001323652	TFBS	TFBS	266	273	0.999331142	+	.	TF=HOXD4;class=Homeo domain factors;sequence=ATCATTAA
ZEB1|NM_001323651	TFBS	TFBS	266	273	0.999331142	+	.	TF=HOXD4;class=Homeo domain factors;sequence=ATCATTAA
ZEB1|NM_001323677	TFBS	TFBS	1640	1647	0.999331142	+	.	TF=HOXD4;class=Homeo domain factors;sequence=ATC

								ATTAA
ZEB1|NM_001323676	TFBS	TFBS	1640	1647	0.999331142	+	.	TF=HOXD4;class=Homeo domain factors;sequence=ATCATTAA
ZEB1|NM_001323649	TFBS	TFBS	266	273	0.999331142	+	.	TF=HOXD4;class=Homeo domain factors;sequence=ATCATTAA
ZEB1|NM_001323675	TFBS	TFBS	1640	1647	0.999331142	+	.	TF=HOXD4;class=Homeo domain factors;sequence=ATCATTAA
ZEB1|NM_001323672	TFBS	TFBS	1640	1647	0.999331142	+	.	TF=HOXD4;class=Homeo domain factors;sequence=ATCATTAA
ZEB1|NM_001323671	TFBS	TFBS	1640	1647	0.999331142	+	.	TF=HOXD4;class=Homeo domain factors;sequence=ATCATTAA
ZEB1|NM_001323642	TFBS	TFBS	664	671	0.999331142	+	.	TF=HOXD4;class=Homeo domain factors;sequence=ATCATTAA
ZEB1|NM_001174096	TFBS	TFBS	266	273	0.999331142	+	.	TF=HOXD4;class=Homeo domain factors;sequence=ATCATTAA
ZEB1|NM_030751	TFBS	TFBS	266	273	0.999331142	+	.	TF=HOXD4;class=Homeo domain factors;sequence=ATCATTAA
ZEB1|NM_001323647	TFBS	TFBS	276	283	0.999212186	-	.	TF=HOXC4;class=Homeo domain factors;sequence=ATCATTAG
ZEB1|NM_001323641	TFBS	TFBS	674	681	0.999212186	-	.	TF=HOXC4;class=Homeo domain factors;sequence=ATCATTAG
ZEB1|NM_001323638	TFBS	TFBS	1075	1082	0.999212186	-	.	TF=HOXC4;class=Homeo domain factors;sequence=ATC

								ATTAG
ZEB1|NM_001323644	TFBS	TFBS	674	681	0.999212186	-	.	TF=HOXC4;class=Homeo domain factors;sequence=ATCATTAG
ZEB1|NM_001323643	TFBS	TFBS	1075	1082	0.999212186	-	.	TF=HOXC4;class=Homeo domain factors;sequence=ATCATTAG
ZEB1|NM_001323674	TFBS	TFBS	276	283	0.999212186	-	.	TF=HOXC4;class=Homeo domain factors;sequence=ATCATTAG
ZEB1|NM_001323650	TFBS	TFBS	276	283	0.999212186	-	.	TF=HOXC4;class=Homeo domain factors;sequence=ATCATTAG
ZEB1|NM_001323648	TFBS	TFBS	276	283	0.999212186	-	.	TF=HOXC4;class=Homeo domain factors;sequence=ATCATTAG
ZEB1|NM_001174095	TFBS	TFBS	276	283	0.999212186	-	.	TF=HOXC4;class=Homeo domain factors;sequence=ATCATTAG
ZEB1|NM_001323646	TFBS	TFBS	674	681	0.999212186	-	.	TF=HOXC4;class=Homeo domain factors;sequence=ATCATTAG
ZEB1|NM_001323645	TFBS	TFBS	674	681	0.999212186	-	.	TF=HOXC4;class=Homeo domain factors;sequence=ATCATTAG
ZEB1|NM_001174093	TFBS	TFBS	276	283	0.999212186	-	.	TF=HOXC4;class=Homeo domain factors;sequence=ATCATTAG
ZEB1|NM_001323655	TFBS	TFBS	276	283	0.999212186	-	.	TF=HOXC4;class=Homeo domain factors;sequence=ATCATTAG
ZEB1|NM_001323654	TFBS	TFBS	276	283	0.999212186	-	.	TF=HOXC4;class=Homeo domain factors;sequence=ATC

								ATTAG
ZEB1|NM_001323653	TFBS	TFBS	276	283	0.999212186	-	.	TF=HOXC4;class=Homeo domain factors;sequence=ATCATTAG
ZEB1|NM_001323652	TFBS	TFBS	276	283	0.999212186	-	.	TF=HOXC4;class=Homeo domain factors;sequence=ATCATTAG
ZEB1|NM_001323651	TFBS	TFBS	276	283	0.999212186	-	.	TF=HOXC4;class=Homeo domain factors;sequence=ATCATTAG
ZEB1|NM_001323649	TFBS	TFBS	276	283	0.999212186	-	.	TF=HOXC4;class=Homeo domain factors;sequence=ATCATTAG
ZEB1|NM_001323642	TFBS	TFBS	674	681	0.999212186	-	.	TF=HOXC4;class=Homeo domain factors;sequence=ATCATTAG
ZEB1|NM_001174096	TFBS	TFBS	276	283	0.999212186	-	.	TF=HOXC4;class=Homeo domain factors;sequence=ATCATTAG
ZEB1|NM_030751	TFBS	TFBS	276	283	0.999212186	-	.	TF=HOXC4;class=Homeo domain factors;sequence=ATCATTAG
ZEB1|NM_001323638	TFBS	TFBS	398	404	0.999127731	+	.	TF=MEIS1;class=Homeo domain factors;sequence=ATGACAG
ZEB1|NM_001323643	TFBS	TFBS	398	404	0.999127731	+	.	TF=MEIS1;class=Homeo domain factors;sequence=ATGACAG
ZEB1|NM_001323647	TFBS	TFBS	266	273	0.999053421	+	.	TF=HOXB4;class=Homeo domain factors;sequence=ATCATTAA
ZEB1|NM_001323641	TFBS	TFBS	664	671	0.999053421	+	.	TF=HOXB4;class=Homeo domain factors;sequence=ATC

								ATTAA
ZEB1|NM_001323638	TFBS	TFBS	1065	1072	0.999053421	+	.	TF=HOXB4;class=Homeo domain factors;sequence=ATCATTAA
ZEB1|NM_001323644	TFBS	TFBS	664	671	0.999053421	+	.	TF=HOXB4;class=Homeo domain factors;sequence=ATCATTAA
ZEB1|NM_001323643	TFBS	TFBS	1065	1072	0.999053421	+	.	TF=HOXB4;class=Homeo domain factors;sequence=ATCATTAA
ZEB1|NM_001323674	TFBS	TFBS	266	273	0.999053421	+	.	TF=HOXB4;class=Homeo domain factors;sequence=ATCATTAA
ZEB1|NM_001323673	TFBS	TFBS	1640	1647	0.999053421	+	.	TF=HOXB4;class=Homeo domain factors;sequence=ATCATTAA
ZEB1|NM_001323650	TFBS	TFBS	266	273	0.999053421	+	.	TF=HOXB4;class=Homeo domain factors;sequence=ATCATTAA
ZEB1|NM_001323648	TFBS	TFBS	266	273	0.999053421	+	.	TF=HOXB4;class=Homeo domain factors;sequence=ATCATTAA
ZEB1|NM_001174095	TFBS	TFBS	266	273	0.999053421	+	.	TF=HOXB4;class=Homeo domain factors;sequence=ATCATTAA
ZEB1|NM_001323646	TFBS	TFBS	664	671	0.999053421	+	.	TF=HOXB4;class=Homeo domain factors;sequence=ATCATTAA
ZEB1|NM_001323645	TFBS	TFBS	664	671	0.999053421	+	.	TF=HOXB4;class=Homeo domain factors;sequence=ATCATTAA
ZEB1|NM_001174093	TFBS	TFBS	266	273	0.999053421	+	.	TF=HOXB4;class=Homeo domain factors;sequence=ATC

								ATTAA
ZEB1|NM_001323655	TFBS	TFBS	266	273	0.999053421	+	.	TF=HOXB4;class=Homeo domain factors;sequence=ATCATTAA
ZEB1|NM_001323654	TFBS	TFBS	266	273	0.999053421	+	.	TF=HOXB4;class=Homeo domain factors;sequence=ATCATTAA
ZEB1|NM_001323653	TFBS	TFBS	266	273	0.999053421	+	.	TF=HOXB4;class=Homeo domain factors;sequence=ATCATTAA
ZEB1|NM_001323652	TFBS	TFBS	266	273	0.999053421	+	.	TF=HOXB4;class=Homeo domain factors;sequence=ATCATTAA
ZEB1|NM_001323651	TFBS	TFBS	266	273	0.999053421	+	.	TF=HOXB4;class=Homeo domain factors;sequence=ATCATTAA
ZEB1|NM_001323677	TFBS	TFBS	1640	1647	0.999053421	+	.	TF=HOXB4;class=Homeo domain factors;sequence=ATCATTAA
ZEB1|NM_001323676	TFBS	TFBS	1640	1647	0.999053421	+	.	TF=HOXB4;class=Homeo domain factors;sequence=ATCATTAA
ZEB1|NM_001323649	TFBS	TFBS	266	273	0.999053421	+	.	TF=HOXB4;class=Homeo domain factors;sequence=ATCATTAA
ZEB1|NM_001323675	TFBS	TFBS	1640	1647	0.999053421	+	.	TF=HOXB4;class=Homeo domain factors;sequence=ATCATTAA
ZEB1|NM_001323672	TFBS	TFBS	1640	1647	0.999053421	+	.	TF=HOXB4;class=Homeo domain factors;sequence=ATCATTAA
ZEB1|NM_001323671	TFBS	TFBS	1640	1647	0.999053421	+	.	TF=HOXB4;class=Homeo domain factors;sequence=ATC

								ATTAA
ZEB1|NM_001323642	TFBS	TFBS	664	671	0.999053421	+	.	TF=HOXB4;class=Homeo domain factors;sequence=ATCATTAA
ZEB1|NM_001174096	TFBS	TFBS	266	273	0.999053421	+	.	TF=HOXB4;class=Homeo domain factors;sequence=ATCATTAA
ZEB1|NM_030751	TFBS	TFBS	266	273	0.999053421	+	.	TF=HOXB4;class=Homeo domain factors;sequence=ATCATTAA
ZEB1|NM_001323638	TFBS	TFBS	395	402	0.998888511	-	.	TF=HOXD4;class=Homeo domain factors;sequence=GTCATTAA
ZEB1|NM_001323643	TFBS	TFBS	395	402	0.998888511	-	.	TF=HOXD4;class=Homeo domain factors;sequence=GTCATTAA
ZEB1|NM_001128128	TFBS	TFBS	844	851	0.998478909	-	.	TF=PITX2;class=Homeo domain factors;sequence=CTAATCCC
ZEB1|NM_001323673	TFBS	TFBS	385	392	0.998478909	-	.	TF=PITX2;class=Homeo domain factors;sequence=CTAATCCC
ZEB1|NM_001323678	TFBS	TFBS	844	851	0.998478909	-	.	TF=PITX2;class=Homeo domain factors;sequence=CTAATCCC
ZEB1|NM_001323677	TFBS	TFBS	385	392	0.998478909	-	.	TF=PITX2;class=Homeo domain factors;sequence=CTAATCCC
ZEB1|NM_001323676	TFBS	TFBS	385	392	0.998478909	-	.	TF=PITX2;class=Homeo domain factors;sequence=CTAATCCC
ZEB1|NM_001323675	TFBS	TFBS	385	392	0.998478909	-	.	TF=PITX2;class=Homeo domain factors;sequence=CTA

								ATCCC
ZEB1|NM_001323672	TFBS	TFBS	3845	3922	0.998478909	-	.	TF=PITX2;class=Homeo domain factors;sequence=CTAATCCC
ZEB1|NM_001323671	TFBS	TFBS	3845	3922	0.998478909	-	.	TF=PITX2;class=Homeo domain factors;sequence=CTAATCCC
ZEB1|NM_001323666	TFBS	TFBS	844	851	0.998478909	-	.	TF=PITX2;class=Homeo domain factors;sequence=CTAATCCC
ZEB1|NM_001323665	TFBS	TFBS	844	851	0.998478909	-	.	TF=PITX2;class=Homeo domain factors;sequence=CTAATCCC
ZEB1|NM_001323664	TFBS	TFBS	844	851	0.998478909	-	.	TF=PITX2;class=Homeo domain factors;sequence=CTAATCCC
ZEB1|NM_001323663	TFBS	TFBS	844	851	0.998478909	-	.	TF=PITX2;class=Homeo domain factors;sequence=CTAATCCC
ZEB1|NM_001323662	TFBS	TFBS	844	851	0.998478909	-	.	TF=PITX2;class=Homeo domain factors;sequence=CTAATCCC
ZEB1|NM_001323661	TFBS	TFBS	1491	1498	0.998478909	-	.	TF=PITX2;class=Homeo domain factors;sequence=CTAATCCC
ZEB1|NM_001323660	TFBS	TFBS	1491	1498	0.998478909	-	.	TF=PITX2;class=Homeo domain factors;sequence=CTAATCCC
ZEB1|NM_001323659	TFBS	TFBS	1491	1498	0.998478909	-	.	TF=PITX2;class=Homeo domain factors;sequence=CTAATCCC
ZEB1|NM_001174094	TFBS	TFBS	844	851	0.998478909	-	.	TF=PITX2;class=Homeo domain factors;sequence=CTA

								ATCCC
ZEB1|NM_001323638	TFBS	TFBS	83	93	0.998457723	-	.	TF=GATA2;class=Other C4 zinc finger-type factors;sequence=TTCTTATCTCT
ZEB1|NM_001323643	TFBS	TFBS	83	93	0.998457723	-	.	TF=GATA2;class=Other C4 zinc finger-type factors;sequence=TTCTTATCTCT
ZEB1|NM_001323638	TFBS	TFBS	395	402	0.998106843	-	.	TF=HOXB4;class=Homeo domain factors;sequence=GTCATTAA
ZEB1|NM_001323643	TFBS	TFBS	395	402	0.998106843	-	.	TF=HOXB4;class=Homeo domain factors;sequence=GTCATTAA
ZEB1|NM_001323647	TFBS	TFBS	1907	1916	0.997317418	+	.	TF=HOXA9;class=Homeo domain factors;sequence=GTCGTAAACC
ZEB1|NM_001323674	TFBS	TFBS	1907	1916	0.997317418	+	.	TF=HOXA9;class=Homeo domain factors;sequence=GTCGTAAACC
ZEB1|NM_001323658	TFBS	TFBS	1355	1364	0.997317418	+	.	TF=HOXA9;class=Homeo domain factors;sequence=GTCGTAAACC
ZEB1|NM_001323657	TFBS	TFBS	1355	1364	0.997317418	+	.	TF=HOXA9;class=Homeo domain factors;sequence=GTCGTAAACC
ZEB1|NM_001323650	TFBS	TFBS	1907	1916	0.997317418	+	.	TF=HOXA9;class=Homeo domain factors;sequence=GTCGTAAACC
ZEB1|NM_001323648	TFBS	TFBS	1907	1916	0.997317418	+	.	TF=HOXA9;class=Homeo domain factors;sequence=GTCGTAAACC
ZEB1|NM_001174095	TFBS	TFBS	1907	1916	0.997317418	+	.	TF=HOXA9;class=Homeo domain factors;sequence=GTC

								GTAAACC
ZEB1|NM_001174093	TFBS	TFBS	1907	1916	0.997317418	+	.	TF=HOXA9;class=Homeo domain factors;sequence=GTCGTAAACC
ZEB1|NM_001323656	TFBS	TFBS	1355	1364	0.997317418	+	.	TF=HOXA9;class=Homeo domain factors;sequence=GTCGTAAACC
ZEB1|NM_001323655	TFBS	TFBS	1907	1916	0.997317418	+	.	TF=HOXA9;class=Homeo domain factors;sequence=GTCGTAAACC
ZEB1|NM_001323654	TFBS	TFBS	1907	1916	0.997317418	+	.	TF=HOXA9;class=Homeo domain factors;sequence=GTCGTAAACC
ZEB1|NM_001323653	TFBS	TFBS	1907	1916	0.997317418	+	.	TF=HOXA9;class=Homeo domain factors;sequence=GTCGTAAACC
ZEB1|NM_001323652	TFBS	TFBS	1907	1916	0.997317418	+	.	TF=HOXA9;class=Homeo domain factors;sequence=GTCGTAAACC
ZEB1|NM_001323651	TFBS	TFBS	1907	1916	0.997317418	+	.	TF=HOXA9;class=Homeo domain factors;sequence=GTCGTAAACC
ZEB1|NM_001323649	TFBS	TFBS	1907	1916	0.997317418	+	.	TF=HOXA9;class=Homeo domain factors;sequence=GTCGTAAACC
ZEB1|NM_001174096	TFBS	TFBS	1907	1916	0.997317418	+	.	TF=HOXA9;class=Homeo domain factors;sequence=GTCGTAAACC
ZEB1|NM_030751	TFBS	TFBS	1907	1916	0.997317418	+	.	TF=HOXA9;class=Homeo domain factors;sequence=GTCGTAAACC
ZEB1|NM_001323661	TFBS	TFBS	569	578	0.997317418	+	.	TF=HOXA9;class=Homeo domain factors;sequence=GTC

								GTAAACC
ZEB1|NM_0 01323660	TF BS	TF BS	56 9	57 8	0.9973 17418	+	.	TF=HOXA9;class=Ho meo domain factors;sequence=GTC GTAAACC
ZEB1|NM_0 01323659	TF BS	TF BS	56 9	57 8	0.9973 17418	+	.	TF=HOXA9;class=Ho meo domain factors;sequence=GTC GTAAACC
ZEB1|NM_0 01128128	TF BS	TF BS	12 90	13 00	0.9969 87594	-	.	TF=MAZ;class=C2H2 zinc finger factors;sequence=GCC CCCTCCCC
ZEB1|NM_0 01323673	TF BS	TF BS	83 1	84 1	0.9969 87594	-	.	TF=MAZ;class=C2H2 zinc finger factors;sequence=GCC CCCTCCCC
ZEB1|NM_0 01323678	TF BS	TF BS	12 90	13 00	0.9969 87594	-	.	TF=MAZ;class=C2H2 zinc finger factors;sequence=GCC CCCTCCCC
ZEB1|NM_0 01323677	TF BS	TF BS	83 1	84 1	0.9969 87594	-	.	TF=MAZ;class=C2H2 zinc finger factors;sequence=GCC CCCTCCCC
ZEB1|NM_0 01323676	TF BS	TF BS	83 1	84 1	0.9969 87594	-	.	TF=MAZ;class=C2H2 zinc finger factors;sequence=GCC CCCTCCCC
ZEB1|NM_0 01323675	TF BS	TF BS	83 1	84 1	0.9969 87594	-	.	TF=MAZ;class=C2H2 zinc finger factors;sequence=GCC CCCTCCCC
ZEB1|NM_0 01323672	TF BS	TF BS	83 1	84 1	0.9969 87594	-	.	TF=MAZ;class=C2H2 zinc finger factors;sequence=GCC CCCTCCCC
ZEB1|NM_0 01323671	TF BS	TF BS	83 1	84 1	0.9969 87594	-	.	TF=MAZ;class=C2H2 zinc finger factors;sequence=GCC CCCTCCCC
ZEB1|NM_0 01323666	TF BS	TF BS	12 90	13 00	0.9969 87594	-	.	TF=MAZ;class=C2H2 zinc finger factors;sequence=GCC

								CCCTCCCC
ZEB1|NM_001323665	TFBS	TFBS	1290	1300	0.996987594	-	.	TF=MAZ;class=C2H2 zinc finger factors;sequence=GCCCCCTCCCC
ZEB1|NM_001323664	TFBS	TFBS	1290	1300	0.996987594	-	.	TF=MAZ;class=C2H2 zinc finger factors;sequence=GCCCCCTCCCC
ZEB1|NM_001323663	TFBS	TFBS	1290	1300	0.996987594	-	.	TF=MAZ;class=C2H2 zinc finger factors;sequence=GCCCCCTCCCC
ZEB1|NM_001323662	TFBS	TFBS	1290	1300	0.996987594	-	.	TF=MAZ;class=C2H2 zinc finger factors;sequence=GCCCCCTCCCC
ZEB1|NM_001323661	TFBS	TFBS	1937	1947	0.996987594	-	.	TF=MAZ;class=C2H2 zinc finger factors;sequence=GCCCCCTCCCC
ZEB1|NM_001323660	TFBS	TFBS	1937	1947	0.996987594	-	.	TF=MAZ;class=C2H2 zinc finger factors;sequence=GCCCCCTCCCC
ZEB1|NM_001323659	TFBS	TFBS	1937	1947	0.996987594	-	.	TF=MAZ;class=C2H2 zinc finger factors;sequence=GCCCCCTCCCC
ZEB1|NM_001174094	TFBS	TFBS	1290	1300	0.996987594	-	.	TF=MAZ;class=C2H2 zinc finger factors;sequence=GCCCCCTCCCC
ZEB1|NM_001323647	TFBS	TFBS	86	93	0.996978314	+	.	TF=NR2C2(var.2);class=Nuclear receptors with C4 zinc fingers;sequence=GGGGTCAA
ZEB1|NM_001323641	TFBS	TFBS	484	491	0.996978314	+	.	TF=NR2C2(var.2);class=Nuclear receptors with C4 zinc fingers;sequence=GGGGTCAA
ZEB1|NM_0	TF	TF	88	89	0.9969	+	.	TF=NR2C2(var.2);class

<table>
<tr><td>01323638</td><td>BS</td><td>BS</td><td>5</td><td>2</td><td>78314</td><td></td><td></td><td>=Nuclear receptors with C4 zinc fingers;sequence=GGG GTCAA</td></tr>
<tr><td>ZEB1|NM_0 01323644</td><td>TF BS</td><td>TF BS</td><td>48 4</td><td>49 1</td><td>0.9969 78314</td><td>+</td><td>.</td><td>TF=NR2C2(var.2);class =Nuclear receptors with C4 zinc fingers;sequence=GGG GTCAA</td></tr>
<tr><td>ZEB1|NM_0 01323643</td><td>TF BS</td><td>TF BS</td><td>88 5</td><td>89 2</td><td>0.9969 78314</td><td>+</td><td>.</td><td>TF=NR2C2(var.2);class =Nuclear receptors with C4 zinc fingers;sequence=GGG GTCAA</td></tr>
<tr><td>ZEB1|NM_0 01323674</td><td>TF BS</td><td>TF BS</td><td>86</td><td>93</td><td>0.9969 78314</td><td>+</td><td>.</td><td>TF=NR2C2(var.2);class =Nuclear receptors with C4 zinc fingers;sequence=GGG GTCAA</td></tr>
<tr><td>ZEB1|NM_0 01323650</td><td>TF BS</td><td>TF BS</td><td>86</td><td>93</td><td>0.9969 78314</td><td>+</td><td>.</td><td>TF=NR2C2(var.2);class =Nuclear receptors with C4 zinc fingers;sequence=GGG GTCAA</td></tr>
<tr><td>ZEB1|NM_0 01323648</td><td>TF BS</td><td>TF BS</td><td>86</td><td>93</td><td>0.9969 78314</td><td>+</td><td>.</td><td>TF=NR2C2(var.2);class =Nuclear receptors with C4 zinc fingers;sequence=GGG GTCAA</td></tr>
<tr><td>ZEB1|NM_0 01174095</td><td>TF BS</td><td>TF BS</td><td>86</td><td>93</td><td>0.9969 78314</td><td>+</td><td>.</td><td>TF=NR2C2(var.2);class =Nuclear receptors with C4 zinc fingers;sequence=GGG GTCAA</td></tr>
<tr><td>ZEB1|NM_0 01323646</td><td>TF BS</td><td>TF BS</td><td>48 4</td><td>49 1</td><td>0.9969 78314</td><td>+</td><td>.</td><td>TF=NR2C2(var.2);class =Nuclear receptors with C4 zinc fingers;sequence=GGG GTCAA</td></tr>
<tr><td>ZEB1|NM_0 01323645</td><td>TF BS</td><td>TF BS</td><td>48 4</td><td>49 1</td><td>0.9969 78314</td><td>+</td><td>.</td><td>TF=NR2C2(var.2);class =Nuclear receptors with C4 zinc fingers;sequence=GGG GTCAA</td></tr>
</table>

ZEB1|NM_001174093	TFBS	TFBS	86	93	0.996978314	+	.	TF=NR2C2(var.2);class=Nuclear receptors with C4 zinc fingers;sequence=GGGGTCAA
ZEB1|NM_001323655	TFBS	TFBS	86	93	0.996978314	+	.	TF=NR2C2(var.2);class=Nuclear receptors with C4 zinc fingers;sequence=GGGGTCAA
ZEB1|NM_001323654	TFBS	TFBS	86	93	0.996978314	+	.	TF=NR2C2(var.2);class=Nuclear receptors with C4 zinc fingers;sequence=GGGGTCAA
ZEB1|NM_001323653	TFBS	TFBS	86	93	0.996978314	+	.	TF=NR2C2(var.2);class=Nuclear receptors with C4 zinc fingers;sequence=GGGGTCAA
ZEB1|NM_001323652	TFBS	TFBS	86	93	0.996978314	+	.	TF=NR2C2(var.2);class=Nuclear receptors with C4 zinc fingers;sequence=GGGGTCAA
ZEB1|NM_001323651	TFBS	TFBS	86	93	0.996978314	+	.	TF=NR2C2(var.2);class=Nuclear receptors with C4 zinc fingers;sequence=GGGGTCAA
ZEB1|NM_001323649	TFBS	TFBS	86	93	0.996978314	+	.	TF=NR2C2(var.2);class=Nuclear receptors with C4 zinc fingers;sequence=GGGGTCAA
ZEB1|NM_001323642	TFBS	TFBS	484	491	0.996978314	+	.	TF=NR2C2(var.2);class=Nuclear receptors with C4 zinc fingers;sequence=GGGGTCAA
ZEB1|NM_001174096	TFBS	TFBS	86	93	0.996978314	+	.	TF=NR2C2(var.2);class=Nuclear receptors with C4 zinc fingers;sequence=GGG

								GTCAA
ZEB1|NM_0 30751	TF BS	TF BS	86	93	0.9969 78314	+	.	TF=NR2C2(var.2);class =Nuclear receptors with C4 zinc fingers;sequence=GGG GTCAA
ZEB1|NM_0 01128128	TF BS	TF BS	84 4	85 1	0.9969 68423	-	.	TF=RHOXF1;class=Ho meo domain factors;sequence=CTA ATCCC
ZEB1|NM_0 01323673	TF BS	TF BS	38 5	39 2	0.9969 68423	-	.	TF=RHOXF1;class=Ho meo domain factors;sequence=CTA ATCCC
ZEB1|NM_0 01323678	TF BS	TF BS	84 4	85 1	0.9969 68423	-	.	TF=RHOXF1;class=Ho meo domain factors;sequence=CTA ATCCC
ZEB1|NM_0 01323677	TF BS	TF BS	38 5	39 2	0.9969 68423	-	.	TF=RHOXF1;class=Ho meo domain factors;sequence=CTA ATCCC
ZEB1|NM_0 01323676	TF BS	TF BS	38 5	39 2	0.9969 68423	-	.	TF=RHOXF1;class=Ho meo domain factors;sequence=CTA ATCCC
ZEB1|NM_0 01323675	TF BS	TF BS	38 5	39 2	0.9969 68423	-	.	TF=RHOXF1;class=Ho meo domain factors;sequence=CTA ATCCC
ZEB1|NM_0 01323672	TF BS	TF BS	38 5	39 2	0.9969 68423	-	.	TF=RHOXF1;class=Ho meo domain factors;sequence=CTA ATCCC
ZEB1|NM_0 01323671	TF BS	TF BS	38 5	39 2	0.9969 68423	-	.	TF=RHOXF1;class=Ho meo domain factors;sequence=CTA ATCCC
ZEB1|NM_0 01323666	TF BS	TF BS	84 4	85 1	0.9969 68423	-	.	TF=RHOXF1;class=Ho meo domain factors;sequence=CTA ATCCC
ZEB1|NM_0 01323665	TF BS	TF BS	84 4	85 1	0.9969 68423	-	.	TF=RHOXF1;class=Ho meo domain

								factors;sequence=CTAATCCC
ZEB1|NM_001323664	TFBS	TFBS	844	851	0.996968423	-	.	TF=RHOXF1;class=Homeo domain factors;sequence=CTAATCCC
ZEB1|NM_001323663	TFBS	TFBS	844	851	0.996968423	-	.	TF=RHOXF1;class=Homeo domain factors;sequence=CTAATCCC
ZEB1|NM_001323662	TFBS	TFBS	844	851	0.996968423	-	.	TF=RHOXF1;class=Homeo domain factors;sequence=CTAATCCC
ZEB1|NM_001323661	TFBS	TFBS	1491	1498	0.996968423	-	.	TF=RHOXF1;class=Homeo domain factors;sequence=CTAATCCC
ZEB1|NM_001323660	TFBS	TFBS	1491	1498	0.996968423	-	.	TF=RHOXF1;class=Homeo domain factors;sequence=CTAATCCC
ZEB1|NM_001323659	TFBS	TFBS	1491	1498	0.996968423	-	.	TF=RHOXF1;class=Homeo domain factors;sequence=CTAATCCC
ZEB1|NM_001174094	TFBS	TFBS	844	851	0.996968423	-	.	TF=RHOXF1;class=Homeo domain factors;sequence=CTAATCCC
ZEB1|NM_001128128	TFBS	TFBS	1338	1347	0.996448102	-	.	TF=BHLHE22(var.2);class=Basic helix-loop-helix factors (bHLH);sequence=TGCAGCTGCG
ZEB1|NM_001323673	TFBS	TFBS	879	888	0.996448102	-	.	TF=BHLHE22(var.2);class=Basic helix-loop-helix factors (bHLH);sequence=TGCAGCTGCG
ZEB1|NM_001323678	TFBS	TFBS	1338	1347	0.996448102	-	.	TF=BHLHE22(var.2);class=Basic helix-loop-helix factors (bHLH);sequence=TGC

								AGCTGCG
ZEB1|NM_001323677	TFBS	TFBS	879	888	0.996448102	-	.	TF=BHLHE22(var.2);class=Basic helix-loop-helix factors (bHLH);sequence=TGCAGCTGCG
ZEB1|NM_001323676	TFBS	TFBS	879	888	0.996448102	-	.	TF=BHLHE22(var.2);class=Basic helix-loop-helix factors (bHLH);sequence=TGCAGCTGCG
ZEB1|NM_001323675	TFBS	TFBS	879	888	0.996448102	-	.	TF=BHLHE22(var.2);class=Basic helix-loop-helix factors (bHLH);sequence=TGCAGCTGCG
ZEB1|NM_001323672	TFBS	TFBS	879	888	0.996448102	-	.	TF=BHLHE22(var.2);class=Basic helix-loop-helix factors (bHLH);sequence=TGCAGCTGCG
ZEB1|NM_001323671	TFBS	TFBS	879	888	0.996448102	-	.	TF=BHLHE22(var.2);class=Basic helix-loop-helix factors (bHLH);sequence=TGCAGCTGCG
ZEB1|NM_001323666	TFBS	TFBS	1338	1347	0.996448102	-	.	TF=BHLHE22(var.2);class=Basic helix-loop-helix factors (bHLH);sequence=TGCAGCTGCG
ZEB1|NM_001323665	TFBS	TFBS	1338	1347	0.996448102	-	.	TF=BHLHE22(var.2);class=Basic helix-loop-helix factors (bHLH);sequence=TGCAGCTGCG
ZEB1|NM_001323664	TFBS	TFBS	1338	1347	0.996448102	-	.	TF=BHLHE22(var.2);class=Basic helix-loop-helix factors (bHLH);sequence=TGCAGCTGCG
ZEB1|NM_001323663	TFBS	TFBS	1338	1347	0.996448102	-	.	TF=BHLHE22(var.2);class=Basic helix-loop-helix factors

								(bHLH);sequence=TGC AGCTGCG
ZEB1|NM_0 01323662	TF BS	TF BS	13 38	13 47	0.9964 48102	-	.	TF=BHLHE22(var.2);cl ass=Basic helix-loop-helix factors (bHLH);sequence=TGC AGCTGCG
ZEB1|NM_0 01323661	TF BS	TF BS	19 85	19 94	0.9964 48102	-	.	TF=BHLHE22(var.2);cl ass=Basic helix-loop-helix factors (bHLH);sequence=TGC AGCTGCG
ZEB1|NM_0 01323660	TF BS	TF BS	19 85	19 94	0.9964 48102	-	.	TF=BHLHE22(var.2);cl ass=Basic helix-loop-helix factors (bHLH);sequence=TGC AGCTGCG
ZEB1|NM_0 01323659	TF BS	TF BS	19 85	19 94	0.9964 48102	-	.	TF=BHLHE22(var.2);cl ass=Basic helix-loop-helix factors (bHLH);sequence=TGC AGCTGCG
ZEB1|NM_0 01174094	TF BS	TF BS	13 38	13 47	0.9964 48102	-	.	TF=BHLHE22(var.2);cl ass=Basic helix-loop-helix factors (bHLH);sequence=TGC AGCTGCG
ZEB1|NM_0 01128128	TF BS	TF BS	13 38	13 47	0.9964 24556	+	.	TF=BHLHE22(var.2);cl ass=Basic helix-loop-helix factors (bHLH);sequence=CGC AGCTGCA
ZEB1|NM_0 01323673	TF BS	TF BS	87 9	88 8	0.9964 24556	+	.	TF=BHLHE22(var.2);cl ass=Basic helix-loop-helix factors (bHLH);sequence=CGC AGCTGCA
ZEB1|NM_0 01323678	TF BS	TF BS	13 38	13 47	0.9964 24556	+	.	TF=BHLHE22(var.2);cl ass=Basic helix-loop-helix factors (bHLH);sequence=CGC AGCTGCA
ZEB1|NM_0 01323677	TF BS	TF BS	87 9	88 8	0.9964 24556	+	.	TF=BHLHE22(var.2);cl ass=Basic

								helix-loop-helix factors (bHLH);sequence=CGCAGCTGCA
ZEB1|NM_001323676	TFBS	TFBS	879	888	0.996424556	+	.	TF=BHLHE22(var.2);class=Basic helix-loop-helix factors (bHLH);sequence=CGCAGCTGCA
ZEB1|NM_001323675	TFBS	TFBS	879	888	0.996424556	+	.	TF=BHLHE22(var.2);class=Basic helix-loop-helix factors (bHLH);sequence=CGCAGCTGCA
ZEB1|NM_001323672	TFBS	TFBS	879	888	0.996424556	+	.	TF=BHLHE22(var.2);class=Basic helix-loop-helix factors (bHLH);sequence=CGCAGCTGCA
ZEB1|NM_001323671	TFBS	TFBS	879	888	0.996424556	+	.	TF=BHLHE22(var.2);class=Basic helix-loop-helix factors (bHLH);sequence=CGCAGCTGCA
ZEB1|NM_001323666	TFBS	TFBS	1338	1347	0.996424556	+	.	TF=BHLHE22(var.2);class=Basic helix-loop-helix factors (bHLH);sequence=CGCAGCTGCA
ZEB1|NM_001323665	TFBS	TFBS	1338	1347	0.996424556	+	.	TF=BHLHE22(var.2);class=Basic helix-loop-helix factors (bHLH);sequence=CGCAGCTGCA
ZEB1|NM_001323664	TFBS	TFBS	1338	1347	0.996424556	+	.	TF=BHLHE22(var.2);class=Basic helix-loop-helix factors (bHLH);sequence=CGCAGCTGCA
ZEB1|NM_001323663	TFBS	TFBS	1338	1347	0.996424556	+	.	TF=BHLHE22(var.2);class=Basic helix-loop-helix factors (bHLH);sequence=CGCAGCTGCA
ZEB1|NM_0	TF	TF	13	13	0.9964	+	.	TF=BHLHE22(var.2);cl

01323662	BS	BS	38	47	24556			ass=Basic helix-loop-helix factors (bHLH);sequence=CGCAGCTGCA
ZEB1|NM_001323661	TF BS	TF BS	1985	1994	0.996424556	+	.	TF=BHLHE22(var.2);class=Basic helix-loop-helix factors (bHLH);sequence=CGCAGCTGCA
ZEB1|NM_001323660	TF BS	TF BS	1985	1994	0.996424556	+	.	TF=BHLHE22(var.2);class=Basic helix-loop-helix factors (bHLH);sequence=CGCAGCTGCA
ZEB1|NM_001323659	TF BS	TF BS	1985	1994	0.996424556	+	.	TF=BHLHE22(var.2);class=Basic helix-loop-helix factors (bHLH);sequence=CGCAGCTGCA
ZEB1|NM_001174094	TF BS	TF BS	1338	1347	0.996424556	+	.	TF=BHLHE22(var.2);class=Basic helix-loop-helix factors (bHLH);sequence=CGCAGCTGCA
ZEB1|NM_001323647	TF BS	TF BS	2241	2247	0.996305873	+	.	TF=FOXL1;class=Fork head / winged helix factors;sequence=ATAAACA
ZEB1|NM_001128128	TF BS	TF BS	2241	2247	0.996305873	+	.	TF=FOXL1;class=Fork head / winged helix factors;sequence=ATAAACA
ZEB1|NM_001128128	TF BS	TF BS	1349	1355	0.996305873	-	.	TF=FOXL1;class=Fork head / winged helix factors;sequence=ATAAACA
ZEB1|NM_001323641	TF BS	TF BS	2241	2247	0.996305873	+	.	TF=FOXL1;class=Fork head / winged helix factors;sequence=ATAAACA
ZEB1|NM_001323641	TF BS	TF BS	122	128	0.996305873	-	.	TF=FOXL1;class=Fork head / winged helix factors;sequence=ATAAACA

ZEB1|NM_001323638	TF BS	TF BS	2241	2247	0.996305873	+	.	TF=FOXL1;class=Fork head / winged helix factors;sequence=ATAAACA
ZEB1|NM_001323638	TF BS	TF BS	523	529	0.996305873	-	.	TF=FOXL1;class=Fork head / winged helix factors;sequence=ATAAACA
ZEB1|NM_001323644	TF BS	TF BS	2241	2247	0.996305873	+	.	TF=FOXL1;class=Fork head / winged helix factors;sequence=ATAAACA
ZEB1|NM_001323644	TF BS	TF BS	122	128	0.996305873	-	.	TF=FOXL1;class=Fork head / winged helix factors;sequence=ATAAACA
ZEB1|NM_001323643	TF BS	TF BS	2241	2247	0.996305873	+	.	TF=FOXL1;class=Fork head / winged helix factors;sequence=ATAAACA
ZEB1|NM_001323643	TF BS	TF BS	523	529	0.996305873	-	.	TF=FOXL1;class=Fork head / winged helix factors;sequence=ATAAACA
ZEB1|NM_001323674	TF BS	TF BS	2241	2247	0.996305873	+	.	TF=FOXL1;class=Fork head / winged helix factors;sequence=ATAAACA
ZEB1|NM_001323673	TF BS	TF BS	1679	1685	0.996305873	+	.	TF=FOXL1;class=Fork head / winged helix factors;sequence=ATAAACA
ZEB1|NM_001323673	TF BS	TF BS	2241	2247	0.996305873	+	.	TF=FOXL1;class=Fork head / winged helix factors;sequence=ATAAACA
ZEB1|NM_001323673	TF BS	TF BS	890	896	0.996305873	-	.	TF=FOXL1;class=Fork head / winged helix factors;sequence=ATAAACA
ZEB1|NM_001323658	TF BS	TF BS	2241	2247	0.996305873	+	.	TF=FOXL1;class=Fork head / winged helix factors;sequence=ATAAACA

ZEB1|NM_001323657	TFBS	TFBS	2241	2247	0.996305873	+	.	TF=FOXL1;class=Fork head / winged helix factors;sequence=ATAAACA
ZEB1|NM_001323650	TFBS	TFBS	2241	2247	0.996305873	+	.	TF=FOXL1;class=Fork head / winged helix factors;sequence=ATAAACA
ZEB1|NM_001323648	TFBS	TFBS	2241	2247	0.996305873	+	.	TF=FOXL1;class=Fork head / winged helix factors;sequence=ATAAACA
ZEB1|NM_001174095	TFBS	TFBS	2241	2247	0.996305873	+	.	TF=FOXL1;class=Fork head / winged helix factors;sequence=ATAAACA
ZEB1|NM_001323646	TFBS	TFBS	2241	2247	0.996305873	+	.	TF=FOXL1;class=Fork head / winged helix factors;sequence=ATAAACA
ZEB1|NM_001323646	TFBS	TFBS	122	128	0.996305873	-	.	TF=FOXL1;class=Fork head / winged helix factors;sequence=ATAAACA
ZEB1|NM_001323645	TFBS	TFBS	2241	2247	0.996305873	+	.	TF=FOXL1;class=Fork head / winged helix factors;sequence=ATAAACA
ZEB1|NM_001323645	TFBS	TFBS	122	128	0.996305873	-	.	TF=FOXL1;class=Fork head / winged helix factors;sequence=ATAAACA
ZEB1|NM_001174093	TFBS	TFBS	2241	2247	0.996305873	+	.	TF=FOXL1;class=Fork head / winged helix factors;sequence=ATAAACA
ZEB1|NM_001323656	TFBS	TFBS	2241	2247	0.996305873	+	.	TF=FOXL1;class=Fork head / winged helix factors;sequence=ATAAACA
ZEB1|NM_001323655	TFBS	TFBS	2241	2247	0.996305873	+	.	TF=FOXL1;class=Fork head / winged helix factors;sequence=ATAAACA

ZEB1|NM_001323654	TF BS	TF BS	2241	2247	0.996305873	+	.	TF=FOXL1;class=Fork head / winged helix factors;sequence=ATAAACA
ZEB1|NM_001323653	TF BS	TF BS	2241	2247	0.996305873	+	.	TF=FOXL1;class=Fork head / winged helix factors;sequence=ATAAACA
ZEB1|NM_001323652	TF BS	TF BS	2241	2247	0.996305873	+	.	TF=FOXL1;class=Fork head / winged helix factors;sequence=ATAAACA
ZEB1|NM_001323651	TF BS	TF BS	2241	2247	0.996305873	+	.	TF=FOXL1;class=Fork head / winged helix factors;sequence=ATAAACA
ZEB1|NM_001323678	TF BS	TF BS	2241	2247	0.996305873	+	.	TF=FOXL1;class=Fork head / winged helix factors;sequence=ATAAACA
ZEB1|NM_001323678	TF BS	TF BS	1349	1355	0.996305873	-	.	TF=FOXL1;class=Fork head / winged helix factors;sequence=ATAAACA
ZEB1|NM_001323677	TF BS	TF BS	1679	1685	0.996305873	+	.	TF=FOXL1;class=Fork head / winged helix factors;sequence=ATAAACA
ZEB1|NM_001323677	TF BS	TF BS	2241	2247	0.996305873	+	.	TF=FOXL1;class=Fork head / winged helix factors;sequence=ATAAACA
ZEB1|NM_001323677	TF BS	TF BS	890	896	0.996305873	-	.	TF=FOXL1;class=Fork head / winged helix factors;sequence=ATAAACA
ZEB1|NM_001323676	TF BS	TF BS	1679	1685	0.996305873	+	.	TF=FOXL1;class=Fork head / winged helix factors;sequence=ATAAACA
ZEB1|NM_001323676	TF BS	TF BS	2241	2247	0.996305873	+	.	TF=FOXL1;class=Fork head / winged helix factors;sequence=ATAAACA

ZEB1|NM_001323676	TFBS	TFBS	890	896	0.996305873	-	.	TF=FOXL1;class=Fork head / winged helix factors;sequence=ATAAACA
ZEB1|NM_001323649	TFBS	TFBS	2241	2247	0.996305873	+	.	TF=FOXL1;class=Fork head / winged helix factors;sequence=ATAAACA
ZEB1|NM_001323675	TFBS	TFBS	1679	1685	0.996305873	+	.	TF=FOXL1;class=Fork head / winged helix factors;sequence=ATAAACA
ZEB1|NM_001323675	TFBS	TFBS	2241	2247	0.996305873	+	.	TF=FOXL1;class=Fork head / winged helix factors;sequence=ATAAACA
ZEB1|NM_001323675	TFBS	TFBS	890	896	0.996305873	-	.	TF=FOXL1;class=Fork head / winged helix factors;sequence=ATAAACA
ZEB1|NM_001323672	TFBS	TFBS	1679	1685	0.996305873	+	.	TF=FOXL1;class=Fork head / winged helix factors;sequence=ATAAACA
ZEB1|NM_001323672	TFBS	TFBS	2241	2247	0.996305873	+	.	TF=FOXL1;class=Fork head / winged helix factors;sequence=ATAAACA
ZEB1|NM_001323672	TFBS	TFBS	890	896	0.996305873	-	.	TF=FOXL1;class=Fork head / winged helix factors;sequence=ATAAACA
ZEB1|NM_001323671	TFBS	TFBS	1679	1685	0.996305873	+	.	TF=FOXL1;class=Fork head / winged helix factors;sequence=ATAAACA
ZEB1|NM_001323671	TFBS	TFBS	2241	2247	0.996305873	+	.	TF=FOXL1;class=Fork head / winged helix factors;sequence=ATAAACA
ZEB1|NM_001323671	TFBS	TFBS	890	896	0.996305873	-	.	TF=FOXL1;class=Fork head / winged helix factors;sequence=ATAAACA

ZEB1|NM_001323666	TFBS	TFBS	2241	2247	0.996305873	+	.	TF=FOXL1;class=Fork head / winged helix factors;sequence=ATAAACA
ZEB1|NM_001323666	TFBS	TFBS	1349	1355	0.996305873	-	.	TF=FOXL1;class=Fork head / winged helix factors;sequence=ATAAACA
ZEB1|NM_001323642	TFBS	TFBS	2241	2247	0.996305873	+	.	TF=FOXL1;class=Fork head / winged helix factors;sequence=ATAAACA
ZEB1|NM_001323642	TFBS	TFBS	122	128	0.996305873	-	.	TF=FOXL1;class=Fork head / winged helix factors;sequence=ATAAACA
ZEB1|NM_001174096	TFBS	TFBS	2241	2247	0.996305873	+	.	TF=FOXL1;class=Fork head / winged helix factors;sequence=ATAAACA
ZEB1|NM_030751	TFBS	TFBS	2241	2247	0.996305873	+	.	TF=FOXL1;class=Fork head / winged helix factors;sequence=ATAAACA
ZEB1|NM_001323665	TFBS	TFBS	2241	2247	0.996305873	+	.	TF=FOXL1;class=Fork head / winged helix factors;sequence=ATAAACA
ZEB1|NM_001323665	TFBS	TFBS	1349	1355	0.996305873	-	.	TF=FOXL1;class=Fork head / winged helix factors;sequence=ATAAACA
ZEB1|NM_001323664	TFBS	TFBS	2241	2247	0.996305873	+	.	TF=FOXL1;class=Fork head / winged helix factors;sequence=ATAAACA
ZEB1|NM_001323664	TFBS	TFBS	1349	1355	0.996305873	-	.	TF=FOXL1;class=Fork head / winged helix factors;sequence=ATAAACA
ZEB1|NM_001323663	TFBS	TFBS	2241	2247	0.996305873	+	.	TF=FOXL1;class=Fork head / winged helix factors;sequence=ATAAACA

ZEB1|NM_001323663	TF BS	TF BS	1349	1355	0.996305873	-	.	TF=FOXL1;class=Fork head / winged helix factors;sequence=ATAAACA
ZEB1|NM_001323662	TF BS	TF BS	2241	2247	0.996305873	+	.	TF=FOXL1;class=Fork head / winged helix factors;sequence=ATAAACA
ZEB1|NM_001323662	TF BS	TF BS	1349	1355	0.996305873	-	.	TF=FOXL1;class=Fork head / winged helix factors;sequence=ATAAACA
ZEB1|NM_001323661	TF BS	TF BS	2241	2247	0.996305873	+	.	TF=FOXL1;class=Fork head / winged helix factors;sequence=ATAAACA
ZEB1|NM_001323660	TF BS	TF BS	2241	2247	0.996305873	+	.	TF=FOXL1;class=Fork head / winged helix factors;sequence=ATAAACA
ZEB1|NM_001323659	TF BS	TF BS	2241	2247	0.996305873	+	.	TF=FOXL1;class=Fork head / winged helix factors;sequence=ATAAACA
ZEB1|NM_001174094	TF BS	TF BS	2241	2247	0.996305873	+	.	TF=FOXL1;class=Fork head / winged helix factors;sequence=ATAAACA
ZEB1|NM_001174094	TF BS	TF BS	1349	1355	0.996305873	-	.	TF=FOXL1;class=Fork head / winged helix factors;sequence=ATAAACA
ZEB1|NM_001323647	TF BS	TF BS	276	283	0.99620351	-	.	TF=HOXA4;class=Homeo domain factors;sequence=ATCATTAG
ZEB1|NM_001323641	TF BS	TF BS	674	681	0.99620351	-	.	TF=HOXA4;class=Homeo domain factors;sequence=ATCATTAG
ZEB1|NM_001323638	TF BS	TF BS	1075	1082	0.99620351	-	.	TF=HOXA4;class=Homeo domain factors;sequence=ATCATTAG

ZEB1|NM_001323644	TFBS	TFBS	674	681	0.99620351	-	.	TF=HOXA4;class=Homeo domain factors;sequence=ATCATTAG
ZEB1|NM_001323643	TFBS	TFBS	1075	1082	0.99620351	-	.	TF=HOXA4;class=Homeo domain factors;sequence=ATCATTAG
ZEB1|NM_001323674	TFBS	TFBS	276	283	0.99620351	-	.	TF=HOXA4;class=Homeo domain factors;sequence=ATCATTAG
ZEB1|NM_001323650	TFBS	TFBS	276	283	0.99620351	-	.	TF=HOXA4;class=Homeo domain factors;sequence=ATCATTAG
ZEB1|NM_001323648	TFBS	TFBS	276	283	0.99620351	-	.	TF=HOXA4;class=Homeo domain factors;sequence=ATCATTAG
ZEB1|NM_001174095	TFBS	TFBS	276	283	0.99620351	-	.	TF=HOXA4;class=Homeo domain factors;sequence=ATCATTAG
ZEB1|NM_001323646	TFBS	TFBS	674	681	0.99620351	-	.	TF=HOXA4;class=Homeo domain factors;sequence=ATCATTAG
ZEB1|NM_001323645	TFBS	TFBS	674	681	0.99620351	-	.	TF=HOXA4;class=Homeo domain factors;sequence=ATCATTAG
ZEB1|NM_001174093	TFBS	TFBS	276	283	0.99620351	-	.	TF=HOXA4;class=Homeo domain factors;sequence=ATCATTAG
ZEB1|NM_001323655	TFBS	TFBS	276	283	0.99620351	-	.	TF=HOXA4;class=Homeo domain factors;sequence=ATCATTAG
ZEB1|NM_001323654	TFBS	TFBS	276	283	0.99620351	-	.	TF=HOXA4;class=Homeo domain factors;sequence=ATCATTAG

ZEB1|NM_001323653	TFBS	TFBS	276	283	0.99620351	-	.	TF=HOXA4;class=Homeo domain factors;sequence=ATCATTAG
ZEB1|NM_001323652	TFBS	TFBS	276	283	0.99620351	-	.	TF=HOXA4;class=Homeo domain factors;sequence=ATCATTAG
ZEB1|NM_001323651	TFBS	TFBS	276	283	0.99620351	-	.	TF=HOXA4;class=Homeo domain factors;sequence=ATCATTAG
ZEB1|NM_001323649	TFBS	TFBS	276	283	0.99620351	-	.	TF=HOXA4;class=Homeo domain factors;sequence=ATCATTAG
ZEB1|NM_001323642	TFBS	TFBS	674	681	0.99620351	-	.	TF=HOXA4;class=Homeo domain factors;sequence=ATCATTAG
ZEB1|NM_001174096	TFBS	TFBS	276	283	0.99620351	-	.	TF=HOXA4;class=Homeo domain factors;sequence=ATCATTAG
ZEB1|NM_030751	TFBS	TFBS	276	283	0.99620351	-	.	TF=HOXA4;class=Homeo domain factors;sequence=ATCATTAG
ZEB1|NM_001323673	TFBS	TFBS	1667	1674	0.996179597	-	.	TF=HOXD4;class=Homeo domain factors;sequence=ATAATTAA
ZEB1|NM_001323677	TFBS	TFBS	1667	1674	0.996179597	-	.	TF=HOXD4;class=Homeo domain factors;sequence=ATAATTAA
ZEB1|NM_001323676	TFBS	TFBS	1667	1674	0.996179597	-	.	TF=HOXD4;class=Homeo domain factors;sequence=ATAATTAA
ZEB1|NM_001323675	TFBS	TFBS	1667	1674	0.996179597	-	.	TF=HOXD4;class=Homeo domain factors;sequence=ATAATTAA

ZEB1|NM_001323672	TFBS	TFBS	1667	1674	0.996179597	-	.	TF=HOXD4;class=Homeo domain factors;sequence=ATAATTAA
ZEB1|NM_001323671	TFBS	TFBS	1667	1674	0.996179597	-	.	TF=HOXD4;class=Homeo domain factors;sequence=ATAATTAA
ZEB1|NM_001128128	TFBS	TFBS	1997	2007	0.996093368	-	.	TF=HOXA10;class=Homeo domain factors;sequence=GGCAATAAAAA
ZEB1|NM_001323678	TFBS	TFBS	1997	2007	0.996093368	-	.	TF=HOXA10;class=Homeo domain factors;sequence=GGCAATAAAAA
ZEB1|NM_001323666	TFBS	TFBS	1997	2007	0.996093368	-	.	TF=HOXA10;class=Homeo domain factors;sequence=GGCAATAAAAA
ZEB1|NM_001323665	TFBS	TFBS	1997	2007	0.996093368	-	.	TF=HOXA10;class=Homeo domain factors;sequence=GGCAATAAAAA
ZEB1|NM_001323664	TFBS	TFBS	1997	2007	0.996093368	-	.	TF=HOXA10;class=Homeo domain factors;sequence=GGCAATAAAAA
ZEB1|NM_001323663	TFBS	TFBS	1997	2007	0.996093368	-	.	TF=HOXA10;class=Homeo domain factors;sequence=GGCAATAAAAA
ZEB1|NM_001323662	TFBS	TFBS	1997	2007	0.996093368	-	.	TF=HOXA10;class=Homeo domain factors;sequence=GGCAATAAAAA
ZEB1|NM_001174094	TFBS	TFBS	1997	2007	0.996093368	-	.	TF=HOXA10;class=Homeo domain factors;sequence=GGCAATAAAAA
ZEB1|NM_001323647	TFBS	TFBS	1997	2006	0.995777597	-	.	TF=HOXD9;class=Homeo domain factors;sequence=GCAATAAAAT

ZEB1|NM_001323674	TFBS	TFBS	1997	2006	0.995777597	-	.	TF=HOXD9;class=Homeo domain factors;sequence=GCAATAAAAT
ZEB1|NM_001323650	TFBS	TFBS	1997	2006	0.995777597	-	.	TF=HOXD9;class=Homeo domain factors;sequence=GCAATAAAAT
ZEB1|NM_001323648	TFBS	TFBS	1997	2006	0.995777597	-	.	TF=HOXD9;class=Homeo domain factors;sequence=GCAATAAAAT
ZEB1|NM_001174095	TFBS	TFBS	1997	2006	0.995777597	-	.	TF=HOXD9;class=Homeo domain factors;sequence=GCAATAAAAT
ZEB1|NM_001174093	TFBS	TFBS	1997	2006	0.995777597	-	.	TF=HOXD9;class=Homeo domain factors;sequence=GCAATAAAAT
ZEB1|NM_001323655	TFBS	TFBS	1997	2006	0.995777597	-	.	TF=HOXD9;class=Homeo domain factors;sequence=GCAATAAAAT
ZEB1|NM_001323654	TFBS	TFBS	1997	2006	0.995777597	-	.	TF=HOXD9;class=Homeo domain factors;sequence=GCAATAAAAT
ZEB1|NM_001323653	TFBS	TFBS	1997	2006	0.995777597	-	.	TF=HOXD9;class=Homeo domain factors;sequence=GCAATAAAAT
ZEB1|NM_001323652	TFBS	TFBS	1997	2006	0.995777597	-	.	TF=HOXD9;class=Homeo domain factors;sequence=GCAATAAAAT
ZEB1|NM_001323651	TFBS	TFBS	1997	2006	0.995777597	-	.	TF=HOXD9;class=Homeo domain factors;sequence=GCAATAAAAT
ZEB1|NM_001323649	TFBS	TFBS	1997	2006	0.995777597	-	.	TF=HOXD9;class=Homeo domain factors;sequence=GCAATAAAAT

ZEB1|NM_001174096	TF BS	TF BS	1997	2006	0.995777597	-	.	TF=HOXD9;class=Homeo domain factors;sequence=GCAATAAAAT
ZEB1|NM_030751	TF BS	TF BS	1997	2006	0.995777597	-	.	TF=HOXD9;class=Homeo domain factors;sequence=GCAATAAAAT
ZEB1|NM_001128128	TF BS	TF BS	1997	2006	0.995482444	-	.	TF=HOXD9;class=Homeo domain factors;sequence=GCAATAAAAA
ZEB1|NM_001323678	TF BS	TF BS	1997	2006	0.995482444	-	.	TF=HOXD9;class=Homeo domain factors;sequence=GCAATAAAAA
ZEB1|NM_001323666	TF BS	TF BS	1997	2006	0.995482444	-	.	TF=HOXD9;class=Homeo domain factors;sequence=GCAATAAAAA
ZEB1|NM_001323665	TF BS	TF BS	1997	2006	0.995482444	-	.	TF=HOXD9;class=Homeo domain factors;sequence=GCAATAAAAA
ZEB1|NM_001323664	TF BS	TF BS	1997	2006	0.995482444	-	.	TF=HOXD9;class=Homeo domain factors;sequence=GCAATAAAAA
ZEB1|NM_001323663	TF BS	TF BS	1997	2006	0.995482444	-	.	TF=HOXD9;class=Homeo domain factors;sequence=GCAATAAAAA
ZEB1|NM_001323662	TF BS	TF BS	1997	2006	0.995482444	-	.	TF=HOXD9;class=Homeo domain factors;sequence=GCAATAAAAA
ZEB1|NM_001174094	TF BS	TF BS	1997	2006	0.995482444	-	.	TF=HOXD9;class=Homeo domain factors;sequence=GCAATAAAAA
ZEB1|NM_001128128	TF BS	TF BS	1369	1377	0.995388085	+	.	TF=NFIX;class=SMAD/NF-1 DNA-binding domain factors;sequence=TGT

								GCCAAG
ZEB1|NM_001323673	TFBS	TFBS	910	918	0.995388085	+	.	TF=NFIX;class=SMAD/NF-1 DNA-binding domain factors;sequence=TGTGCCAAG
ZEB1|NM_001323678	TFBS	TFBS	1369	1377	0.995388085	+	.	TF=NFIX;class=SMAD/NF-1 DNA-binding domain factors;sequence=TGTGCCAAG
ZEB1|NM_001323677	TFBS	TFBS	910	918	0.995388085	+	.	TF=NFIX;class=SMAD/NF-1 DNA-binding domain factors;sequence=TGTGCCAAG
ZEB1|NM_001323676	TFBS	TFBS	910	918	0.995388085	+	.	TF=NFIX;class=SMAD/NF-1 DNA-binding domain factors;sequence=TGTGCCAAG
ZEB1|NM_001323675	TFBS	TFBS	910	918	0.995388085	+	.	TF=NFIX;class=SMAD/NF-1 DNA-binding domain factors;sequence=TGTGCCAAG
ZEB1|NM_001323672	TFBS	TFBS	910	918	0.995388085	+	.	TF=NFIX;class=SMAD/NF-1 DNA-binding domain factors;sequence=TGTGCCAAG
ZEB1|NM_001323671	TFBS	TFBS	910	918	0.995388085	+	.	TF=NFIX;class=SMAD/NF-1 DNA-binding domain factors;sequence=TGTGCCAAG
ZEB1|NM_001323666	TFBS	TFBS	1369	1377	0.995388085	+	.	TF=NFIX;class=SMAD/NF-1 DNA-binding domain factors;sequence=TGTGCCAAG
ZEB1|NM_001323665	TFBS	TFBS	1369	1377	0.995388085	+	.	TF=NFIX;class=SMAD/NF-1 DNA-binding domain

								factors;sequence=TGT GCCAAG
ZEB1|NM_0 01323664	TF BS	TF BS	13 69	13 77	0.9953 88085	+	.	TF=NFIX;class=SMAD /NF-1 DNA-binding domain factors;sequence=TGT GCCAAG
ZEB1|NM_0 01323663	TF BS	TF BS	13 69	13 77	0.9953 88085	+	.	TF=NFIX;class=SMAD /NF-1 DNA-binding domain factors;sequence=TGT GCCAAG
ZEB1|NM_0 01323662	TF BS	TF BS	13 69	13 77	0.9953 88085	+	.	TF=NFIX;class=SMAD /NF-1 DNA-binding domain factors;sequence=TGT GCCAAG
ZEB1|NM_0 01174094	TF BS	TF BS	13 69	13 77	0.9953 88085	+	.	TF=NFIX;class=SMAD /NF-1 DNA-binding domain factors;sequence=TGT GCCAAG
ZEB1|NM_0 01323638	TF BS	TF BS	39 5	40 2	0.9953 249	-	.	TF=HOXA4;class=Ho meo domain factors;sequence=GTC ATTAA
ZEB1|NM_0 01323643	TF BS	TF BS	39 5	40 2	0.9953 249	-	.	TF=HOXA4;class=Ho meo domain factors;sequence=GTC ATTAA
ZEB1|NM_0 01323647	TF BS	TF BS	97 1	97 7	0.9949 89704	-	.	TF=MEIS1;class=Hom eo domain factors;sequence=CTG ACAG
ZEB1|NM_0 01323641	TF BS	TF BS	13 69	13 75	0.9949 89704	-	.	TF=MEIS1;class=Hom eo domain factors;sequence=CTG ACAG
ZEB1|NM_0 01323638	TF BS	TF BS	17 70	17 76	0.9949 89704	-	.	TF=MEIS1;class=Hom eo domain factors;sequence=CTG ACAG
ZEB1|NM_0 01323644	TF BS	TF BS	13 69	13 75	0.9949 89704	-	.	TF=MEIS1;class=Hom eo domain

								factors;sequence=CTG ACAG
ZEB1|NM_0 01323643	TF BS	TF BS	17 70	17 76	0.9949 89704	-	.	TF=MEIS1;class=Hom eo domain factors;sequence=CTG ACAG
ZEB1|NM_0 01323674	TF BS	TF BS	97 1	97 7	0.9949 89704	-	.	TF=MEIS1;class=Hom eo domain factors;sequence=CTG ACAG
ZEB1|NM_0 01323658	TF BS	TF BS	41 9	42 5	0.9949 89704	-	.	TF=MEIS1;class=Hom eo domain factors;sequence=CTG ACAG
ZEB1|NM_0 01323657	TF BS	TF BS	41 9	42 5	0.9949 89704	-	.	TF=MEIS1;class=Hom eo domain factors;sequence=CTG ACAG
ZEB1|NM_0 01323650	TF BS	TF BS	97 1	97 7	0.9949 89704	-	.	TF=MEIS1;class=Hom eo domain factors;sequence=CTG ACAG
ZEB1|NM_0 01323648	TF BS	TF BS	97 1	97 7	0.9949 89704	-	.	TF=MEIS1;class=Hom eo domain factors;sequence=CTG ACAG
ZEB1|NM_0 01174095	TF BS	TF BS	97 1	97 7	0.9949 89704	-	.	TF=MEIS1;class=Hom eo domain factors;sequence=CTG ACAG
ZEB1|NM_0 01323646	TF BS	TF BS	13 69	13 75	0.9949 89704	-	.	TF=MEIS1;class=Hom eo domain factors;sequence=CTG ACAG
ZEB1|NM_0 01323645	TF BS	TF BS	13 69	13 75	0.9949 89704	-	.	TF=MEIS1;class=Hom eo domain factors;sequence=CTG ACAG
ZEB1|NM_0 01174093	TF BS	TF BS	97 1	97 7	0.9949 89704	-	.	TF=MEIS1;class=Hom eo domain factors;sequence=CTG ACAG
ZEB1|NM_0 01323656	TF BS	TF BS	41 9	42 5	0.9949 89704	-	.	TF=MEIS1;class=Hom eo domain

								factors;sequence=CTGACAG
ZEB1|NM_001323655	TFBS	TFBS	971	977	0.994989704	-	.	TF=MEIS1;class=Homeo domain factors;sequence=CTGACAG
ZEB1|NM_001323654	TFBS	TFBS	971	977	0.994989704	-	.	TF=MEIS1;class=Homeo domain factors;sequence=CTGACAG
ZEB1|NM_001323653	TFBS	TFBS	971	977	0.994989704	-	.	TF=MEIS1;class=Homeo domain factors;sequence=CTGACAG
ZEB1|NM_001323652	TFBS	TFBS	971	977	0.994989704	-	.	TF=MEIS1;class=Homeo domain factors;sequence=CTGACAG
ZEB1|NM_001323651	TFBS	TFBS	971	977	0.994989704	-	.	TF=MEIS1;class=Homeo domain factors;sequence=CTGACAG
ZEB1|NM_001323649	TFBS	TFBS	971	977	0.994989704	-	.	TF=MEIS1;class=Homeo domain factors;sequence=CTGACAG
ZEB1|NM_001323642	TFBS	TFBS	1369	1375	0.994989704	-	.	TF=MEIS1;class=Homeo domain factors;sequence=CTGACAG
ZEB1|NM_001174096	TFBS	TFBS	971	977	0.994989704	-	.	TF=MEIS1;class=Homeo domain factors;sequence=CTGACAG
ZEB1|NM_030751	TFBS	TFBS	971	977	0.994989704	-	.	TF=MEIS1;class=Homeo domain factors;sequence=CTGACAG
ZEB1|NM_001323661	TFBS	TFBS	1997	2007	0.994904316	-	.	TF=CDX1;class=Homeo domain factors;sequence=GGCAATAAAAC
ZEB1|NM_001323660	TFBS	TFBS	1997	2007	0.994904316	-	.	TF=CDX1;class=Homeo domain

								factors;sequence=GGC AATAAAAC
ZEB1|NM_0 01323659	TF BS	TF BS	19 97	20 07	0.9949 04316	-	.	TF=CDX1;class=Home o domain factors;sequence=GGC AATAAAAC
ZEB1|NM_0 01323673	TF BS	TF BS	16 67	16 74	0.9940 71307	-	.	TF=HOXC4;class=Ho meo domain factors;sequence=ATA ATTAA
ZEB1|NM_0 01323677	TF BS	TF BS	16 67	16 74	0.9940 71307	-	.	TF=HOXC4;class=Ho meo domain factors;sequence=ATA ATTAA
ZEB1|NM_0 01323676	TF BS	TF BS	16 67	16 74	0.9940 71307	-	.	TF=HOXC4;class=Ho meo domain factors;sequence=ATA ATTAA
ZEB1|NM_0 01323675	TF BS	TF BS	16 67	16 74	0.9940 71307	-	.	TF=HOXC4;class=Ho meo domain factors;sequence=ATA ATTAA
ZEB1|NM_0 01323672	TF BS	TF BS	16 67	16 74	0.9940 71307	-	.	TF=HOXC4;class=Ho meo domain factors;sequence=ATA ATTAA
ZEB1|NM_0 01323671	TF BS	TF BS	16 67	16 74	0.9940 71307	-	.	TF=HOXC4;class=Ho meo domain factors;sequence=ATA ATTAA
ZEB1|NM_0 01323647	TF BS	TF BS	19 41	19 46	0.9940 48003	-	.	TF=ZNF354C;class=C2 H2 zinc finger factors;sequence=CTC CAC
ZEB1|NM_0 01128128	TF BS	TF BS	20 9	21 4	0.9940 48003	-	.	TF=ZNF354C;class=C2 H2 zinc finger factors;sequence=CTC CAC
ZEB1|NM_0 01128128	TF BS	TF BS	44 5	45 0	0.9940 48003	-	.	TF=ZNF354C;class=C2 H2 zinc finger factors;sequence=CTC CAC
ZEB1|NM_0 01323641	TF BS	TF BS	15 3	15 8	0.9940 48003	+	.	TF=ZNF354C;class=C2 H2 zinc finger

								factors;sequence=CTC CAC
ZEB1|NM_0 01323638	TF BS	TF BS	55 4	55 9	0.9940 48003	+	.	TF=ZNF354C;class=C2 H2 zinc finger factors;sequence=CTC CAC
ZEB1|NM_0 01323644	TF BS	TF BS	15 3	15 8	0.9940 48003	+	.	TF=ZNF354C;class=C2 H2 zinc finger factors;sequence=CTC CAC
ZEB1|NM_0 01323643	TF BS	TF BS	55 4	55 9	0.9940 48003	+	.	TF=ZNF354C;class=C2 H2 zinc finger factors;sequence=CTC CAC
ZEB1|NM_0 01323674	TF BS	TF BS	19 41	19 46	0.9940 48003	-	.	TF=ZNF354C;class=C2 H2 zinc finger factors;sequence=CTC CAC
ZEB1|NM_0 01323673	TF BS	TF BS	17 43	17 48	0.9940 48003	-	.	TF=ZNF354C;class=C2 H2 zinc finger factors;sequence=CTC CAC
ZEB1|NM_0 01323673	TF BS	TF BS	17 71	17 76	0.9940 48003	-	.	TF=ZNF354C;class=C2 H2 zinc finger factors;sequence=CTC CAC
ZEB1|NM_0 01323658	TF BS	TF BS	13 89	13 94	0.9940 48003	-	.	TF=ZNF354C;class=C2 H2 zinc finger factors;sequence=CTC CAC
ZEB1|NM_0 01323658	TF BS	TF BS	16 42	16 47	0.9940 48003	-	.	TF=ZNF354C;class=C2 H2 zinc finger factors;sequence=CTC CAC
ZEB1|NM_0 01323658	TF BS	TF BS	18 78	18 83	0.9940 48003	-	.	TF=ZNF354C;class=C2 H2 zinc finger factors;sequence=CTC CAC
ZEB1|NM_0 01323657	TF BS	TF BS	13 89	13 94	0.9940 48003	-	.	TF=ZNF354C;class=C2 H2 zinc finger factors;sequence=CTC CAC
ZEB1|NM_0 01323657	TF BS	TF BS	16 42	16 47	0.9940 48003	-	.	TF=ZNF354C;class=C2 H2 zinc finger

								factors;sequence=CTC CAC
ZEB1|NM_0 01323657	TF BS	TF BS	18 78	18 83	0.9940 48003	-	.	TF=ZNF354C;class=C2 H2 zinc finger factors;sequence=CTC CAC
ZEB1|NM_0 01323650	TF BS	TF BS	19 41	19 46	0.9940 48003	-	.	TF=ZNF354C;class=C2 H2 zinc finger factors;sequence=CTC CAC
ZEB1|NM_0 01323648	TF BS	TF BS	19 41	19 46	0.9940 48003	-	.	TF=ZNF354C;class=C2 H2 zinc finger factors;sequence=CTC CAC
ZEB1|NM_0 01174095	TF BS	TF BS	19 41	19 46	0.9940 48003	-	.	TF=ZNF354C;class=C2 H2 zinc finger factors;sequence=CTC CAC
ZEB1|NM_0 01323646	TF BS	TF BS	15 3	15 8	0.9940 48003	+	.	TF=ZNF354C;class=C2 H2 zinc finger factors;sequence=CTC CAC
ZEB1|NM_0 01323645	TF BS	TF BS	15 3	15 8	0.9940 48003	+	.	TF=ZNF354C;class=C2 H2 zinc finger factors;sequence=CTC CAC
ZEB1|NM_0 01174093	TF BS	TF BS	19 41	19 46	0.9940 48003	-	.	TF=ZNF354C;class=C2 H2 zinc finger factors;sequence=CTC CAC
ZEB1|NM_0 01323656	TF BS	TF BS	13 89	13 94	0.9940 48003	-	.	TF=ZNF354C;class=C2 H2 zinc finger factors;sequence=CTC CAC
ZEB1|NM_0 01323656	TF BS	TF BS	16 42	16 47	0.9940 48003	-	.	TF=ZNF354C;class=C2 H2 zinc finger factors;sequence=CTC CAC
ZEB1|NM_0 01323656	TF BS	TF BS	18 78	18 83	0.9940 48003	-	.	TF=ZNF354C;class=C2 H2 zinc finger factors;sequence=CTC CAC
ZEB1|NM_0 01323655	TF BS	TF BS	19 41	19 46	0.9940 48003	-	.	TF=ZNF354C;class=C2 H2 zinc finger

<table>
<tr><td></td><td></td><td></td><td></td><td></td><td></td><td></td><td></td><td>factors;sequence=CTCCAC</td></tr>
<tr><td>ZEB1|NM_001323654</td><td>TFBS</td><td>TFBS</td><td>1941</td><td>1946</td><td>0.994048003</td><td>-</td><td>.</td><td>TF=ZNF354C;class=C2H2 zinc finger factors;sequence=CTCCAC</td></tr>
<tr><td>ZEB1|NM_001323653</td><td>TFBS</td><td>TFBS</td><td>1941</td><td>1946</td><td>0.994048003</td><td>-</td><td>.</td><td>TF=ZNF354C;class=C2H2 zinc finger factors;sequence=CTCCAC</td></tr>
<tr><td>ZEB1|NM_001323652</td><td>TFBS</td><td>TFBS</td><td>1941</td><td>1946</td><td>0.994048003</td><td>-</td><td>.</td><td>TF=ZNF354C;class=C2H2 zinc finger factors;sequence=CTCCAC</td></tr>
<tr><td>ZEB1|NM_001323651</td><td>TFBS</td><td>TFBS</td><td>1941</td><td>1946</td><td>0.994048003</td><td>-</td><td>.</td><td>TF=ZNF354C;class=C2H2 zinc finger factors;sequence=CTCCAC</td></tr>
<tr><td>ZEB1|NM_001323678</td><td>TFBS</td><td>TFBS</td><td>209</td><td>214</td><td>0.994048003</td><td>-</td><td>.</td><td>TF=ZNF354C;class=C2H2 zinc finger factors;sequence=CTCCAC</td></tr>
<tr><td>ZEB1|NM_001323678</td><td>TFBS</td><td>TFBS</td><td>445</td><td>450</td><td>0.994048003</td><td>-</td><td>.</td><td>TF=ZNF354C;class=C2H2 zinc finger factors;sequence=CTCCAC</td></tr>
<tr><td>ZEB1|NM_001323677</td><td>TFBS</td><td>TFBS</td><td>1743</td><td>1748</td><td>0.994048003</td><td>-</td><td>.</td><td>TF=ZNF354C;class=C2H2 zinc finger factors;sequence=CTCCAC</td></tr>
<tr><td>ZEB1|NM_001323677</td><td>TFBS</td><td>TFBS</td><td>1771</td><td>1776</td><td>0.994048003</td><td>-</td><td>.</td><td>TF=ZNF354C;class=C2H2 zinc finger factors;sequence=CTCCAC</td></tr>
<tr><td>ZEB1|NM_001323676</td><td>TFBS</td><td>TFBS</td><td>1743</td><td>1748</td><td>0.994048003</td><td>-</td><td>.</td><td>TF=ZNF354C;class=C2H2 zinc finger factors;sequence=CTCCAC</td></tr>
<tr><td>ZEB1|NM_001323676</td><td>TFBS</td><td>TFBS</td><td>1771</td><td>1776</td><td>0.994048003</td><td>-</td><td>.</td><td>TF=ZNF354C;class=C2H2 zinc finger factors;sequence=CTCCAC</td></tr>
<tr><td>ZEB1|NM_001323649</td><td>TFBS</td><td>TFBS</td><td>1941</td><td>1946</td><td>0.994048003</td><td>-</td><td>.</td><td>TF=ZNF354C;class=C2H2 zinc finger</td></tr>
</table>

								factors;sequence=CTC CAC
ZEB1|NM_0 01323675	TF BS	TF BS	17 43	17 48	0.9940 48003	-	.	TF=ZNF354C;class=C2 H2 zinc finger factors;sequence=CTC CAC
ZEB1|NM_0 01323675	TF BS	TF BS	17 71	17 76	0.9940 48003	-	.	TF=ZNF354C;class=C2 H2 zinc finger factors;sequence=CTC CAC
ZEB1|NM_0 01323672	TF BS	TF BS	17 43	17 48	0.9940 48003	-	.	TF=ZNF354C;class=C2 H2 zinc finger factors;sequence=CTC CAC
ZEB1|NM_0 01323672	TF BS	TF BS	17 71	17 76	0.9940 48003	-	.	TF=ZNF354C;class=C2 H2 zinc finger factors;sequence=CTC CAC
ZEB1|NM_0 01323671	TF BS	TF BS	17 43	17 48	0.9940 48003	-	.	TF=ZNF354C;class=C2 H2 zinc finger factors;sequence=CTC CAC
ZEB1|NM_0 01323671	TF BS	TF BS	17 71	17 76	0.9940 48003	-	.	TF=ZNF354C;class=C2 H2 zinc finger factors;sequence=CTC CAC
ZEB1|NM_0 01323666	TF BS	TF BS	20 9	21 4	0.9940 48003	-	.	TF=ZNF354C;class=C2 H2 zinc finger factors;sequence=CTC CAC
ZEB1|NM_0 01323666	TF BS	TF BS	44 5	45 0	0.9940 48003	-	.	TF=ZNF354C;class=C2 H2 zinc finger factors;sequence=CTC CAC
ZEB1|NM_0 01323642	TF BS	TF BS	15 3	15 8	0.9940 48003	+	.	TF=ZNF354C;class=C2 H2 zinc finger factors;sequence=CTC CAC
ZEB1|NM_0 01174096	TF BS	TF BS	19 41	19 46	0.9940 48003	-	.	TF=ZNF354C;class=C2 H2 zinc finger factors;sequence=CTC CAC
ZEB1|NM_0 30751	TF BS	TF BS	19 41	19 46	0.9940 48003	-	.	TF=ZNF354C;class=C2 H2 zinc finger

								factors;sequence=CTC CAC
ZEB1|NM_0 01323665	TF BS	TF BS	20 9	21 4	0.9940 48003	-	.	TF=ZNF354C;class=C2 H2 zinc finger factors;sequence=CTC CAC
ZEB1|NM_0 01323665	TF BS	TF BS	44 5	45 0	0.9940 48003	-	.	TF=ZNF354C;class=C2 H2 zinc finger factors;sequence=CTC CAC
ZEB1|NM_0 01323664	TF BS	TF BS	20 9	21 4	0.9940 48003	-	.	TF=ZNF354C;class=C2 H2 zinc finger factors;sequence=CTC CAC
ZEB1|NM_0 01323664	TF BS	TF BS	44 5	45 0	0.9940 48003	-	.	TF=ZNF354C;class=C2 H2 zinc finger factors;sequence=CTC CAC
ZEB1|NM_0 01323663	TF BS	TF BS	20 9	21 4	0.9940 48003	-	.	TF=ZNF354C;class=C2 H2 zinc finger factors;sequence=CTC CAC
ZEB1|NM_0 01323663	TF BS	TF BS	44 5	45 0	0.9940 48003	-	.	TF=ZNF354C;class=C2 H2 zinc finger factors;sequence=CTC CAC
ZEB1|NM_0 01323662	TF BS	TF BS	20 9	21 4	0.9940 48003	-	.	TF=ZNF354C;class=C2 H2 zinc finger factors;sequence=CTC CAC
ZEB1|NM_0 01323662	TF BS	TF BS	44 5	45 0	0.9940 48003	-	.	TF=ZNF354C;class=C2 H2 zinc finger factors;sequence=CTC CAC
ZEB1|NM_0 01323661	TF BS	TF BS	60 3	60 8	0.9940 48003	-	.	TF=ZNF354C;class=C2 H2 zinc finger factors;sequence=CTC CAC
ZEB1|NM_0 01323661	TF BS	TF BS	85 6	86 1	0.9940 48003	-	.	TF=ZNF354C;class=C2 H2 zinc finger factors;sequence=CTC CAC
ZEB1|NM_0 01323661	TF BS	TF BS	10 92	10 97	0.9940 48003	-	.	TF=ZNF354C;class=C2 H2 zinc finger

								factors;sequence=CTCCAC
ZEB1|NM_001323660	TFBS	TFBS	603	608	0.994048003	-	.	TF=ZNF354C;class=C2H2 zinc finger factors;sequence=CTCCAC
ZEB1|NM_001323660	TFBS	TFBS	856	861	0.994048003	-	.	TF=ZNF354C;class=C2H2 zinc finger factors;sequence=CTCCAC
ZEB1|NM_001323660	TFBS	TFBS	1092	1097	0.994048003	-	.	TF=ZNF354C;class=C2H2 zinc finger factors;sequence=CTCCAC
ZEB1|NM_001323659	TFBS	TFBS	603	608	0.994048003	-	.	TF=ZNF354C;class=C2H2 zinc finger factors;sequence=CTCCAC
ZEB1|NM_001323659	TFBS	TFBS	856	861	0.994048003	-	.	TF=ZNF354C;class=C2H2 zinc finger factors;sequence=CTCCAC
ZEB1|NM_001323659	TFBS	TFBS	1092	1097	0.994048003	-	.	TF=ZNF354C;class=C2H2 zinc finger factors;sequence=CTCCAC
ZEB1|NM_001174094	TFBS	TFBS	209	214	0.994048003	-	.	TF=ZNF354C;class=C2H2 zinc finger factors;sequence=CTCCAC
ZEB1|NM_001174094	TFBS	TFBS	445	450	0.994048003	-	.	TF=ZNF354C;class=C2H2 zinc finger factors;sequence=CTCCAC
ZEB1|NM_001323661	TFBS	TFBS	1997	2007	0.993947523	-	.	TF=HOXD13;class=Homeo domain factors;sequence=GGCAATAAAAC
ZEB1|NM_001323660	TFBS	TFBS	1997	2007	0.993947523	-	.	TF=HOXD13;class=Homeo domain factors;sequence=GGCAATAAAAC
ZEB1|NM_001323659	TFBS	TFBS	1997	2007	0.993947523	-	.	TF=HOXD13;class=Homeo domain

								factors;sequence=GGCAATAAAAC
ZEB1|NM_001128128	TFBS	TFBS	296	308	0.993509177	+	.	TF=OVOL2;class=C2H2 zinc finger factors;sequence=GAACCGTTATGTC
ZEB1|NM_001323658	TFBS	TFBS	1729	1741	0.993509177	+	.	TF=OVOL2;class=C2H2 zinc finger factors;sequence=GAACCGTTATGTC
ZEB1|NM_001323657	TFBS	TFBS	1729	1741	0.993509177	+	.	TF=OVOL2;class=C2H2 zinc finger factors;sequence=GAACCGTTATGTC
ZEB1|NM_001323656	TFBS	TFBS	1729	1741	0.993509177	+	.	TF=OVOL2;class=C2H2 zinc finger factors;sequence=GAACCGTTATGTC
ZEB1|NM_001323678	TFBS	TFBS	296	308	0.993509177	+	.	TF=OVOL2;class=C2H2 zinc finger factors;sequence=GAACCGTTATGTC
ZEB1|NM_001323666	TFBS	TFBS	296	308	0.993509177	+	.	TF=OVOL2;class=C2H2 zinc finger factors;sequence=GAACCGTTATGTC
ZEB1|NM_001323665	TFBS	TFBS	296	308	0.993509177	+	.	TF=OVOL2;class=C2H2 zinc finger factors;sequence=GAACCGTTATGTC
ZEB1|NM_001323664	TFBS	TFBS	296	308	0.993509177	+	.	TF=OVOL2;class=C2H2 zinc finger factors;sequence=GAACCGTTATGTC
ZEB1|NM_001323663	TFBS	TFBS	296	308	0.993509177	+	.	TF=OVOL2;class=C2H2 zinc finger factors;sequence=GAACCGTTATGTC
ZEB1|NM_001323662	TFBS	TFBS	296	308	0.993509177	+	.	TF=OVOL2;class=C2H2 zinc finger factors;sequence=GAACCGTTATGTC
ZEB1|NM_001323661	TFBS	TFBS	943	955	0.993509177	+	.	TF=OVOL2;class=C2H2 zinc finger

								factors;sequence=GAA CCGTTATGTC
ZEB1|NM_0 01323660	TF BS	TF BS	94 3	95 5	0.9935 09177	+	.	TF=OVOL2;class=C2H 2 zinc finger factors;sequence=GAA CCGTTATGTC
ZEB1|NM_0 01323659	TF BS	TF BS	94 3	95 5	0.9935 09177	+	.	TF=OVOL2;class=C2H 2 zinc finger factors;sequence=GAA CCGTTATGTC
ZEB1|NM_0 01174094	TF BS	TF BS	29 6	30 8	0.9935 09177	+	.	TF=OVOL2;class=C2H 2 zinc finger factors;sequence=GAA CCGTTATGTC
ZEB1|NM_0 01323673	TF BS	TF BS	19 59	19 68	0.9929 58964	-	.	TF=MXI1;class=Basic helix-loop-helix factors (bHLH);sequence=CAC ACATGTC
ZEB1|NM_0 01323677	TF BS	TF BS	19 59	19 68	0.9929 58964	-	.	TF=MXI1;class=Basic helix-loop-helix factors (bHLH);sequence=CAC ACATGTC
ZEB1|NM_0 01323676	TF BS	TF BS	19 59	19 68	0.9929 58964	-	.	TF=MXI1;class=Basic helix-loop-helix factors (bHLH);sequence=CAC ACATGTC
ZEB1|NM_0 01323675	TF BS	TF BS	19 59	19 68	0.9929 58964	-	.	TF=MXI1;class=Basic helix-loop-helix factors (bHLH);sequence=CAC ACATGTC
ZEB1|NM_0 01323672	TF BS	TF BS	19 59	19 68	0.9929 58964	-	.	TF=MXI1;class=Basic helix-loop-helix factors (bHLH);sequence=CAC ACATGTC
ZEB1|NM_0 01323671	TF BS	TF BS	19 59	19 68	0.9929 58964	-	.	TF=MXI1;class=Basic helix-loop-helix factors (bHLH);sequence=CAC ACATGTC
ZEB1|NM_0 01323638	TF BS	TF BS	39 5	40 2	0.9928 97745	+	.	TF=HOXB6;class=Ho meo domain factors;sequence=TTA ATGAC
ZEB1|NM_0 01323643	TF BS	TF BS	39 5	40 2	0.9928 97745	+	.	TF=HOXB6;class=Ho meo domain

								factors;sequence=TTA ATGAC
ZEB1|NM_0 01323673	TF BS	TF BS	16 67	16 74	0.9928 87021	-	.	TF=HOXB4;class=Ho meo domain factors;sequence=ATA ATTAA
ZEB1|NM_0 01323677	TF BS	TF BS	16 67	16 74	0.9928 87021	-	.	TF=HOXB4;class=Ho meo domain factors;sequence=ATA ATTAA
ZEB1|NM_0 01323676	TF BS	TF BS	16 67	16 74	0.9928 87021	-	.	TF=HOXB4;class=Ho meo domain factors;sequence=ATA ATTAA
ZEB1|NM_0 01323675	TF BS	TF BS	16 67	16 74	0.9928 87021	-	.	TF=HOXB4;class=Ho meo domain factors;sequence=ATA ATTAA
ZEB1|NM_0 01323672	TF BS	TF BS	16 67	16 74	0.9928 87021	-	.	TF=HOXB4;class=Ho meo domain factors;sequence=ATA ATTAA
ZEB1|NM_0 01323671	TF BS	TF BS	16 67	16 74	0.9928 87021	-	.	TF=HOXB4;class=Ho meo domain factors;sequence=ATA ATTAA
ZEB1|NM_0 01128128	TF BS	TF BS	14 49	14 58	0.9925 53464	-	.	TF=MXI1;class=Basic helix-loop-helix factors (bHLH);sequence=ACC ACATGTT
ZEB1|NM_0 01323673	TF BS	TF BS	99 0	99 9	0.9925 53464	-	.	TF=MXI1;class=Basic helix-loop-helix factors (bHLH);sequence=ACC ACATGTT
ZEB1|NM_0 01323678	TF BS	TF BS	14 49	14 58	0.9925 53464	-	.	TF=MXI1;class=Basic helix-loop-helix factors (bHLH);sequence=ACC ACATGTT
ZEB1|NM_0 01323677	TF BS	TF BS	99 0	99 9	0.9925 53464	-	.	TF=MXI1;class=Basic helix-loop-helix factors (bHLH);sequence=ACC ACATGTT
ZEB1|NM_0 01323676	TF BS	TF BS	99 0	99 9	0.9925 53464	-	.	TF=MXI1;class=Basic helix-loop-helix factors

								(bHLH);sequence=ACCACATGTT
ZEB1|NM_001323675	TFBS	TFBS	990	999	0.992553464	-	.	TF=MXI1;class=Basic helix-loop-helix factors (bHLH);sequence=ACCACATGTT
ZEB1|NM_001323672	TFBS	TFBS	990	999	0.992553464	-	.	TF=MXI1;class=Basic helix-loop-helix factors (bHLH);sequence=ACCACATGTT
ZEB1|NM_001323671	TFBS	TFBS	990	999	0.992553464	-	.	TF=MXI1;class=Basic helix-loop-helix factors (bHLH);sequence=ACCACATGTT
ZEB1|NM_001323666	TFBS	TFBS	1449	1458	0.992553464	-	.	TF=MXI1;class=Basic helix-loop-helix factors (bHLH);sequence=ACCACATGTT
ZEB1|NM_001323665	TFBS	TFBS	1449	1458	0.992553464	-	.	TF=MXI1;class=Basic helix-loop-helix factors (bHLH);sequence=ACCACATGTT
ZEB1|NM_001323664	TFBS	TFBS	1449	1458	0.992553464	-	.	TF=MXI1;class=Basic helix-loop-helix factors (bHLH);sequence=ACCACATGTT
ZEB1|NM_001323663	TFBS	TFBS	1449	1458	0.992553464	-	.	TF=MXI1;class=Basic helix-loop-helix factors (bHLH);sequence=ACCACATGTT
ZEB1|NM_001323662	TFBS	TFBS	1449	1458	0.992553464	-	.	TF=MXI1;class=Basic helix-loop-helix factors (bHLH);sequence=ACCACATGTT
ZEB1|NM_001174094	TFBS	TFBS	1449	1458	0.992553464	-	.	TF=MXI1;class=Basic helix-loop-helix factors (bHLH);sequence=ACCACATGTT
ZEB1|NM_001323647	TFBS	TFBS	85	93	0.992368199	+	.	TF=NR2C1;class=Nuclear receptors with C4 zinc fingers;sequence=TGGGGTCAA
ZEB1|NM_0	TF	TF	48	49	0.9923	+	.	TF=NR2C1;class=Nucl

01323641	BS	BS	3	1	68199			ear receptors with C4 zinc fingers;sequence=TGGGGTCAA
ZEB1|NM_001323638	TF BS	TF BS	884	892	0.992368199	+	.	TF=NR2C1;class=Nuclear receptors with C4 zinc fingers;sequence=TGGGGTCAA
ZEB1|NM_001323644	TF BS	TF BS	483	491	0.992368199	+	.	TF=NR2C1;class=Nuclear receptors with C4 zinc fingers;sequence=TGGGGTCAA
ZEB1|NM_001323643	TF BS	TF BS	884	892	0.992368199	+	.	TF=NR2C1;class=Nuclear receptors with C4 zinc fingers;sequence=TGGGGTCAA
ZEB1|NM_001323674	TF BS	TF BS	85	93	0.992368199	+	.	TF=NR2C1;class=Nuclear receptors with C4 zinc fingers;sequence=TGGGGTCAA
ZEB1|NM_001323650	TF BS	TF BS	85	93	0.992368199	+	.	TF=NR2C1;class=Nuclear receptors with C4 zinc fingers;sequence=TGGGGTCAA
ZEB1|NM_001323648	TF BS	TF BS	85	93	0.992368199	+	.	TF=NR2C1;class=Nuclear receptors with C4 zinc fingers;sequence=TGGGGTCAA
ZEB1|NM_001174095	TF BS	TF BS	85	93	0.992368199	+	.	TF=NR2C1;class=Nuclear receptors with C4 zinc fingers;sequence=TGGGGTCAA
ZEB1|NM_001323646	TF BS	TF BS	483	491	0.992368199	+	.	TF=NR2C1;class=Nuclear receptors with C4 zinc fingers;sequence=TGGGGTCAA

ZEB1|NM_001323645	TFBS	TFBS	483	491	0.992368199	+	.	TF=NR2C1;class=Nuclear receptors with C4 zinc fingers;sequence=TGGGGTCAA
ZEB1|NM_001174093	TFBS	TFBS	85	93	0.992368199	+	.	TF=NR2C1;class=Nuclear receptors with C4 zinc fingers;sequence=TGGGGTCAA
ZEB1|NM_001323655	TFBS	TFBS	85	93	0.992368199	+	.	TF=NR2C1;class=Nuclear receptors with C4 zinc fingers;sequence=TGGGGTCAA
ZEB1|NM_001323654	TFBS	TFBS	85	93	0.992368199	+	.	TF=NR2C1;class=Nuclear receptors with C4 zinc fingers;sequence=TGGGGTCAA
ZEB1|NM_001323653	TFBS	TFBS	85	93	0.992368199	+	.	TF=NR2C1;class=Nuclear receptors with C4 zinc fingers;sequence=TGGGGTCAA
ZEB1|NM_001323652	TFBS	TFBS	85	93	0.992368199	+	.	TF=NR2C1;class=Nuclear receptors with C4 zinc fingers;sequence=TGGGGTCAA
ZEB1|NM_001323651	TFBS	TFBS	85	93	0.992368199	+	.	TF=NR2C1;class=Nuclear receptors with C4 zinc fingers;sequence=TGGGGTCAA
ZEB1|NM_001323649	TFBS	TFBS	85	93	0.992368199	+	.	TF=NR2C1;class=Nuclear receptors with C4 zinc fingers;sequence=TGGGGTCAA
ZEB1|NM_001323642	TFBS	TFBS	483	491	0.992368199	+	.	TF=NR2C1;class=Nuclear receptors with C4 zinc fingers;sequence=TGG

								GGTCAA
ZEB1|NM_001174096	TFBS	TFBS	85	93	0.992368199	+	.	TF=NR2C1;class=Nuclear receptors with C4 zinc fingers;sequence=TGGGGTCAA
ZEB1|NM_030751	TFBS	TFBS	85	93	0.992368199	+	.	TF=NR2C1;class=Nuclear receptors with C4 zinc fingers;sequence=TGGGGTCAA
ZEB1|NM_001128128	TFBS	TFBS	29	36	0.992088812	-	.	TF=RHOXF1;class=Homeo domain factors;sequence=ATGATCCT
ZEB1|NM_001323658	TFBS	TFBS	1462	1469	0.992088812	-	.	TF=RHOXF1;class=Homeo domain factors;sequence=ATGATCCT
ZEB1|NM_001323657	TFBS	TFBS	1462	1469	0.992088812	-	.	TF=RHOXF1;class=Homeo domain factors;sequence=ATGATCCT
ZEB1|NM_001323656	TFBS	TFBS	1462	1469	0.992088812	-	.	TF=RHOXF1;class=Homeo domain factors;sequence=ATGATCCT
ZEB1|NM_001323678	TFBS	TFBS	29	36	0.992088812	-	.	TF=RHOXF1;class=Homeo domain factors;sequence=ATGATCCT
ZEB1|NM_001323666	TFBS	TFBS	29	36	0.992088812	-	.	TF=RHOXF1;class=Homeo domain factors;sequence=ATGATCCT
ZEB1|NM_001323665	TFBS	TFBS	29	36	0.992088812	-	.	TF=RHOXF1;class=Homeo domain factors;sequence=ATGATCCT
ZEB1|NM_001323664	TFBS	TFBS	29	36	0.992088812	-	.	TF=RHOXF1;class=Homeo domain factors;sequence=ATGATCCT
ZEB1|NM_0	TF	TF	29	36	0.9920	-	.	TF=RHOXF1;class=Ho

01323663	BS	BS			88812			meo domain factors;sequence=ATGATCCT
ZEB1|NM_001323662	TFBS	TFBS	29	36	0.992088812	-	.	TF=RHOXF1;class=Homeo domain factors;sequence=ATGATCCT
ZEB1|NM_001323661	TFBS	TFBS	676	683	0.992088812	-	.	TF=RHOXF1;class=Homeo domain factors;sequence=ATGATCCT
ZEB1|NM_001323660	TFBS	TFBS	676	683	0.992088812	-	.	TF=RHOXF1;class=Homeo domain factors;sequence=ATGATCCT
ZEB1|NM_001323659	TFBS	TFBS	676	683	0.992088812	-	.	TF=RHOXF1;class=Homeo domain factors;sequence=ATGATCCT
ZEB1|NM_001174094	TFBS	TFBS	29	36	0.992088812	-	.	TF=RHOXF1;class=Homeo domain factors;sequence=ATGATCCT
ZEB1|NM_001128128	TFBS	TFBS	1997	2008	0.992060007	-	.	TF=CDX2;class=Homeo domain factors;sequence=AGGCAATAAAAA
ZEB1|NM_001323678	TFBS	TFBS	1997	2008	0.992060007	-	.	TF=CDX2;class=Homeo domain factors;sequence=AGGCAATAAAAA
ZEB1|NM_001323666	TFBS	TFBS	1997	2008	0.992060007	-	.	TF=CDX2;class=Homeo domain factors;sequence=AGGCAATAAAAA
ZEB1|NM_001323665	TFBS	TFBS	1997	2008	0.992060007	-	.	TF=CDX2;class=Homeo domain factors;sequence=AGGCAATAAAAA
ZEB1|NM_001323664	TFBS	TFBS	1997	2008	0.992060007	-	.	TF=CDX2;class=Homeo domain factors;sequence=AGGCAATAAAAA
ZEB1|NM_0	TF	TF	19	20	0.9920	-	.	TF=CDX2;class=Home

01323663	BS	BS	97	08	60007			o domain factors;sequence=AGGCAATAAAAA
ZEB1|NM_001323662	TFBS	TFBS	1997	2008	0.992060007	-	.	TF=CDX2;class=Homeo domain factors;sequence=AGGCAATAAAAA
ZEB1|NM_001174094	TFBS	TFBS	1997	2008	0.992060007	-	.	TF=CDX2;class=Homeo domain factors;sequence=AGGCAATAAAAA
ZEB1|NM_001323641	TFBS	TFBS	86	93	0.991957077	+	.	TF=NR2C2(var.2);class=Nuclear receptors with C4 zinc fingers;sequence=AGGGTCAG
ZEB1|NM_001323638	TFBS	TFBS	487	494	0.991957077	+	.	TF=NR2C2(var.2);class=Nuclear receptors with C4 zinc fingers;sequence=AGGGTCAG
ZEB1|NM_001323644	TFBS	TFBS	86	93	0.991957077	+	.	TF=NR2C2(var.2);class=Nuclear receptors with C4 zinc fingers;sequence=AGGGTCAG
ZEB1|NM_001323643	TFBS	TFBS	487	494	0.991957077	+	.	TF=NR2C2(var.2);class=Nuclear receptors with C4 zinc fingers;sequence=AGGGTCAG
ZEB1|NM_001323646	TFBS	TFBS	86	93	0.991957077	+	.	TF=NR2C2(var.2);class=Nuclear receptors with C4 zinc fingers;sequence=AGGGTCAG
ZEB1|NM_001323645	TFBS	TFBS	86	93	0.991957077	+	.	TF=NR2C2(var.2);class=Nuclear receptors with C4 zinc fingers;sequence=AGGGTCAG
ZEB1|NM_001323642	TFBS	TFBS	86	93	0.991957077	+	.	TF=NR2C2(var.2);class=Nuclear receptors with C4 zinc

								fingers;sequence=AGGGTCAG
ZEB1|NM_001323647	TFBS	TFBS	1997	2007	0.99187081	-	.	TF=CDX4;class=Homeo domain factors;sequence=GGCAATAAAAT
ZEB1|NM_001323674	TFBS	TFBS	1997	2007	0.99187081	-	.	TF=CDX4;class=Homeo domain factors;sequence=GGCAATAAAAT
ZEB1|NM_001323650	TFBS	TFBS	1997	2007	0.99187081	-	.	TF=CDX4;class=Homeo domain factors;sequence=GGCAATAAAAT
ZEB1|NM_001323648	TFBS	TFBS	1997	2007	0.99187081	-	.	TF=CDX4;class=Homeo domain factors;sequence=GGCAATAAAAT
ZEB1|NM_001174095	TFBS	TFBS	1997	2007	0.99187081	-	.	TF=CDX4;class=Homeo domain factors;sequence=GGCAATAAAAT
ZEB1|NM_001174093	TFBS	TFBS	1997	2007	0.99187081	-	.	TF=CDX4;class=Homeo domain factors;sequence=GGCAATAAAAT
ZEB1|NM_001323655	TFBS	TFBS	1997	2007	0.99187081	-	.	TF=CDX4;class=Homeo domain factors;sequence=GGCAATAAAAT
ZEB1|NM_001323654	TFBS	TFBS	1997	2007	0.99187081	-	.	TF=CDX4;class=Homeo domain factors;sequence=GGCAATAAAAT
ZEB1|NM_001323653	TFBS	TFBS	1997	2007	0.99187081	-	.	TF=CDX4;class=Homeo domain factors;sequence=GGCAATAAAAT
ZEB1|NM_001323652	TFBS	TFBS	1997	2007	0.99187081	-	.	TF=CDX4;class=Homeo domain factors;sequence=GGCAATAAAAT
ZEB1|NM_001323651	TFBS	TFBS	1997	2007	0.99187081	-	.	TF=CDX4;class=Homeo domain

								factors;sequence=GGC AATAAAAT
ZEB1|NM_0 01323649	TF BS	TF BS	19 97	20 07	0.9918 7081	-	.	TF=CDX4;class=Home o domain factors;sequence=GGC AATAAAAT
ZEB1|NM_0 01174096	TF BS	TF BS	19 97	20 07	0.9918 7081	-	.	TF=CDX4;class=Home o domain factors;sequence=GGC AATAAAAT
ZEB1|NM_0 30751	TF BS	TF BS	19 97	20 07	0.9918 7081	-	.	TF=CDX4;class=Home o domain factors;sequence=GGC AATAAAAT
ZEB1|NM_0 01323647	TF BS	TF BS	26 6	27 3	0.9915 28411	+	.	TF=HOXA4;class=Ho meo domain factors;sequence=ATC ATTAA
ZEB1|NM_0 01323641	TF BS	TF BS	66 4	67 1	0.9915 28411	+	.	TF=HOXA4;class=Ho meo domain factors;sequence=ATC ATTAA
ZEB1|NM_0 01323638	TF BS	TF BS	10 65	10 72	0.9915 28411	+	.	TF=HOXA4;class=Ho meo domain factors;sequence=ATC ATTAA
ZEB1|NM_0 01323644	TF BS	TF BS	66 4	67 1	0.9915 28411	+	.	TF=HOXA4;class=Ho meo domain factors;sequence=ATC ATTAA
ZEB1|NM_0 01323643	TF BS	TF BS	10 65	10 72	0.9915 28411	+	.	TF=HOXA4;class=Ho meo domain factors;sequence=ATC ATTAA
ZEB1|NM_0 01323674	TF BS	TF BS	26 6	27 3	0.9915 28411	+	.	TF=HOXA4;class=Ho meo domain factors;sequence=ATC ATTAA
ZEB1|NM_0 01323673	TF BS	TF BS	16 40	16 47	0.9915 28411	+	.	TF=HOXA4;class=Ho meo domain factors;sequence=ATC ATTAA
ZEB1|NM_0 01323650	TF BS	TF BS	26 6	27 3	0.9915 28411	+	.	TF=HOXA4;class=Ho meo domain

								factors;sequence=ATC ATTAA
ZEB1|NM_0 01323648	TF BS	TF BS	26 6	27 3	0.9915 28411	+	.	TF=HOXA4;class=Ho meo domain factors;sequence=ATC ATTAA
ZEB1|NM_0 01174095	TF BS	TF BS	26 6	27 3	0.9915 28411	+	.	TF=HOXA4;class=Ho meo domain factors;sequence=ATC ATTAA
ZEB1|NM_0 01323646	TF BS	TF BS	66 4	67 1	0.9915 28411	+	.	TF=HOXA4;class=Ho meo domain factors;sequence=ATC ATTAA
ZEB1|NM_0 01323645	TF BS	TF BS	66 4	67 1	0.9915 28411	+	.	TF=HOXA4;class=Ho meo domain factors;sequence=ATC ATTAA
ZEB1|NM_0 01174093	TF BS	TF BS	26 6	27 3	0.9915 28411	+	.	TF=HOXA4;class=Ho meo domain factors;sequence=ATC ATTAA
ZEB1|NM_0 01323655	TF BS	TF BS	26 6	27 3	0.9915 28411	+	.	TF=HOXA4;class=Ho meo domain factors;sequence=ATC ATTAA
ZEB1|NM_0 01323654	TF BS	TF BS	26 6	27 3	0.9915 28411	+	.	TF=HOXA4;class=Ho meo domain factors;sequence=ATC ATTAA
ZEB1|NM_0 01323653	TF BS	TF BS	26 6	27 3	0.9915 28411	+	.	TF=HOXA4;class=Ho meo domain factors;sequence=ATC ATTAA
ZEB1|NM_0 01323652	TF BS	TF BS	26 6	27 3	0.9915 28411	+	.	TF=HOXA4;class=Ho meo domain factors;sequence=ATC ATTAA
ZEB1|NM_0 01323651	TF BS	TF BS	26 6	27 3	0.9915 28411	+	.	TF=HOXA4;class=Ho meo domain factors;sequence=ATC ATTAA
ZEB1|NM_0 01323677	TF BS	TF BS	16 40	16 47	0.9915 28411	+	.	TF=HOXA4;class=Ho meo domain

								factors;sequence=ATCATTAA
ZEB1|NM_001323676	TFBS	TFBS	1640	1647	0.991528411	+	.	TF=HOXA4;class=Homeo domain factors;sequence=ATCATTAA
ZEB1|NM_001323649	TFBS	TFBS	266	273	0.991528411	+	.	TF=HOXA4;class=Homeo domain factors;sequence=ATCATTAA
ZEB1|NM_001323675	TFBS	TFBS	1640	1647	0.991528411	+	.	TF=HOXA4;class=Homeo domain factors;sequence=ATCATTAA
ZEB1|NM_001323672	TFBS	TFBS	1640	1647	0.991528411	+	.	TF=HOXA4;class=Homeo domain factors;sequence=ATCATTAA
ZEB1|NM_001323671	TFBS	TFBS	1640	1647	0.991528411	+	.	TF=HOXA4;class=Homeo domain factors;sequence=ATCATTAA
ZEB1|NM_001323642	TFBS	TFBS	664	671	0.991528411	+	.	TF=HOXA4;class=Homeo domain factors;sequence=ATCATTAA
ZEB1|NM_001174096	TFBS	TFBS	266	273	0.991528411	+	.	TF=HOXA4;class=Homeo domain factors;sequence=ATCATTAA
ZEB1|NM_030751	TFBS	TFBS	266	273	0.991528411	+	.	TF=HOXA4;class=Homeo domain factors;sequence=ATCATTAA
ZEB1|NM_001323638	TFBS	TFBS	395	402	0.991524102	-	.	TF=GSX2;class=Homeo domain factors;sequence=GTCATTAA
ZEB1|NM_001323643	TFBS	TFBS	395	402	0.991524102	-	.	TF=GSX2;class=Homeo domain factors;sequence=GTCATTAA
ZEB1|NM_001323647	TFBS	TFBS	2325	2333	0.991488497	-	.	TF=THAP1;class=C2CH THAP-type zinc

								finger factors;sequence=CTGCCCTCA
ZEB1|NM_001128128	TF BS	TF BS	2325	2333	0.991488497	-	.	TF=THAP1;class=C2CH THAP-type zinc finger factors;sequence=CTGCCCTCA
ZEB1|NM_001323641	TF BS	TF BS	2325	2333	0.991488497	-	.	TF=THAP1;class=C2CH THAP-type zinc finger factors;sequence=CTGCCCTCA
ZEB1|NM_001323638	TF BS	TF BS	2325	2333	0.991488497	-	.	TF=THAP1;class=C2CH THAP-type zinc finger factors;sequence=CTGCCCTCA
ZEB1|NM_001323644	TF BS	TF BS	2325	2333	0.991488497	-	.	TF=THAP1;class=C2CH THAP-type zinc finger factors;sequence=CTGCCCTCA
ZEB1|NM_001323643	TF BS	TF BS	2325	2333	0.991488497	-	.	TF=THAP1;class=C2CH THAP-type zinc finger factors;sequence=CTGCCCTCA
ZEB1|NM_001323674	TF BS	TF BS	2325	2333	0.991488497	-	.	TF=THAP1;class=C2CH THAP-type zinc finger factors;sequence=CTGCCCTCA
ZEB1|NM_001323673	TF BS	TF BS	2325	2333	0.991488497	-	.	TF=THAP1;class=C2CH THAP-type zinc finger factors;sequence=CTGCCCTCA
ZEB1|NM_001323658	TF BS	TF BS	2325	2333	0.991488497	-	.	TF=THAP1;class=C2CH THAP-type zinc finger factors;sequence=CTGCCCTCA
ZEB1|NM_0	TF	TF	23	23	0.9914	-	.	TF=THAP1;class=C2C

01323657	BS	BS	25	33	88497			H THAP-type zinc finger factors;sequence=CTG CCCTCA
ZEB1|NM_0 01323650	TF BS	TF BS	23 25	23 33	0.9914 88497	-	.	TF=THAP1;class=C2C H THAP-type zinc finger factors;sequence=CTG CCCTCA
ZEB1|NM_0 01323648	TF BS	TF BS	23 25	23 33	0.9914 88497	-	.	TF=THAP1;class=C2C H THAP-type zinc finger factors;sequence=CTG CCCTCA
ZEB1|NM_0 01174095	TF BS	TF BS	23 25	23 33	0.9914 88497	-	.	TF=THAP1;class=C2C H THAP-type zinc finger factors;sequence=CTG CCCTCA
ZEB1|NM_0 01323646	TF BS	TF BS	23 25	23 33	0.9914 88497	-	.	TF=THAP1;class=C2C H THAP-type zinc finger factors;sequence=CTG CCCTCA
ZEB1|NM_0 01323645	TF BS	TF BS	23 25	23 33	0.9914 88497	-	.	TF=THAP1;class=C2C H THAP-type zinc finger factors;sequence=CTG CCCTCA
ZEB1|NM_0 01174093	TF BS	TF BS	23 25	23 33	0.9914 88497	-	.	TF=THAP1;class=C2C H THAP-type zinc finger factors;sequence=CTG CCCTCA
ZEB1|NM_0 01323656	TF BS	TF BS	23 25	23 33	0.9914 88497	-	.	TF=THAP1;class=C2C H THAP-type zinc finger factors;sequence=CTG CCCTCA
ZEB1|NM_0 01323655	TF BS	TF BS	23 25	23 33	0.9914 88497	-	.	TF=THAP1;class=C2C H THAP-type zinc finger factors;sequence=CTG CCCTCA

ZEB1|NM_001323654	TF BS	TF BS	2325	2333	0.991488497	-	.	TF=THAP1;class=C2CH THAP-type zinc finger factors;sequence=CTGCCCTCA
ZEB1|NM_001323653	TF BS	TF BS	2325	2333	0.991488497	-	.	TF=THAP1;class=C2CH THAP-type zinc finger factors;sequence=CTGCCCTCA
ZEB1|NM_001323652	TF BS	TF BS	2325	2333	0.991488497	-	.	TF=THAP1;class=C2CH THAP-type zinc finger factors;sequence=CTGCCCTCA
ZEB1|NM_001323651	TF BS	TF BS	2325	2333	0.991488497	-	.	TF=THAP1;class=C2CH THAP-type zinc finger factors;sequence=CTGCCCTCA
ZEB1|NM_001323678	TF BS	TF BS	2325	2333	0.991488497	-	.	TF=THAP1;class=C2CH THAP-type zinc finger factors;sequence=CTGCCCTCA
ZEB1|NM_001323677	TF BS	TF BS	2325	2333	0.991488497	-	.	TF=THAP1;class=C2CH THAP-type zinc finger factors;sequence=CTGCCCTCA
ZEB1|NM_001323676	TF BS	TF BS	2325	2333	0.991488497	-	.	TF=THAP1;class=C2CH THAP-type zinc finger factors;sequence=CTGCCCTCA
ZEB1|NM_001323649	TF BS	TF BS	2325	2333	0.991488497	-	.	TF=THAP1;class=C2CH THAP-type zinc finger factors;sequence=CTGCCCTCA
ZEB1|NM_001323675	TF BS	TF BS	2325	2333	0.991488497	-	.	TF=THAP1;class=C2CH THAP-type zinc finger factors;sequence=CTG

								CCCTCA
ZEB1|NM_001323672	TFBS	TFBS	2325	2333	0.991488497	-	.	TF=THAP1;class=C2CH THAP-type zinc finger factors;sequence=CTGCCCTCA
ZEB1|NM_001323671	TFBS	TFBS	2325	2333	0.991488497	-	.	TF=THAP1;class=C2CH THAP-type zinc finger factors;sequence=CTGCCCTCA
ZEB1|NM_001323666	TFBS	TFBS	2325	2333	0.991488497	-	.	TF=THAP1;class=C2CH THAP-type zinc finger factors;sequence=CTGCCCTCA
ZEB1|NM_001323642	TFBS	TFBS	2325	2333	0.991488497	-	.	TF=THAP1;class=C2CH THAP-type zinc finger factors;sequence=CTGCCCTCA
ZEB1|NM_001174096	TFBS	TFBS	2325	2333	0.991488497	-	.	TF=THAP1;class=C2CH THAP-type zinc finger factors;sequence=CTGCCCTCA
ZEB1|NM_030751	TFBS	TFBS	2325	2333	0.991488497	-	.	TF=THAP1;class=C2CH THAP-type zinc finger factors;sequence=CTGCCCTCA
ZEB1|NM_001323665	TFBS	TFBS	2325	2333	0.991488497	-	.	TF=THAP1;class=C2CH THAP-type zinc finger factors;sequence=CTGCCCTCA
ZEB1|NM_001323664	TFBS	TFBS	2325	2333	0.991488497	-	.	TF=THAP1;class=C2CH THAP-type zinc finger factors;sequence=CTGCCCTCA
ZEB1|NM_001323663	TFBS	TFBS	2325	2333	0.991488497	-	.	TF=THAP1;class=C2CH THAP-type zinc finger

								factors;sequence=CTGCCCTCA
ZEB1|NM_001323662	TFBS	TFBS	2325	2333	0.991488497	-	.	TF=THAP1;class=C2CH THAP-type zinc finger factors;sequence=CTGCCCTCA
ZEB1|NM_001323661	TFBS	TFBS	2325	2333	0.991488497	-	.	TF=THAP1;class=C2CH THAP-type zinc finger factors;sequence=CTGCCCTCA
ZEB1|NM_001323660	TFBS	TFBS	2325	2333	0.991488497	-	.	TF=THAP1;class=C2CH THAP-type zinc finger factors;sequence=CTGCCCTCA
ZEB1|NM_001323659	TFBS	TFBS	2325	2333	0.991488497	-	.	TF=THAP1;class=C2CH THAP-type zinc finger factors;sequence=CTGCCCTCA
ZEB1|NM_001174094	TFBS	TFBS	2325	2333	0.991488497	-	.	TF=THAP1;class=C2CH THAP-type zinc finger factors;sequence=CTGCCCTCA
ZEB1|NM_001323673	TFBS	TFBS	1666	1675	0.991127936	-	.	TF=MNX1;class=Homeo domain factors;sequence=AATAATTAAA
ZEB1|NM_001323677	TFBS	TFBS	1666	1675	0.991127936	-	.	TF=MNX1;class=Homeo domain factors;sequence=AATAATTAAA
ZEB1|NM_001323676	TFBS	TFBS	1666	1675	0.991127936	-	.	TF=MNX1;class=Homeo domain factors;sequence=AATAATTAAA
ZEB1|NM_001323675	TFBS	TFBS	1666	1675	0.991127936	-	.	TF=MNX1;class=Homeo domain factors;sequence=AATAATTAAA
ZEB1|NM_0	TF	TF	16	16	0.9911	-	.	TF=MNX1;class=Home

01323672	BS	BS	66	75	27936			o domain factors;sequence=AATAATTAAA
ZEB1|NM_001323671	TFBS	TFBS	1666	1675	0.991127936	-	.	TF=MNX1;class=Homeo domain factors;sequence=AATAATTAAA
ZEB1|NM_001128128	TFBS	TFBS	1369	1378	0.9906813	+	.	TF=NFIA;class=SMAD/NF-1 DNA-binding domain factors;sequence=TGTGCCAAGG
ZEB1|NM_001323673	TFBS	TFBS	910	919	0.9906813	+	.	TF=NFIA;class=SMAD/NF-1 DNA-binding domain factors;sequence=TGTGCCAAGG
ZEB1|NM_001323678	TFBS	TFBS	1369	1378	0.9906813	+	.	TF=NFIA;class=SMAD/NF-1 DNA-binding domain factors;sequence=TGTGCCAAGG
ZEB1|NM_001323677	TFBS	TFBS	910	919	0.9906813	+	.	TF=NFIA;class=SMAD/NF-1 DNA-binding domain factors;sequence=TGTGCCAAGG
ZEB1|NM_001323676	TFBS	TFBS	910	919	0.9906813	+	.	TF=NFIA;class=SMAD/NF-1 DNA-binding domain factors;sequence=TGTGCCAAGG
ZEB1|NM_001323675	TFBS	TFBS	910	919	0.9906813	+	.	TF=NFIA;class=SMAD/NF-1 DNA-binding domain factors;sequence=TGTGCCAAGG
ZEB1|NM_001323672	TFBS	TFBS	910	919	0.9906813	+	.	TF=NFIA;class=SMAD/NF-1 DNA-binding domain factors;sequence=TGTGCCAAGG
ZEB1|NM_001323671	TFBS	TFBS	910	919	0.9906813	+	.	TF=NFIA;class=SMAD/NF-1 DNA-binding

								domain factors;sequence=TGTGCCAAGG
ZEB1|NM_001323666	TFBS	TFBS	1369	1378	0.9906813	+	.	TF=NFIA;class=SMAD/NF-1 DNA-binding domain factors;sequence=TGTGCCAAGG
ZEB1|NM_001323665	TFBS	TFBS	1369	1378	0.9906813	+	.	TF=NFIA;class=SMAD/NF-1 DNA-binding domain factors;sequence=TGTGCCAAGG
ZEB1|NM_001323664	TFBS	TFBS	1369	1378	0.9906813	+	.	TF=NFIA;class=SMAD/NF-1 DNA-binding domain factors;sequence=TGTGCCAAGG
ZEB1|NM_001323663	TFBS	TFBS	1369	1378	0.9906813	+	.	TF=NFIA;class=SMAD/NF-1 DNA-binding domain factors;sequence=TGTGCCAAGG
ZEB1|NM_001323662	TFBS	TFBS	1369	1378	0.9906813	+	.	TF=NFIA;class=SMAD/NF-1 DNA-binding domain factors;sequence=TGTGCCAAGG
ZEB1|NM_001174094	TFBS	TFBS	1369	1378	0.9906813	+	.	TF=NFIA;class=SMAD/NF-1 DNA-binding domain factors;sequence=TGTGCCAAGG
ZEB1|NM_001128128	TFBS	TFBS	1908	1919	0.990464788	+	.	TF=KLF4;class=C2H2 zinc finger factors;sequence=AGCCCCACCCCC
ZEB1|NM_001323673	TFBS	TFBS	1449	1460	0.990464788	+	.	TF=KLF4;class=C2H2 zinc finger factors;sequence=AGCCCCACCCCC
ZEB1|NM_001323678	TFBS	TFBS	1908	1919	0.990464788	+	.	TF=KLF4;class=C2H2 zinc finger factors;sequence=AGC

								CCCACCCCC
ZEB1|NM_001323677	TFBS	TFBS	1449	1460	0.990464788	+	.	TF=KLF4;class=C2H2 zinc finger factors;sequence=AGCCCCACCCCC
ZEB1|NM_001323676	TFBS	TFBS	1449	1460	0.990464788	+	.	TF=KLF4;class=C2H2 zinc finger factors;sequence=AGCCCCACCCCC
ZEB1|NM_001323675	TFBS	TFBS	1449	1460	0.990464788	+	.	TF=KLF4;class=C2H2 zinc finger factors;sequence=AGCCCCACCCCC
ZEB1|NM_001323672	TFBS	TFBS	1449	1460	0.990464788	+	.	TF=KLF4;class=C2H2 zinc finger factors;sequence=AGCCCCACCCCC
ZEB1|NM_001323671	TFBS	TFBS	1449	1460	0.990464788	+	.	TF=KLF4;class=C2H2 zinc finger factors;sequence=AGCCCCACCCCC
ZEB1|NM_001323666	TFBS	TFBS	1908	1919	0.990464788	+	.	TF=KLF4;class=C2H2 zinc finger factors;sequence=AGCCCCACCCCC
ZEB1|NM_001323665	TFBS	TFBS	1908	1919	0.990464788	+	.	TF=KLF4;class=C2H2 zinc finger factors;sequence=AGCCCCACCCCC
ZEB1|NM_001323664	TFBS	TFBS	1908	1919	0.990464788	+	.	TF=KLF4;class=C2H2 zinc finger factors;sequence=AGCCCCACCCCC
ZEB1|NM_001323663	TFBS	TFBS	1908	1919	0.990464788	+	.	TF=KLF4;class=C2H2 zinc finger factors;sequence=AGCCCCACCCCC
ZEB1|NM_001323662	TFBS	TFBS	1908	1919	0.990464788	+	.	TF=KLF4;class=C2H2 zinc finger factors;sequence=AGCCCCACCCCC
ZEB1|NM_001174094	TFBS	TFBS	1908	1919	0.990464788	+	.	TF=KLF4;class=C2H2 zinc finger factors;sequence=AGC

CCCACCCCC
