# Supplementary material for: Development of a Ferroptosis-Related lncRNA Signature to Predict the Prognosis and Immune Landscape of Bladder Cancer
Source: Dis Markers. 2021 Jun 20;2021:1031906. doi: 10.1155/2021/1031906 (PMC8238626; doi:10.1155/2021/1031906)
Supplement: Supplementary Materials — Supplementary Figure S1: validation of the risk model with resampling. Note: (a) Kaplan-Meier survival analysis of cases in high-risk group and low-risk group (p < 0.001). (b) ROC curves of the risk score in internal validation dataset. Supplementary Figure S2: Kaplan-Meier subgroup survival analysis in TCGA-BLCA patients. Note: (a) age ≤ 64 (p < 0.001). (b) Age > 64 (p < 0.001). (c) Male (p < 0.001). (d) Female (p < 0.001). (e) Pathological T 1-2 stages (p < 0.001). (f) Pathological T 3-4 stages (p < 0.001). (g) N0 stages (p < 0.001). (h) N1-N3 stages (p < 0.001). (i) M0 stage (p < 0.001). (j) M1 stage (p < 0.05). Supplementary Figure S3: functional prediction of AC090825.1 and MAGI2-AS3. Note: (a) the expression difference of AC090825.1 in BCa samples and paracarcinoma tissue via Wilcoxon signed-rank test. (b) The expression difference of MAGI2-AS3 in BCa samples and paracarcinoma tissue via Wilcoxon signed-rank test. (c) The difference values between the expression of AC090825.1 and MAGI2-AS3 in BCa samples and paracarcinoma samples. (d) The cases suffered poorer survival when the expression value of AC090825.1 was higher than that of MAGI2-AS3 (p < 0.01). (e) The expression levels of AC090825.1 and MAGI2-AS3 were positively correlated both in BCa samples and paracarcinoma samples. (f) Network of lncRNAs, the target genes and their possible transcription factors. (g) UpSet diagram showing 6 overlapped transcription factors. (h) The correlation network of lncRNAs, the target genes and transcription factors. BCa: bladder cancer. Supplementary Table S1: 20 ferroptosis regulators were differentially expressed between BCa and paracarinoma samples. Supplementary Table S2: the Pearson correlation analysis of lncRNAs and ferroptosis regulators. Supplementary Table S3: Kaplan-Meier survival analysis of the lncRNA pairs. Supplementary Table S4: enrichment results from LncSEA website. Supplementary Table S5: GSVA results of different clusters based on ferroptosis regulators [file 1031906.f1.zip › Supplementary_Table_S2.PDF]

Supplementary Table S2. The Pearson correlation analysis of lncRNAs and ferroptosis regulators.

Ferroptosis_gene	lncRNA	pearson.cor	pvalue
ORAI1	MIR4435-2HG	0.515	5.40E-29
ORAI1	AC147651.3	0.518	2.26E-29
ORAI1	CYTOR	0.57	1.82E-36
ATF3	AC025259.3	0.536	1.01E-31
ATM	SAP30L-AS1	0.504	9.99E-28
ATM	BX322557.1	0.523	5.84E-30
ATM	AC024075.1	0.52	1.31E-29
ATM	ATP1B3-AS1	0.501	2.32E-27
ATM	AC118344.1	0.527	1.38E-30
ATM	AC067852.6	0.543	9.90E-33
ATM	AL161725.1	0.511	1.75E-28
ATM	PAXIP1-AS2	0.565	9.57E-36
ATM	AC108449.2	0.523	5.54E-30
ATM	AC053513.1	0.536	9.74E-32
ATM	AL049840.1	0.52	1.24E-29
ATM	AL132989.1	0.525	2.83E-30
ATM	AP003392.1	0.56	4.67E-35
ATM	Z68871.1	0.537	6.49E-32
ATM	AP000941.1	0.503	1.39E-27
ATM	AC138956.2	0.515	4.98E-29
ATM	AC108010.1	0.507	5.64E-28
ATM	NPTN-IT1	0.565	7.82E-36
ATM	AL133230.1	0.516	3.86E-29
ATM	AC000120.1	0.509	3.02E-28
ATM	AC012181.3	0.506	7.39E-28
ATM	AP000766.1	0.515	5.06E-29
ATM	AC253536.3	0.505	7.66E-28
ATM	AC004494.1	0.586	5.80E-39
ZEB1	AL450003.2	0.524	4.10E-30
ZEB1	AP001189.3	0.741	3.60E-72
ZEB1	AC087286.2	0.541	2.38E-32
ZEB1	AC079313.2	0.577	1.55E-37
ZEB1	AC037198.2	0.513	1.05E-28
ZEB1	AC135012.3	0.569	1.91E-36
ZEB1	AC129507.1	0.51	2.24E-28
ZEB1	AC036108.3	0.588	2.38E-39
ZEB1	LINC01235	0.525	3.02E-30
ZEB1	AC012085.2	0.509	2.70E-28
ZEB1	LINC01936	0.616	4.79E-44
ZEB1	AC005291.1	0.513	8.66E-29

ZEB1	MAGI2-AS3	0.72	2.85E-66
ZEB1	CARMN	0.62	1.02E-44
ZEB1	AL445423.1	0.501	2.52E-27
ZEB1	AC134312.5	0.51	2.40E-28
ZEB1	AC027097.2	0.51	2.35E-28
ZEB1	AC090825.1	0.644	3.02E-49
ZEB1	AL136084.3	0.709	1.25E-63
ZEB1	DNM3OS	0.61	6.50E-43
ZEB1	ADAMTS9-AS1	0.62	1.14E-44
ZEB1	CTD-2270F17.1	0.527	1.78E-30
ZEB1	AC005291.2	0.575	2.41E-37
ZEB1	MEF2C-AS1	0.554	3.86E-34
ZEB1	LINC01778	0.529	9.49E-31
ZEB1	AC104083.1	0.603	8.96E-42
ZEB1	AF001548.1	0.574	3.43E-37
ZEB1	AC002398.2	0.543	1.03E-32
ZEB1	AL662860.1	0.541	1.89E-32
ZEB1	AC079313.1	0.56	4.57E-35
ZEB1	AC108463.2	0.62	1.07E-44
ZEB1	AC131097.4	0.576	2.03E-37
ZEB1	AC092376.2	0.534	1.91E-31
ZEB1	AC053503.4	0.604	5.75E-42
ZEB1	AC027449.1	0.511	1.56E-28
ZEB1	AP001189.1	0.561	2.94E-35
ZEB1	AC005180.1	0.674	2.96E-55
ZEB1	MSC-AS1	0.58	4.79E-38
ZEB1	LINC01197	0.555	2.83E-34
ZEB1	AL109741.1	0.612	3.31E-43
ZEB1	FENDRR	0.644	4.53E-49
ZEB1	LINC02202	0.55	1.15E-33
ZEB1	AL133415.1	0.592	5.97E-40
ZEB1	AC116021.2	0.501	2.36E-27
ZEB1	C20orf166-AS1	0.555	2.16E-34
ZEB1	AP003071.4	0.698	8.67E-61
ZEB1	AL365214.2	0.525	2.76E-30
ZEB1	ADAMTS9-AS2	0.666	1.34E-53
ZEB1	AP001107.5	0.605	4.65E-42
ZEB1	AC087521.1	0.528	1.08E-30
ZEB1	AC093010.3	0.546	4.87E-33
ZEB1	AC079789.1	0.52	1.19E-29
ZEB1	AC007671.1	0.511	1.44E-28
ZEB1	AC013553.3	0.544	9.25E-33
ZEB1	LINC01081	0.59	1.10E-39
ZEB1	HAND2-AS1	0.656	1.72E-51

ZEB1	AC005180.2	0.675	1.39E-55
ZEB1	AC092535.4	0.509	2.50E-28
ZEB1	LINC00702	0.591	9.44E-40
ZEB1	AC009093.2	0.574	4.00E-37
ZEB1	MBNL1-AS1	0.62	1.12E-44
ZEB1	MEG3	0.532	3.14E-31
ZEB1	SH3RF3-AS1	0.649	3.60E-50
ZEB1	AC099560.1	0.552	5.74E-34
ZEB1	LINC01614	0.595	2.12E-40
ZEB1	AC093278.2	0.585	8.69E-39
ZEB1	MIR100HG	0.791	1.47E-88
ZEB1	JAZF1-AS1	0.544	9.60E-33
ZEB1	AC090559.1	0.627	4.75E-46
ZEB1	PCAT19	0.506	7.32E-28
ZEB1	AL139393.2	0.523	5.55E-30
ZEB1	AC022893.1	0.512	1.12E-28
ZEB1	AC009093.1	0.619	1.41E-44
ZEB1	ACTA2-AS1	0.702	8.19E-62
ZEB1	AL031651.2	0.625	1.54E-45
ZEB1	AP003071.3	0.55	1.40E-33
ZEB1	LINC01352	0.626	1.03E-45
ZEB1	AC134312.1	0.522	7.22E-30
ZEB1	AC079298.3	0.548	2.17E-33
ZEB1	AC091588.3	0.51	1.90E-28
ZEB1	BX322562.1	0.507	5.63E-28
ZEB1	HLX-AS1	0.515	5.77E-29
SLC38A1	AC092611.2	0.523	5.05E-30
SLC38A1	CYTOR	-0.519	1.77E-29
ABCC1	TCF4-AS1	0.507	4.95E-28
ABCC1	LINC00942	0.593	3.52E-40
ABCC1	AL355303.1	0.574	4.39E-37
ABCC1	AL033397.1	0.626	7.60E-46
ABCC1	AP003119.1	0.536	1.18E-31
ABCC1	AC145207.8	0.527	1.35E-30
IREB2	AC024075.1	0.568	3.39E-36
IREB2	AC005288.1	0.629	2.85E-46
IREB2	AC008764.2	0.528	1.07E-30
IREB2	OIP5-AS1	0.634	2.62E-47
IREB2	AC108449.2	0.567	3.87E-36
IREB2	Z68871.1	0.592	6.20E-40
IREB2	AC138035.1	0.51	2.30E-28
IREB2	AC108010.1	0.629	2.90E-46
IREB2	NPTN-IT1	0.519	1.44E-29
IREB2	AC015849.3	0.508	4.20E-28

IREB2	UBL7-AS1	0.6	3.44E-41
IREB2	AC135050.5	0.528	1.16E-30
IREB2	AC010834.3	0.528	1.31E-30
TF	AC027288.1	0.517	2.78E-29
TF	AC016550.3	0.504	1.20E-27
TF	AC022017.1	0.523	5.65E-30
TF	AC099520.1	0.51	1.97E-28
TF	AC083795.2	0.503	1.55E-27
TF	ZNF385D-AS2	0.505	8.98E-28
TF	LINC01289	0.524	3.53E-30
TF	AC083900.1	0.507	5.28E-28
TF	AL355674.1	0.512	1.14E-28
TF	AL139240.1	0.519	1.74E-29
TF	AL512366.1	0.534	1.90E-31
TF	AL358176.1	0.53	6.64E-31
TF	AP003715.1	0.509	3.02E-28
TF	FREM2-AS1	0.544	9.12E-33
TF	LINC01209	0.534	1.89E-31
TF	AC006150.1	0.508	3.65E-28
TF	AC092958.2	0.508	4.14E-28
TF	AC108721.1	0.507	5.38E-28
TF	AC093893.2	0.511	1.69E-28
TF	AC022367.1	0.543	1.01E-32
TF	AC004470.1	0.51	2.10E-28
TF	AL356022.1	0.502	2.07E-27
TF	AP001652.1	0.523	4.46E-30
TF	AL445430.1	0.52	1.07E-29
TF	AL353597.2	0.501	2.87E-27
TF	AL390243.1	0.502	2.10E-27
TF	LINC02355	0.506	7.49E-28
TF	AL691497.1	0.522	7.39E-30
TF	AC003984.1	0.505	9.53E-28
TF	AC073218.2	0.601	2.42E-41
TF	AL596330.1	0.514	7.29E-29
TF	AC099511.1	0.509	2.85E-28
TF	AC099520.2	0.522	6.96E-30
LTF	LINC00402	0.502	2.04E-27
LTF	AC009041.2	0.516	4.41E-29
LTF	UNQ6494	0.514	6.93E-29
LTF	LINC01781	0.55	1.07E-33
LTF	LINC02285	0.507	5.03E-28
LTF	LINC02384	0.549	1.52E-33
LTF	LINC01857	0.557	1.43E-34
LTF	FAM30A	0.552	6.64E-34

LTF	AL365361.1	0.535	1.31E-31
LTF	LINC02273	0.504	1.01E-27
LTF	LINC00861	0.573	6.06E-37
LTF	AC012645.3	0.508	4.11E-28
LTF	PCED1B-AS1	0.522	7.21E-30
LTF	AC243960.1	0.566	6.49E-36
LTF	AC119428.2	0.505	8.38E-28
LTF	LINC00426	0.549	1.69E-33
LTF	LINC00494	0.507	5.42E-28
LTF	AC006369.1	0.517	3.35E-29
LTF	ITGB2-AS1	0.535	1.32E-31
LTF	LINC02422	0.557	1.47E-34
LTF	AL928768.1	0.543	1.07E-32
LTF	LINC01914	0.507	4.71E-28
BRAF	AC004492.1	0.517	3.28E-29
BRAF	AC024937.3	0.53	7.29E-31
BRAF	USP3-AS1	0.57	1.53E-36
BRAF	NADK2-AS1	0.593	4.12E-40
BRAF	AC007622.2	0.51	2.10E-28
BRAF	MCCC1-AS1	0.578	1.10E-37
BRAF	AC006378.1	0.503	1.41E-27
BRAF	GTF3C2-AS1	0.549	1.50E-33
BRAF	AC092802.2	0.523	5.81E-30
BRAF	AP001094.2	0.501	2.82E-27
BRAF	LINC01873	0.506	6.56E-28
BRAF	AC007390.2	0.575	3.32E-37
BRAF	AC120114.2	0.516	3.43E-29
BRAF	AC018682.1	0.533	2.65E-31
BRAF	AL606489.1	0.506	7.10E-28
BRAF	NCBP2-AS1	0.532	3.08E-31
BRAF	ITPR1-AS1	0.523	5.05E-30
BRAF	ZBTB40-IT1	0.506	6.86E-28
BRAF	AL365436.2	0.517	2.91E-29
BRAF	AP001625.2	0.502	2.13E-27
BRAF	AGAP11	0.509	2.50E-28
BRAF	ACOXL-AS1	0.531	4.76E-31
BRAF	AL590652.1	0.527	1.68E-30
BRAF	AL135999.3	0.521	9.15E-30
BRAF	AP003392.1	0.511	1.67E-28
BRAF	AC009095.1	0.502	2.13E-27
BRAF	AC002064.2	0.508	3.39E-28
BRAF	AC008543.3	0.569	2.14E-36
BRAF	AL731566.1	0.544	7.19E-33
BRAF	ACAP2-IT1	0.526	2.14E-30

BRAF	AC005586.1	0.508	4.29E-28
BRAF	AC078795.1	0.518	2.12E-29
BRAF	AC005746.1	0.512	1.18E-28
BRAF	AC108010.1	0.563	1.58E-35
BRAF	MIR4435-2HG	-0.505	7.58E-28
BRAF	AC109460.2	0.503	1.32E-27
BRAF	AC073957.3	0.512	1.16E-28
BRAF	SEMA6A-AS1	0.503	1.35E-27
BRAF	AC008770.4	0.522	6.67E-30
BRAF	AL450263.1	0.617	4.21E-44
BRAF	AL021068.1	0.56	4.22E-35
BRAF	AC002128.1	0.518	2.12E-29
BRAF	AC090198.1	0.541	2.11E-32
BRAF	AL391834.1	0.533	2.36E-31
BRAF	LINC01336	0.561	3.62E-35
BRAF	NDUFB2-AS1	0.545	5.26E-33
BRAF	AC000120.1	0.505	7.79E-28
BRAF	INE1	0.503	1.50E-27
BRAF	ZNF436-AS1	0.515	4.59E-29
BRAF	AC061975.7	0.621	6.11E-45
BRAF	AC098869.2	0.514	6.59E-29
BRAF	ZDHHC20-IT1	0.523	4.56E-30
BRAF	AC090739.1	0.51	2.31E-28
BRAF	AC025917.1	0.552	6.97E-34
BRAF	CYTOR	-0.565	8.60E-36
BRAF	AL359220.1	0.549	1.83E-33
BRAF	SPAG5-AS1	0.514	7.47E-29
BRAF	AC073333.1	0.503	1.57E-27
BRAF	AC005253.1	0.504	1.27E-27
BRAF	AP000692.1	0.502	1.89E-27
BRAF	AC003681.1	0.512	1.08E-28
BRAF	AC104532.2	0.511	1.53E-28
BRAF	AC002128.2	0.506	7.31E-28
BRAF	AL157838.1	0.527	1.56E-30
BRAF	AC011477.3	0.541	2.16E-32
BRAF	AC007128.1	0.516	3.65E-29
BRAF	AL157786.1	0.549	1.48E-33
BRAF	AC093690.1	0.506	6.16E-28
ALOXE3	AL034376.2	0.509	3.25E-28
CS	AC099850.3	0.511	1.53E-28
HILPDA	AC010655.2	0.668	5.43E-54
LPCAT3	AC005288.1	0.565	1.00E-35
LPCAT3	PLBD1-AS1	0.51	2.21E-28
LPCAT3	AC092611.2	0.52	1.23E-29

LPCAT3	AC108010.1	0.519	1.57E-29
LPCAT3	AC016876.2	-0.573	6.01E-37
LPCAT3	AC012510.1	-0.575	3.12E-37
MDM2	AC025423.1	0.64	2.03E-48
MDM2	AP001372.1	0.613	1.61E-43
PEBP1	LINC00941	-0.503	1.55E-27
PEBP1	AL158206.1	0.561	3.58E-35
PEBP1	MYOSLID	-0.563	1.58E-35
PEBP1	GATA3-AS1	0.525	2.83E-30
PEBP1	AL161772.1	0.537	7.54E-32
PEBP1	AL135999.3	0.521	7.90E-30
PEBP1	AC002384.1	-0.519	1.56E-29
PEBP1	DANCR	0.504	1.29E-27
PEBP1	CEBPA-AS1	0.513	8.80E-29
PEBP1	TBX2-AS1	0.574	3.83E-37
PEBP1	SNHG18	0.523	4.98E-30
PEBP1	AC061975.7	0.529	8.63E-31
PEBP1	AP005432.2	0.581	3.28E-38
PEBP1	AC011477.3	0.51	2.26E-28
PEBP1	AC026765.2	0.501	2.91E-27
ATG13	AC091544.5	-0.504	9.96E-28
BECN1	AC005288.1	0.704	2.87E-62
BECN1	AC016876.2	-0.529	8.58E-31
BECN1	AC012510.1	-0.52	1.36E-29
STAT3	SNHG10	-0.522	7.16E-30
STAT3	AL121829.2	-0.514	6.33E-29
STAT3	AL390294.1	-0.507	4.72E-28
STAT3	AC073534.2	-0.502	1.83E-27
STAT3	GATA3-AS1	-0.543	1.03E-32
STAT3	AL450384.2	-0.505	8.23E-28
STAT3	AP000866.5	-0.501	2.60E-27
STAT3	HCP5	0.546	4.03E-33
STAT3	AP003352.1	-0.518	2.49E-29
STAT3	DANCR	-0.518	2.50E-29
STAT3	SNHG9	-0.513	1.01E-28
STAT3	TBX2-AS1	-0.549	1.87E-33
STAT3	AC016876.3	-0.501	2.96E-27
STAT3	SNHG7	-0.507	5.45E-28
STAT3	AC005785.1	-0.501	2.70E-27
CTSB	AC011899.2	0.554	3.69E-34
CTSB	IQCH-AS1	-0.508	3.65E-28
CTSB	AL109811.3	-0.558	8.17E-35
CTSB	LINC01094	0.69	5.68E-59
CTSB	AC006435.2	-0.533	2.69E-31

CTSB	AC004982.1	-0.521	7.96E-30
CTSB	AL158206.1	-0.543	1.28E-32
CTSB	AL390294.1	-0.536	9.94E-32
CTSB	AC136475.2	0.515	4.66E-29
CTSB	PTOV1-AS2	-0.53	5.88E-31
CTSB	AC010761.1	-0.512	1.33E-28
CTSB	AL359715.3	-0.525	2.77E-30
CTSB	AC004921.1	0.556	2.08E-34
CTSB	AL023284.4	-0.509	2.87E-28
CTSB	PAXIP1-AS1	-0.506	6.14E-28
CTSB	AL139287.1	-0.52	1.36E-29
CTSB	AL133371.2	0.532	3.11E-31
CTSB	GATA3-AS1	-0.57	1.48E-36
CTSB	AL450384.2	-0.527	1.67E-30
CTSB	AC004148.2	-0.578	9.59E-38
CTSB	AL590652.1	-0.505	8.74E-28
CTSB	SH3BP5-AS1	-0.535	1.51E-31
CTSB	AL022322.1	-0.532	3.75E-31
CTSB	LINC01355	-0.506	6.95E-28
CTSB	AC099066.2	0.533	2.43E-31
CTSB	AC074327.1	0.512	1.24E-28
CTSB	AC017002.3	0.513	9.70E-29
CTSB	AL691482.3	-0.536	1.05E-31
CTSB	SMIM25	0.695	3.10E-60
CTSB	AC006042.1	-0.567	4.10E-36
CTSB	AL390719.2	-0.596	1.56E-40
CTSB	AL133410.1	-0.52	1.34E-29
CTSB	LINC01943	0.571	1.18E-36
CTSB	MIR4435-2HG	0.594	3.16E-40
CTSB	PRKCZ-AS1	-0.506	6.41E-28
CTSB	VIM-AS1	0.51	1.97E-28
CTSB	AC147651.3	0.595	1.75E-40
CTSB	AC108860.2	-0.537	8.13E-32
CTSB	AC108134.1	-0.508	3.76E-28
CTSB	AL671710.1	-0.501	2.30E-27
CTSB	AC016738.1	-0.502	1.96E-27
CTSB	TTC28-AS1	-0.507	5.49E-28
CTSB	AL359715.2	-0.538	6.20E-32
CTSB	ZNF436-AS1	-0.585	7.83E-39
CTSB	AC073335.2	-0.55	1.06E-33
CTSB	KRT7-AS	-0.525	2.89E-30
CTSB	AC061975.7	-0.522	7.55E-30
CTSB	MCF2L-AS1	-0.54	2.65E-32
CTSB	AC084125.2	-0.511	1.72E-28

CTSB	AC004982.2	-0.521	9.35E-30
CTSB	CYTOR	0.613	2.12E-43
CTSB	ZNF32-AS1	-0.567	3.90E-36
CTSB	AC006033.2	0.532	3.27E-31
CTSB	AC091544.5	-0.537	6.86E-32
CTSB	AC090559.1	0.574	3.84E-37
CTSB	AC107068.1	-0.523	4.56E-30
CTSB	GAPLINC	0.515	5.65E-29
CTSB	AC003070.1	-0.51	2.10E-28
CTSB	AC011477.3	-0.583	1.37E-38
CTSB	AC010503.4	-0.519	1.74E-29
CTSB	TUG1	-0.513	1.02E-28
CDKN2A	CDKN2B-AS1	0.633	5.09E-47
CDKN2A	LINC00294	0.502	1.76E-27
CDKN2A	AL449423.1	0.665	1.97E-53
CDKN2A	CDKN2A-AS1	0.745	2.05E-73
EIF2AK2	AC099850.3	0.509	3.07E-28
EIF2AK2	SNHG9	-0.535	1.25E-31
EIF2AK2	AP001610.1	0.567	4.66E-36
EIF2AK2	AL445490.1	0.55	1.43E-33
EIF2AK2	AC009950.1	0.502	1.97E-27
KEAP1	AC114271.1	0.544	8.94E-33
CYBB	U62317.5	0.567	4.36E-36
CYBB	AC034238.1	0.565	9.33E-36
CYBB	AL035530.1	0.509	2.53E-28
CYBB	AC004865.2	0.671	1.22E-54
CYBB	AF127936.1	0.701	1.27E-61
CYBB	LINC01684	0.53	6.40E-31
CYBB	LINC02446	0.617	4.45E-44
CYBB	AC079921.2	0.557	1.44E-34
CYBB	AC034199.1	0.585	6.63E-39
CYBB	AC011899.2	0.806	1.64E-94
CYBB	AC007569.1	0.529	7.40E-31
CYBB	ZEB2-AS1	0.582	2.67E-38
CYBB	AL133467.1	0.547	3.16E-33
CYBB	LINC01094	0.843	1.71E-111
CYBB	AC021188.1	0.576	2.33E-37
CYBB	AC004687.1	0.542	1.68E-32
CYBB	LINC02345	0.605	3.80E-42
CYBB	CARD8-AS1	0.565	7.72E-36
CYBB	AC136475.2	0.56	5.20E-35
CYBB	AL590764.1	0.73	4.84E-69
CYBB	AC243960.3	0.527	1.46E-30
CYBB	AC004921.1	0.747	6.71E-74

CYBB	MIR155HG	0.693	1.10E-59
CYBB	AP003774.4	0.508	3.26E-28
CYBB	PIK3CD-AS1	0.538	5.86E-32
CYBB	AC015819.1	0.637	8.91E-48
CYBB	AC109446.3	0.51	2.25E-28
CYBB	AL023284.4	-0.553	4.49E-34
CYBB	U62317.2	0.526	1.87E-30
CYBB	LINC01150	0.654	3.74E-51
CYBB	LINC02285	0.717	1.11E-65
CYBB	AC023449.2	0.522	6.51E-30
CYBB	AC011899.3	0.708	3.04E-63
CYBB	AC245128.3	0.554	2.91E-34
CYBB	AL133371.2	0.774	1.41E-82
CYBB	GATA3-AS1	-0.52	1.38E-29
CYBB	AC012236.1	0.517	3.32E-29
CYBB	LINC01857	0.586	6.36E-39
CYBB	AL162414.1	0.534	1.77E-31
CYBB	AL365361.1	0.648	7.40E-50
CYBB	AC064805.1	0.503	1.60E-27
CYBB	AC074327.1	0.579	5.78E-38
CYBB	AC017002.3	0.629	2.28E-46
CYBB	LINC00996	0.718	6.30E-66
CYBB	TMEM51-AS1	-0.507	5.18E-28
CYBB	SMIM25	0.81	2.67E-96
CYBB	LINC02084	0.575	2.74E-37
CYBB	LINC00861	0.586	5.03E-39
CYBB	AP002954.1	0.639	2.92E-48
CYBB	LINC02362	0.516	3.41E-29
CYBB	FO393401.1	0.542	1.35E-32
CYBB	AC004988.1	0.589	2.09E-39
CYBB	AL390719.2	-0.563	1.79E-35
CYBB	AC145098.1	0.577	1.52E-37
CYBB	HCP5	0.527	1.68E-30
CYBB	L3MBTL4-AS1	0.673	4.54E-55
CYBB	TRG-AS1	0.777	1.37E-83
CYBB	LINC01678	0.603	1.06E-41
CYBB	LINC01943	0.755	2.77E-76
CYBB	MIR4435-2HG	0.508	3.52E-28
CYBB	AL034397.3	0.578	8.57E-38
CYBB	AC022126.1	0.537	6.85E-32
CYBB	LINC01506	0.554	3.31E-34
CYBB	VIM-AS1	0.611	4.09E-43
CYBB	AC147651.3	0.747	5.89E-74
CYBB	LINC02325	0.575	2.58E-37

CYBB	PCED1B-AS1	0.79	4.16E-88
CYBB	AC243960.1	0.635	1.67E-47
CYBB	AC004847.1	0.636	1.32E-47
CYBB	AC243829.4	0.641	1.40E-48
CYBB	AC138207.5	0.727	3.32E-68
CYBB	LINC00528	0.55	1.22E-33
CYBB	AL354732.1	0.537	8.37E-32
CYBB	TRBV11-2	0.602	1.54E-41
CYBB	USP30-AS1	0.636	1.33E-47
CYBB	LINC01934	0.62	1.23E-44
CYBB	AC008105.3	0.588	2.45E-39
CYBB	LINC00426	0.689	1.23E-58
CYBB	AL161935.3	0.624	1.99E-45
CYBB	LINC02195	0.632	6.65E-47
CYBB	AC004585.1	0.638	5.86E-48
CYBB	PSMB8-AS1	0.648	6.70E-50
CYBB	AC069209.1	0.604	5.78E-42
CYBB	AC007728.2	0.623	2.57E-45
CYBB	AL592164.1	0.548	2.56E-33
CYBB	AL590648.3	0.502	2.14E-27
CYBB	C9orf139	0.603	1.12E-41
CYBB	AC083949.1	0.628	4.47E-46
CYBB	AC006369.1	0.584	1.13E-38
CYBB	LINC01614	0.519	1.63E-29
CYBB	CYTOR	0.567	4.59E-36
CYBB	ITGB2-AS1	0.674	2.52E-55
CYBB	MMP2-AS1	0.509	2.88E-28
CYBB	AC006033.2	0.768	1.73E-80
CYBB	AL591468.1	0.663	4.35E-53
CYBB	AC091544.5	-0.51	1.87E-28
CYBB	AC090559.1	0.841	2.02E-110
CYBB	LINC01914	0.53	5.84E-31
CYBB	LINC01871	0.706	6.62E-63
CYBB	AL390036.1	0.568	3.03E-36
CYBB	LINC02099	0.539	4.40E-32
CYBB	AL023653.1	0.504	1.26E-27
CYBB	HLA-DQB1-AS1	0.652	8.88E-51
CYBB	GAPLINC	0.514	6.02E-29
CYBB	AC110995.1	0.507	5.02E-28
CYBB	AC015911.3	0.682	4.55E-57
CYBB	AC008750.1	0.501	2.79E-27
CYBB	LINC01281	0.58	4.75E-38
CYBB	AC010503.4	-0.506	6.16E-28
CYBB	AC109826.1	0.678	3.01E-56

DPP4	LINC01094	0.517	2.85E-29
DPP4	AC008063.1	0.741	2.35E-72
HDDC3	AC068338.2	0.577	1.46E-37
HDDC3	AC023509.4	0.522	6.62E-30
HDDC3	AC068831.2	0.503	1.32E-27
NOX4	LINC01638	0.556	1.57E-34
NOX4	AC090125.1	0.548	2.11E-33
NOX4	AC027288.3	0.559	6.91E-35
NOX4	MAGI2-AS3	0.514	6.14E-29
NOX4	AC134312.5	0.62	1.14E-44
NOX4	LINC02104	0.559	6.54E-35
NOX4	LINC01050	0.517	2.67E-29
NOX4	AC104083.1	0.508	3.46E-28
NOX4	AC108463.2	0.533	2.84E-31
NOX4	AL136129.1	0.684	1.12E-57
NOX4	LINC01048	0.539	4.25E-32
NOX4	AC090673.1	0.545	6.66E-33
NOX4	LINC01943	0.53	5.56E-31
NOX4	LINC01705	0.571	1.08E-36
NOX4	FNDC1-IT1	0.538	4.76E-32
NOX4	AL354732.1	0.645	2.18E-49
NOX4	AC009093.2	0.634	2.47E-47
NOX4	AC004160.1	0.521	8.01E-30
NOX4	AL356417.2	0.705	1.63E-62
NOX4	LINC01614	0.655	2.41E-51
NOX4	CYTOR	0.507	5.00E-28
NOX4	AC007750.1	0.629	2.16E-46
NOX4	LINC01561	0.579	6.51E-38
NOX4	AC009093.1	0.643	5.29E-49
NOX4	GAPLINC	0.63	1.98E-46
NOX4	AL031651.2	0.518	2.37E-29
NOX4	AL162457.2	0.579	5.88E-38
NOX4	AC079298.3	0.556	1.52E-34
AKR1C1	TCF4-AS1	0.513	8.23E-29
AKR1C1	AL391427.1	0.675	1.23E-55
AKR1C1	AL355303.1	0.573	5.30E-37
AKR1C1	AL033397.1	0.591	7.58E-40
AKR1C1	AC005722.3	0.546	4.78E-33
AKR1C1	AP003119.1	0.544	9.65E-33
AKR1C2	LINC01564	0.576	2.01E-37
AKR1C2	AL391427.1	0.749	1.28E-74
AKR1C2	TP53TG1	0.527	1.73E-30
AKR1C3	LINC01564	0.516	3.91E-29
AKR1C3	COLCA1	0.513	9.23E-29

AKR1C3	AL391427.1	0.707	4.13E-63
CDH1	ZEB2-AS1	-0.501	2.47E-27
CDH1	SPINT1-AS1	0.529	8.35E-31
CDH1	AL035661.1	0.566	7.30E-36
CDH1	AC010503.4	0.534	1.80E-31
ITGA6	AC078883.1	0.548	2.20E-33
ITGB4	AC245041.3	0.533	2.72E-31
ITGB4	MIR205HG	0.562	2.12E-35
CD44	AL133330.1	0.663	4.28E-53
CD44	AC080129.2	-0.53	7.07E-31
CD44	LINC00941	0.617	3.06E-44
CD44	AL121829.2	-0.514	6.06E-29
CD44	AC004982.1	-0.545	6.25E-33
CD44	AL158206.1	-0.561	2.99E-35
CD44	AC108112.1	-0.501	2.85E-27
CD44	AP000866.2	-0.511	1.63E-28
CD44	AL390294.1	-0.51	2.29E-28
CD44	MYOSLID	0.606	3.54E-42
CD44	LINC01833	-0.513	8.69E-29
CD44	PAXIP1-AS1	-0.532	3.96E-31
CD44	FAM83A-AS1	0.501	2.44E-27
CD44	AC012615.1	-0.525	3.07E-30
CD44	AC104825.3	-0.508	4.17E-28
CD44	AL358075.2	-0.504	1.02E-27
CD44	GATA3-AS1	-0.574	3.49E-37
CD44	LINC01534	-0.506	7.03E-28
CD44	AP000866.5	-0.534	1.72E-31
CD44	AL135999.3	-0.586	5.50E-39
CD44	LINC01213	-0.518	2.00E-29
CD44	LINC00460	0.509	3.05E-28
CD44	AL691482.3	-0.518	2.49E-29
CD44	Z73429.1	-0.512	1.29E-28
CD44	AC006042.1	-0.539	4.11E-32
CD44	AL390719.2	-0.516	4.30E-29
CD44	AC002384.1	0.518	2.31E-29
CD44	CEBPA-AS1	-0.528	1.17E-30
CD44	AC108860.2	-0.575	3.12E-37
CD44	AC108134.1	-0.503	1.56E-27
CD44	ELF3-AS1	-0.537	7.09E-32
CD44	TBX2-AS1	-0.575	3.31E-37
CD44	SH3PXD2A-AS1	0.525	2.94E-30
CD44	AL365356.5	0.557	1.11E-34
CD44	SCOC-AS1	-0.51	1.87E-28
CD44	AC061975.7	-0.516	3.81E-29

CD44	LINC02081	0.53	6.49E-31
CD44	MCF2L-AS1	-0.577	1.51E-37
CD44	OSER1-AS1	-0.538	6.15E-32
CD44	AL355353.1	-0.532	3.88E-31
CD44	AC004982.2	-0.546	5.09E-33
CD44	AC008738.2	-0.503	1.67E-27
CD44	AL356215.1	0.599	4.37E-41
CD44	AC011477.3	-0.564	1.10E-35
CD44	CD44-AS1	0.715	5.37E-65
OTUB1	AP003068.1	0.537	8.62E-32
SLC7A11	AC105177.1	0.522	7.75E-30
SLC7A11	AC004990.1	0.54	2.96E-32
SLC7A11	AC078845.1	0.51	1.86E-28
SLC7A11	TCF4-AS1	0.564	1.17E-35
SLC7A11	LINC00536	0.508	4.09E-28
SLC7A11	AL596223.1	0.656	1.82E-51
SLC7A11	AP001596.1	0.574	3.96E-37
SLC7A11	LINC00942	0.682	5.08E-57
SLC7A11	AL355303.1	0.705	1.85E-62
SLC7A11	AL033397.1	0.777	1.85E-83
SLC7A11	SLC7A11-AS1	0.558	1.05E-34
SLC7A11	AC089983.1	0.501	2.74E-27
SLC7A11	AC005722.3	0.587	3.91E-39
SLC7A11	AP003119.1	0.65	2.48E-50
SLC7A11	AC145207.8	0.602	1.45E-41
FANCD2	AC099850.3	0.522	7.59E-30
FANCD2	THUMPD3-AS1	0.539	4.30E-32
TFAM	AC005288.1	0.516	4.09E-29
TFAM	AL731577.2	0.504	1.00E-27
HSPA5	AC010487.2	-0.545	5.70E-33
HSPA5	AL023284.4	-0.508	3.80E-28
HSPA5	AL450384.2	-0.529	9.57E-31
HSPA5	TMEM51-AS1	-0.514	7.87E-29
HSPA5	AL390719.2	-0.506	6.02E-28
HSPA5	AC005785.1	-0.514	6.25E-29
HSPA5	AC013489.1	-0.504	1.05E-27
HSPA5	AC078880.3	-0.539	4.04E-32
HSPA5	AC010300.1	-0.518	2.16E-29
GCLC	AL033397.2	0.742	1.94E-72
GCLC	LINC01564	0.628	3.82E-46
GCLC	AC145207.8	0.531	4.16E-31
CISD1	AL158206.1	-0.529	7.36E-31
CISD1	AL691482.3	-0.511	1.48E-28
CISD1	AC108134.1	-0.512	1.25E-28

CISD1	AC010761.4	-0.506	5.87E-28
CISD1	ZNF436-AS1	-0.501	2.43E-27
CISD1	AC073335.2	-0.531	5.14E-31
CISD1	KRT7-AS	-0.504	1.10E-27
CISD1	AC003070.1	-0.51	2.29E-28
FTMT	AL357127.1	0.505	8.26E-28
FTMT	AL589642.1	0.731	2.02E-69
FTMT	AC116616.1	0.617	4.40E-44
HSPB1	TP53TG1	0.511	1.81E-28
PROM2	AP002761.4	0.53	5.52E-31
PROM2	MIR205HG	0.505	8.76E-28
HIF1A	LINC01089	-0.501	2.56E-27
HIF1A	AL121829.2	-0.518	2.45E-29
HIF1A	AC108673.3	-0.501	2.27E-27
HIF1A	AC024060.1	-0.573	6.27E-37
HIF1A	GATA3-AS1	-0.519	1.67E-29
HIF1A	AP000866.5	-0.508	3.55E-28
HIF1A	H1FX-AS1	-0.511	1.56E-28
HIF1A	AC008105.2	-0.551	9.77E-34
HIF1A	AL390719.2	-0.524	4.12E-30
HIF1A	AC015912.3	-0.509	2.91E-28
HIF1A	TBX2-AS1	-0.542	1.62E-32
HIF1A	SCOC-AS1	-0.512	1.12E-28
HIF1A	AC092849.1	-0.524	3.77E-30
HIF1A	C17orf82	-0.515	5.27E-29
HIF1A	OSER1-AS1	-0.504	1.04E-27
HIF1A	AL355353.1	-0.533	2.73E-31
HIF1A	KMT2E-AS1	-0.502	2.11E-27
HIF1A	AC010331.1	-0.546	4.85E-33
HIF1A	AP000553.1	-0.511	1.42E-28
HIF1A	HIF1A-AS1	0.522	7.36E-30
HIF1A	AC073896.4	-0.515	4.57E-29
HIF1A	AC009065.4	-0.567	4.36E-36
TPD52	AC009686.2	0.542	1.55E-32
VHL	AC005288.1	0.504	1.03E-27
VHL	FGD5-AS1	0.707	5.26E-63
VHL	THUMPD3-AS1	0.58	5.64E-38
VHL	NORAD	0.511	1.56E-28
VHL	AC018809.2	0.562	2.13E-35
VHL	AC060780.1	0.514	5.99E-29
VHL	MAP3K14-AS1	0.503	1.67E-27
VHL	AC011477.3	0.516	3.65E-29
CHMP5	EBLN3P	0.532	3.84E-31
MT1G	LINC01094	0.514	5.98E-29

MT1G	SMIM25	0.551	9.57E-34
MT1G	MIR4435-2HG	0.507	5.48E-28
NQO1	AC092115.3	0.539	4.33E-32
PRDX6	TMEM51-AS1	-0.517	2.94E-29
CAV1	IQCH-AS1	-0.533	2.35E-31
CAV1	LINC00941	0.539	3.59E-32
CAV1	AL121829.2	-0.506	7.45E-28
CAV1	LINC01094	0.503	1.43E-27
CAV1	AL390294.1	-0.536	9.84E-32
CAV1	RASAL2-AS1	0.508	3.50E-28
CAV1	AC012467.2	-0.505	9.90E-28
CAV1	MYOSLID	0.53	6.59E-31
CAV1	AL023284.4	-0.573	5.11E-37
CAV1	SLC16A1-AS1	0.532	3.15E-31
CAV1	SPINT1-AS1	-0.522	7.18E-30
CAV1	GATA3-AS1	-0.535	1.34E-31
CAV1	AC002066.1	0.537	7.11E-32
CAV1	AC004148.2	-0.508	4.16E-28
CAV1	AL590428.1	0.503	1.55E-27
CAV1	TMEM51-AS1	-0.546	5.14E-33
CAV1	AL691482.3	-0.56	5.14E-35
CAV1	AL390719.2	-0.604	6.12E-42
CAV1	RNF157-AS1	-0.503	1.55E-27
CAV1	AC020663.2	-0.507	5.50E-28
CAV1	AC002384.1	0.532	3.29E-31
CAV1	MIR4435-2HG	0.641	1.61E-48
CAV1	DANCR	-0.528	1.25E-30
CAV1	AC108134.1	-0.511	1.51E-28
CAV1	AATBC	-0.534	2.10E-31
CAV1	NKILA	0.501	2.87E-27
CAV1	AL161431.1	0.577	1.45E-37
CAV1	PICSAR	0.556	1.75E-34
CAV1	AC099676.1	-0.506	5.86E-28
CAV1	LINC01615	0.551	9.23E-34
CAV1	MCF2L-AS1	-0.576	1.94E-37
CAV1	CYTOR	0.646	1.32E-49
CAV1	AC007750.1	0.51	2.09E-28
CAV1	AL035661.1	-0.524	3.45E-30
CAV1	AC011477.3	-0.537	7.73E-32
CAV1	AC010503.4	-0.542	1.37E-32
MDM4	AC011477.2	0.518	2.11E-29
MDM4	AC009107.2	0.583	1.94E-38
MDM4	AC016747.3	0.59	1.31E-39
MDM4	AC004492.1	0.524	4.17E-30

MDM4	AC093535.1	0.52	1.32E-29
MDM4	AP003170.3	0.526	1.92E-30
MDM4	AL021878.2	0.53	6.28E-31
MDM4	AC008555.2	0.503	1.35E-27
MDM4	AL008582.1	0.509	2.97E-28
MDM4	AC024937.3	0.607	1.72E-42
MDM4	LINC01089	0.508	3.44E-28
MDM4	AC007216.4	0.55	1.30E-33
MDM4	USP3-AS1	0.579	6.59E-38
MDM4	AC009690.2	0.529	7.76E-31
MDM4	AL096870.2	0.559	5.96E-35
MDM4	AL031670.1	0.519	1.70E-29
MDM4	AC009996.1	0.531	4.27E-31
MDM4	AC024361.1	0.501	2.78E-27
MDM4	TAPT1-AS1	0.53	5.56E-31
MDM4	EDRF1-AS1	0.629	2.29E-46
MDM4	AC008760.1	0.506	6.70E-28
MDM4	LINC00893	0.584	1.31E-38
MDM4	AC007622.2	0.534	1.81E-31
MDM4	AL158163.1	0.611	3.74E-43
MDM4	MCCC1-AS1	0.576	1.92E-37
MDM4	AL157392.3	0.597	1.05E-40
MDM4	AL109811.3	0.574	4.45E-37
MDM4	IGBP1-AS1	0.642	8.28E-49
MDM4	AC087741.1	0.521	8.76E-30
MDM4	AC006435.2	0.631	1.08E-46
MDM4	AL031846.2	0.577	1.32E-37
MDM4	AL450998.3	0.58	4.46E-38
MDM4	AC015813.1	0.539	3.83E-32
MDM4	AL512770.1	0.641	1.24E-48
MDM4	AL031709.1	0.578	1.07E-37
MDM4	AC024075.1	0.538	4.77E-32
MDM4	AL353622.1	0.509	2.51E-28
MDM4	GMDS-AS1	0.533	2.35E-31
MDM4	AL158152.1	0.526	1.85E-30
MDM4	AC006378.1	0.525	2.65E-30
MDM4	SCARNA15	0.614	1.10E-43
MDM4	AL391684.1	0.605	4.55E-42
MDM4	ZNF252P-AS1	0.583	1.90E-38
MDM4	AL354892.3	0.647	7.88E-50
MDM4	AP000866.6	0.522	7.49E-30
MDM4	AP000873.4	0.679	2.37E-56
MDM4	AC005154.1	0.61	6.01E-43
MDM4	AL512791.1	0.576	1.80E-37

MDM4	NARF-IT1	0.551	8.51E-34
MDM4	AC008764.2	0.554	3.26E-34
MDM4	PITRM1-AS1	0.517	2.91E-29
MDM4	AC096586.2	0.619	1.94E-44
MDM4	AC002553.1	0.672	8.17E-55
MDM4	AL049780.1	0.555	2.86E-34
MDM4	AC092802.2	0.603	8.89E-42
MDM4	LINC00894	0.55	1.07E-33
MDM4	AC005519.2	0.708	3.07E-63
MDM4	AC118344.1	0.512	1.21E-28
MDM4	AL031600.1	0.53	5.88E-31
MDM4	AC067852.6	0.546	4.40E-33
MDM4	AC112907.3	0.519	1.73E-29
MDM4	C1orf220	0.594	2.40E-40
MDM4	AF129075.1	0.604	6.14E-42
MDM4	AL355488.2	0.594	3.11E-40
MDM4	LINC00843	0.525	3.06E-30
MDM4	AL683813.1	0.56	5.09E-35
MDM4	Z98885.3	0.53	6.12E-31
MDM4	AC021491.2	0.512	1.25E-28
MDM4	AC010201.2	0.62	9.80E-45
MDM4	AC016737.1	0.535	1.35E-31
MDM4	AL022328.1	0.591	7.31E-40
MDM4	AC004893.2	0.595	2.01E-40
MDM4	AL022311.1	0.565	8.55E-36
MDM4	AC007038.1	0.592	7.07E-40
MDM4	AC116914.2	0.522	6.39E-30
MDM4	UBOX5-AS1	0.566	5.59E-36
MDM4	AC116552.1	0.56	4.06E-35
MDM4	PTOV1-AS2	0.577	1.23E-37
MDM4	AC010761.1	0.564	1.15E-35
MDM4	AC133550.2	0.573	5.31E-37
MDM4	CR936218.1	0.636	1.12E-47
MDM4	AL021707.3	0.524	4.08E-30
MDM4	AC068790.7	0.503	1.36E-27
MDM4	AC129510.1	0.592	6.35E-40
MDM4	SNHG4	0.503	1.63E-27
MDM4	AP002026.1	0.523	5.19E-30
MDM4	AC037459.3	0.574	3.93E-37
MDM4	RBM5-AS1	0.553	4.97E-34
MDM4	AC011462.4	0.549	1.88E-33
MDM4	AP002340.1	0.545	5.46E-33
MDM4	AC007390.2	0.637	9.60E-48
MDM4	AC026362.1	0.574	3.89E-37

MDM4	SEMA3F-AS1	0.666	1.30E-53
MDM4	AL513327.3	0.539	4.01E-32
MDM4	AL135999.1	0.656	1.51E-51
MDM4	AL359715.3	0.57	1.40E-36
MDM4	AC114939.1	0.546	5.20E-33
MDM4	AC005104.1	0.64	1.85E-48
MDM4	GAS8-AS1	0.525	2.95E-30
MDM4	NCBP2-AS1	0.58	4.58E-38
MDM4	AL360091.1	0.516	3.96E-29
MDM4	AC002519.1	0.509	2.70E-28
MDM4	AC013468.1	0.507	5.10E-28
MDM4	AC022154.1	0.509	2.75E-28
MDM4	AC090589.3	0.65	2.18E-50
MDM4	AC025287.3	0.585	8.75E-39
MDM4	AC012170.2	0.647	8.87E-50
MDM4	AC100778.2	0.611	4.86E-43
MDM4	AC245060.6	0.563	1.86E-35
MDM4	AC010761.3	0.559	7.71E-35
MDM4	AC114490.1	0.556	1.74E-34
MDM4	LINC01772	0.577	1.27E-37
MDM4	AC066613.1	0.662	7.11E-53
MDM4	LINC00106	0.539	3.65E-32
MDM4	AL049869.3	0.575	3.31E-37
MDM4	AC010168.2	0.529	8.10E-31
MDM4	Z83843.1	0.702	6.92E-62
MDM4	PSMA3-AS1	0.735	1.48E-70
MDM4	AC010976.1	0.621	6.29E-45
MDM4	AL606760.2	0.516	4.02E-29
MDM4	AC025430.1	0.521	8.43E-30
MDM4	AC048382.2	0.561	3.66E-35
MDM4	AC024075.2	0.596	1.15E-40
MDM4	AP002490.1	0.591	8.04E-40
MDM4	AL359921.1	0.613	2.20E-43
MDM4	AC124319.2	0.605	3.80E-42
MDM4	AC243919.1	0.524	3.28E-30
MDM4	AC023510.2	0.53	5.59E-31
MDM4	AL669831.5	0.548	2.32E-33
MDM4	AC053513.1	0.547	3.01E-33
MDM4	AC004908.1	0.607	1.66E-42
MDM4	AL049840.1	0.615	6.86E-44
MDM4	STAM-AS1	0.508	3.71E-28
MDM4	AC091544.6	0.528	1.15E-30
MDM4	YEATS2-AS1	0.507	4.52E-28
MDM4	AL050343.1	0.53	5.59E-31

MDM4	AC005546.1	0.518	2.01E-29
MDM4	AC011461.1	0.6	3.45E-41
MDM4	AL031320.2	0.575	2.84E-37
MDM4	BCDIN3D-AS1	0.511	1.61E-28
MDM4	AC005070.3	0.525	2.48E-30
MDM4	AC040934.1	0.538	6.26E-32
MDM4	Z97832.2	0.533	2.46E-31
MDM4	AC008735.4	0.593	4.70E-40
MDM4	AC132938.5	0.543	1.17E-32
MDM4	LINC01004	0.647	7.92E-50
MDM4	AC024075.3	0.505	9.81E-28
MDM4	AL132989.1	0.74	6.77E-72
MDM4	AC093752.3	0.624	1.78E-45
MDM4	AC139887.2	0.545	6.07E-33
MDM4	AC004148.2	0.663	5.69E-53
MDM4	MIR3936HG	0.502	2.22E-27
MDM4	AC138150.1	0.534	2.05E-31
MDM4	AP001001.1	0.546	4.52E-33
MDM4	AP4B1-AS1	0.618	2.15E-44
MDM4	AL513190.1	0.54	2.60E-32
MDM4	AP001793.2	0.555	2.81E-34
MDM4	AC120053.1	0.509	2.63E-28
MDM4	AL731569.1	0.52	1.09E-29
MDM4	AC007566.1	0.569	2.32E-36
MDM4	AL590652.1	0.537	7.86E-32
MDM4	OCIAD1-AS1	0.543	1.07E-32
MDM4	AC109587.1	0.53	5.49E-31
MDM4	AC079322.1	0.527	1.62E-30
MDM4	AC018754.1	0.606	2.95E-42
MDM4	SH3BP5-AS1	0.628	3.60E-46
MDM4	AC008894.2	0.595	2.28E-40
MDM4	AL442128.2	0.532	3.98E-31
MDM4	AC025043.1	0.542	1.69E-32
MDM4	AC104964.3	0.533	2.65E-31
MDM4	LINC01355	0.669	3.58E-54
MDM4	AP003392.1	0.564	1.22E-35
MDM4	RHOA-IT1	0.526	1.99E-30
MDM4	PRMT5-AS1	0.576	1.78E-37
MDM4	AL139349.1	0.591	7.47E-40
MDM4	AC139887.4	0.604	5.33E-42
MDM4	AC024267.3	0.558	8.63E-35
MDM4	AC007619.1	0.517	2.65E-29
MDM4	AL355388.2	0.511	1.51E-28
MDM4	AC021321.1	0.502	2.18E-27

MDM4	AL139089.1	0.501	2.50E-27
MDM4	RUSC1-AS1	0.587	4.09E-39
MDM4	AC009095.1	0.612	3.14E-43
MDM4	AC004076.2	0.546	4.74E-33
MDM4	EHMT2-AS1	0.502	1.97E-27
MDM4	AC005329.3	0.508	3.71E-28
MDM4	AL022328.2	0.648	5.31E-50
MDM4	AC008741.2	0.594	2.61E-40
MDM4	AC104564.4	0.578	9.67E-38
MDM4	AC002553.4	0.571	9.80E-37
MDM4	AL138921.1	0.546	4.55E-33
MDM4	AC025034.1	0.529	9.17E-31
MDM4	RAD51-AS1	0.695	4.35E-60
MDM4	AC139887.1	0.623	2.71E-45
MDM4	HM13-IT1	0.572	7.52E-37
MDM4	AC084018.2	0.606	3.43E-42
MDM4	AC095057.3	0.587	3.76E-39
MDM4	AP000866.1	0.554	2.99E-34
MDM4	AL158163.2	0.51	2.44E-28
MDM4	AL353804.2	0.511	1.47E-28
MDM4	AC008735.2	0.6	2.92E-41
MDM4	DCUN1D2-AS	0.503	1.71E-27
MDM4	AC008543.3	0.525	2.75E-30
MDM4	AC006042.1	0.613	1.64E-43
MDM4	AC022400.1	0.512	1.18E-28
MDM4	AC022558.1	0.558	9.50E-35
MDM4	AC073575.2	0.58	5.11E-38
MDM4	AP001107.4	0.581	3.19E-38
MDM4	AL031775.2	0.51	2.36E-28
MDM4	AC012358.1	0.606	2.42E-42
MDM4	AC008124.1	0.515	4.79E-29
MDM4	MALAT1	0.628	3.42E-46
MDM4	AL731566.1	0.543	1.28E-32
MDM4	BMS1P4	0.507	4.31E-28
MDM4	AC010260.1	0.507	4.39E-28
MDM4	AC020558.2	0.539	3.98E-32
MDM4	AL390208.1	0.608	1.30E-42
MDM4	RNF139-AS1	0.524	4.18E-30
MDM4	AC007038.2	0.542	1.36E-32
MDM4	AC138956.2	0.65	2.56E-50
MDM4	ACAP2-IT1	0.51	2.04E-28
MDM4	ZFHX2-AS1	0.596	1.31E-40
MDM4	AL117209.1	0.554	2.89E-34
MDM4	AC138035.1	0.504	1.29E-27

MDM4	AC091185.1	0.539	4.69E-32
MDM4	C1orf132	0.617	3.30E-44
MDM4	AC023355.1	0.514	6.72E-29
MDM4	AC110285.2	0.503	1.58E-27
MDM4	AL354733.3	0.549	1.52E-33
MDM4	CTBP1-AS	0.68	1.00E-56
MDM4	AL035563.1	0.534	1.75E-31
MDM4	AC021078.1	0.647	9.73E-50
MDM4	ERVK13-1	0.571	1.09E-36
MDM4	TH2LCRR	0.562	2.44E-35
MDM4	AC078795.1	0.584	1.09E-38
MDM4	AC087481.3	0.627	5.16E-46
MDM4	AC009120.4	0.594	3.46E-40
MDM4	AL360181.2	0.601	1.88E-41
MDM4	AC211476.2	0.518	2.04E-29
MDM4	AC107375.1	0.519	1.64E-29
MDM4	AL591848.4	0.511	1.43E-28
MDM4	AC114730.3	0.542	1.67E-32
MDM4	AL136295.2	0.531	5.12E-31
MDM4	AC108010.1	0.525	2.76E-30
MDM4	AL139099.3	0.606	3.03E-42
MDM4	AC073842.2	0.532	3.39E-31
MDM4	PRKCZ-AS1	0.528	1.31E-30
MDM4	AC245884.8	0.547	3.26E-33
MDM4	AC109460.2	0.605	3.67E-42
MDM4	AL450384.1	0.506	6.06E-28
MDM4	AL157871.5	0.576	2.07E-37
MDM4	AL596202.1	0.533	2.92E-31
MDM4	AL365330.1	0.64	2.10E-48
MDM4	NPTN-IT1	0.508	3.87E-28
MDM4	AC080162.1	0.508	3.75E-28
MDM4	AC027514.2	0.516	4.31E-29
MDM4	AL158834.2	0.587	3.84E-39
MDM4	AC073957.3	0.591	9.87E-40
MDM4	SEMA6A-AS1	0.586	6.32E-39
MDM4	AL035448.1	0.512	1.15E-28
MDM4	AC254562.3	0.506	6.27E-28
MDM4	AP003096.1	0.559	5.57E-35
MDM4	AC008870.2	0.545	6.10E-33
MDM4	AC027601.1	0.522	7.48E-30
MDM4	AP001628.1	0.528	1.14E-30
MDM4	AC010998.2	0.559	7.49E-35
MDM4	AC008522.1	0.521	8.88E-30
MDM4	AC008770.4	0.575	2.64E-37

MDM4	GABPB1-AS1	0.506	7.28E-28
MDM4	LINC00969	0.505	8.92E-28
MDM4	AC022558.3	0.629	2.56E-46
MDM4	AL450263.1	0.506	5.79E-28
MDM4	AL117350.1	0.527	1.66E-30
MDM4	AC092301.1	0.637	8.31E-48
MDM4	AC090260.1	0.509	3.10E-28
MDM4	AC011489.1	0.589	2.04E-39
MDM4	AL162274.1	0.522	6.37E-30
MDM4	AC079385.1	0.521	9.08E-30
MDM4	FAM13A-AS1	0.583	1.85E-38
MDM4	WASHC5-AS1	0.509	2.86E-28
MDM4	AP002907.1	0.504	1.13E-27
MDM4	AL031717.1	0.594	2.44E-40
MDM4	AL132989.2	0.518	2.35E-29
MDM4	AC012615.6	0.694	5.37E-60
MDM4	AL513365.2	0.555	2.74E-34
MDM4	AC002128.1	0.636	1.25E-47
MDM4	AC018695.4	0.552	5.66E-34
MDM4	AC090198.1	0.605	3.70E-42
MDM4	AC092338.1	0.539	3.51E-32
MDM4	MORF4L2-AS1	0.547	3.17E-33
MDM4	AL592430.1	0.529	9.85E-31
MDM4	AC132872.3	0.505	8.67E-28
MDM4	AC232271.1	0.635	1.89E-47
MDM4	AC008731.1	0.533	2.72E-31
MDM4	AC006480.2	0.509	2.52E-28
MDM4	AL355075.2	0.641	1.36E-48
MDM4	AC016738.1	0.527	1.76E-30
MDM4	AC138393.3	0.55	1.39E-33
MDM4	AL049552.1	0.597	7.98E-41
MDM4	AC253536.6	0.533	2.38E-31
MDM4	AC015802.4	0.521	9.22E-30
MDM4	AC009090.1	0.511	1.40E-28
MDM4	Z83844.2	0.527	1.70E-30
MDM4	AC000120.1	0.565	7.54E-36
MDM4	AC018638.7	0.556	1.76E-34
MDM4	MANEA-AS1	0.637	9.60E-48
MDM4	KLF3-AS1	0.572	7.46E-37
MDM4	INE1	0.667	7.06E-54
MDM4	AC008750.5	0.576	1.90E-37
MDM4	AC137932.3	0.599	3.66E-41
MDM4	AL359715.2	0.569	2.19E-36
MDM4	UBE2Q1-AS1	0.559	6.78E-35

MDM4	AL021707.4	0.585	7.36E-39
MDM4	AC084824.4	0.564	1.09E-35
MDM4	PLCG1-AS1	0.504	1.04E-27
MDM4	AC015849.3	0.624	2.40E-45
MDM4	AC008735.1	0.607	1.84E-42
MDM4	AC055855.2	0.697	1.68E-60
MDM4	ZNF436-AS1	0.551	8.59E-34
MDM4	AC073335.2	0.57	1.57E-36
MDM4	PDXDC2P-NPIPB14P	0.544	7.27E-33
MDM4	AC099676.1	0.566	7.04E-36
MDM4	DPP9-AS1	0.513	8.27E-29
MDM4	LENG8-AS1	0.618	2.01E-44
MDM4	N4BP2L2-IT2	0.647	8.46E-50
MDM4	AC013356.2	0.538	6.37E-32
MDM4	MCM3AP-AS1	0.566	6.21E-36
MDM4	AL390728.6	0.565	9.58E-36
MDM4	AC092794.1	0.595	2.23E-40
MDM4	PSPC1-AS2	0.595	2.34E-40
MDM4	ALG13-AS1	0.642	9.59E-49
MDM4	AC073487.1	0.667	6.21E-54
MDM4	AC098869.2	0.555	2.82E-34
MDM4	AC106864.1	0.562	2.72E-35
MDM4	ZDHHC20-IT1	0.561	3.25E-35
MDM4	AC090510.2	0.518	2.21E-29
MDM4	AL133406.2	0.625	1.56E-45
MDM4	AC090739.1	0.575	3.33E-37
MDM4	AC084125.2	0.55	1.27E-33
MDM4	AL513327.1	0.577	1.39E-37
MDM4	AC127024.4	0.664	2.73E-53
MDM4	AC096642.1	0.596	1.39E-40
MDM4	AL450306.1	0.519	1.50E-29
MDM4	AL354953.1	0.522	7.08E-30
MDM4	AC084018.1	0.636	1.13E-47
MDM4	AC022400.5	0.573	5.14E-37
MDM4	AL139407.1	0.567	3.94E-36
MDM4	AL118558.1	0.538	5.75E-32
MDM4	AC025917.1	0.512	1.33E-28
MDM4	FMR1-IT1	0.57	1.66E-36
MDM4	ZNF32-AS1	0.538	4.76E-32
MDM4	AC010201.1	0.543	1.31E-32
MDM4	PCBP1-AS1	0.514	6.62E-29
MDM4	AC011481.1	0.502	2.22E-27
MDM4	SPAG5-AS1	0.598	6.14E-41
MDM4	SSBP3-AS1	0.567	4.99E-36

MDM4	AC005387.2	0.578	9.32E-38
MDM4	AC011472.1	0.537	7.47E-32
MDM4	PSMD6-AS2	0.555	2.60E-34
MDM4	ARHGAP27P1-BPTFP1-KPNA2P3	0.508	3.33E-28
MDM4	AC023908.3	0.63	1.72E-46
MDM4	AC138956.1	0.639	3.57E-48
MDM4	AC005253.1	0.596	1.19E-40
MDM4	AP001107.1	0.562	2.74E-35
MDM4	AL928654.3	0.659	2.93E-52
MDM4	SNHG20	0.523	5.17E-30
MDM4	AC036103.1	0.527	1.67E-30
MDM4	AC009120.2	0.646	1.41E-49
MDM4	AP000692.1	0.555	2.13E-34
MDM4	AC003681.1	0.51	2.20E-28
MDM4	LINC00265	0.591	9.29E-40
MDM4	AC012170.3	0.576	2.02E-37
MDM4	AC015727.1	0.516	3.64E-29
MDM4	AC104532.2	0.556	1.79E-34
MDM4	AC090607.1	0.521	1.03E-29
MDM4	AC087301.1	0.542	1.54E-32
MDM4	AL117339.4	0.506	6.24E-28
MDM4	AC032044.1	0.524	3.62E-30
MDM4	AC079336.5	0.521	8.04E-30
MDM4	AC008764.8	0.632	6.43E-47
MDM4	AC093788.1	0.559	6.65E-35
MDM4	LINC00641	0.596	1.48E-40
MDM4	AC004461.2	0.53	5.91E-31
MDM4	EXTL3-AS1	0.553	4.61E-34
MDM4	AC092119.2	0.587	4.38E-39
MDM4	AC005154.5	0.521	9.73E-30
MDM4	AC144548.2	0.6	2.97E-41
MDM4	ZNF32-AS2	0.602	1.48E-41
MDM4	AC025918.1	0.54	3.09E-32
MDM4	AC136604.2	0.508	3.33E-28
MDM4	TRAPPC12-AS1	0.519	1.86E-29
MDM4	CAPN10-AS1	0.646	1.35E-49
MDM4	AC004253.1	0.553	4.81E-34
MDM4	AL021368.3	0.598	5.88E-41
MDM4	AL157838.1	0.52	1.06E-29
MDM4	AC003070.1	0.552	5.90E-34
MDM4	AC084824.3	0.632	5.73E-47
MDM4	AC135050.5	0.538	5.02E-32
MDM4	AL034550.1	0.519	1.51E-29
MDM4	AL022328.3	0.651	1.71E-50

MDM4	AC048341.2	0.556	1.87E-34
MDM4	DKFZP434I0714	0.546	4.21E-33
MDM4	NFYC-AS1	0.545	6.18E-33
MDM4	AC010618.2	0.54	3.23E-32
MDM4	CCDC18-AS1	0.643	5.69E-49
MDM4	ZNF346-IT1	0.557	1.26E-34
MDM4	AL136295.7	0.519	1.53E-29
MDM4	AC010530.1	0.52	1.14E-29
MDM4	AL031714.1	0.663	5.26E-53
MDM4	NDUFA6-AS1	0.548	2.31E-33
MDM4	Z84485.1	0.512	1.28E-28
MDM4	AC010834.3	0.577	1.20E-37
MDM4	ANKRD10-IT1	0.654	3.06E-51
MDM4	AC093495.1	0.611	3.83E-43
MDM4	AC092123.2	0.537	6.50E-32
MDM4	AC010320.3	0.516	3.90E-29
MDM4	AC008937.3	0.507	4.74E-28
PRKAA1	NNT-AS1	0.553	5.28E-34
PRKAA1	AC093297.3	0.557	1.40E-34
PRKAA1	AC114956.3	0.558	9.75E-35
TMEM173	LINC01094	0.505	9.50E-28
TMEM173	SMIM25	0.528	1.31E-30
TMEM173	LINC01943	0.516	3.97E-29
TMEM173	MIR4435-2HG	0.541	2.18E-32
TMEM173	DANCR	-0.509	2.58E-28
TMEM173	VIM-AS1	0.553	5.47E-34
TMEM173	AC147651.3	0.536	9.47E-32
TMEM173	PCED1B-AS1	0.515	5.91E-29
TMEM173	AC108860.2	-0.523	5.46E-30
TMEM173	USP30-AS1	0.529	7.96E-31
TMEM173	PSMB8-AS1	0.543	1.16E-32
TMEM173	AC007728.2	0.503	1.47E-27
TMEM173	AC061975.7	-0.532	3.78E-31
TMEM173	CYTOR	0.558	1.04E-34
TMEM173	AL157394.1	0.599	4.18E-41
PRR4	AC006064.2	0.537	8.65E-32
PRR4	AC016747.3	0.64	2.34E-48
PRR4	LINC01911	0.539	3.46E-32
PRR4	AC111182.1	0.518	2.17E-29
PRR4	AP000897.1	0.591	8.24E-40
PRR4	AL139081.1	0.542	1.43E-32
PRR4	AC025031.1	0.567	4.37E-36
PRR4	SAP30L-AS1	0.564	1.33E-35
PRR4	AC013714.1	0.533	2.94E-31

PRR4	USP12-AS1	0.567	4.05E-36
PRR4	AP001922.3	0.546	4.00E-33
PRR4	AC009248.2	0.617	3.48E-44
PRR4	AC073569.1	0.546	5.08E-33
PRR4	AC116424.1	0.611	4.65E-43
PRR4	AC022778.1	0.609	1.04E-42
PRR4	AC106037.2	0.648	5.79E-50
PRR4	AL391863.1	0.61	6.98E-43
PRR4	AC011825.2	0.614	1.21E-43
PRR4	AC020908.3	0.506	6.57E-28
PRR4	AP006248.4	0.519	1.46E-29
PRR4	DIAPH3-AS2	0.548	2.16E-33
PRR4	AL590822.2	0.594	3.05E-40
PRR4	AC068473.2	0.523	5.19E-30
PRR4	ST7-OT4	0.604	6.48E-42
PRR4	LATS2-AS1	0.588	2.47E-39
PRR4	MIR133A1HG	0.613	1.97E-43
PRR4	AC027288.1	0.51	2.01E-28
PRR4	AP003086.3	0.626	1.03E-45
PRR4	AC024937.3	0.562	2.07E-35
PRR4	AC105345.2	0.526	1.96E-30
PRR4	AC087286.1	0.606	3.13E-42
PRR4	AL157955.2	0.556	1.56E-34
PRR4	AL354989.1	0.503	1.51E-27
PRR4	USP3-AS1	0.507	4.39E-28
PRR4	AC016738.2	0.534	1.82E-31
PRR4	AC023855.1	0.542	1.42E-32
PRR4	LARS2-AS1	0.593	4.85E-40
PRR4	AC104984.6	0.585	6.61E-39
PRR4	AC087749.1	0.625	1.33E-45
PRR4	AC064852.1	0.62	8.98E-45
PRR4	AC023154.1	0.51	2.35E-28
PRR4	AC012568.1	0.584	9.62E-39
PRR4	AC092620.3	0.547	3.46E-33
PRR4	THOC7-AS1	0.633	4.39E-47
PRR4	AIRN	0.633	4.01E-47
PRR4	AC012301.1	0.505	7.51E-28
PRR4	AC015853.3	0.528	1.25E-30
PRR4	AC018557.2	0.519	1.59E-29
PRR4	AC004039.1	0.612	2.53E-43
PRR4	AL162253.1	0.564	1.12E-35
PRR4	NADK2-AS1	0.549	1.75E-33
PRR4	AC133106.1	0.541	2.40E-32
PRR4	AP001439.1	0.525	2.51E-30

PRR4	AC068888.2	0.585	6.98E-39
PRR4	AP001350.1	0.586	6.30E-39
PRR4	DPYD-AS2	0.512	1.17E-28
PRR4	AC009996.1	0.521	8.06E-30
PRR4	AC083806.2	0.631	1.11E-46
PRR4	SMAD1-AS2	0.604	6.63E-42
PRR4	AC117500.3	0.643	6.95E-49
PRR4	EDRF1-AS1	0.591	7.30E-40
PRR4	AC009318.1	0.556	1.88E-34
PRR4	AC110813.1	0.615	6.88E-44
PRR4	NAALADL2-AS1	0.55	1.21E-33
PRR4	AC004223.2	0.59	1.51E-39
PRR4	AC008026.3	0.578	8.25E-38
PRR4	AL023755.1	0.517	2.66E-29
PRR4	AC007622.2	0.581	3.58E-38
PRR4	AC010082.1	0.524	3.79E-30
PRR4	Z82217.1	0.648	5.24E-50
PRR4	AL357552.2	0.557	1.09E-34
PRR4	AC244131.2	0.511	1.76E-28
PRR4	AL157392.3	0.501	2.75E-27
PRR4	AC107464.2	0.609	8.84E-43
PRR4	AL162430.2	0.558	9.09E-35
PRR4	AC104109.4	0.554	3.46E-34
PRR4	TSPAN9-IT1	0.618	2.78E-44
PRR4	DOCK4-AS1	0.578	8.96E-38
PRR4	ITPK1-AS1	0.572	9.04E-37
PRR4	AC017002.5	0.523	4.51E-30
PRR4	AC012150.2	0.601	1.74E-41
PRR4	AC131182.1	0.601	1.90E-41
PRR4	AC019270.1	0.585	7.32E-39
PRR4	AC026474.1	0.508	3.25E-28
PRR4	AC092828.1	0.638	4.33E-48
PRR4	AL096772.1	0.583	1.93E-38
PRR4	SALRNA2	0.562	2.11E-35
PRR4	AC005740.3	0.546	4.38E-33
PRR4	AC008871.1	0.511	1.65E-28
PRR4	AL359851.1	0.624	2.22E-45
PRR4	AC012404.2	0.508	3.54E-28
PRR4	AC096564.2	0.511	1.40E-28
PRR4	AL450998.3	0.598	5.35E-41
PRR4	SALRNA3	0.518	2.40E-29
PRR4	AP001429.1	0.554	3.89E-34
PRR4	AL357874.2	0.518	2.51E-29
PRR4	AL360157.1	0.595	1.90E-40

PRR4	AC020661.5	0.617	4.16E-44
PRR4	MTOR-AS1	0.629	2.79E-46
PRR4	AC108449.1	0.546	5.21E-33
PRR4	AC027335.1	0.587	4.25E-39
PRR4	AC073651.1	0.664	3.91E-53
PRR4	AP000266.1	0.557	1.19E-34
PRR4	AC108159.1	0.545	6.76E-33
PRR4	AF127577.2	0.581	3.02E-38
PRR4	Z97652.1	0.524	3.45E-30
PRR4	AC009163.3	0.608	1.30E-42
PRR4	AC036222.2	0.512	1.24E-28
PRR4	AC136424.2	0.585	7.47E-39
PRR4	AC015923.1	0.625	1.23E-45
PRR4	AL158152.1	0.627	5.47E-46
PRR4	AP006259.1	0.582	2.66E-38
PRR4	PRICKLE2-AS2	0.534	1.73E-31
PRR4	Z99289.1	0.571	1.23E-36
PRR4	AL022323.2	0.503	1.62E-27
PRR4	SCARNA15	0.526	2.28E-30
PRR4	AL031005.1	0.615	9.19E-44
PRR4	EGFLAM-AS2	0.509	3.23E-28
PRR4	AP000753.2	0.591	9.34E-40
PRR4	AP001775.2	0.51	2.42E-28
PRR4	AC027514.1	0.633	4.62E-47
PRR4	GTF3C2-AS1	0.558	1.03E-34
PRR4	AL133499.1	0.54	2.95E-32
PRR4	AL031668.1	0.636	1.54E-47
PRR4	AC022973.3	0.665	2.11E-53
PRR4	AL109930.1	0.502	2.22E-27
PRR4	AC010261.2	0.614	1.23E-43
PRR4	COL18A1-AS2	0.577	1.25E-37
PRR4	AC107909.2	0.547	3.73E-33
PRR4	AC009365.2	0.625	1.21E-45
PRR4	AC099811.4	0.615	6.95E-44
PRR4	AC110603.1	0.553	4.53E-34
PRR4	AP003785.1	0.603	9.49E-42
PRR4	AC026782.1	0.546	3.96E-33
PRR4	AL035658.1	0.553	4.96E-34
PRR4	PITRM1-AS1	0.563	1.65E-35
PRR4	AL445685.1	0.509	2.77E-28
PRR4	AC022960.1	0.577	1.33E-37
PRR4	AC092755.1	0.522	6.98E-30
PRR4	AC073130.2	0.507	4.43E-28
PRR4	AC018511.1	0.573	4.75E-37

PRR4	AC009303.2	0.58	5.67E-38
PRR4	AL121900.1	0.62	1.19E-44
PRR4	ZBTB20-AS5	0.6	2.91E-41
PRR4	AC009063.1	0.535	1.46E-31
PRR4	AC092802.2	0.532	3.18E-31
PRR4	AL512506.1	0.54	3.38E-32
PRR4	AC104129.1	0.548	2.32E-33
PRR4	AC105206.1	0.591	9.66E-40
PRR4	AC110048.2	0.505	8.16E-28
PRR4	AC005519.2	0.524	3.56E-30
PRR4	PLCE1-AS1	0.512	1.30E-28
PRR4	AC090518.1	0.553	5.51E-34
PRR4	AC018511.2	0.622	5.25E-45
PRR4	AC067852.6	0.526	1.84E-30
PRR4	AC068790.5	0.522	7.32E-30
PRR4	AC013549.2	0.594	2.84E-40
PRR4	AL161725.1	0.622	4.26E-45
PRR4	AF129075.1	0.542	1.59E-32
PRR4	AC012555.2	0.529	7.52E-31
PRR4	LINC00843	0.594	2.74E-40
PRR4	AL360169.1	0.61	6.76E-43
PRR4	Z98885.3	0.614	1.05E-43
PRR4	AC004943.1	0.61	5.26E-43
PRR4	LRP1-AS	0.504	1.18E-27
PRR4	AL161457.2	0.557	1.29E-34
PRR4	AC008277.1	0.656	1.77E-51
PRR4	LINC01981	0.508	3.59E-28
PRR4	AL591242.1	0.502	1.81E-27
PRR4	AC106873.1	0.618	2.36E-44
PRR4	AC005838.2	0.527	1.60E-30
PRR4	CYYR1-AS1	0.528	1.08E-30
PRR4	AL139022.1	0.598	6.41E-41
PRR4	CNOT10-AS1	0.595	1.69E-40
PRR4	AC083805.2	0.54	3.09E-32
PRR4	AL731577.1	0.594	3.24E-40
PRR4	AL807761.4	0.597	8.56E-41
PRR4	AC009145.3	0.507	4.36E-28
PRR4	EGFLAM-AS3	0.586	5.23E-39
PRR4	AC007038.1	0.548	2.24E-33
PRR4	AC097381.2	0.529	9.08E-31
PRR4	CTB-113P19.1	0.549	1.66E-33
PRR4	AL158832.1	0.632	6.49E-47
PRR4	AC021744.1	0.542	1.35E-32
PRR4	AL020995.1	0.615	6.71E-44

PRR4	AL022238.2	0.632	8.23E-47
PRR4	BRWD1-IT1	0.559	6.13E-35
PRR4	AC007448.2	0.605	4.22E-42
PRR4	AC026401.1	0.578	1.06E-37
PRR4	CFLAR-AS1	0.643	5.85E-49
PRR4	AC004217.2	0.571	1.11E-36
PRR4	AP001970.1	0.586	4.89E-39
PRR4	AC006355.2	0.524	3.74E-30
PRR4	AL354733.2	0.655	2.42E-51
PRR4	AL513185.1	0.569	2.51E-36
PRR4	AC008395.1	0.598	5.32E-41
PRR4	AC102945.1	0.539	4.19E-32
PRR4	SND1-IT1	0.628	3.42E-46
PRR4	AC090559.2	0.546	4.38E-33
PRR4	AP003467.1	0.61	5.60E-43
PRR4	AC007496.2	0.561	2.95E-35
PRR4	AC103702.1	0.602	1.33E-41
PRR4	RERG-AS1	0.525	2.93E-30
PRR4	AL157938.2	0.645	2.37E-49
PRR4	CR936218.1	0.612	3.25E-43
PRR4	MCM8-AS1	0.588	2.75E-39
PRR4	AC068790.7	0.532	3.49E-31
PRR4	AL512366.1	0.519	1.83E-29
PRR4	AC010285.2	0.51	1.93E-28
PRR4	AC026124.1	0.569	1.99E-36
PRR4	AC129510.1	0.508	4.30E-28
PRR4	AC009032.1	0.573	6.67E-37
PRR4	RBM5-AS1	0.567	4.32E-36
PRR4	TXNDC12-AS1	0.506	7.31E-28
PRR4	AC005828.2	0.605	4.17E-42
PRR4	SNHG22	0.538	5.23E-32
PRR4	FGF12-AS1	0.564	1.24E-35
PRR4	AL117381.1	0.522	6.95E-30
PRR4	CXXC4-AS1	0.556	1.73E-34
PRR4	AC022613.3	0.65	2.65E-50
PRR4	AC005828.1	0.613	2.08E-43
PRR4	AC007390.2	0.61	5.34E-43
PRR4	AC111194.2	0.574	4.08E-37
PRR4	AC090220.1	0.63	1.82E-46
PRR4	AL391421.1	0.546	4.41E-33
PRR4	AC018761.3	0.51	2.22E-28
PRR4	AL139193.2	0.603	8.27E-42
PRR4	AC107021.1	0.536	9.20E-32
PRR4	SHANK2-AS2	0.513	9.46E-29

PRR4	AC016405.1	0.587	4.02E-39
PRR4	ITPKB-AS1	0.574	4.06E-37
PRR4	AC013549.3	0.558	9.11E-35
PRR4	AC087501.3	0.608	1.21E-42
PRR4	AC092168.2	0.505	8.09E-28
PRR4	AC025576.2	0.532	3.15E-31
PRR4	AL136311.1	0.686	5.15E-58
PRR4	AC092127.1	0.592	5.43E-40
PRR4	AL354794.1	0.54	2.86E-32
PRR4	AC005104.1	0.541	2.22E-32
PRR4	AC027801.5	0.507	4.88E-28
PRR4	AC018682.1	0.548	2.48E-33
PRR4	AC073964.1	0.584	1.21E-38
PRR4	AC004466.2	0.502	1.79E-27
PRR4	ITGB5-AS1	0.592	5.66E-40
PRR4	AC107896.1	0.563	1.51E-35
PRR4	AP002892.1	0.607	2.38E-42
PRR4	AL360091.1	0.553	4.40E-34
PRR4	FP700111.1	0.519	1.84E-29
PRR4	AC024475.1	0.567	4.26E-36
PRR4	AC110491.2	0.601	2.22E-41
PRR4	AC087260.1	0.529	7.70E-31
PRR4	AC007731.4	0.588	2.50E-39
PRR4	AP003043.1	0.529	7.58E-31
PRR4	AC023034.1	0.54	3.07E-32
PRR4	AC012379.1	0.58	4.20E-38
PRR4	AC008700.1	0.54	2.73E-32
PRR4	VWC2L-IT1	0.51	2.43E-28
PRR4	AL389885.1	0.523	4.54E-30
PRR4	AL022322.2	0.598	6.01E-41
PRR4	AC009754.1	0.532	3.02E-31
PRR4	AC012170.2	0.531	4.37E-31
PRR4	AL157938.3	0.635	1.57E-47
PRR4	AC007114.2	0.607	2.04E-42
PRR4	SPATA13-AS1	0.574	3.52E-37
PRR4	AC066613.2	0.582	2.46E-38
PRR4	AP006333.2	0.53	6.47E-31
PRR4	AC092868.2	0.581	3.92E-38
PRR4	AL583808.2	0.545	6.82E-33
PRR4	AL499616.1	0.556	2.04E-34
PRR4	AL355613.1	0.586	6.31E-39
PRR4	AC007342.1	0.559	6.05E-35
PRR4	AC010761.3	0.53	6.95E-31
PRR4	AL592301.1	0.535	1.31E-31

PRR4	AL138916.2	0.521	8.33E-30
PRR4	AL133284.1	0.609	7.52E-43
PRR4	LINC01772	0.587	3.67E-39
PRR4	AC066613.1	0.542	1.66E-32
PRR4	WWC3-AS1	0.518	1.89E-29
PRR4	AF131216.1	0.522	5.94E-30
PRR4	AC120498.1	0.517	2.53E-29
PRR4	ASH1L-IT1	0.613	1.62E-43
PRR4	AL589863.1	0.633	4.77E-47
PRR4	AL049869.3	0.554	3.07E-34
PRR4	AC113146.1	0.56	4.26E-35
PRR4	AC073326.1	0.509	2.77E-28
PRR4	GSN-AS1	0.617	3.65E-44
PRR4	AL669841.1	0.559	7.00E-35
PRR4	AL034349.1	0.527	1.69E-30
PRR4	Z83843.1	0.656	1.48E-51
PRR4	AC096721.1	0.629	2.54E-46
PRR4	AC010976.1	0.705	1.60E-62
PRR4	AC010401.2	0.516	4.32E-29
PRR4	AC090971.2	0.556	1.71E-34
PRR4	AL117341.1	0.623	2.55E-45
PRR4	AC002558.3	0.61	6.77E-43
PRR4	IQCJ-SCHIP1-AS1	0.584	9.68E-39
PRR4	SEC62-AS1	0.576	1.80E-37
PRR4	AC025430.1	0.641	1.52E-48
PRR4	ARHGAP26-IT1	0.518	2.37E-29
PRR4	AC104827.1	0.624	1.87E-45
PRR4	AL358075.1	0.557	1.24E-34
PRR4	AC104316.1	0.538	5.72E-32
PRR4	AC105150.1	0.604	5.82E-42
PRR4	AP002490.1	0.55	1.18E-33
PRR4	AC104964.2	0.603	1.09E-41
PRR4	AC087284.1	0.526	2.12E-30
PRR4	AC124319.2	0.563	1.58E-35
PRR4	AL138808.1	0.557	1.22E-34
PRR4	AC022447.1	0.536	1.11E-31
PRR4	AC016493.1	0.626	1.06E-45
PRR4	AL355483.3	0.505	8.43E-28
PRR4	AL357093.1	0.522	6.56E-30
PRR4	AL669831.5	0.514	7.58E-29
PRR4	AC053513.1	0.622	5.32E-45
PRR4	AC105760.1	0.607	2.27E-42
PRR4	AL139118.1	0.528	1.31E-30
PRR4	AL021937.3	0.509	2.97E-28

PRR4	AC007601.1	0.529	7.98E-31
PRR4	CACNA1C-AS3	0.617	4.11E-44
PRR4	AL049840.1	0.559	7.09E-35
PRR4	AL133227.1	0.54	3.13E-32
PRR4	AC025038.1	0.577	1.63E-37
PRR4	LINC01796	0.531	4.46E-31
PRR4	LPP-AS1	0.6	2.50E-41
PRR4	AC007688.3	0.562	2.13E-35
PRR4	AC006206.1	0.539	4.28E-32
PRR4	AC132192.1	0.575	2.85E-37
PRR4	AC016866.2	0.537	8.34E-32
PRR4	RNF216-IT1	0.51	2.33E-28
PRR4	AC087439.1	0.525	2.43E-30
PRR4	AC046158.1	0.615	6.73E-44
PRR4	AC024270.5	0.536	1.06E-31
PRR4	AP000462.2	0.527	1.38E-30
PRR4	AL050343.1	0.535	1.29E-31
PRR4	AC005546.1	0.528	1.26E-30
PRR4	AL031320.2	0.571	1.29E-36
PRR4	AC090617.5	0.55	1.13E-33
PRR4	AP001020.3	0.651	1.31E-50
PRR4	AC074366.1	0.531	4.75E-31
PRR4	AC124283.3	0.538	6.31E-32
PRR4	AL136982.3	0.635	2.15E-47
PRR4	AC099811.5	0.585	7.05E-39
PRR4	SMYD3-IT1	0.595	1.83E-40
PRR4	AC067817.1	0.556	1.57E-34
PRR4	POC1B-AS1	0.52	1.38E-29
PRR4	DOCK9-AS1	0.64	2.25E-48
PRR4	AC016245.1	0.586	5.86E-39
PRR4	RNASEH2B-AS1	0.549	1.61E-33
PRR4	AL121759.1	0.537	7.24E-32
PRR4	AL590729.1	0.528	1.28E-30
PRR4	AC092653.1	0.562	2.35E-35
PRR4	AC007431.2	0.624	1.89E-45
PRR4	AC096741.2	0.625	1.61E-45
PRR4	AP003696.1	0.533	2.89E-31
PRR4	AL356481.2	0.579	6.71E-38
PRR4	ITCH-AS1	0.602	1.36E-41
PRR4	NFIA-AS1	0.624	1.68E-45
PRR4	AC005096.1	0.587	3.36E-39
PRR4	AL365436.2	0.55	1.43E-33
PRR4	AC005070.3	0.628	3.85E-46
PRR4	AC040934.1	0.541	2.11E-32

PRR4	AP000897.2	0.578	1.03E-37
PRR4	TCF7L1-IT1	0.517	2.93E-29
PRR4	AL158834.1	0.625	1.49E-45
PRR4	AC087749.2	0.591	8.66E-40
PRR4	FOXN3-AS2	0.563	1.70E-35
PRR4	AC138058.1	0.59	1.06E-39
PRR4	AC110792.3	0.62	9.82E-45
PRR4	AL132989.1	0.602	1.19E-41
PRR4	B4GALT4-AS1	0.503	1.45E-27
PRR4	AC012291.1	0.575	3.12E-37
PRR4	AC092800.1	0.596	1.23E-40
PRR4	AL158071.4	0.51	2.32E-28
PRR4	AC018552.4	0.523	5.67E-30
PRR4	AC023794.1	0.529	9.66E-31
PRR4	KIZ-AS1	0.574	3.62E-37
PRR4	TRMT2B-AS1	0.528	1.07E-30
PRR4	AC111170.1	0.608	1.48E-42
PRR4	AC007378.1	0.564	1.21E-35
PRR4	AC078962.3	0.551	8.48E-34
PRR4	AC138150.1	0.592	6.02E-40
PRR4	AC018410.1	0.568	2.75E-36
PRR4	AC125793.1	0.511	1.45E-28
PRR4	AC009686.1	0.553	4.01E-34
PRR4	AL162386.2	0.554	3.74E-34
PRR4	FOXP1-AS1	0.564	1.45E-35
PRR4	AC022173.1	0.601	1.91E-41
PRR4	CARS-AS1	0.609	9.68E-43
PRR4	AL160163.1	0.54	3.10E-32
PRR4	AC002558.2	0.602	1.25E-41
PRR4	LNX1-AS1	0.565	8.21E-36
PRR4	AC019130.1	0.605	3.87E-42
PRR4	AP005432.1	0.501	2.60E-27
PRR4	AF131215.2	0.582	2.00E-38
PRR4	AC009480.1	0.606	3.04E-42
PRR4	DLEU2L	0.558	9.70E-35
PRR4	AC100827.4	0.533	2.92E-31
PRR4	FAM53B-AS1	0.663	5.37E-53
PRR4	AC091231.1	0.614	1.10E-43
PRR4	AC100774.1	0.645	2.75E-49
PRR4	AC139792.1	0.622	4.69E-45
PRR4	AC107982.2	0.511	1.65E-28
PRR4	AL157392.1	0.506	7.08E-28
PRR4	AC017101.1	0.602	1.46E-41
PRR4	AL139193.1	0.577	1.61E-37

PRR4	AC002347.2	0.58	5.23E-38
PRR4	AL161757.2	0.576	1.69E-37
PRR4	AL133243.3	0.506	6.48E-28
PRR4	AC007673.1	0.584	9.60E-39
PRR4	MAST4-IT1	0.566	5.82E-36
PRR4	AF178030.1	0.513	9.79E-29
PRR4	AL662889.1	0.565	9.48E-36
PRR4	PHACTR2-AS1	0.615	9.28E-44
PRR4	NAV2-AS5	0.538	6.13E-32
PRR4	AC100830.2	0.501	2.56E-27
PRR4	AC021237.1	0.577	1.19E-37
PRR4	AC022973.2	0.613	1.66E-43
PRR4	FOXP1-IT1	0.627	5.55E-46
PRR4	AC013356.3	0.545	5.82E-33
PRR4	AC092839.2	0.543	1.26E-32
PRR4	AC037487.1	0.568	2.84E-36
PRR4	AL450226.1	0.621	8.33E-45
PRR4	AL118556.1	0.523	4.69E-30
PRR4	AP003397.1	0.603	1.07E-41
PRR4	LINC01209	0.51	2.23E-28
PRR4	AC025043.1	0.572	8.16E-37
PRR4	AC104984.3	0.54	3.03E-32
PRR4	AL360268.1	0.581	3.49E-38
PRR4	LZTS1-AS1	0.585	8.44E-39
PRR4	AC020913.3	0.526	2.22E-30
PRR4	AL161909.2	0.591	9.29E-40
PRR4	AL354893.2	0.525	2.84E-30
PRR4	AC087893.1	0.526	1.90E-30
PRR4	AL031428.1	0.572	7.50E-37
PRR4	AL356805.1	0.602	1.30E-41
PRR4	LINC01355	0.506	6.95E-28
PRR4	RHOA-IT1	0.587	4.05E-39
PRR4	AC092669.1	0.538	4.71E-32
PRR4	FP325330.3	0.531	4.90E-31
PRR4	AC099313.1	0.528	1.24E-30
PRR4	AC015845.1	0.635	1.93E-47
PRR4	COL18A1-AS1	0.574	4.34E-37
PRR4	AC087721.1	0.525	3.16E-30
PRR4	AC022447.3	0.501	2.34E-27
PRR4	AC048380.1	0.545	6.09E-33
PRR4	AC078778.2	0.608	1.11E-42
PRR4	AC007598.1	0.61	6.48E-43
PRR4	AC007619.1	0.604	6.88E-42
PRR4	AC005344.1	0.614	1.08E-43

PRR4	AC015971.1	0.641	1.11E-48
PRR4	AC022537.1	0.522	6.84E-30
PRR4	AC092067.1	0.581	3.27E-38
PRR4	AC023830.1	0.564	1.41E-35
PRR4	AL138820.1	0.507	5.15E-28
PRR4	CTD-2350J17.1	0.61	6.88E-43
PRR4	AC011379.1	0.628	3.81E-46
PRR4	BTBD9-AS1	0.584	1.21E-38
PRR4	AC010210.1	0.534	1.79E-31
PRR4	AC018410.2	0.515	5.27E-29
PRR4	AL355075.4	0.588	2.86E-39
PRR4	AC009095.1	0.598	5.94E-41
PRR4	AC005726.3	0.647	9.09E-50
PRR4	AL162390.1	0.555	2.79E-34
PRR4	AC018878.1	0.618	2.66E-44
PRR4	AC068790.4	0.517	2.75E-29
PRR4	AL162171.3	0.578	9.01E-38
PRR4	FO393419.2	0.598	6.13E-41
PRR4	AL355336.1	0.613	1.63E-43
PRR4	AC023794.4	0.595	2.09E-40
PRR4	CACNA1C-AS4	0.622	5.51E-45
PRR4	AC112512.1	0.539	4.18E-32
PRR4	DENND6A-AS1	0.517	3.01E-29
PRR4	SYNJ2-IT1	0.587	4.53E-39
PRR4	AP003168.2	0.614	1.15E-43
PRR4	AC021739.4	0.606	3.04E-42
PRR4	AC055811.3	0.602	1.19E-41
PRR4	AC068790.2	0.578	9.94E-38
PRR4	AC009119.3	0.524	3.76E-30
PRR4	AC002064.2	0.547	3.47E-33
PRR4	AC016644.1	0.608	1.31E-42
PRR4	AC116903.2	0.526	2.13E-30
PRR4	AC068790.6	0.559	7.72E-35
PRR4	LINC02160	0.536	9.65E-32
PRR4	AL512656.1	0.552	6.15E-34
PRR4	ZMYM4-AS1	0.608	1.62E-42
PRR4	LINC00624	0.509	2.61E-28
PRR4	AP001442.1	0.614	1.26E-43
PRR4	AC016027.3	0.535	1.40E-31
PRR4	AC025034.1	0.597	9.04E-41
PRR4	AC020612.3	0.604	7.16E-42
PRR4	AC131571.1	0.521	8.41E-30
PRR4	AL355916.2	0.529	7.45E-31
PRR4	AC116535.2	0.528	1.08E-30

PRR4	AC078883.2	0.599	4.04E-41
PRR4	AL096869.1	0.56	4.79E-35
PRR4	AC060809.1	0.571	1.17E-36
PRR4	SHANK2-AS1	0.539	4.36E-32
PRR4	AC008243.1	0.529	7.95E-31
PRR4	AC007362.1	0.52	1.21E-29
PRR4	AC055733.2	0.558	9.58E-35
PRR4	AL513523.3	0.52	1.23E-29
PRR4	AL591623.1	0.623	3.49E-45
PRR4	AL353804.2	0.66	2.36E-52
PRR4	ZBTB20-AS3	0.615	9.74E-44
PRR4	SEMA5A-AS1	0.563	1.81E-35
PRR4	AC008264.2	0.601	1.97E-41
PRR4	AC068790.3	0.521	9.38E-30
PRR4	AC022558.1	0.556	1.98E-34
PRR4	AL035588.1	0.586	6.40E-39
PRR4	AC087241.4	0.584	1.00E-38
PRR4	MYLK-AS2	0.502	2.25E-27
PRR4	AC009464.1	0.574	4.53E-37
PRR4	AC069542.1	0.547	3.16E-33
PRR4	Z99289.3	0.603	8.86E-42
PRR4	AC007599.2	0.619	1.54E-44
PRR4	AC010307.3	0.548	2.31E-33
PRR4	AC087761.1	0.572	7.71E-37
PRR4	AC005757.1	0.587	3.99E-39
PRR4	AC004593.1	0.636	1.33E-47
PRR4	FTX	0.661	1.76E-52
PRR4	AC118555.1	0.569	2.27E-36
PRR4	AC012409.2	0.548	2.27E-33
PRR4	AC007496.1	0.574	4.09E-37
PRR4	AC072022.2	0.536	1.08E-31
PRR4	MALAT1	0.553	5.21E-34
PRR4	MAGI1-AS1	0.622	4.55E-45
PRR4	LINC01878	0.548	2.23E-33
PRR4	AC108063.1	0.553	4.29E-34
PRR4	BICRA-AS1	0.572	7.60E-37
PRR4	AC009303.3	0.567	5.22E-36
PRR4	MYCBP2-AS1	0.616	6.70E-44
PRR4	AC073569.3	0.597	1.02E-40
PRR4	AP000941.1	0.609	9.27E-43
PRR4	AC011939.2	0.594	3.15E-40
PRR4	AL356022.1	0.502	1.93E-27
PRR4	DLEU1-AS1	0.576	2.20E-37
PRR4	AC087294.1	0.563	1.46E-35

PRR4	AC136424.1	0.611	4.09E-43
PRR4	MYCBP2-AS2	0.596	1.21E-40
PRR4	AC090802.1	0.538	5.38E-32
PRR4	AL390208.1	0.561	3.45E-35
PRR4	AL645504.1	0.515	5.24E-29
PRR4	AC018462.1	0.582	2.32E-38
PRR4	AC138956.2	0.526	2.41E-30
PRR4	ACAP2-IT1	0.585	9.36E-39
PRR4	AL031594.1	0.641	1.21E-48
PRR4	AC092574.2	0.67	1.48E-54
PRR4	STARD13-AS	0.567	4.13E-36
PRR4	ASTN2-AS1	0.616	5.72E-44
PRR4	SPRY4-IT1	0.536	9.90E-32
PRR4	AC091185.1	0.544	7.76E-33
PRR4	AC005072.1	0.616	5.91E-44
PRR4	AL445223.1	0.622	4.44E-45
PRR4	MIR381HG	0.524	3.59E-30
PRR4	PCCA-AS1	0.641	1.31E-48
PRR4	AC103739.1	0.526	1.90E-30
PRR4	AC064874.1	0.637	9.06E-48
PRR4	AL513412.1	0.595	2.14E-40
PRR4	AC008649.2	0.552	7.32E-34
PRR4	AP001469.1	0.523	5.62E-30
PRR4	AC079203.2	0.532	3.44E-31
PRR4	AC022400.2	0.615	9.22E-44
PRR4	TTC21B-AS1	0.623	3.37E-45
PRR4	DAPK1-IT1	0.621	7.00E-45
PRR4	AC211433.1	0.566	5.89E-36
PRR4	AC008147.1	0.524	4.36E-30
PRR4	AL139353.2	0.517	2.98E-29
PRR4	ITCH-IT1	0.55	1.23E-33
PRR4	AP005131.1	0.532	3.14E-31
PRR4	AL136979.1	0.573	6.19E-37
PRR4	CFAP44-AS1	0.519	1.46E-29
PRR4	AC130895.1	0.624	2.30E-45
PRR4	CLYBL-AS1	0.503	1.60E-27
PRR4	AC105339.2	0.556	1.92E-34
PRR4	ELMO1-AS1	0.522	7.81E-30
PRR4	TH2LCRR	0.535	1.57E-31
PRR4	AC007000.3	0.501	2.73E-27
PRR4	FBXW7-AS1	0.582	2.66E-38
PRR4	AL136988.2	0.601	2.18E-41
PRR4	AL035634.1	0.6	2.71E-41
PRR4	AC114737.1	0.582	2.71E-38

PRR4	AL365356.1	0.642	7.36E-49
PRR4	AC078795.1	0.611	4.91E-43
PRR4	AC025031.4	0.654	3.72E-51
PRR4	AC009120.4	0.511	1.81E-28
PRR4	AC009054.1	0.537	7.54E-32
PRR4	UST-AS1	0.537	6.58E-32
PRR4	AC211476.2	0.523	5.01E-30
PRR4	AC005746.1	0.554	3.36E-34
PRR4	AC012613.2	0.576	2.26E-37
PRR4	AC010776.1	0.566	6.83E-36
PRR4	AC017006.2	0.51	2.19E-28
PRR4	AL078581.3	0.547	3.73E-33
PRR4	AC018946.1	0.594	3.07E-40
PRR4	AC011586.1	0.509	3.14E-28
PRR4	AC016229.1	0.555	2.26E-34
PRR4	C9orf41-AS1	0.618	2.43E-44
PRR4	AL157871.5	0.599	4.23E-41
PRR4	AC108102.1	0.632	6.28E-47
PRR4	AC134407.1	0.59	1.28E-39
PRR4	AC027018.1	0.649	3.74E-50
PRR4	AC087362.1	0.556	2.03E-34
PRR4	AC020661.3	0.632	6.45E-47
PRR4	NAALADL2-AS3	0.559	6.08E-35
PRR4	AL359095.1	0.592	5.11E-40
PRR4	AC069257.1	0.522	7.70E-30
PRR4	TTLL7-IT1	0.514	6.83E-29
PRR4	NPTN-IT1	0.56	4.27E-35
PRR4	AC080162.1	0.604	7.46E-42
PRR4	PLCE1-AS2	0.541	2.17E-32
PRR4	AC027514.2	0.615	9.73E-44
PRR4	AC016831.5	0.607	2.16E-42
PRR4	AC011284.1	0.538	5.24E-32
PRR4	PRICKLE2-AS1	0.555	2.87E-34
PRR4	ERI3-IT1	0.578	1.13E-37
PRR4	AC138028.1	0.54	2.62E-32
PRR4	AC004672.1	0.643	6.59E-49
PRR4	AC079866.2	0.589	1.85E-39
PRR4	KDM5C-IT1	0.588	3.17E-39
PRR4	AC027796.5	0.533	2.63E-31
PRR4	AC004672.2	0.589	1.58E-39
PRR4	AC068025.2	0.566	7.01E-36
PRR4	AL035448.1	0.612	3.14E-43
PRR4	AC008592.2	0.52	1.27E-29
PRR4	AL360169.2	0.634	3.53E-47

PRR4	LINC01362	0.577	1.19E-37
PRR4	AC131971.1	0.649	3.82E-50
PRR4	AC012157.1	0.54	2.97E-32
PRR4	RC3H1-IT1	0.598	5.76E-41
PRR4	AC079915.1	0.622	4.65E-45
PRR4	GRK5-IT1	0.541	1.93E-32
PRR4	AC103876.1	0.566	6.29E-36
PRR4	AC008770.4	0.523	4.51E-30
PRR4	AP001178.2	0.586	6.49E-39
PRR4	AC005920.2	0.624	2.19E-45
PRR4	Z99127.1	0.552	5.94E-34
PRR4	PLCB1-IT1	0.63	1.38E-46
PRR4	AC069079.1	0.548	2.24E-33
PRR4	AC130650.1	0.573	4.94E-37
PRR4	AL596247.1	0.533	2.39E-31
PRR4	AC011933.4	0.539	3.70E-32
PRR4	AL591721.1	0.505	8.78E-28
PRR4	RMRP	0.583	1.87E-38
PRR4	AL049840.5	0.505	9.18E-28
PRR4	AL136115.2	0.607	2.20E-42
PRR4	AL161644.1	0.598	5.60E-41
PRR4	AC020978.2	0.511	1.49E-28
PRR4	AC008543.5	0.503	1.56E-27
PRR4	AC108463.3	0.613	1.55E-43
PRR4	TBC1D22A-AS1	0.628	3.40E-46
PRR4	LINC01803	0.539	3.68E-32
PRR4	AL133260.2	0.578	9.19E-38
PRR4	U73169.1	0.602	1.30E-41
PRR4	AL031666.3	0.568	2.94E-36
PRR4	AC135782.3	0.52	1.39E-29
PRR4	AC091059.1	0.529	8.19E-31
PRR4	AL022097.1	0.576	1.78E-37
PRR4	AL162274.1	0.519	1.48E-29
PRR4	AC037487.2	0.615	8.62E-44
PRR4	AC011753.2	0.613	1.77E-43
PRR4	Z95331.1	0.686	5.37E-58
PRR4	AC103739.2	0.597	9.83E-41
PRR4	CSNK1G2-AS1	0.585	8.07E-39
PRR4	AC114760.2	0.551	8.12E-34
PRR4	AC079385.1	0.518	2.18E-29
PRR4	AL450344.2	0.596	1.29E-40
PRR4	ANKRD44-IT1	0.55	1.21E-33
PRR4	AC005695.2	0.599	4.14E-41
PRR4	AC020661.4	0.592	6.12E-40

PRR4	AL096869.2	0.574	4.17E-37
PRR4	FAM13A-AS1	0.517	3.00E-29
PRR4	WASHC5-AS1	0.595	1.69E-40
PRR4	LYST-AS1	0.558	8.68E-35
PRR4	HMBOX1-IT1	0.558	9.31E-35
PRR4	AL031717.1	0.511	1.55E-28
PRR4	AL360091.2	0.607	1.97E-42
PRR4	AL132989.2	0.725	8.09E-68
PRR4	AC009884.1	0.541	2.41E-32
PRR4	AC092802.3	0.544	8.71E-33
PRR4	AL513365.2	0.516	4.33E-29
PRR4	AL162724.1	0.567	5.11E-36
PRR4	AC024145.1	0.569	2.63E-36
PRR4	AC106037.1	0.501	2.71E-27
PRR4	AC100778.1	0.534	1.63E-31
PRR4	FP325332.1	0.642	9.01E-49
PRR4	AC092338.1	0.537	7.19E-32
PRR4	AC244093.3	0.538	5.63E-32
PRR4	AL807757.1	0.615	6.90E-44
PRR4	AC090971.5	0.502	1.99E-27
PRR4	AMMECR1-IT1	0.613	2.13E-43
PRR4	AL133230.1	0.591	7.95E-40
PRR4	AC007601.2	0.566	5.39E-36
PRR4	AC091053.2	0.629	2.09E-46
PRR4	AC006141.1	0.632	5.79E-47
PRR4	AL592430.1	0.572	8.02E-37
PRR4	AC087501.1	0.618	1.98E-44
PRR4	RDH10-AS1	0.549	1.68E-33
PRR4	AC003101.1	0.559	6.75E-35
PRR4	AC022001.2	0.554	3.69E-34
PRR4	AC100763.1	0.627	6.58E-46
PRR4	AC073592.1	0.509	2.53E-28
PRR4	AC096733.1	0.628	3.64E-46
PRR4	AC005520.3	0.611	3.58E-43
PRR4	AC008731.1	0.53	5.58E-31
PRR4	AC005480.1	0.627	5.61E-46
PRR4	XIAP-AS1	0.632	6.44E-47
PRR4	AC007431.1	0.581	3.06E-38
PRR4	AL355075.2	0.504	1.19E-27
PRR4	AC107909.1	0.622	3.91E-45
PRR4	AP000919.2	0.614	1.49E-43
PRR4	AL357093.2	0.625	1.49E-45
PRR4	AC138393.3	0.588	2.74E-39
PRR4	AL121895.1	0.572	7.39E-37

PRR4	AC012100.2	0.541	2.22E-32
PRR4	AL050331.3	0.514	7.81E-29
PRR4	AC116096.1	0.536	1.09E-31
PRR4	CASC17	0.506	6.39E-28
PRR4	MKNK1-AS1	0.559	7.16E-35
PRR4	AC000120.1	0.647	7.65E-50
PRR4	AP002884.5	0.553	4.36E-34
PRR4	LINC00102	0.59	1.16E-39
PRR4	AC008937.2	0.608	1.12E-42
PRR4	AL583722.4	0.54	3.27E-32
PRR4	MANEA-AS1	0.518	2.06E-29
PRR4	AC011466.2	0.548	2.42E-33
PRR4	Z93022.1	0.553	4.97E-34
PRR4	AL157832.2	0.555	2.10E-34
PRR4	AC130651.2	0.581	3.35E-38
PRR4	ARHGEF3-AS1	0.623	2.95E-45
PRR4	AC096947.1	0.588	2.43E-39
PRR4	AC004594.1	0.557	1.22E-34
PRR4	AL133481.1	0.621	5.85E-45
PRR4	AL442071.1	0.548	2.56E-33
PRR4	AL023581.1	0.606	3.02E-42
PRR4	AL121652.1	0.605	4.14E-42
PRR4	AC099811.1	0.59	1.35E-39
PRR4	AC008467.1	0.505	8.66E-28
PRR4	AC011092.2	0.548	2.11E-33
PRR4	AC008750.5	0.558	9.45E-35
PRR4	INO80-AS1	0.528	1.14E-30
PRR4	AC092608.1	0.515	5.55E-29
PRR4	BIRC6-AS1	0.632	7.11E-47
PRR4	AC013286.1	0.628	4.32E-46
PRR4	WWOX-AS1	0.599	4.07E-41
PRR4	LIMS1-AS1	0.532	3.10E-31
PRR4	SMAD1-AS1	0.592	6.34E-40
PRR4	MAMDC2-AS1	0.553	4.31E-34
PRR4	AC015849.4	0.595	1.83E-40
PRR4	AC087521.2	0.608	1.14E-42
PRR4	AC100800.1	0.562	2.74E-35
PRR4	AC011595.1	0.567	4.05E-36
PRR4	AC012404.1	0.541	1.93E-32
PRR4	LARGE-IT1	0.557	1.45E-34
PRR4	AL133343.3	0.504	1.02E-27
PRR4	AC097382.1	0.541	2.16E-32
PRR4	SNAP47-AS1	0.549	1.48E-33
PRR4	AC007598.2	0.57	1.86E-36

PRR4	TMLHE-AS1	0.535	1.37E-31
PRR4	EFCAB14-AS1	0.551	7.92E-34
PRR4	AC024619.3	0.512	1.34E-28
PRR4	AL353801.2	0.555	2.66E-34
PRR4	AL049637.1	0.633	4.07E-47
PRR4	BACH1-IT3	0.554	3.76E-34
PRR4	AC114763.2	0.642	9.53E-49
PRR4	RASA2-IT1	0.552	6.77E-34
PRR4	AL354726.1	0.6	2.47E-41
PRR4	AC131934.1	0.565	9.44E-36
PRR4	AL161907.1	0.515	4.52E-29
PRR4	WDFY3-AS1	0.513	8.59E-29
PRR4	AC023632.5	0.513	9.72E-29
PRR4	AC008474.1	0.539	4.45E-32
PRR4	AC055764.1	0.534	1.97E-31
PRR4	AC069208.1	0.528	1.31E-30
PRR4	MAGI1-IT1	0.552	6.26E-34
PRR4	AC010320.4	0.676	7.02E-56
PRR4	AL445465.1	0.521	8.78E-30
PRR4	AC055811.1	0.553	5.46E-34
PRR4	AP000654.1	0.575	2.58E-37
PRR4	ALG9-IT1	0.613	1.72E-43
PRR4	AL360091.3	0.555	2.17E-34
PRR4	AC013652.2	0.565	8.50E-36
PRR4	AC092794.2	0.531	5.29E-31
PRR4	AC104232.2	0.568	3.18E-36
PRR4	AC116158.1	0.599	4.77E-41
PRR4	AC087286.4	0.587	3.23E-39
PRR4	AC079035.1	0.598	6.96E-41
PRR4	AC092892.1	0.536	9.55E-32
PRR4	RABGAP1L-IT1	0.522	5.93E-30
PRR4	N4BP2L2-IT2	0.567	4.98E-36
PRR4	E2F3-IT1	0.552	6.72E-34
PRR4	AL390774.2	0.616	4.94E-44
PRR4	AC016910.1	0.61	6.55E-43
PRR4	AC092794.1	0.501	2.88E-27
PRR4	LINC02443	0.533	2.35E-31
PRR4	AC008737.1	0.508	4.06E-28
PRR4	AL357568.1	0.509	3.05E-28
PRR4	AC068389.4	0.632	6.43E-47
PRR4	OPA1-AS1	0.655	2.00E-51
PRR4	AL031772.1	0.637	7.45E-48
PRR4	AL136984.1	0.66	2.58E-52
PRR4	AC024267.5	0.632	5.84E-47

PRR4	ALG13-AS1	0.52	1.19E-29
PRR4	THRA1/BTR	0.558	1.02E-34
PRR4	AC073487.1	0.586	5.52E-39
PRR4	AC022272.1	0.551	8.95E-34
PRR4	AC027228.1	0.59	1.20E-39
PRR4	AC016292.1	0.564	1.11E-35
PRR4	AC098869.2	0.551	1.02E-33
PRR4	AC096558.2	0.527	1.41E-30
PRR4	AL035071.2	0.593	4.67E-40
PRR4	AC144521.1	0.516	4.38E-29
PRR4	AC023510.1	0.549	1.70E-33
PRR4	AL606534.2	0.513	9.24E-29
PRR4	ATP11A-AS1	0.523	5.08E-30
PRR4	AL451062.1	0.519	1.51E-29
PRR4	PLS1-AS1	0.537	8.12E-32
PRR4	AL133406.2	0.524	4.18E-30
PRR4	AC015563.2	0.531	5.04E-31
PRR4	AC009142.1	0.576	2.20E-37
PRR4	AC007533.1	0.599	3.76E-41
PRR4	ZBTB20-AS4	0.579	7.17E-38
PRR4	INTS9-AS1	0.534	1.76E-31
PRR4	AC090213.1	0.56	5.50E-35
PRR4	AC090739.1	0.525	2.56E-30
PRR4	AC008957.2	0.542	1.45E-32
PRR4	AL513327.1	0.569	1.98E-36
PRR4	AC127024.4	0.515	4.70E-29
PRR4	AC097103.1	0.508	3.44E-28
PRR4	MIR181A1HG	0.507	4.50E-28
PRR4	AL450306.1	0.569	1.91E-36
PRR4	AL360093.1	0.602	1.38E-41
PRR4	AC022400.5	0.506	5.93E-28
PRR4	AC111170.2	0.541	1.96E-32
PRR4	AL445931.1	0.526	1.90E-30
PRR4	AC103810.2	0.566	6.64E-36
PRR4	AL139407.1	0.626	7.86E-46
PRR4	RSF1-IT1	0.501	2.81E-27
PRR4	AC090617.1	0.591	1.02E-39
PRR4	ZRANB2-AS1	0.518	2.11E-29
PRR4	AL118558.1	0.522	7.59E-30
PRR4	AC044840.1	0.531	4.97E-31
PRR4	AC011933.2	0.629	2.07E-46
PRR4	AC092171.1	0.521	9.02E-30
PRR4	AC084291.1	0.522	7.84E-30
PRR4	CLIP1-AS1	0.615	8.04E-44

PRR4	MGC15885	0.575	2.58E-37
PRR4	ARAP1-AS2	0.595	1.63E-40
PRR4	AL096855.1	0.514	7.73E-29
PRR4	AL451047.1	0.611	4.58E-43
PRR4	AC087620.1	0.6	3.07E-41
PRR4	AC093019.2	0.582	2.18E-38
PRR4	SPAG5-AS1	0.504	1.29E-27
PRR4	AC010632.2	0.543	1.12E-32
PRR4	ATG10-AS1	0.551	9.78E-34
PRR4	LINC01359	0.607	2.35E-42
PRR4	AL450344.1	0.603	9.28E-42
PRR4	AC104958.1	0.596	1.34E-40
PRR4	AL049820.1	0.611	3.57E-43
PRR4	AL135838.1	0.567	4.95E-36
PRR4	AC022540.1	0.62	1.31E-44
PRR4	AP005899.1	0.501	2.33E-27
PRR4	AL162171.2	0.689	1.26E-58
PRR4	AC008781.2	0.63	1.67E-46
PRR4	AC012254.3	0.643	6.14E-49
PRR4	LINC02465	0.526	2.08E-30
PRR4	PSMD6-AS2	0.548	2.35E-33
PRR4	AC095033.1	0.532	3.67E-31
PRR4	AC024267.4	0.522	6.69E-30
PRR4	FAR1-IT1	0.515	5.37E-29
PRR4	AP002884.4	0.585	7.02E-39
PRR4	AL365258.1	0.615	7.95E-44
PRR4	AC013564.1	0.633	5.44E-47
PRR4	AC007906.1	0.528	1.16E-30
PRR4	AC127024.3	0.59	1.42E-39
PRR4	AC063965.1	0.634	2.57E-47
PRR4	AP001107.1	0.507	5.14E-28
PRR4	AC108727.1	0.528	1.07E-30
PRR4	AC079336.3	0.552	7.46E-34
PRR4	AC012494.1	0.597	9.13E-41
PRR4	AC008147.2	0.63	2.03E-46
PRR4	AL353804.1	0.512	1.29E-28
PRR4	SHANK2-AS3	0.505	8.94E-28
PRR4	TBL1XR1-AS1	0.52	1.21E-29
PRR4	AC036103.1	0.63	1.79E-46
PRR4	AC004054.1	0.577	1.51E-37
PRR4	AC016590.2	0.556	1.67E-34
PRR4	ZBTB20-AS2	0.57	1.71E-36
PRR4	AL139120.1	0.545	6.82E-33
PRR4	AC008885.2	0.558	1.06E-34

PRR4	AC018607.1	0.552	5.87E-34
PRR4	AC073575.1	0.616	6.25E-44
PRR4	Z98749.1	0.625	1.29E-45
PRR4	AC003681.1	0.672	7.35E-55
PRR4	AC007494.2	0.587	4.33E-39
PRR4	AC008507.2	0.528	1.01E-30
PRR4	AC010320.2	0.537	6.57E-32
PRR4	PRKCA-AS1	0.571	1.14E-36
PRR4	AC009269.3	0.61	7.08E-43
PRR4	AL133245.1	0.61	5.64E-43
PRR4	AC015727.1	0.528	1.20E-30
PRR4	AC090607.1	0.513	9.96E-29
PRR4	TCEAL3-AS1	0.624	2.45E-45
PRR4	DANT2	0.585	8.78E-39
PRR4	AC005912.2	0.62	1.23E-44
PRR4	AC007014.1	0.533	2.41E-31
PRR4	AC090515.5	0.555	2.62E-34
PRR4	AC005899.5	0.576	1.84E-37
PRR4	LINC02039	0.51	1.94E-28
PRR4	AC091906.1	0.564	1.15E-35
PRR4	AC090519.2	0.643	5.56E-49
PRR4	AC011443.1	0.545	5.86E-33
PRR4	AC058791.1	0.602	1.61E-41
PRR4	STX18-IT1	0.596	1.21E-40
PRR4	AP002892.2	0.591	8.53E-40
PRR4	AL603756.1	0.568	2.77E-36
PRR4	AC018521.7	0.548	2.39E-33
PRR4	AL138963.1	0.601	1.91E-41
PRR4	LINC02250	0.602	1.55E-41
PRR4	AC253536.3	0.524	3.65E-30
PRR4	AC092120.1	0.559	7.58E-35
PRR4	AC093849.1	0.59	1.06E-39
PRR4	AC005154.5	0.511	1.63E-28
PRR4	AC020611.2	0.581	3.84E-38
PRR4	AL024474.2	0.551	9.00E-34
PRR4	NCOA7-AS1	0.571	1.20E-36
PRR4	AC012370.1	0.586	5.89E-39
PRR4	AL162584.1	0.57	1.71E-36
PRR4	AC021231.1	0.52	1.11E-29
PRR4	TET2-AS1	0.555	2.37E-34
PRR4	AC104982.2	0.59	1.28E-39
PRR4	C1orf195	0.588	2.50E-39
PRR4	AC025918.1	0.582	2.60E-38
PRR4	AF107885.2	0.627	5.08E-46

PRR4	AC079336.4	0.542	1.36E-32
PRR4	AC005522.2	0.626	9.01E-46
PRR4	LINC01813	0.509	2.67E-28
PRR4	AL162385.2	0.579	7.01E-38
PRR4	CACNA1C-IT3	0.612	3.09E-43
PRR4	AL133480.1	0.61	5.73E-43
PRR4	RAI1-AS1	0.553	4.01E-34
PRR4	AC104791.1	0.557	1.44E-34
PRR4	AC127164.1	0.588	2.60E-39
PRR4	AC007529.1	0.519	1.85E-29
PRR4	AL021368.3	0.545	6.18E-33
PRR4	AC007878.1	0.655	2.17E-51
PRR4	LAMTOR5-AS1	0.53	5.63E-31
PRR4	AC017002.1	0.514	7.09E-29
PRR4	AC103810.5	0.58	5.30E-38
PRR4	AP001020.2	0.584	1.00E-38
PRR4	LINC02157	0.515	5.88E-29
PRR4	AL031710.1	0.537	8.33E-32
PRR4	AC007003.1	0.539	4.09E-32
PRR4	LARGE-AS1	0.616	5.07E-44
PRR4	AC134349.1	0.637	9.16E-48
PRR4	U51244.1	0.581	3.81E-38
PRR4	AC092120.2	0.57	1.64E-36
PRR4	ATG10-IT1	0.551	9.08E-34
PRR4	CDC42-IT1	0.663	4.74E-53
PRR4	AC011120.1	0.625	1.19E-45
PRR4	AL132656.3	0.568	3.56E-36
PRR4	Z94721.2	0.641	1.17E-48
PRR4	AP001267.4	0.518	2.35E-29
PRR4	AC096708.3	0.628	3.76E-46
PRR4	AC084824.3	0.611	3.52E-43
PRR4	AC009563.1	0.61	5.00E-43
PRR4	AL353801.1	0.535	1.23E-31
PRR4	AC008056.1	0.51	2.41E-28
PRR4	AC011389.1	0.588	2.62E-39
PRR4	AC026894.1	0.567	5.00E-36
PRR4	AC005034.2	0.505	8.12E-28
PRR4	AC073591.1	0.548	2.29E-33
PRR4	AP002439.1	0.505	8.58E-28
PRR4	AL034550.1	0.507	5.15E-28
PRR4	AC027796.1	0.549	1.59E-33
PRR4	AL391863.2	0.612	3.22E-43
PRR4	MACROD2-AS1	0.558	1.04E-34
PRR4	ZBTB20-AS1	0.535	1.31E-31

PRR4	AC006441.1	0.587	4.24E-39
PRR4	AC110921.1	0.595	1.92E-40
PRR4	BIRC6-AS2	0.625	1.40E-45
PRR4	AL355297.2	0.539	4.13E-32
PRR4	AC093752.2	0.571	1.21E-36
PRR4	AC244093.5	0.596	1.30E-40
PRR4	AC134775.1	0.576	1.78E-37
PRR4	ZNF346-IT1	0.528	1.03E-30
PRR4	AC069029.1	0.504	1.02E-27
PRR4	AC027544.2	0.572	8.22E-37
PRR4	AC010530.1	0.573	6.20E-37
PRR4	EPN2-AS1	0.576	2.31E-37
PRR4	AC073476.3	0.555	2.88E-34
PRR4	EIF1AX-AS1	0.624	2.32E-45
PRR4	AC046158.2	0.618	2.50E-44
PRR4	AC092140.1	0.501	2.34E-27
PRR4	AL359094.1	0.627	6.97E-46
PRR4	AC007161.1	0.586	5.21E-39
PRR4	AC010834.3	0.537	8.30E-32
PRR4	AC005899.8	0.579	6.18E-38
PRR4	LINC02399	0.526	1.83E-30
PRR4	AL158207.2	0.555	2.57E-34
PRR4	AC010978.1	0.615	7.90E-44
PRR4	AL136115.1	0.607	1.72E-42
PRR4	AC093690.1	0.629	3.03E-46
PRR4	AL158801.3	0.63	1.57E-46
PRR4	AC009063.2	0.509	3.08E-28
PRR4	AL159174.1	0.531	4.73E-31
PRR4	MPRIP-AS1	0.568	2.81E-36
PRR4	AC006387.1	0.574	4.57E-37
PRR4	AC079209.2	0.599	3.63E-41
PRR4	AC008937.3	0.552	6.32E-34
PRR4	AC090589.2	0.524	4.13E-30
PRR4	AL445248.1	0.535	1.47E-31
PRR4	AL353771.1	0.598	6.86E-41
PRR4	AL136379.1	0.644	2.91E-49
PRR4	AP000919.3	0.581	2.89E-38
PRR4	AL445489.1	0.601	2.24E-41
