# Supplementary material for: Development of a Ferroptosis-Related lncRNA Signature to Predict the Prognosis and Immune Landscape of Bladder Cancer
Source: Dis Markers. 2021 Jun 20;2021:1031906. doi: 10.1155/2021/1031906 (PMC8238626; doi:10.1155/2021/1031906)
Supplement: Supplementary Materials — Supplementary Figure S1: validation of the risk model with resampling. Note: (a) Kaplan-Meier survival analysis of cases in high-risk group and low-risk group (p < 0.001). (b) ROC curves of the risk score in internal validation dataset. Supplementary Figure S2: Kaplan-Meier subgroup survival analysis in TCGA-BLCA patients. Note: (a) age ≤ 64 (p < 0.001). (b) Age > 64 (p < 0.001). (c) Male (p < 0.001). (d) Female (p < 0.001). (e) Pathological T 1-2 stages (p < 0.001). (f) Pathological T 3-4 stages (p < 0.001). (g) N0 stages (p < 0.001). (h) N1-N3 stages (p < 0.001). (i) M0 stage (p < 0.001). (j) M1 stage (p < 0.05). Supplementary Figure S3: functional prediction of AC090825.1 and MAGI2-AS3. Note: (a) the expression difference of AC090825.1 in BCa samples and paracarcinoma tissue via Wilcoxon signed-rank test. (b) The expression difference of MAGI2-AS3 in BCa samples and paracarcinoma tissue via Wilcoxon signed-rank test. (c) The difference values between the expression of AC090825.1 and MAGI2-AS3 in BCa samples and paracarcinoma samples. (d) The cases suffered poorer survival when the expression value of AC090825.1 was higher than that of MAGI2-AS3 (p < 0.01). (e) The expression levels of AC090825.1 and MAGI2-AS3 were positively correlated both in BCa samples and paracarcinoma samples. (f) Network of lncRNAs, the target genes and their possible transcription factors. (g) UpSet diagram showing 6 overlapped transcription factors. (h) The correlation network of lncRNAs, the target genes and transcription factors. BCa: bladder cancer. Supplementary Table S1: 20 ferroptosis regulators were differentially expressed between BCa and paracarinoma samples. Supplementary Table S2: the Pearson correlation analysis of lncRNAs and ferroptosis regulators. Supplementary Table S3: Kaplan-Meier survival analysis of the lncRNA pairs. Supplementary Table S4: enrichment results from LncSEA website. Supplementary Table S5: GSVA results of different clusters based on ferroptosis regulators [file 1031906.f1.zip › Supplementary_Table_S3.PDF]

Supplementary Table S3. Kaplan-Meier survival analysis of the lncRNA pairs.

lncRNA_pair	pvalue
AC025259.3_AC079313.2	0.74484172
AC025259.3_AC135012.3	0.576596349
AC025259.3_AC036108.3	0.954511176
AC025259.3_AC012085.2	0.191742987
AC025259.3_MAGI2-AS3	0.816973847
AC025259.3_CARMN	0.313173056
AC025259.3_AL445423.1	0.258627355
AC025259.3_AC134312.5	0.81997528
AC025259.3_AC090825.1	0.642554094
AC025259.3_ADAMTS9-AS1	0.423194447
AC025259.3_MEF2C-AS1	0.437036527
AC025259.3_LINC01778	0.217472726
AC025259.3_AF001548.1	0.46016261
AC025259.3_AC002398.2	0.274990445
AC025259.3_AL662860.1	0.760272528
AC025259.3_AC079313.1	0.640105571
AC025259.3_AC131097.4	0.210678556
AC025259.3_AC092376.2	0.341045394
AC025259.3_AC053503.4	0.428232515
AC025259.3_AC027449.1	0.723790906
AC025259.3_AP001189.1	0.889974049
AC025259.3_AC005180.1	0.866653442
AC025259.3_FENDRR	0.191875996
AC025259.3_LINC02202	0.709967533
AC025259.3_AP003071.4	0.647495883
AC025259.3_AL365214.2	0.682376637
AC025259.3_ADAMTS9-AS2	0.593074388
AC025259.3_AP001107.5	0.77645967
AC025259.3_AC087521.1	0.174700132
AC025259.3_AC007671.1	0.262524086
AC025259.3_LINC01081	0.842720455
AC025259.3_HAND2-AS1	0.611269327
AC025259.3_AC005180.2	0.515090968
AC025259.3_LINC00702	0.331496414
AC025259.3_MBNL1-AS1	0.642523462
AC025259.3_LINC01614	0.197095881
AC025259.3_MIR100HG	0.176140014
AC025259.3_JAZF1-AS1	0.798048088
AC025259.3_AC022893.1	0.589618307
AC025259.3_ACTA2-AS1	0.865083787
AC025259.3_AP003071.3	0.963095283

AC025259.3_LINC01352	0.754878067
AC025259.3_AC134312.1	0.423013826
AC025259.3_AC091588.3	0.933707462
AC025259.3_HLX-AS1	0.154761093
AC025259.3_LINC00402	0.361730561
AC025259.3_LINC01781	0.703629198
AC025259.3_AC007128.1	0.709466597
AC025259.3_AC099850.3	0.453422575
AC025259.3_AC010655.2	0.458659055
AC025259.3_LINC00941	0.86438278
AC025259.3_MYOSLID	0.958756902
AC025259.3_AL161772.1	0.835372727
AC025259.3_AP005432.2	0.17097029
AC025259.3_AC099066.2	0.858582981
AC025259.3_CDKN2B-AS1	0.369395081
AC025259.3_AL449423.1	0.470202132
AC025259.3_AL445490.1	0.650452297
AC025259.3_U62317.2	0.013057568
AC025259.3_AC004988.1	0.028643947
AC025259.3_LINC02195	0.012514199
AC025259.3_AC090125.1	0.319797226
AC025259.3_LINC01048	0.175109392
AC025259.3_AC090673.1	0.92385728
AC025259.3_AL356417.2	0.441380766
AC025259.3_AC007750.1	0.374462749
AC025259.3_LINC01561	0.702057643
AC025259.3_LINC01833	0.28778544
AC025259.3_LINC00460	0.912979211
AC025259.3_LINC00536	0.142165032
AC025259.3_AP000553.1	0.437608136
AC025259.3_AATBC	0.008679564
AC025259.3_AL161431.1	0.162999734
AC025259.3_PICSAR	0.84793586
AC025259.3_LINC01615	0.50910978
AC025259.3_LINC00641	0.603143915
AC025259.3_MIR133A1HG	0.451885041
AC025259.3_AC104984.6	0.615771239
AC025259.3_AL023755.1	0.436238586
AC025259.3_AL359851.1	0.164226041
AC025259.3_AC015923.1	0.218730459
AC025259.3_AC005828.1	0.272924174
AC025259.3_AC023034.1	0.247509479
AC025259.3_AC099811.5	0.829350956
AC025259.3_FP325330.3	0.822861601

AC025259.3_AL355075.4	0.814551516
AC025259.3_RMRP	0.593230643
AC025259.3_AC037487.2	0.531218345
AC025259.3_AC006141.1	0.164306928
AC025259.3_LINC02443	0.113648425
AC025259.3_MIR181A1HG	0.758451339
AC025259.3_LINC02465	0.369605915
AC025259.3_AC091906.1	0.852268663
AC079313.2_AC135012.3	0.609952372
AC079313.2_AC036108.3	0.132220263
AC079313.2_AC012085.2	0.865748285
AC079313.2_MAGI2-AS3	0.258303167
AC079313.2_CARMN	0.418951491
AC079313.2_AL445423.1	0.873699915
AC079313.2_AC134312.5	0.830079297
AC079313.2_AC090825.1	0.245957762
AC079313.2_ADAMTS9-AS1	0.937500094
AC079313.2_MEF2C-AS1	0.825060731
AC079313.2_LINC01778	0.890479377
AC079313.2_AF001548.1	0.402374954
AC079313.2_AC002398.2	0.950234375
AC079313.2_AL662860.1	0.112770293
AC079313.2_AC079313.1	0.384730401
AC079313.2_AC131097.4	0.142671535
AC079313.2_AC092376.2	0.774375101
AC079313.2_AC053503.4	0.130395224
AC079313.2_AC027449.1	0.613318705
AC079313.2_AP001189.1	0.444529485
AC079313.2_AC005180.1	0.995595664
AC079313.2_FENDRR	0.845346298
AC079313.2_LINC02202	0.0581344
AC079313.2_AP003071.4	0.832121256
AC079313.2_AL365214.2	0.086950908
AC079313.2_ADAMTS9-AS2	0.991949439
AC079313.2_AP001107.5	0.827307812
AC079313.2_AC087521.1	0.340595814
AC079313.2_AC007671.1	0.851900557
AC079313.2_LINC01081	0.706684225
AC079313.2_HAND2-AS1	0.506932171
AC079313.2_AC005180.2	0.849506524
AC079313.2_LINC00702	0.880770791
AC079313.2_MBNL1-AS1	0.419510648
AC079313.2_LINC01614	0.175115965
AC079313.2_MIR100HG	0.925904653

AC079313.2_JAZF1-AS1	0.831319582
AC079313.2_AC022893.1	0.278945117
AC079313.2_ACTA2-AS1	0.708076617
AC079313.2_AP003071.3	0.969749782
AC079313.2_LINC01352	0.590851029
AC079313.2_AC134312.1	0.809202929
AC079313.2_AC091588.3	0.753313877
AC079313.2_HLX-AS1	0.770150564
AC079313.2_LINC00402	0.960693966
AC079313.2_LINC01781	0.558589985
AC079313.2_AC007128.1	0.27373507
AC079313.2_AC099850.3	0.833380994
AC079313.2_AC010655.2	0.120982237
AC079313.2_LINC00941	0.59879892
AC079313.2_MYOSLID	0.618239613
AC079313.2_AL161772.1	0.15600072
AC079313.2_AP005432.2	0.350628819
AC079313.2_AC099066.2	0.34943237
AC079313.2_CDKN2B-AS1	0.3026834
AC079313.2_AL449423.1	0.477986338
AC079313.2_AL445490.1	0.685438644
AC079313.2_U62317.2	0.983918388
AC079313.2_AC004988.1	0.265760932
AC079313.2_LINC02195	0.024849276
AC079313.2_AC090125.1	0.846303912
AC079313.2_LINC01048	0.74692023
AC079313.2_AC090673.1	0.958220365
AC079313.2_AL356417.2	0.944726724
AC079313.2_AC007750.1	0.690179999
AC079313.2_LINC01561	0.981897815
AC079313.2_LINC01833	0.013206181
AC079313.2_LINC00460	0.783732051
AC079313.2_LINC00536	0.463466872
AC079313.2_AP000553.1	0.619450809
AC079313.2_AATBC	0.396020334
AC079313.2_AL161431.1	0.364320905
AC079313.2_PICSAR	0.026216817
AC079313.2_LINC01615	0.448320715
AC079313.2_LINC00641	0.200182911
AC079313.2_MIR133A1HG	0.52423708
AC079313.2_AC104984.6	0.277431504
AC079313.2_AL023755.1	0.116553653
AC079313.2_AL359851.1	0.642623959
AC079313.2_AC015923.1	0.434761321

AC079313.2_AC005828.1	0.651610864
AC079313.2_AC023034.1	0.091896048
AC079313.2_AC099811.5	0.519155133
AC079313.2_FP325330.3	0.186477504
AC079313.2_AL355075.4	0.651498199
AC079313.2_RMRP	0.880789632
AC079313.2_AC037487.2	0.657804465
AC079313.2_AC006141.1	0.640546637
AC079313.2_LINC02443	0.268304021
AC079313.2_MIR181A1HG	0.621274957
AC079313.2_LINC02465	0.315683895
AC079313.2_AC091906.1	0.446518937
AC135012.3_AC036108.3	0.715432325
AC135012.3_AC012085.2	0.350860101
AC135012.3_MAGI2-AS3	0.407874937
AC135012.3_CARMN	0.566511474
AC135012.3_AL445423.1	0.639116586
AC135012.3_AC134312.5	0.360391478
AC135012.3_AC090825.1	0.919804442
AC135012.3_ADAMTS9-AS1	0.132218553
AC135012.3_MEF2C-AS1	0.461483482
AC135012.3_LINC01778	0.103798911
AC135012.3_AF001548.1	0.589547935
AC135012.3_AC002398.2	0.255415665
AC135012.3_AL662860.1	0.919627565
AC135012.3_AC079313.1	0.844651035
AC135012.3_AC131097.4	0.940180258
AC135012.3_AC092376.2	0.848620718
AC135012.3_AC053503.4	0.031051843
AC135012.3_AC027449.1	0.507511369
AC135012.3_AP001189.1	0.57357408
AC135012.3_AC005180.1	0.076354248
AC135012.3_FENDRR	0.09243187
AC135012.3_LINC02202	0.162097501
AC135012.3_AP003071.4	0.003473122
AC135012.3_AL365214.2	0.894777444
AC135012.3_ADAMTS9-AS2	0.906734935
AC135012.3_AP001107.5	0.233417159
AC135012.3_AC087521.1	0.884998864
AC135012.3_AC007671.1	0.903739494
AC135012.3_LINC01081	0.961695004
AC135012.3_HAND2-AS1	0.527608125
AC135012.3_AC005180.2	0.886571057
AC135012.3_LINC00702	0.585451409

AC135012.3_MBNL1-AS1	0.859486007
AC135012.3_LINC01614	0.095955582
AC135012.3_MIR100HG	0.678188409
AC135012.3_JAZF1-AS1	0.639127535
AC135012.3_AC022893.1	0.62130207
AC135012.3_ACTA2-AS1	0.46653338
AC135012.3_AP003071.3	0.954601106
AC135012.3_LINC01352	0.601700425
AC135012.3_AC134312.1	0.870749375
AC135012.3_AC091588.3	0.890091447
AC135012.3_HLX-AS1	0.158651078
AC135012.3_LINC00402	0.374125348
AC135012.3_LINC01781	0.654703801
AC135012.3_AC007128.1	0.137188492
AC135012.3_AC099850.3	0.354835519
AC135012.3_AC010655.2	0.643315294
AC135012.3_LINC00941	0.935211049
AC135012.3_MYOSLID	0.152741481
AC135012.3_AL161772.1	0.359451615
AC135012.3_AP005432.2	0.169591923
AC135012.3_AC099066.2	0.800986547
AC135012.3_CDKN2B-AS1	0.141876518
AC135012.3_AL449423.1	0.468007917
AC135012.3_AL445490.1	0.357936712
AC135012.3_AC004988.1	0.790570662
AC135012.3_LINC02195	0.40228594
AC135012.3_AC090125.1	0.857242632
AC135012.3_LINC01048	0.903182645
AC135012.3_AC090673.1	0.155631221
AC135012.3_AL356417.2	0.080640598
AC135012.3_AC007750.1	0.636965027
AC135012.3_LINC01561	0.243247596
AC135012.3_LINC01833	0.814657844
AC135012.3_LINC00460	0.923377418
AC135012.3_LINC00536	0.249446013
AC135012.3_AP000553.1	0.513073602
AC135012.3_AATBC	0.865508297
AC135012.3_AL161431.1	0.014120778
AC135012.3_PICSAR	0.375914395
AC135012.3_LINC01615	0.290336232
AC135012.3_LINC00641	0.174131458
AC135012.3_MIR133A1HG	0.319156685
AC135012.3_AC104984.6	0.339467079
AC135012.3_AL023755.1	0.41673466

AC135012.3_AL359851.1	0.868156122
AC135012.3_AC015923.1	0.266564038
AC135012.3_AC005828.1	0.408125681
AC135012.3_AC023034.1	0.768974017
AC135012.3_AC099811.5	0.417272922
AC135012.3_FP325330.3	0.6211301
AC135012.3_AL355075.4	0.252896482
AC135012.3_RMRP	0.983919989
AC135012.3_AC037487.2	0.724451097
AC135012.3_AC006141.1	0.745732368
AC135012.3_LINC02443	0.146776455
AC135012.3_MIR181A1HG	0.143559333
AC135012.3_LINC02465	0.245926764
AC135012.3_AC091906.1	0.318723075
AC036108.3_AC012085.2	0.724047773
AC036108.3_MAGI2-AS3	0.329660545
AC036108.3_CARMN	0.439080547
AC036108.3_AL445423.1	0.782288832
AC036108.3_AC134312.5	0.415917374
AC036108.3_AC090825.1	0.707220439
AC036108.3_ADAMTS9-AS1	0.440913738
AC036108.3_MEF2C-AS1	0.502630435
AC036108.3_LINC01778	0.868053812
AC036108.3_AF001548.1	0.087377543
AC036108.3_AC002398.2	0.404570752
AC036108.3_AL662860.1	0.692164123
AC036108.3_AC079313.1	0.060252648
AC036108.3_AC131097.4	0.17625431
AC036108.3_AC092376.2	0.927724072
AC036108.3_AC053503.4	0.064175621
AC036108.3_AC027449.1	0.995533717
AC036108.3_AP001189.1	0.195199182
AC036108.3_AC005180.1	0.021978232
AC036108.3_FENDRR	0.406620606
AC036108.3_LINC02202	0.690208791
AC036108.3_AP003071.4	0.299676421
AC036108.3_AL365214.2	0.308081961
AC036108.3_ADAMTS9-AS2	0.160301881
AC036108.3_AP001107.5	0.087906779
AC036108.3_AC087521.1	0.758672715
AC036108.3_AC007671.1	0.108773993
AC036108.3_LINC01081	0.580384009
AC036108.3_HAND2-AS1	0.248103335
AC036108.3_AC005180.2	0.95964053

AC036108.3_LINC00702	0.068199045
AC036108.3_MBNL1-AS1	0.808821259
AC036108.3_LINC01614	0.147904344
AC036108.3_MIR100HG	0.165516922
AC036108.3_JAZF1-AS1	0.479928606
AC036108.3_AC022893.1	0.290747129
AC036108.3_ACTA2-AS1	0.838631313
AC036108.3_AP003071.3	0.478916322
AC036108.3_LINC01352	0.789312055
AC036108.3_AC134312.1	0.060883022
AC036108.3_AC091588.3	0.866624918
AC036108.3_HLX-AS1	0.07470095
AC036108.3_LINC00402	0.674533436
AC036108.3_LINC01781	0.260938418
AC036108.3_AC007128.1	0.318880952
AC036108.3_AC099850.3	0.787775639
AC036108.3_AC010655.2	0.233455482
AC036108.3_LINC00941	0.85279006
AC036108.3_MYOSLID	0.679306826
AC036108.3_AL161772.1	0.441743438
AC036108.3_AP005432.2	0.005314681
AC036108.3_AC099066.2	0.176515416
AC036108.3_CDKN2B-AS1	0.781340766
AC036108.3_AL449423.1	0.371349351
AC036108.3_AL445490.1	0.585145716
AC036108.3_U62317.2	0.243805677
AC036108.3_AC004988.1	0.070170929
AC036108.3_LINC02195	0.072811728
AC036108.3_AC090125.1	0.678393397
AC036108.3_LINC01048	0.984436998
AC036108.3_AC090673.1	0.348641461
AC036108.3_AL356417.2	0.994815917
AC036108.3_AC007750.1	0.996537917
AC036108.3_LINC01561	0.921203657
AC036108.3_LINC01833	0.163540598
AC036108.3_LINC00460	0.96636369
AC036108.3_LINC00536	0.01144786
AC036108.3_AP000553.1	0.834912477
AC036108.3_AATBC	0.237907703
AC036108.3_AL161431.1	0.004494628
AC036108.3_PICSAR	0.221844657
AC036108.3_LINC01615	0.260323213
AC036108.3_LINC00641	0.076853334
AC036108.3_MIR133A1HG	0.941349593

AC036108.3_AC104984.6	0.484995526
AC036108.3_AL023755.1	0.213866488
AC036108.3_AL359851.1	0.862647971
AC036108.3_AC015923.1	0.256980987
AC036108.3_AC005828.1	0.441528909
AC036108.3_AC023034.1	0.352918205
AC036108.3_AC099811.5	0.256613985
AC036108.3_FP325330.3	0.275849708
AC036108.3_AL355075.4	0.699289055
AC036108.3_RMRP	0.111952669
AC036108.3_AC037487.2	0.42618844
AC036108.3_AC006141.1	0.528274881
AC036108.3_LINC02443	0.366979662
AC036108.3_MIR181A1HG	0.16395421
AC036108.3_LINC02465	0.356986142
AC036108.3_AC091906.1	0.40848861
AC012085.2_MAGI2-AS3	0.349189152
AC012085.2_CARMN	0.786526168
AC012085.2_AL445423.1	0.541469957
AC012085.2_AC134312.5	0.143740109
AC012085.2_AC090825.1	0.93349742
AC012085.2_ADAMTS9-AS1	0.31501346
AC012085.2_MEF2C-AS1	0.981764863
AC012085.2_LINC01778	0.293589597
AC012085.2_AF001548.1	0.127494712
AC012085.2_AC002398.2	0.331489684
AC012085.2_AL662860.1	0.73900549
AC012085.2_AC079313.1	0.937123976
AC012085.2_AC131097.4	0.760267261
AC012085.2_AC092376.2	0.618987889
AC012085.2_AC053503.4	0.078038292
AC012085.2_AC027449.1	0.639252153
AC012085.2_AP001189.1	0.297928398
AC012085.2_AC005180.1	0.395918563
AC012085.2_FENDRR	0.040623748
AC012085.2_LINC02202	0.479097761
AC012085.2_AP003071.4	0.310260481
AC012085.2_AL365214.2	0.055608269
AC012085.2_ADAMTS9-AS2	0.223929905
AC012085.2_AP001107.5	0.640315719
AC012085.2_AC087521.1	0.212440299
AC012085.2_AC007671.1	0.963208613
AC012085.2_LINC01081	0.642044638
AC012085.2_HAND2-AS1	0.993390973

AC012085.2_AC005180.2	0.912060512
AC012085.2_LINC00702	0.309957892
AC012085.2_MBNL1-AS1	0.167762852
AC012085.2_LINC01614	0.419982696
AC012085.2_MIR100HG	0.602078727
AC012085.2_JAZF1-AS1	0.733605664
AC012085.2_AC022893.1	0.521827149
AC012085.2_ACTA2-AS1	0.442263634
AC012085.2_AP003071.3	0.46073412
AC012085.2_LINC01352	0.530090457
AC012085.2_AC134312.1	0.536041599
AC012085.2_AC091588.3	0.517081196
AC012085.2_HLX-AS1	0.119505436
AC012085.2_LINC00402	0.698729083
AC012085.2_LINC01781	0.37619107
AC012085.2_AC007128.1	0.721834953
AC012085.2_AC099850.3	0.822813831
AC012085.2_AC010655.2	0.842398165
AC012085.2_LINC00941	0.798872188
AC012085.2_MYOSLID	0.436266419
AC012085.2_AL161772.1	0.78065419
AC012085.2_AP005432.2	0.232179275
AC012085.2_AC099066.2	0.809747675
AC012085.2_CDKN2B-AS1	0.477064061
AC012085.2_AL449423.1	0.598737071
AC012085.2_AL445490.1	0.952210708
AC012085.2_U62317.2	0.369798153
AC012085.2_AC004988.1	0.687831387
AC012085.2_LINC02195	0.700912916
AC012085.2_AC090125.1	0.637487408
AC012085.2_LINC01048	0.469640507
AC012085.2_AC090673.1	0.109780225
AC012085.2_AL356417.2	0.07762097
AC012085.2_AC007750.1	0.785185712
AC012085.2_LINC01561	0.366919155
AC012085.2_LINC01833	0.102087501
AC012085.2_LINC00460	0.718708202
AC012085.2_LINC00536	0.026016075
AC012085.2_AP000553.1	0.783986287
AC012085.2_AATBC	0.959960253
AC012085.2_AL161431.1	0.039007737
AC012085.2_PICSAR	0.144472202
AC012085.2_LINC01615	0.328547168
AC012085.2_LINC00641	0.588864523

AC012085.2_MIR133A1HG	0.469993206
AC012085.2_AC104984.6	0.316430938
AC012085.2_AL023755.1	0.238798622
AC012085.2_AL359851.1	0.231135395
AC012085.2_AC015923.1	0.051276278
AC012085.2_AC005828.1	0.376696157
AC012085.2_AC023034.1	0.989098265
AC012085.2_AC099811.5	0.958395496
AC012085.2_FP325330.3	0.127435413
AC012085.2_AL355075.4	0.155255778
AC012085.2_RMRP	0.880824756
AC012085.2_AC037487.2	0.400030237
AC012085.2_AC006141.1	0.04427653
AC012085.2_LINC02443	0.113033798
AC012085.2_MIR181A1HG	0.756206675
AC012085.2_LINC02465	0.62985379
AC012085.2_AC091906.1	0.15408027
MAGI2-AS3_CARMN	0.898521232
MAGI2-AS3_AL445423.1	0.620250035
MAGI2-AS3_AC134312.5	0.755231587
MAGI2-AS3_AC090825.1	0.00106639
MAGI2-AS3_ADAMTS9-AS1	0.137510305
MAGI2-AS3_MEF2C-AS1	0.528936567
MAGI2-AS3_LINC01778	0.427778885
MAGI2-AS3_AF001548.1	0.728337992
MAGI2-AS3_AC002398.2	0.547745624
MAGI2-AS3_AL662860.1	0.357946483
MAGI2-AS3_AC079313.1	0.671523732
MAGI2-AS3_AC131097.4	0.247650946
MAGI2-AS3_AC092376.2	0.619759077
MAGI2-AS3_AC053503.4	0.221663615
MAGI2-AS3_AC027449.1	0.83876656
MAGI2-AS3_AP001189.1	0.244994496
MAGI2-AS3_AC005180.1	0.04096028
MAGI2-AS3_FENDRR	0.336003036
MAGI2-AS3_LINC02202	0.6590159
MAGI2-AS3_AP003071.4	0.732807591
MAGI2-AS3_AL365214.2	0.772991706
MAGI2-AS3_AP001107.5	0.275009988
MAGI2-AS3_AC087521.1	0.043380715
MAGI2-AS3_AC007671.1	0.84535188
MAGI2-AS3_LINC01081	0.018714888
MAGI2-AS3_HAND2-AS1	0.879918478
MAGI2-AS3_AC005180.2	0.149712496

MAGI2-AS3_LINC00702	0.096187727
MAGI2-AS3_MBNL1-AS1	0.43616155
MAGI2-AS3_LINC01614	0.097829673
MAGI2-AS3_MIR100HG	0.015084977
MAGI2-AS3_AC022893.1	0.97533661
MAGI2-AS3_ACTA2-AS1	0.392354297
MAGI2-AS3_AP003071.3	0.899150622
MAGI2-AS3_LINC01352	0.533913447
MAGI2-AS3_AC091588.3	0.542356725
MAGI2-AS3_HLX-AS1	0.667761863
MAGI2-AS3_LINC00402	0.040870121
MAGI2-AS3_LINC01781	0.854122355
MAGI2-AS3_AC007128.1	0.313559697
MAGI2-AS3_AC099850.3	0.957537716
MAGI2-AS3_AC010655.2	0.292210284
MAGI2-AS3_LINC00941	0.410121607
MAGI2-AS3_MYOSLID	0.440000376
MAGI2-AS3_AL161772.1	0.472515301
MAGI2-AS3_AP005432.2	0.614079483
MAGI2-AS3_AC099066.2	0.694710467
MAGI2-AS3_CDKN2B-AS1	0.393489
MAGI2-AS3_AL449423.1	0.874341804
MAGI2-AS3_AL445490.1	0.132975975
MAGI2-AS3_U62317.2	0.636228414
MAGI2-AS3_AC004988.1	0.347520998
MAGI2-AS3_LINC02195	0.006013702
MAGI2-AS3_AC090125.1	0.790880414
MAGI2-AS3_LINC01048	0.956412037
MAGI2-AS3_AC090673.1	0.892252415
MAGI2-AS3_AL356417.2	0.677426005
MAGI2-AS3_AC007750.1	0.989378672
MAGI2-AS3_LINC01561	0.97465488
MAGI2-AS3_LINC01833	0.012635833
MAGI2-AS3_LINC00460	0.217829259
MAGI2-AS3_LINC00536	8.14E-06
MAGI2-AS3_AP000553.1	0.915724109
MAGI2-AS3_AATBC	0.067086981
MAGI2-AS3_AL161431.1	0.059700593
MAGI2-AS3_PICSAR	0.831534593
MAGI2-AS3_LINC01615	0.085510612
MAGI2-AS3_LINC00641	0.139929044
MAGI2-AS3_MIR133A1HG	0.444611284
MAGI2-AS3_AC104984.6	0.617489274
MAGI2-AS3_AL023755.1	0.300204424

MAGI2-AS3_AL359851.1	0.212852055
MAGI2-AS3_AC015923.1	0.098383299
MAGI2-AS3_AC005828.1	0.724948825
MAGI2-AS3_AC023034.1	0.158401573
MAGI2-AS3_AC099811.5	0.997297611
MAGI2-AS3_FP325330.3	0.026632584
MAGI2-AS3_AL355075.4	0.601595079
MAGI2-AS3_RMRP	0.000579441
MAGI2-AS3_AC037487.2	0.075314771
MAGI2-AS3_AC006141.1	0.712773903
MAGI2-AS3_LINC02443	0.184881321
MAGI2-AS3_LINC02465	0.989335393
MAGI2-AS3_AC091906.1	0.314073376
CARMN_AL445423.1	0.763080246
CARMN_AC134312.5	0.680351118
CARMN_AC090825.1	0.350591528
CARMN_ADAMTS9-AS1	0.03794082
CARMN_MEF2C-AS1	0.155066013
CARMN_LINC01778	0.105247147
CARMN_AF001548.1	0.22756964
CARMN_AC002398.2	0.170554556
CARMN_AL662860.1	0.646829788
CARMN_AC079313.1	0.857952244
CARMN_AC131097.4	0.345470301
CARMN_AC092376.2	0.209882932
CARMN_AC053503.4	0.039567602
CARMN_AC027449.1	0.87338362
CARMN_AP001189.1	0.662274406
CARMN_AC005180.1	0.677724643
CARMN_FENDRR	0.006420594
CARMN_LINC02202	0.260097677
CARMN_AP003071.4	0.119833699
CARMN_AL365214.2	0.312645329
CARMN_ADAMTS9-AS2	0.543204354
CARMN_AP001107.5	0.284100129
CARMN_AC087521.1	0.848343978
CARMN_AC007671.1	0.621237838
CARMN_LINC01081	0.894973357
CARMN_HAND2-AS1	0.490916824
CARMN_AC005180.2	0.242742437
CARMN_LINC00702	0.125558727
CARMN_MBNL1-AS1	0.179224151
CARMN_LINC01614	0.04321467
CARMN_MIR100HG	0.760526762

CARMN_JAZF1-AS1	0.907204679
CARMN_AC022893.1	0.79788335
CARMN_ACTA2-AS1	0.087391154
CARMN_AP003071.3	0.127683275
CARMN_LINC01352	0.805049052
CARMN_AC134312.1	0.329790308
CARMN_AC091588.3	0.991382501
CARMN_HLX-AS1	0.838759579
CARMN_LINC00402	0.069434927
CARMN_LINC01781	0.595235202
CARMN_AC007128.1	0.340812516
CARMN_AC099850.3	0.354835519
CARMN_AC010655.2	0.851966664
CARMN_LINC00941	0.801775131
CARMN_MYOSLID	0.701558714
CARMN_AL161772.1	0.555144872
CARMN_AP005432.2	0.032402342
CARMN_AC099066.2	0.850990608
CARMN_CDKN2B-AS1	0.053313226
CARMN_AL449423.1	0.624095843
CARMN_AL445490.1	0.384069861
CARMN_AC004988.1	0.107060594
CARMN_LINC02195	0.237150625
CARMN_AC090125.1	0.918199918
CARMN_LINC01048	0.926269472
CARMN_AC090673.1	0.414907491
CARMN_AL356417.2	0.263913644
CARMN_AC007750.1	0.797981197
CARMN_LINC01561	0.662351473
CARMN_LINC01833	0.169638835
CARMN_LINC00460	0.853035205
CARMN_LINC00536	0.054734436
CARMN_AP000553.1	0.78965384
CARMN_AATBC	0.238382608
CARMN_AL161431.1	0.007071572
CARMN_PICSAR	0.303630376
CARMN_LINC01615	0.722469614
CARMN_LINC00641	0.109885371
CARMN_MIR133A1HG	0.156551092
CARMN_AC104984.6	0.214642867
CARMN_AL023755.1	0.144946059
CARMN_AL359851.1	0.177569584
CARMN_AC015923.1	0.002991827
CARMN_AC005828.1	0.029119543

CARMN_AC023034.1	0.27366129
CARMN_AC099811.5	0.842291368
CARMN_FP325330.3	0.010334867
CARMN_AL355075.4	0.281023504
CARMN_RMRP	0.367689655
CARMN_AC037487.2	0.359494453
CARMN_AC006141.1	0.638598204
CARMN_LINC02443	0.169140428
CARMN_MIR181A1HG	0.866241882
CARMN_LINC02465	0.823500706
CARMN_AC091906.1	0.304364265
AL445423.1_AC134312.5	0.067583192
AL445423.1_AC090825.1	0.975276719
AL445423.1_ADAMTS9-AS1	0.926057049
AL445423.1_MEF2C-AS1	0.714784292
AL445423.1_LINC01778	0.940320898
AL445423.1_AF001548.1	0.985486654
AL445423.1_AC002398.2	0.906270913
AL445423.1_AL662860.1	0.876861623
AL445423.1_AC079313.1	0.566261892
AL445423.1_AC131097.4	0.591520259
AL445423.1_AC092376.2	0.936768262
AL445423.1_AC053503.4	0.146049409
AL445423.1_AC027449.1	0.970964168
AL445423.1_AP001189.1	0.611814169
AL445423.1_AC005180.1	0.458263323
AL445423.1_FENDRR	0.728468019
AL445423.1_LINC02202	0.677158211
AL445423.1_AP003071.4	0.609319858
AL445423.1_AL365214.2	0.88916944
AL445423.1_ADAMTS9-AS2	0.698432194
AL445423.1_AP001107.5	0.066390824
AL445423.1_AC087521.1	0.484502572
AL445423.1_AC007671.1	0.658359447
AL445423.1_LINC01081	0.408384303
AL445423.1_HAND2-AS1	0.453653568
AL445423.1_AC005180.2	0.154914129
AL445423.1_LINC00702	0.034493651
AL445423.1_MBNL1-AS1	0.996590863
AL445423.1_LINC01614	0.011569176
AL445423.1_MIR100HG	0.279059879
AL445423.1_JAZF1-AS1	0.896560852
AL445423.1_AC022893.1	0.398508924
AL445423.1_ACTA2-AS1	0.822289868

AL445423.1_AP003071.3	0.86798575
AL445423.1_LINC01352	0.24362232
AL445423.1_AC134312.1	0.829809148
AL445423.1_AC091588.3	0.579026492
AL445423.1_HLX-AS1	0.873153944
AL445423.1_LINC00402	0.739267181
AL445423.1_LINC01781	0.894010666
AL445423.1_AC007128.1	0.930766021
AL445423.1_AC099850.3	0.13058989
AL445423.1_AC010655.2	0.106098491
AL445423.1_LINC00941	0.74462203
AL445423.1_MYOSLID	0.723474764
AL445423.1_AL161772.1	0.768613946
AL445423.1_AP005432.2	0.265478592
AL445423.1_AC099066.2	0.611749198
AL445423.1_CDKN2B-AS1	0.864238314
AL445423.1_AL449423.1	0.70629228
AL445423.1_AL445490.1	0.276189243
AL445423.1_AC004988.1	0.258452624
AL445423.1_LINC02195	0.406731469
AL445423.1_AC090125.1	0.309161327
AL445423.1_LINC01048	0.665554075
AL445423.1_AC090673.1	0.95390935
AL445423.1_AL356417.2	0.235422877
AL445423.1_AC007750.1	0.836508026
AL445423.1_LINC01561	0.771206699
AL445423.1_LINC01833	0.501884466
AL445423.1_LINC00460	0.233370436
AL445423.1_LINC00536	0.703596831
AL445423.1_AP000553.1	0.928042184
AL445423.1_AATBC	0.230510982
AL445423.1_AL161431.1	0.051241029
AL445423.1_PICSAR	0.90980408
AL445423.1_LINC01615	0.03529139
AL445423.1_LINC00641	0.091992737
AL445423.1_MIR133A1HG	0.916978062
AL445423.1_AC104984.6	0.624826213
AL445423.1_AL023755.1	0.483298249
AL445423.1_AL359851.1	0.967354393
AL445423.1_AC015923.1	0.515623398
AL445423.1_AC005828.1	0.573174481
AL445423.1_AC023034.1	0.782035944
AL445423.1_AC099811.5	0.45150802
AL445423.1_FP325330.3	0.354075914

AL445423.1_AL355075.4	0.779442423
AL445423.1_RMRP	0.76064857
AL445423.1_AC037487.2	0.97452236
AL445423.1_AC006141.1	0.934817137
AL445423.1_LINC02443	0.568443431
AL445423.1_MIR181A1HG	0.951252024
AL445423.1_LINC02465	0.954207644
AL445423.1_AC091906.1	0.759439456
AC134312.5_AC090825.1	0.913430322
AC134312.5_ADAMTS9-AS1	0.897695695
AC134312.5_MEF2C-AS1	0.614095481
AC134312.5_LINC01778	0.236947373
AC134312.5_AF001548.1	0.518154983
AC134312.5_AC002398.2	0.887444469
AC134312.5_AL662860.1	0.873826729
AC134312.5_AC079313.1	0.298608625
AC134312.5_AC131097.4	0.963809931
AC134312.5_AC092376.2	0.486600058
AC134312.5_AC053503.4	0.355379205
AC134312.5_AC027449.1	0.648964621
AC134312.5_AP001189.1	0.824928725
AC134312.5_AC005180.1	0.528272801
AC134312.5_FENDRR	0.742259501
AC134312.5_LINC02202	0.369728199
AC134312.5_AP003071.4	0.875965429
AC134312.5_AL365214.2	0.275445766
AC134312.5_ADAMTS9-AS2	0.975542474
AC134312.5_AP001107.5	0.748177346
AC134312.5_AC087521.1	0.271981828
AC134312.5_AC007671.1	0.235142567
AC134312.5_LINC01081	0.708860203
AC134312.5_HAND2-AS1	0.297363013
AC134312.5_AC005180.2	0.228967921
AC134312.5_LINC00702	0.746074175
AC134312.5_MBNL1-AS1	0.21242532
AC134312.5_LINC01614	0.344265579
AC134312.5_MIR100HG	0.149058436
AC134312.5_JAZF1-AS1	0.84448696
AC134312.5_AC022893.1	0.308241863
AC134312.5_ACTA2-AS1	0.137001666
AC134312.5_AP003071.3	0.823648955
AC134312.5_LINC01352	0.269756909
AC134312.5_AC134312.1	0.888217067
AC134312.5_AC091588.3	0.877429704

AC134312.5_HLX-AS1	0.724761946
AC134312.5_LINC00402	0.682283833
AC134312.5_LINC01781	0.723103664
AC134312.5_AC007128.1	0.12368055
AC134312.5_AC099850.3	0.052136699
AC134312.5_AC010655.2	0.403146561
AC134312.5_LINC00941	0.461513959
AC134312.5_MYOSLID	0.830665427
AC134312.5_AL161772.1	0.774378633
AC134312.5_AP005432.2	0.198020761
AC134312.5_AC099066.2	0.190770522
AC134312.5_CDKN2B-AS1	0.305254312
AC134312.5_AL449423.1	0.633424439
AC134312.5_AL445490.1	0.339709154
AC134312.5_U62317.2	0.304831993
AC134312.5_AC004988.1	0.466149614
AC134312.5_LINC02195	0.018152359
AC134312.5_AC090125.1	0.416120125
AC134312.5_LINC01048	0.578647951
AC134312.5_AC090673.1	0.716555782
AC134312.5_AL356417.2	0.631846707
AC134312.5_AC007750.1	0.752539161
AC134312.5_LINC01561	0.936390081
AC134312.5_LINC01833	0.121980265
AC134312.5_LINC00460	0.063136131
AC134312.5_LINC00536	0.406655276
AC134312.5_AP000553.1	0.838675094
AC134312.5_AATBC	0.019616239
AC134312.5_AL161431.1	0.24046929
AC134312.5_PICSAR	0.412656928
AC134312.5_LINC01615	0.727275395
AC134312.5_LINC00641	0.026855154
AC134312.5_MIR133A1HG	0.142562995
AC134312.5_AC104984.6	0.860968626
AC134312.5_AL023755.1	0.10522879
AC134312.5_AL359851.1	0.148899893
AC134312.5_AC015923.1	0.125021791
AC134312.5_AC005828.1	0.512762846
AC134312.5_AC023034.1	0.281194476
AC134312.5_AC099811.5	0.934973694
AC134312.5_FP325330.3	0.030834402
AC134312.5_AL355075.4	0.725642761
AC134312.5_RMRP	0.380301559
AC134312.5_AC037487.2	0.963332066

AC134312.5_AC006141.1	0.225586226
AC134312.5_LINC02443	0.084396373
AC134312.5_MIR181A1HG	0.288040645
AC134312.5_LINC02465	0.402206696
AC134312.5_AC091906.1	0.030752994
AC090825.1_ADAMTS9-AS1	0.02016342
AC090825.1_MEF2C-AS1	0.885512926
AC090825.1_LINC01778	0.605436048
AC090825.1_AF001548.1	0.950745118
AC090825.1_AC002398.2	0.032520618
AC090825.1_AL662860.1	0.538835595
AC090825.1_AC079313.1	0.822712622
AC090825.1_AC131097.4	0.469643304
AC090825.1_AC092376.2	0.479821551
AC090825.1_AC053503.4	0.248969359
AC090825.1_AC027449.1	0.2867515
AC090825.1_AP001189.1	0.178828911
AC090825.1_AC005180.1	0.893475554
AC090825.1_FENDRR	0.067365142
AC090825.1_LINC02202	0.567456933
AC090825.1_AP003071.4	0.765751519
AC090825.1_AL365214.2	0.857739876
AC090825.1_ADAMTS9-AS2	0.739193991
AC090825.1_AP001107.5	0.236654816
AC090825.1_AC087521.1	0.561552417
AC090825.1_AC007671.1	0.582797052
AC090825.1_LINC01081	0.990374204
AC090825.1_HAND2-AS1	0.055236642
AC090825.1_AC005180.2	0.08771262
AC090825.1_LINC00702	0.340027833
AC090825.1_MBNL1-AS1	0.948103308
AC090825.1_LINC01614	0.28010282
AC090825.1_MIR100HG	0.548353355
AC090825.1_JAZF1-AS1	0.693882124
AC090825.1_AC022893.1	0.725182688
AC090825.1_ACTA2-AS1	0.254338325
AC090825.1_AP003071.3	0.293045291
AC090825.1_LINC01352	0.616956645
AC090825.1_AC134312.1	0.550843616
AC090825.1_AC091588.3	0.489174995
AC090825.1_HLX-AS1	0.152307781
AC090825.1_LINC00402	0.679861086
AC090825.1_LINC01781	0.381093886
AC090825.1_AC007128.1	0.683740355

AC090825.1_AC099850.3	0.455563488
AC090825.1_AC010655.2	0.439297027
AC090825.1_LINC00941	0.414743988
AC090825.1_MYOSLID	0.375139388
AC090825.1_AL161772.1	0.397639079
AC090825.1_AP005432.2	0.376311178
AC090825.1_AC099066.2	0.602958016
AC090825.1_CDKN2B-AS1	0.391953181
AC090825.1_AL449423.1	0.856201322
AC090825.1_AL445490.1	0.76115534
AC090825.1_AC004988.1	0.452654408
AC090825.1_LINC02195	0.05602311
AC090825.1_AC090125.1	0.204165185
AC090825.1_LINC01048	0.285260463
AC090825.1_AC090673.1	0.374859346
AC090825.1_AL356417.2	0.46895251
AC090825.1_AC007750.1	0.520697512
AC090825.1_LINC01561	0.889727645
AC090825.1_LINC01833	0.699885742
AC090825.1_LINC00460	0.746485717
AC090825.1_LINC00536	0.123254744
AC090825.1_AP000553.1	0.393356677
AC090825.1_AATBC	0.176733013
AC090825.1_AL161431.1	0.217790534
AC090825.1_PICSAR	0.123674284
AC090825.1_LINC01615	0.503401216
AC090825.1_LINC00641	0.72050813
AC090825.1_MIR133A1HG	0.900955918
AC090825.1_AC104984.6	0.080519977
AC090825.1_AL023755.1	0.69006097
AC090825.1_AL359851.1	0.436358609
AC090825.1_AC015923.1	0.156243596
AC090825.1_AC005828.1	0.102279219
AC090825.1_AC023034.1	0.909761309
AC090825.1_AC099811.5	0.404901092
AC090825.1_FP325330.3	0.478580064
AC090825.1_AL355075.4	0.642428697
AC090825.1_RMRP	0.815443498
AC090825.1_AC037487.2	0.225096235
AC090825.1_AC006141.1	0.53300053
AC090825.1_LINC02443	0.330809649
AC090825.1_MIR181A1HG	0.842200873
AC090825.1_LINC02465	0.869433758
AC090825.1_AC091906.1	0.738186748

ADAMTS9-AS1_MEF2C-AS1	0.318446127
ADAMTS9-AS1_LINC01778	0.001366957
ADAMTS9-AS1_AF001548.1	0.137127726
ADAMTS9-AS1_AC002398.2	0.622038179
ADAMTS9-AS1_AL662860.1	0.111040948
ADAMTS9-AS1_AC079313.1	0.211380945
ADAMTS9-AS1_AC131097.4	0.122898383
ADAMTS9-AS1_AC092376.2	0.03298668
ADAMTS9-AS1_AC053503.4	0.043191427
ADAMTS9-AS1_AC027449.1	0.654529407
ADAMTS9-AS1_AP001189.1	0.064921015
ADAMTS9-AS1_AC005180.1	0.417470354
ADAMTS9-AS1_FENDRR	3.88E-05
ADAMTS9-AS1_LINC02202	0.04217057
ADAMTS9-AS1_AP003071.4	0.031041713
ADAMTS9-AS1_AL365214.2	0.011295549
ADAMTS9-AS1_ADAMTS9-AS2	0.019996703
ADAMTS9-AS1_AP001107.5	0.903518083
ADAMTS9-AS1_AC087521.1	0.005603343
ADAMTS9-AS1_AC007671.1	0.039938289
ADAMTS9-AS1_LINC01081	0.7241333
ADAMTS9-AS1_HAND2-AS1	0.012000188
ADAMTS9-AS1_AC005180.2	0.279362416
ADAMTS9-AS1_LINC00702	0.990848961
ADAMTS9-AS1_MBNL1-AS1	0.832396911
ADAMTS9-AS1_LINC01614	0.22407335
ADAMTS9-AS1_MIR100HG	0.684501966
ADAMTS9-AS1_JAZF1-AS1	0.101809995
ADAMTS9-AS1_AC022893.1	0.120336332
ADAMTS9-AS1_ACTA2-AS1	0.013625588
ADAMTS9-AS1_AP003071.3	0.893682222
ADAMTS9-AS1_LINC01352	0.125102241
ADAMTS9-AS1_AC134312.1	0.455398304
ADAMTS9-AS1_AC091588.3	0.204772535
ADAMTS9-AS1_HLX-AS1	0.843961726
ADAMTS9-AS1_LINC00402	0.016598139
ADAMTS9-AS1_LINC01781	0.132048941
ADAMTS9-AS1_AC007128.1	0.804593003
ADAMTS9-AS1_AC099850.3	0.618675112
ADAMTS9-AS1_AC010655.2	0.084106155
ADAMTS9-AS1_LINC00941	0.111764525
ADAMTS9-AS1_MYOSLID	0.110892503
ADAMTS9-AS1_AL161772.1	0.432249814
ADAMTS9-AS1_AP005432.2	0.153099544

ADAMTS9-AS1_AC099066.2	0.178755657
ADAMTS9-AS1_CDKN2B-AS1	0.003934597
ADAMTS9-AS1_AL449423.1	0.915227197
ADAMTS9-AS1_AL445490.1	0.013185293
ADAMTS9-AS1_U62317.2	0.28732889
ADAMTS9-AS1_AC004988.1	0.189766083
ADAMTS9-AS1_LINC02195	0.044791092
ADAMTS9-AS1_AC090125.1	0.800386593
ADAMTS9-AS1_LINC01048	0.433416926
ADAMTS9-AS1_AC090673.1	0.409664065
ADAMTS9-AS1_AL356417.2	0.474655397
ADAMTS9-AS1_AC007750.1	0.045045452
ADAMTS9-AS1_LINC01561	0.722614474
ADAMTS9-AS1_LINC01833	0.474891334
ADAMTS9-AS1_LINC00460	0.72629523
ADAMTS9-AS1_LINC00536	0.455171628
ADAMTS9-AS1_AP000553.1	0.62778984
ADAMTS9-AS1_AATBC	0.196725817
ADAMTS9-AS1_AL161431.1	0.031646944
ADAMTS9-AS1_PICSAR	0.041186206
ADAMTS9-AS1_LINC01615	0.449519195
ADAMTS9-AS1_LINC00641	0.462542288
ADAMTS9-AS1_MIR133A1HG	0.025240146
ADAMTS9-AS1_AC104984.6	0.090566422
ADAMTS9-AS1_AL023755.1	0.010961999
ADAMTS9-AS1_AL359851.1	0.014716402
ADAMTS9-AS1_AC015923.1	0.000394525
ADAMTS9-AS1_AC005828.1	0.017934383
ADAMTS9-AS1_AC023034.1	0.098364869
ADAMTS9-AS1_AC099811.5	0.347524781
ADAMTS9-AS1_FP325330.3	0.025670917
ADAMTS9-AS1_AL355075.4	0.060060313
ADAMTS9-AS1_RMRP	0.89987458
ADAMTS9-AS1_AC037487.2	0.621959874
ADAMTS9-AS1_AC006141.1	0.016389904
ADAMTS9-AS1_LINC02443	0.078846747
ADAMTS9-AS1_MIR181A1HG	0.073744292
ADAMTS9-AS1_LINC02465	0.059208915
ADAMTS9-AS1_AC091906.1	0.577494107
MEF2C-AS1_LINC01778	0.065150006
MEF2C-AS1_AF001548.1	0.4571515
MEF2C-AS1_AC002398.2	0.422038287
MEF2C-AS1_AL662860.1	0.904450989
MEF2C-AS1_AC079313.1	0.410736318

MEF2C-AS1_AC131097.4	0.524975932
MEF2C-AS1_AC092376.2	0.172441104
MEF2C-AS1_AC053503.4	0.059052181
MEF2C-AS1_AC027449.1	0.359827159
MEF2C-AS1_AP001189.1	0.578112328
MEF2C-AS1_AC005180.1	0.564560634
MEF2C-AS1_FENDRR	0.036562391
MEF2C-AS1_LINC02202	0.487477482
MEF2C-AS1_AP003071.4	0.816995348
MEF2C-AS1_AL365214.2	0.481275105
MEF2C-AS1_ADAMTS9-AS2	0.35543219
MEF2C-AS1_AP001107.5	0.291101882
MEF2C-AS1_AC087521.1	0.335321212
MEF2C-AS1_AC007671.1	0.684330077
MEF2C-AS1_LINC01081	0.547061059
MEF2C-AS1_HAND2-AS1	0.487770338
MEF2C-AS1_AC005180.2	0.1133898
MEF2C-AS1_LINC00702	0.98172785
MEF2C-AS1_MBNL1-AS1	0.597210342
MEF2C-AS1_LINC01614	0.136590541
MEF2C-AS1_MIR100HG	0.468303085
MEF2C-AS1_JAZF1-AS1	0.678383177
MEF2C-AS1_AC022893.1	0.805637853
MEF2C-AS1_ACTA2-AS1	0.66500251
MEF2C-AS1_AP003071.3	0.298560763
MEF2C-AS1_LINC01352	0.904509361
MEF2C-AS1_AC134312.1	0.685043062
MEF2C-AS1_AC091588.3	0.863337374
MEF2C-AS1_HLX-AS1	0.145394262
MEF2C-AS1_LINC00402	0.517072552
MEF2C-AS1_LINC01781	0.572066198
MEF2C-AS1_AC007128.1	0.82148536
MEF2C-AS1_AC099850.3	0.354835519
MEF2C-AS1_AC010655.2	0.813224906
MEF2C-AS1_LINC00941	0.535234512
MEF2C-AS1_MYOSLID	0.456886163
MEF2C-AS1_AL161772.1	0.102078965
MEF2C-AS1_AP005432.2	0.049366856
MEF2C-AS1_AC099066.2	0.809387779
MEF2C-AS1_CDKN2B-AS1	0.564918125
MEF2C-AS1_AL449423.1	0.841987658
MEF2C-AS1_AL445490.1	0.776927209
MEF2C-AS1_AC004988.1	0.61875144
MEF2C-AS1_LINC02195	0.820033407

MEF2C-AS1_AC090125.1	0.614237009
MEF2C-AS1_LINC01048	0.994715225
MEF2C-AS1_AC090673.1	0.14655895
MEF2C-AS1_AL356417.2	0.040987985
MEF2C-AS1_AC007750.1	0.865945061
MEF2C-AS1_LINC01561	0.252492638
MEF2C-AS1_LINC01833	0.685060481
MEF2C-AS1_LINC00460	0.084780932
MEF2C-AS1_LINC00536	0.143876848
MEF2C-AS1_AP000553.1	0.958325515
MEF2C-AS1_AATBC	0.766413365
MEF2C-AS1_AL161431.1	0.013016614
MEF2C-AS1_PICSAR	0.210169266
MEF2C-AS1_LINC01615	0.56067219
MEF2C-AS1_MIR133A1HG	0.432150827
MEF2C-AS1_AC104984.6	0.246697362
MEF2C-AS1_AL023755.1	0.076202839
MEF2C-AS1_AL359851.1	0.128325256
MEF2C-AS1_AC015923.1	0.082028764
MEF2C-AS1_AC005828.1	0.260148481
MEF2C-AS1_AC023034.1	0.968679157
MEF2C-AS1_AC099811.5	0.287221112
MEF2C-AS1_FP325330.3	0.487867565
MEF2C-AS1_AL355075.4	0.041832463
MEF2C-AS1_RMRP	0.661577041
MEF2C-AS1_AC037487.2	0.20026512
MEF2C-AS1_AC006141.1	0.500630064
MEF2C-AS1_LINC02443	0.249253002
MEF2C-AS1_MIR181A1HG	0.817638799
MEF2C-AS1_LINC02465	0.41892673
MEF2C-AS1_AC091906.1	0.914128012
LINC01778_AF001548.1	0.096844467
LINC01778_AC002398.2	0.023604986
LINC01778_AL662860.1	0.162746791
LINC01778_AC079313.1	0.962526521
LINC01778_AC131097.4	0.85372158
LINC01778_AC092376.2	0.438323842
LINC01778_AC053503.4	0.003155965
LINC01778_AC027449.1	0.573408462
LINC01778_AP001189.1	0.155218612
LINC01778_AC005180.1	0.107215044
LINC01778_FENDRR	0.827918752
LINC01778_LINC02202	0.72156064
LINC01778_AP003071.4	0.135295579

LINC01778_AL365214.2	0.963174866
LINC01778_ADAMTS9-AS2	0.047765765
LINC01778_AP001107.5	0.057867526
LINC01778_AC087521.1	0.466357528
LINC01778_AC007671.1	0.612523673
LINC01778_LINC01081	0.215775835
LINC01778_HAND2-AS1	0.0958909
LINC01778_AC005180.2	0.669469124
LINC01778_LINC00702	0.343346369
LINC01778_MBNL1-AS1	0.564661434
LINC01778_LINC01614	0.084469631
LINC01778_MIR100HG	0.158150625
LINC01778_JAZF1-AS1	0.30125581
LINC01778_AC022893.1	0.142970034
LINC01778_ACTA2-AS1	0.290968414
LINC01778_AP003071.3	0.163325606
LINC01778_LINC01352	0.502121508
LINC01778_AC134312.1	0.080498382
LINC01778_AC091588.3	0.934930777
LINC01778_HLX-AS1	0.221282512
LINC01778_LINC00402	0.748615074
LINC01778_LINC01781	0.056725184
LINC01778_AC007128.1	0.719143987
LINC01778_AC099850.3	0.42748968
LINC01778_AC010655.2	0.912357638
LINC01778_LINC00941	0.287629653
LINC01778_MYOSLID	0.559125048
LINC01778_AL161772.1	0.48903308
LINC01778_AP005432.2	0.353185766
LINC01778_AC099066.2	0.1505472
LINC01778_CDKN2B-AS1	0.424040319
LINC01778_AL449423.1	0.919012058
LINC01778_AL445490.1	0.85411246
LINC01778_AC004988.1	0.55690523
LINC01778_LINC02195	0.636377106
LINC01778_AC090125.1	0.158309939
LINC01778_LINC01048	0.181073864
LINC01778_AC090673.1	0.021819268
LINC01778_AL356417.2	0.322443647
LINC01778_AC007750.1	0.157020563
LINC01778_LINC01561	0.154209048
LINC01778_LINC01833	0.725780447
LINC01778_LINC00460	0.344980139
LINC01778_LINC00536	0.020103606

LINC01778_AP000553.1	0.139291374
LINC01778_AATBC	0.179877982
LINC01778_AL161431.1	0.092416383
LINC01778_PICSAR	0.291614498
LINC01778_LINC01615	0.679948355
LINC01778_LINC00641	0.909384629
LINC01778_MIR133A1HG	0.104854912
LINC01778_AC104984.6	0.629121492
LINC01778_AL023755.1	0.997408288
LINC01778_AL359851.1	0.136072448
LINC01778_AC015923.1	0.218343277
LINC01778_AC005828.1	0.494623872
LINC01778_AC023034.1	0.494991011
LINC01778_AC099811.5	0.291061135
LINC01778_FP325330.3	0.624572713
LINC01778_AL355075.4	0.876073286
LINC01778_RMRP	0.502740553
LINC01778_AC037487.2	0.292339488
LINC01778_AC006141.1	0.474543523
LINC01778_LINC02443	0.830632822
LINC01778_MIR181A1HG	0.054509541
LINC01778_LINC02465	0.137963973
LINC01778_AC091906.1	0.888558803
AF001548.1_AC002398.2	0.154551533
AF001548.1_AL662860.1	0.4113311
AF001548.1_AC079313.1	0.773505573
AF001548.1_AC131097.4	0.763200283
AF001548.1_AC092376.2	0.261931031
AF001548.1_AC053503.4	0.048692653
AF001548.1_AC027449.1	0.514345981
AF001548.1_AP001189.1	0.024584129
AF001548.1_AC005180.1	0.067364075
AF001548.1_FENDRR	0.834752491
AF001548.1_LINC02202	0.420992952
AF001548.1_AP003071.4	0.809168584
AF001548.1_AL365214.2	0.205473285
AF001548.1_ADAMTS9-AS2	0.192802263
AF001548.1_AP001107.5	0.239096465
AF001548.1_AC087521.1	0.118812578
AF001548.1_AC007671.1	0.14882377
AF001548.1_LINC01081	0.398623697
AF001548.1_HAND2-AS1	0.118742469
AF001548.1_AC005180.2	0.788473304
AF001548.1_LINC00702	0.024720561

AF001548.1_MBNL1-AS1	0.025336532
AF001548.1_LINC01614	0.195408443
AF001548.1_MIR100HG	0.455631198
AF001548.1_JAZF1-AS1	0.633982556
AF001548.1_AC022893.1	0.167443052
AF001548.1_ACTA2-AS1	0.849538916
AF001548.1_AP003071.3	0.682005626
AF001548.1_LINC01352	0.28823178
AF001548.1_AC134312.1	0.338086175
AF001548.1_AC091588.3	0.324189779
AF001548.1_HLX-AS1	0.266468569
AF001548.1_LINC00402	0.703142961
AF001548.1_LINC01781	0.516889356
AF001548.1_AC007128.1	0.922992866
AF001548.1_AC099850.3	0.384656464
AF001548.1_AC010655.2	0.221262939
AF001548.1_LINC00941	0.881359235
AF001548.1_MYOSLID	0.862891497
AF001548.1_AL161772.1	0.822010299
AF001548.1_AP005432.2	0.416869157
AF001548.1_AC099066.2	0.436869722
AF001548.1_CDKN2B-AS1	0.943485704
AF001548.1_AL449423.1	0.582525262
AF001548.1_AL445490.1	0.533555275
AF001548.1_U62317.2	0.588845637
AF001548.1_AC004988.1	0.854582204
AF001548.1_LINC02195	0.009937329
AF001548.1_AC090125.1	0.455985911
AF001548.1_LINC01048	0.112985327
AF001548.1_AC090673.1	0.106221244
AF001548.1_AL356417.2	0.364140851
AF001548.1_AC007750.1	0.770472141
AF001548.1_LINC01561	0.392197332
AF001548.1_LINC01833	0.210451904
AF001548.1_LINC00460	0.367245483
AF001548.1_LINC00536	0.17865152
AF001548.1_AP000553.1	0.652120319
AF001548.1_AATBC	0.168634779
AF001548.1_AL161431.1	0.083532423
AF001548.1_PICSAR	0.282944566
AF001548.1_LINC01615	0.705754223
AF001548.1_LINC00641	0.146057192
AF001548.1_MIR133A1HG	0.569242884
AF001548.1_AC104984.6	0.87238891

AF001548.1_AL023755.1	0.661371763
AF001548.1_AL359851.1	0.450479501
AF001548.1_AC015923.1	0.531239914
AF001548.1_AC005828.1	0.460882723
AF001548.1_AC023034.1	0.503318126
AF001548.1_AC099811.5	0.190201073
AF001548.1_FP325330.3	0.943261239
AF001548.1_AL355075.4	0.703801897
AF001548.1_RMRP	0.467018627
AF001548.1_AC037487.2	0.512010871
AF001548.1_AC006141.1	0.882793134
AF001548.1_LINC02443	0.661756945
AF001548.1_MIR181A1HG	0.199304759
AF001548.1_LINC02465	0.471367279
AF001548.1_AC091906.1	0.312820039
AC002398.2_AL662860.1	0.814784934
AC002398.2_AC079313.1	0.986345769
AC002398.2_AC131097.4	0.195097262
AC002398.2_AC092376.2	0.299834183
AC002398.2_AC053503.4	0.930704482
AC002398.2_AC027449.1	0.64128444
AC002398.2_AP001189.1	0.777006522
AC002398.2_AC005180.1	0.935312788
AC002398.2_FENDRR	0.001382599
AC002398.2_LINC02202	0.037622404
AC002398.2_AP003071.4	0.017232783
AC002398.2_AL365214.2	0.149880878
AC002398.2_ADAMTS9-AS2	0.881215869
AC002398.2_AP001107.5	0.474833839
AC002398.2_AC087521.1	0.051351777
AC002398.2_AC007671.1	0.308763045
AC002398.2_LINC01081	0.133300299
AC002398.2_HAND2-AS1	0.395149864
AC002398.2_AC005180.2	0.000364432
AC002398.2_LINC00702	0.819597589
AC002398.2_MBNL1-AS1	0.001106669
AC002398.2_LINC01614	0.715704019
AC002398.2_MIR100HG	0.937684519
AC002398.2_JAZF1-AS1	0.519104142
AC002398.2_AC022893.1	0.639062476
AC002398.2_ACTA2-AS1	0.116659109
AC002398.2_AP003071.3	0.207286386
AC002398.2_LINC01352	0.639357353
AC002398.2_AC134312.1	0.410756151

AC002398.2_AC091588.3	0.67666041
AC002398.2_HLX-AS1	0.65497934
AC002398.2_LINC00402	0.194307218
AC002398.2_LINC01781	0.269677833
AC002398.2_AC007128.1	0.648493576
AC002398.2_AC099850.3	0.14257109
AC002398.2_AC010655.2	0.118416382
AC002398.2_LINC00941	0.269681298
AC002398.2_MYOSLID	0.497871912
AC002398.2_AL161772.1	0.529310516
AC002398.2_AP005432.2	0.753543448
AC002398.2_AC099066.2	0.567641465
AC002398.2_CDKN2B-AS1	0.183987094
AC002398.2_AL449423.1	0.78597945
AC002398.2_AL445490.1	0.127779132
AC002398.2_U62317.2	0.367784104
AC002398.2_AC004988.1	0.019794017
AC002398.2_LINC02195	0.048975702
AC002398.2_AC090125.1	0.737878297
AC002398.2_LINC01048	0.314654589
AC002398.2_AC090673.1	0.84989208
AC002398.2_AL356417.2	0.918075021
AC002398.2_AC007750.1	0.119904525
AC002398.2_LINC01561	0.588224127
AC002398.2_LINC01833	0.03312568
AC002398.2_LINC00460	0.238821139
AC002398.2_LINC00536	0.388946844
AC002398.2_AP000553.1	0.175284519
AC002398.2_AATBC	0.125539218
AC002398.2_AL161431.1	0.644678499
AC002398.2_PICSAR	0.188108509
AC002398.2_LINC01615	0.371073068
AC002398.2_LINC00641	0.003590573
AC002398.2_MIR133A1HG	0.617202727
AC002398.2_AC104984.6	0.277695037
AC002398.2_AL023755.1	0.564408192
AC002398.2_AL359851.1	0.165155662
AC002398.2_AC015923.1	0.26998138
AC002398.2_AC005828.1	0.185818332
AC002398.2_AC023034.1	0.71879255
AC002398.2_AC099811.5	0.555012099
AC002398.2_FP325330.3	0.129450759
AC002398.2_AL355075.4	0.096277167
AC002398.2_RMRP	0.591121417

AC002398.2_AC037487.2	0.041638362
AC002398.2_AC006141.1	0.305117895
AC002398.2_LINC02443	0.392120655
AC002398.2_MIR181A1HG	0.906232949
AC002398.2_LINC02465	0.959100658
AC002398.2_AC091906.1	0.392414043
AL662860.1_AC079313.1	0.571808147
AL662860.1_AC131097.4	0.307785097
AL662860.1_AC092376.2	0.811440383
AL662860.1_AC053503.4	0.383346517
AL662860.1_AC027449.1	0.942017893
AL662860.1_AP001189.1	0.964576645
AL662860.1_AC005180.1	0.204892926
AL662860.1_FENDRR	0.074492035
AL662860.1_LINC02202	0.295276017
AL662860.1_AP003071.4	0.37331442
AL662860.1_AL365214.2	0.410936382
AL662860.1_ADAMTS9-AS2	0.404717036
AL662860.1_AP001107.5	0.396532307
AL662860.1_AC087521.1	0.816131979
AL662860.1_AC007671.1	0.378209979
AL662860.1_LINC01081	0.927322936
AL662860.1_HAND2-AS1	0.701428741
AL662860.1_AC005180.2	0.918715214
AL662860.1_LINC00702	0.086601526
AL662860.1_MBNL1-AS1	0.035116812
AL662860.1_LINC01614	0.093696727
AL662860.1_MIR100HG	0.946732623
AL662860.1_JAZF1-AS1	0.23487975
AL662860.1_AC022893.1	0.459497933
AL662860.1_ACTA2-AS1	0.052687528
AL662860.1_AP003071.3	0.907495856
AL662860.1_LINC01352	0.732456225
AL662860.1_AC134312.1	0.668557394
AL662860.1_AC091588.3	0.44927037
AL662860.1_HLX-AS1	0.689301723
AL662860.1_LINC00402	0.055934504
AL662860.1_LINC01781	0.559014743
AL662860.1_AC007128.1	0.929423187
AL662860.1_AC099850.3	0.252496528
AL662860.1_AC010655.2	0.35771531
AL662860.1_LINC00941	0.634954584
AL662860.1_MYOSLID	0.435539814
AL662860.1_AL161772.1	0.576205751

AL662860.1_AP005432.2	0.159901515
AL662860.1_AC099066.2	0.391949506
AL662860.1_CDKN2B-AS1	0.057893939
AL662860.1_AL449423.1	0.732818075
AL662860.1_AL445490.1	0.251070321
AL662860.1_AC004988.1	0.069052582
AL662860.1_LINC02195	0.779618874
AL662860.1_AC090125.1	0.894879312
AL662860.1_LINC01048	0.839805999
AL662860.1_AC090673.1	0.531298912
AL662860.1_AL356417.2	0.67380993
AL662860.1_AC007750.1	0.537466192
AL662860.1_LINC01561	0.370575331
AL662860.1_LINC01833	0.049398704
AL662860.1_LINC00460	0.583116481
AL662860.1_LINC00536	0.534645102
AL662860.1_AP000553.1	0.211381717
AL662860.1_AATBC	0.2389535
AL662860.1_AL161431.1	0.306559893
AL662860.1_PICSAR	0.423591929
AL662860.1_LINC01615	0.313161427
AL662860.1_LINC00641	0.446484182
AL662860.1_MIR133A1HG	0.299032444
AL662860.1_AC104984.6	0.086789475
AL662860.1_AL023755.1	0.354392888
AL662860.1_AL359851.1	0.443511684
AL662860.1_AC015923.1	0.171606206
AL662860.1_AC005828.1	0.381574906
AL662860.1_AC023034.1	0.8125485
AL662860.1_AC099811.5	0.652016538
AL662860.1_FP325330.3	0.08012111
AL662860.1_AL355075.4	0.236660644
AL662860.1_RMRP	0.570360163
AL662860.1_AC037487.2	0.82110052
AL662860.1_AC006141.1	0.212299534
AL662860.1_LINC02443	0.104650787
AL662860.1_MIR181A1HG	0.172183199
AL662860.1_LINC02465	0.809837465
AL662860.1_AC091906.1	0.5632545
AC079313.1_AC131097.4	0.95796516
AC079313.1_AC092376.2	0.783626601
AC079313.1_AC053503.4	0.153079731
AC079313.1_AC027449.1	0.238474918
AC079313.1_AP001189.1	0.08569226

AC079313.1_AC005180.1	0.573627244
AC079313.1_FENDRR	0.557840209
AC079313.1_LINC02202	0.533965226
AC079313.1_AP003071.4	0.182374271
AC079313.1_AL365214.2	0.885667439
AC079313.1_ADAMTS9-AS2	0.87389792
AC079313.1_AP001107.5	0.098688945
AC079313.1_AC087521.1	0.002319309
AC079313.1_AC007671.1	0.719228018
AC079313.1_LINC01081	0.644773373
AC079313.1_HAND2-AS1	0.620456159
AC079313.1_AC005180.2	0.694719507
AC079313.1_LINC00702	0.074080917
AC079313.1_MBNL1-AS1	0.862391639
AC079313.1_LINC01614	0.191032483
AC079313.1_MIR100HG	0.25075032
AC079313.1_JAZF1-AS1	0.666797463
AC079313.1_AC022893.1	0.89789541
AC079313.1_ACTA2-AS1	0.538708356
AC079313.1_AP003071.3	0.023244385
AC079313.1_LINC01352	0.220284906
AC079313.1_AC134312.1	0.746034464
AC079313.1_AC091588.3	0.185264651
AC079313.1_HLX-AS1	0.461592608
AC079313.1_LINC00402	0.855374924
AC079313.1_LINC01781	0.695187199
AC079313.1_AC007128.1	0.420204163
AC079313.1_AC099850.3	0.423840304
AC079313.1_AC010655.2	0.777491809
AC079313.1_LINC00941	0.709438355
AC079313.1_MYOSLID	0.306568711
AC079313.1_AL161772.1	0.273774071
AC079313.1_AP005432.2	0.099719095
AC079313.1_AC099066.2	0.226820058
AC079313.1_CDKN2B-AS1	0.920077285
AC079313.1_AL449423.1	0.847866001
AC079313.1_AL445490.1	0.642814393
AC079313.1_AC004988.1	0.872973877
AC079313.1_LINC02195	0.116120626
AC079313.1_AC090125.1	0.791953273
AC079313.1_LINC01048	0.225874192
AC079313.1_AC090673.1	0.318966457
AC079313.1_AL356417.2	0.160412505
AC079313.1_AC007750.1	0.281455908

AC079313.1_LINC01561	0.476993122
AC079313.1_LINC01833	0.282183691
AC079313.1_LINC00460	0.621019191
AC079313.1_LINC00536	0.249793726
AC079313.1_AP000553.1	0.724615502
AC079313.1_AATBC	0.679415198
AC079313.1_AL161431.1	0.022242395
AC079313.1_PICSAR	0.720573796
AC079313.1_LINC01615	0.027388326
AC079313.1_LINC00641	0.421588317
AC079313.1_MIR133A1HG	0.486395441
AC079313.1_AC104984.6	0.798639076
AC079313.1_AL023755.1	0.902106237
AC079313.1_AL359851.1	0.506884359
AC079313.1_AC015923.1	0.821554051
AC079313.1_AC005828.1	0.655280053
AC079313.1_AC023034.1	0.987380662
AC079313.1_AC099811.5	0.271702758
AC079313.1_FP325330.3	0.948540711
AC079313.1_AL355075.4	0.853730648
AC079313.1_RMRP	0.984444592
AC079313.1_AC037487.2	0.975046352
AC079313.1_AC006141.1	0.953551831
AC079313.1_LINC02443	0.97186011
AC079313.1_MIR181A1HG	0.607841613
AC079313.1_LINC02465	0.500264641
AC079313.1_AC091906.1	0.76445069
AC131097.4_AC092376.2	0.904973214
AC131097.4_AC053503.4	0.926795731
AC131097.4_AC027449.1	0.807132391
AC131097.4_AP001189.1	0.225937424
AC131097.4_AC005180.1	0.371092869
AC131097.4_FENDRR	0.790169121
AC131097.4_LINC02202	0.162228848
AC131097.4_AP003071.4	0.930199347
AC131097.4_AL365214.2	0.948028954
AC131097.4_ADAMTS9-AS2	0.954138715
AC131097.4_AP001107.5	0.370522714
AC131097.4_AC087521.1	0.108248475
AC131097.4_AC007671.1	0.699423519
AC131097.4_LINC01081	0.944726028
AC131097.4_HAND2-AS1	0.30065805
AC131097.4_AC005180.2	0.601935716
AC131097.4_LINC00702	0.062593242

AC131097.4_MBNL1-AS1	0.181087018
AC131097.4_LINC01614	0.961647309
AC131097.4_MIR100HG	0.992300503
AC131097.4_JAZF1-AS1	0.764115243
AC131097.4_AC022893.1	0.370699798
AC131097.4_ACTA2-AS1	0.872316104
AC131097.4_AP003071.3	0.180566549
AC131097.4_LINC01352	0.136496014
AC131097.4_AC134312.1	0.869653409
AC131097.4_AC091588.3	0.51290208
AC131097.4_HLX-AS1	0.361114451
AC131097.4_LINC00402	0.342033634
AC131097.4_LINC01781	0.505413287
AC131097.4_AC007128.1	0.072552707
AC131097.4_AC010655.2	0.97398649
AC131097.4_LINC00941	0.904317069
AC131097.4_MYOSLID	0.737517045
AC131097.4_AL161772.1	0.963104091
AC131097.4_AP005432.2	0.428449927
AC131097.4_AC099066.2	0.753682749
AC131097.4_CDKN2B-AS1	0.753849994
AC131097.4_AL449423.1	0.506499611
AC131097.4_AL445490.1	0.852824385
AC131097.4_AC004988.1	0.344012102
AC131097.4_LINC02195	0.808748702
AC131097.4_AC090125.1	0.328803424
AC131097.4_LINC01048	0.269190277
AC131097.4_AC090673.1	0.817624658
AC131097.4_AL356417.2	0.531340719
AC131097.4_AC007750.1	0.928471593
AC131097.4_LINC01561	0.986151584
AC131097.4_LINC01833	0.713402005
AC131097.4_LINC00460	0.597409692
AC131097.4_LINC00536	0.561408078
AC131097.4_AP000553.1	0.782514258
AC131097.4_AATBC	0.909377935
AC131097.4_AL161431.1	0.247906112
AC131097.4_PICSAR	0.842393823
AC131097.4_LINC01615	0.478749775
AC131097.4_LINC00641	0.628183204
AC131097.4_MIR133A1HG	0.498523284
AC131097.4_AC104984.6	0.518952205
AC131097.4_AL023755.1	0.480102167
AC131097.4_AL359851.1	0.675007599

AC131097.4_AC015923.1	0.536880732
AC131097.4_AC005828.1	0.627333681
AC131097.4_AC023034.1	0.538315634
AC131097.4_AC099811.5	0.923477252
AC131097.4_FP325330.3	0.475561997
AC131097.4_AL355075.4	0.683487479
AC131097.4_RMRP	0.797320953
AC131097.4_AC037487.2	0.836215858
AC131097.4_AC006141.1	0.419349434
AC131097.4_LINC02443	0.301258701
AC131097.4_MIR181A1HG	0.730952273
AC131097.4_LINC02465	0.753459712
AC131097.4_AC091906.1	0.907365434
AC092376.2_AC053503.4	0.038051344
AC092376.2_AC027449.1	0.441352881
AC092376.2_AP001189.1	0.226534907
AC092376.2_AC005180.1	0.10626079
AC092376.2_FENDRR	0.048815355
AC092376.2_LINC02202	0.69053074
AC092376.2_AP003071.4	0.728513702
AC092376.2_AL365214.2	0.160210576
AC092376.2_ADAMTS9-AS2	0.435286387
AC092376.2_AP001107.5	0.057599454
AC092376.2_AC087521.1	0.483003518
AC092376.2_AC007671.1	0.691594997
AC092376.2_LINC01081	0.870246675
AC092376.2_HAND2-AS1	0.518270133
AC092376.2_AC005180.2	0.750413325
AC092376.2_LINC00702	0.013337431
AC092376.2_MBNL1-AS1	0.952018416
AC092376.2_LINC01614	0.010696189
AC092376.2_MIR100HG	0.303716302
AC092376.2_JAZF1-AS1	0.923157735
AC092376.2_AC022893.1	0.883767148
AC092376.2_ACTA2-AS1	0.910315616
AC092376.2_AP003071.3	0.597869504
AC092376.2_LINC01352	0.469375881
AC092376.2_AC134312.1	0.850764801
AC092376.2_AC091588.3	0.264293698
AC092376.2_HLX-AS1	0.058514642
AC092376.2_LINC00402	0.14066552
AC092376.2_LINC01781	0.771114754
AC092376.2_AC007128.1	0.754959868
AC092376.2_AC099850.3	0.455563488

AC092376.2_AC010655.2	0.320064203
AC092376.2_LINC00941	0.749000507
AC092376.2_MYOSLID	0.769186521
AC092376.2_AL161772.1	0.334114038
AC092376.2_AP005432.2	0.12684622
AC092376.2_AC099066.2	0.662094339
AC092376.2_CDKN2B-AS1	0.342415066
AC092376.2_AL449423.1	0.729909199
AC092376.2_AL445490.1	0.575196441
AC092376.2_AC004988.1	0.495498937
AC092376.2_LINC02195	0.833532452
AC092376.2_AC090125.1	0.271449174
AC092376.2_LINC01048	0.878031798
AC092376.2_AC090673.1	0.22570361
AC092376.2_AL356417.2	0.415295216
AC092376.2_AC007750.1	0.985115857
AC092376.2_LINC01561	0.892453029
AC092376.2_LINC01833	0.448571005
AC092376.2_LINC00460	0.192769133
AC092376.2_LINC00536	0.013033774
AC092376.2_AP000553.1	0.854537355
AC092376.2_AATBC	0.840074916
AC092376.2_AL161431.1	0.01079213
AC092376.2_PICSAR	0.284224285
AC092376.2_LINC01615	0.056908792
AC092376.2_LINC00641	0.87590619
AC092376.2_MIR133A1HG	0.974422483
AC092376.2_AC104984.6	0.145086676
AC092376.2_AL023755.1	0.132636697
AC092376.2_AL359851.1	0.333712992
AC092376.2_AC015923.1	0.002788356
AC092376.2_AC005828.1	0.524800988
AC092376.2_AC023034.1	0.617411194
AC092376.2_AC099811.5	0.89895824
AC092376.2_FP325330.3	0.035609894
AC092376.2_AL355075.4	0.182654236
AC092376.2_RMRP	0.435043779
AC092376.2_AC037487.2	0.912423438
AC092376.2_AC006141.1	0.1526914
AC092376.2_LINC02443	0.043442294
AC092376.2_MIR181A1HG	0.435693682
AC092376.2_LINC02465	0.261452256
AC092376.2_AC091906.1	0.439589758
AC053503.4_AC027449.1	0.033562638

AC053503.4_AP001189.1	0.131050324
AC053503.4_AC005180.1	0.167344468
AC053503.4_FENDRR	0.06478271
AC053503.4_LINC02202	0.028802921
AC053503.4_AP003071.4	0.021746418
AC053503.4_AL365214.2	0.024798539
AC053503.4_ADAMTS9-AS2	0.036783831
AC053503.4_AP001107.5	0.335677879
AC053503.4_AC087521.1	0.094135658
AC053503.4_AC007671.1	0.10563071
AC053503.4_LINC01081	0.116287223
AC053503.4_HAND2-AS1	0.056533042
AC053503.4_AC005180.2	0.369277825
AC053503.4_LINC00702	0.122369981
AC053503.4_MBNL1-AS1	0.2970184
AC053503.4_LINC01614	0.464482388
AC053503.4_MIR100HG	0.795040953
AC053503.4_JAZF1-AS1	0.062440494
AC053503.4_AC022893.1	0.327130732
AC053503.4_ACTA2-AS1	0.262977643
AC053503.4_AP003071.3	0.02734237
AC053503.4_LINC01352	0.02080177
AC053503.4_AC134312.1	0.046594928
AC053503.4_AC091588.3	0.036783831
AC053503.4_HLX-AS1	0.056533778
AC053503.4_LINC00402	0.031229738
AC053503.4_LINC01781	0.028484497
AC053503.4_AC007128.1	0.337494897
AC053503.4_AC099850.3	0.422382946
AC053503.4_AC010655.2	0.262385508
AC053503.4_LINC00941	0.462623997
AC053503.4_MYOSLID	0.528496632
AC053503.4_AL161772.1	0.16453608
AC053503.4_AP005432.2	0.319381856
AC053503.4_AC099066.2	0.241457544
AC053503.4_CDKN2B-AS1	0.01856066
AC053503.4_AL449423.1	0.077458678
AC053503.4_AL445490.1	0.119040208
AC053503.4_U62317.2	0.359200184
AC053503.4_AC004988.1	0.017057804
AC053503.4_LINC02195	0.038845467
AC053503.4_AC090125.1	0.07958997
AC053503.4_LINC01048	0.041118493
AC053503.4_AC090673.1	0.033794585

AC053503.4_AL356417.2	0.089518584
AC053503.4_AC007750.1	0.085297175
AC053503.4_LINC01561	0.104753749
AC053503.4_LINC01833	0.058630333
AC053503.4_LINC00460	0.029887548
AC053503.4_LINC00536	0.075527273
AC053503.4_AP000553.1	0.03185524
AC053503.4_AATBC	0.103652928
AC053503.4_AL161431.1	0.159710112
AC053503.4_PICSAR	0.021070691
AC053503.4_LINC01615	0.39818183
AC053503.4_LINC00641	0.684522217
AC053503.4_MIR133A1HG	0.046941096
AC053503.4_AC104984.6	0.046347334
AC053503.4_AL023755.1	0.050358332
AC053503.4_AL359851.1	0.03185524
AC053503.4_AC015923.1	0.007450892
AC053503.4_AC005828.1	0.021283143
AC053503.4_AC023034.1	0.090279109
AC053503.4_AC099811.5	0.048361052
AC053503.4_FP325330.3	0.036717397
AC053503.4_AL355075.4	0.217734804
AC053503.4_RMRP	0.031720807
AC053503.4_AC037487.2	0.039227272
AC053503.4_AC006141.1	0.046941096
AC053503.4_LINC02443	0.070645069
AC053503.4_MIR181A1HG	0.03185524
AC053503.4_LINC02465	0.028494321
AC053503.4_AC091906.1	0.068046385
AC027449.1_AP001189.1	0.969359855
AC027449.1_AC005180.1	0.541512642
AC027449.1_FENDRR	0.213253427
AC027449.1_LINC02202	0.157158749
AC027449.1_AP003071.4	0.690929574
AC027449.1_AL365214.2	0.643244524
AC027449.1_ADAMTS9-AS2	0.786345002
AC027449.1_AP001107.5	0.847706756
AC027449.1_AC087521.1	0.270611731
AC027449.1_AC007671.1	0.781636607
AC027449.1_LINC01081	0.907452947
AC027449.1_HAND2-AS1	0.888707291
AC027449.1_AC005180.2	0.027167439
AC027449.1_LINC00702	0.834589963
AC027449.1_MBNL1-AS1	0.0033679

AC027449.1_LINC01614	0.567565169
AC027449.1_MIR100HG	0.345596356
AC027449.1_JAZF1-AS1	0.501419079
AC027449.1_AC022893.1	0.440931517
AC027449.1_ACTA2-AS1	0.030065783
AC027449.1_AP003071.3	0.500833474
AC027449.1_LINC01352	0.680072888
AC027449.1_AC134312.1	0.975664791
AC027449.1_AC091588.3	0.359987301
AC027449.1_HLX-AS1	0.969251771
AC027449.1_LINC00402	0.434534288
AC027449.1_LINC01781	0.269941674
AC027449.1_AC007128.1	0.351195321
AC027449.1_AC099850.3	0.252496528
AC027449.1_AC010655.2	0.572090649
AC027449.1_LINC00941	0.805916037
AC027449.1_MYOSLID	0.876130975
AC027449.1_AL161772.1	0.745710476
AC027449.1_AP005432.2	0.170622536
AC027449.1_AC099066.2	0.768244527
AC027449.1_CDKN2B-AS1	0.244845656
AC027449.1_AL449423.1	0.681442601
AC027449.1_AL445490.1	0.847634744
AC027449.1_AC004988.1	0.109980578
AC027449.1_LINC02195	0.936336363
AC027449.1_AC090125.1	0.697756458
AC027449.1_LINC01048	0.732408462
AC027449.1_AC090673.1	0.455672122
AC027449.1_AL356417.2	0.795212019
AC027449.1_AC007750.1	0.230339186
AC027449.1_LINC01561	0.179186361
AC027449.1_LINC01833	0.699722077
AC027449.1_LINC00460	0.871307784
AC027449.1_LINC00536	0.831940662
AC027449.1_AP000553.1	0.514449325
AC027449.1_AATBC	0.554468717
AC027449.1_AL161431.1	0.613180583
AC027449.1_PICSAR	0.608731994
AC027449.1_LINC01615	0.40137923
AC027449.1_LINC00641	0.004055522
AC027449.1_MIR133A1HG	0.696644505
AC027449.1_AC104984.6	0.712101249
AC027449.1_AL023755.1	0.807256044
AC027449.1_AL359851.1	0.632664453

AC027449.1_AC015923.1	0.517922611
AC027449.1_AC005828.1	0.389488048
AC027449.1_AC023034.1	0.472401361
AC027449.1_AC099811.5	0.889003489
AC027449.1_FP325330.3	0.361547229
AC027449.1_AL355075.4	0.299926111
AC027449.1_RMRP	0.852591304
AC027449.1_AC037487.2	0.263687222
AC027449.1_AC006141.1	0.617990489
AC027449.1_LINC02443	0.401354349
AC027449.1_MIR181A1HG	0.577008606
AC027449.1_LINC02465	0.600463598
AC027449.1_AC091906.1	0.472612817
AP001189.1_AC005180.1	0.851670053
AP001189.1_FENDRR	0.000631882
AP001189.1_LINC02202	0.050866447
AP001189.1_AP003071.4	0.036773771
AP001189.1_AL365214.2	0.107703899
AP001189.1_ADAMTS9-AS2	0.519859826
AP001189.1_AP001107.5	0.567936186
AP001189.1_AC087521.1	0.133196445
AP001189.1_AC007671.1	0.22125544
AP001189.1_LINC01081	0.418296766
AP001189.1_HAND2-AS1	0.686680367
AP001189.1_AC005180.2	0.021696711
AP001189.1_LINC00702	0.799078088
AP001189.1_MBNL1-AS1	0.160587205
AP001189.1_LINC01614	0.302662001
AP001189.1_MIR100HG	0.94893032
AP001189.1_JAZF1-AS1	0.215184778
AP001189.1_AC022893.1	0.095175932
AP001189.1_ACTA2-AS1	0.001936308
AP001189.1_AP003071.3	0.945197562
AP001189.1_LINC01352	0.851836834
AP001189.1_AC134312.1	0.179709069
AP001189.1_AC091588.3	0.038742935
AP001189.1_HLX-AS1	0.490301888
AP001189.1_LINC00402	0.12811287
AP001189.1_LINC01781	0.1133463
AP001189.1_AC007128.1	0.836675686
AP001189.1_AC099850.3	0.644717504
AP001189.1_AC010655.2	0.113354086
AP001189.1_LINC00941	0.298278683
AP001189.1_MYOSLID	0.399311883

AP001189.1_AL161772.1	0.343329608
AP001189.1_AP005432.2	0.040852505
AP001189.1_AC099066.2	0.820811036
AP001189.1_CDKN2B-AS1	0.156484617
AP001189.1_AL449423.1	0.266464323
AP001189.1_AL445490.1	0.091326021
AP001189.1_AC004988.1	0.034067213
AP001189.1_LINC02195	0.103363356
AP001189.1_AC090125.1	0.846178034
AP001189.1_LINC01048	0.69550371
AP001189.1_AC090673.1	0.375214336
AP001189.1_AL356417.2	0.88465714
AP001189.1_AC007750.1	0.368393042
AP001189.1_LINC01561	0.366936915
AP001189.1_LINC01833	0.091899494
AP001189.1_LINC00460	0.981438025
AP001189.1_LINC00536	0.923610935
AP001189.1_AP000553.1	0.255361296
AP001189.1_AATBC	0.06224941
AP001189.1_AL161431.1	0.127030619
AP001189.1_PICSAR	0.01227403
AP001189.1_LINC01615	0.787719302
AP001189.1_LINC00641	0.983203483
AP001189.1_MIR133A1HG	0.16463405
AP001189.1_AC104984.6	0.030166416
AP001189.1_AL023755.1	0.000536995
AP001189.1_AL359851.1	0.086027872
AP001189.1_AC015923.1	0.000318328
AP001189.1_AC005828.1	0.021701792
AP001189.1_AC023034.1	0.07221574
AP001189.1_AC099811.5	0.120390424
AP001189.1_FP325330.3	0.004273688
AP001189.1_AL355075.4	0.06058411
AP001189.1_RMRP	0.455525087
AP001189.1_AC037487.2	0.177105897
AP001189.1_AC006141.1	0.101055919
AP001189.1_LINC02443	0.016756909
AP001189.1_MIR181A1HG	0.249465222
AP001189.1_LINC02465	0.28628842
AP001189.1_AC091906.1	0.219848763
AC005180.1_FENDRR	0.029272498
AC005180.1_LINC02202	0.497968564
AC005180.1_AP003071.4	0.288583439
AC005180.1_AL365214.2	0.135157964

AC005180.1_ADAMTS9-AS2	0.340659364
AC005180.1_AP001107.5	0.96995151
AC005180.1_AC087521.1	0.708213173
AC005180.1_AC007671.1	0.307671818
AC005180.1_LINC01081	0.294258679
AC005180.1_HAND2-AS1	0.497286744
AC005180.1_AC005180.2	0.140991472
AC005180.1_LINC00702	0.960817019
AC005180.1_MBNL1-AS1	0.676113599
AC005180.1_LINC01614	0.430553198
AC005180.1_MIR100HG	0.760850405
AC005180.1_JAZF1-AS1	0.129175098
AC005180.1_AC022893.1	0.491121249
AC005180.1_ACTA2-AS1	0.094730671
AC005180.1_AP003071.3	0.901003289
AC005180.1_LINC01352	0.201814014
AC005180.1_AC134312.1	0.216052157
AC005180.1_AC091588.3	0.103192315
AC005180.1_HLX-AS1	0.436601898
AC005180.1_LINC00402	0.242257577
AC005180.1_LINC01781	0.621444055
AC005180.1_AC007128.1	0.10535408
AC005180.1_AC099850.3	0.522742125
AC005180.1_AC010655.2	0.479133762
AC005180.1_LINC00941	0.737502721
AC005180.1_MYOSLID	0.878116044
AC005180.1_AL161772.1	0.438216945
AC005180.1_AP005432.2	0.168835456
AC005180.1_AC099066.2	0.94386231
AC005180.1_CDKN2B-AS1	0.257151029
AC005180.1_AL449423.1	0.825120695
AC005180.1_AL445490.1	0.296408608
AC005180.1_U62317.2	0.580688581
AC005180.1_AC004988.1	0.039866378
AC005180.1_LINC02195	0.021387026
AC005180.1_AC090125.1	0.499880289
AC005180.1_LINC01048	0.417874479
AC005180.1_AC090673.1	0.494876654
AC005180.1_AL356417.2	0.146380002
AC005180.1_AC007750.1	0.67169056
AC005180.1_LINC01561	0.577413269
AC005180.1_LINC01833	0.020274411
AC005180.1_LINC00460	0.969688109
AC005180.1_LINC00536	0.606454208

AC005180.1_AP000553.1	0.271759922
AC005180.1_AATBC	0.520656244
AC005180.1_AL161431.1	0.432114644
AC005180.1_PICSAR	0.072699757
AC005180.1_LINC01615	0.579456744
AC005180.1_LINC00641	0.085522869
AC005180.1_MIR133A1HG	0.065497753
AC005180.1_AC104984.6	0.345994756
AC005180.1_AL023755.1	0.023254183
AC005180.1_AL359851.1	0.09759399
AC005180.1_AC015923.1	0.084220557
AC005180.1_AC005828.1	0.028582527
AC005180.1_AC023034.1	0.124645207
AC005180.1_AC099811.5	0.086544183
AC005180.1_FP325330.3	0.020508315
AC005180.1_AL355075.4	0.385539255
AC005180.1_RMRP	0.199582629
AC005180.1_AC037487.2	0.282124626
AC005180.1_AC006141.1	0.072960601
AC005180.1_LINC02443	0.043141112
AC005180.1_MIR181A1HG	0.216400394
AC005180.1_LINC02465	0.185837817
AC005180.1_AC091906.1	0.453331735
FENDRR_LINC02202	0.012671088
FENDRR_AP003071.4	0.40587425
FENDRR_AL365214.2	0.861164789
FENDRR_ADAMTS9-AS2	3.39E-05
FENDRR_AP001107.5	0.018534026
FENDRR_AC087521.1	0.010250782
FENDRR_AC007671.1	0.005850909
FENDRR_LINC01081	2.73E-05
FENDRR_HAND2-AS1	0.136183873
FENDRR_AC005180.2	0.197037144
FENDRR_LINC00702	0.411397725
FENDRR_MBNL1-AS1	0.762712448
FENDRR_LINC01614	0.048089044
FENDRR_MIR100HG	0.02868353
FENDRR_JAZF1-AS1	0.000896037
FENDRR_AC022893.1	0.667185217
FENDRR_ACTA2-AS1	0.613671748
FENDRR_AP003071.3	0.000184962
FENDRR_LINC01352	0.030153523
FENDRR_AC134312.1	0.004036641
FENDRR_AC091588.3	0.00050388

FENDRR_HLX-AS1	0.445200574
FENDRR_LINC00402	7.20E-05
FENDRR_LINC01781	0.274515594
FENDRR_AC007128.1	0.884431713
FENDRR_AC099850.3	0.96667354
FENDRR_AC010655.2	0.409426866
FENDRR_LINC00941	0.869310824
FENDRR_MYOSLID	0.057190105
FENDRR_AL161772.1	0.687625692
FENDRR_AP005432.2	0.889097257
FENDRR_AC099066.2	0.109503915
FENDRR_CDKN2B-AS1	0.892978067
FENDRR_AL449423.1	0.230472739
FENDRR_AL445490.1	0.504166627
FENDRR_U62317.2	0.179228514
FENDRR_AC004988.1	0.807593242
FENDRR_LINC02195	0.108690552
FENDRR_AC090125.1	0.607466548
FENDRR_LINC01048	0.032488947
FENDRR_AC090673.1	0.175773701
FENDRR_AL356417.2	0.805677917
FENDRR_AC007750.1	0.403360804
FENDRR_LINC01561	0.374027592
FENDRR_LINC01833	0.152169497
FENDRR_LINC00460	0.220332652
FENDRR_LINC00536	1.01E-06
FENDRR_AP000553.1	0.285680776
FENDRR_AATBC	0.019889822
FENDRR_AL161431.1	0.186859163
FENDRR_PICSAR	0.907138608
FENDRR_LINC01615	0.449930849
FENDRR_LINC00641	0.577956043
FENDRR_MIR133A1HG	0.590459737
FENDRR_AC104984.6	0.357929328
FENDRR_AL023755.1	0.873004321
FENDRR_AL359851.1	0.530134944
FENDRR_AC015923.1	0.504257847
FENDRR_AC005828.1	0.851040808
FENDRR_AC023034.1	0.907724744
FENDRR_AC099811.5	0.374458363
FENDRR_FP325330.3	0.718569481
FENDRR_AL355075.4	0.727533842
FENDRR_RMRP	0.515013081
FENDRR_AC037487.2	0.839103977

FENDRR_AC006141.1	0.358348474
FENDRR_LINC02443	0.801071329
FENDRR_MIR181A1HG	0.431689074
FENDRR_LINC02465	0.46777787
FENDRR_AC091906.1	0.527059979
LINC02202_AP003071.4	0.747012903
LINC02202_AL365214.2	0.436467008
LINC02202_ADAMTS9-AS2	0.733975091
LINC02202_AP001107.5	0.350868755
LINC02202_AC087521.1	0.05266201
LINC02202_AC007671.1	0.25730576
LINC02202_LINC01081	0.91230324
LINC02202_HAND2-AS1	0.310883673
LINC02202_AC005180.2	0.215354856
LINC02202_LINC00702	0.112153849
LINC02202_MBNL1-AS1	0.352100278
LINC02202_LINC01614	0.066291057
LINC02202_MIR100HG	0.295462304
LINC02202_JAZF1-AS1	0.953988017
LINC02202_AC022893.1	0.95581014
LINC02202_ACTA2-AS1	0.104013974
LINC02202_AP003071.3	0.468576148
LINC02202_LINC01352	0.383992445
LINC02202_AC134312.1	0.055373943
LINC02202_AC091588.3	0.934036304
LINC02202_HLX-AS1	0.03186106
LINC02202_LINC00402	0.945730965
LINC02202_LINC01781	0.476415539
LINC02202_AC007128.1	0.51726612
LINC02202_AC099850.3	0.906830357
LINC02202_AC010655.2	0.438441136
LINC02202_LINC00941	0.367407985
LINC02202_MYOSLID	0.566148641
LINC02202_AL161772.1	0.768379194
LINC02202_AP005432.2	0.068151804
LINC02202_AC099066.2	0.538486349
LINC02202_CDKN2B-AS1	0.385689036
LINC02202_AL449423.1	0.376933989
LINC02202_AL445490.1	0.576924226
LINC02202_AC004988.1	0.569488837
LINC02202_LINC02195	0.927429274
LINC02202_AC090125.1	0.856254179
LINC02202_LINC01048	0.265521003
LINC02202_AC090673.1	0.049377404

LINC02202_AL356417.2	0.147693366
LINC02202_AC007750.1	0.484643662
LINC02202_LINC01561	0.716097865
LINC02202_LINC01833	0.469580731
LINC02202_LINC00460	0.088135562
LINC02202_LINC00536	0.001590365
LINC02202_AP000553.1	0.718296865
LINC02202_AATBC	0.217709327
LINC02202_AL161431.1	0.024882407
LINC02202_PICSAR	0.883909363
LINC02202_LINC01615	0.075341886
LINC02202_LINC00641	0.682683364
LINC02202_MIR133A1HG	0.666809743
LINC02202_AC104984.6	0.482344785
LINC02202_AL023755.1	0.213788512
LINC02202_AL359851.1	0.468264546
LINC02202_AC015923.1	0.239403725
LINC02202_AC005828.1	0.269874447
LINC02202_AC023034.1	0.849310251
LINC02202_AC099811.5	0.663615699
LINC02202_FP325330.3	0.074518069
LINC02202_AL355075.4	0.99267725
LINC02202_RMRP	0.152004967
LINC02202_AC037487.2	0.658254612
LINC02202_AC006141.1	0.483724505
LINC02202_LINC02443	0.759704957
LINC02202_MIR181A1HG	0.061707089
LINC02202_LINC02465	0.787604746
LINC02202_AC091906.1	0.322105239
AP003071.4_AL365214.2	0.632769045
AP003071.4_ADAMTS9-AS2	0.525053456
AP003071.4_AP001107.5	0.476209068
AP003071.4_AC087521.1	0.654394467
AP003071.4_AC007671.1	0.281469952
AP003071.4_LINC01081	0.809651984
AP003071.4_HAND2-AS1	0.680540265
AP003071.4_AC005180.2	0.580220312
AP003071.4_LINC00702	0.346097114
AP003071.4_MBNL1-AS1	0.518190335
AP003071.4_LINC01614	0.177046998
AP003071.4_MIR100HG	0.15884277
AP003071.4_JAZF1-AS1	0.605834372
AP003071.4_AC022893.1	0.212239895
AP003071.4_ACTA2-AS1	0.529447922

AP003071.4_AP003071.3	0.604903333
AP003071.4_LINC01352	0.640134849
AP003071.4_AC134312.1	0.936590205
AP003071.4_AC091588.3	0.56426
AP003071.4_HLX-AS1	0.873831601
AP003071.4_LINC00402	0.65175286
AP003071.4_LINC01781	0.842968753
AP003071.4_AC007128.1	0.181148236
AP003071.4_AC099850.3	0.287260412
AP003071.4_AC010655.2	0.218287538
AP003071.4_LINC00941	0.809732923
AP003071.4_MYOSLID	0.816535203
AP003071.4_AL161772.1	0.562090097
AP003071.4_AP005432.2	0.214703867
AP003071.4_AC099066.2	0.497182033
AP003071.4_CDKN2B-AS1	0.0677543
AP003071.4_AL449423.1	0.404542886
AP003071.4_AL445490.1	0.390034599
AP003071.4_U62317.2	0.654244003
AP003071.4_AC004988.1	0.330701413
AP003071.4_LINC02195	0.001140007
AP003071.4_AC090125.1	0.705990342
AP003071.4_LINC01048	0.178454119
AP003071.4_AC090673.1	0.801361758
AP003071.4_AL356417.2	0.368745029
AP003071.4_AC007750.1	0.490595552
AP003071.4_LINC01561	0.625859425
AP003071.4_LINC01833	0.433129766
AP003071.4_LINC00460	0.200448155
AP003071.4_LINC00536	0.027281411
AP003071.4_AP000553.1	0.968290623
AP003071.4_AATBC	0.1750806
AP003071.4_AL161431.1	0.173027778
AP003071.4_PICSAR	0.739979701
AP003071.4_LINC01615	0.358817065
AP003071.4_LINC00641	0.171618996
AP003071.4_MIR133A1HG	0.724474223
AP003071.4_AC104984.6	0.579431624
AP003071.4_AL023755.1	0.339020498
AP003071.4_AL359851.1	0.653611451
AP003071.4_AC015923.1	0.622095207
AP003071.4_AC005828.1	0.314839508
AP003071.4_AC023034.1	0.429710728
AP003071.4_AC099811.5	0.520646522

AP003071.4_FP325330.3	0.415937052
AP003071.4_AL355075.4	0.518648884
AP003071.4_RMRP	0.624066146
AP003071.4_AC037487.2	0.841598041
AP003071.4_AC006141.1	0.986840353
AP003071.4_LINC02443	0.108780576
AP003071.4_MIR181A1HG	0.62640617
AP003071.4_LINC02465	0.537908702
AP003071.4_AC091906.1	0.170649341
AL365214.2_ADAMTS9-AS2	0.010502468
AL365214.2_AP001107.5	0.220937224
AL365214.2_AC087521.1	0.812048215
AL365214.2_AC007671.1	0.256302924
AL365214.2_LINC01081	0.762716565
AL365214.2_HAND2-AS1	0.439871736
AL365214.2_AC005180.2	0.318570228
AL365214.2_LINC00702	0.029646164
AL365214.2_MBNL1-AS1	0.796217503
AL365214.2_LINC01614	0.01458588
AL365214.2_MIR100HG	0.196026208
AL365214.2_JAZF1-AS1	0.7340583
AL365214.2_AC022893.1	0.715553839
AL365214.2_ACTA2-AS1	0.337416201
AL365214.2_AP003071.3	0.337039149
AL365214.2_LINC01352	0.033728758
AL365214.2_AC134312.1	0.691687736
AL365214.2_AC091588.3	0.192634789
AL365214.2_HLX-AS1	0.241790264
AL365214.2_LINC00402	0.236134819
AL365214.2_LINC01781	0.721605693
AL365214.2_AC007128.1	0.866583137
AL365214.2_AC099850.3	0.455563488
AL365214.2_AC010655.2	0.897492231
AL365214.2_LINC00941	0.199785658
AL365214.2_MYOSLID	0.544118695
AL365214.2_AL161772.1	0.322482152
AL365214.2_AP005432.2	0.015323956
AL365214.2_AC099066.2	0.914362547
AL365214.2_CDKN2B-AS1	0.321194886
AL365214.2_AL449423.1	0.851701597
AL365214.2_AL445490.1	0.919513895
AL365214.2_AC004988.1	0.787523676
AL365214.2_LINC02195	0.708168141
AL365214.2_AC090125.1	0.81376453

AL365214.2_LINC01048	0.416105397
AL365214.2_AC090673.1	0.10679925
AL365214.2_AL356417.2	0.238252491
AL365214.2_AC007750.1	0.856733732
AL365214.2_LINC01561	0.288226375
AL365214.2_LINC01833	0.431872721
AL365214.2_LINC00460	0.420600745
AL365214.2_LINC00536	0.218632265
AL365214.2_AP000553.1	0.546382236
AL365214.2_AATBC	0.67205468
AL365214.2_AL161431.1	0.090432287
AL365214.2_PICSAR	0.572085928
AL365214.2_LINC01615	0.999714547
AL365214.2_MIR133A1HG	0.660602377
AL365214.2_AC104984.6	0.374075015
AL365214.2_AL023755.1	0.232796333
AL365214.2_AL359851.1	0.321704564
AL365214.2_AC015923.1	0.273976536
AL365214.2_AC005828.1	0.757333014
AL365214.2_AC023034.1	0.750921315
AL365214.2_AC099811.5	0.711499084
AL365214.2_FP325330.3	0.982594949
AL365214.2_AL355075.4	0.294143975
AL365214.2_RMRP	0.442553803
AL365214.2_AC037487.2	0.651481689
AL365214.2_AC006141.1	0.786152289
AL365214.2_LINC02443	0.435052657
AL365214.2_MIR181A1HG	0.999769781
AL365214.2_LINC02465	0.871323098
AL365214.2_AC091906.1	0.967694956
ADAMTS9-AS2_AP001107.5	0.365526802
ADAMTS9-AS2_AC087521.1	0.583084998
ADAMTS9-AS2_AC007671.1	0.767490057
ADAMTS9-AS2_LINC01081	0.958373351
ADAMTS9-AS2_HAND2-AS1	0.325133134
ADAMTS9-AS2_AC005180.2	0.09381287
ADAMTS9-AS2_LINC00702	0.947207267
ADAMTS9-AS2_MBNL1-AS1	0.598319018
ADAMTS9-AS2_LINC01614	0.090082681
ADAMTS9-AS2_MIR100HG	0.664434957
ADAMTS9-AS2_JAZF1-AS1	0.466060399
ADAMTS9-AS2_AC022893.1	0.042916227
ADAMTS9-AS2_ACTA2-AS1	0.37497638
ADAMTS9-AS2_AP003071.3	0.423351593

ADAMTS9-AS2_LINC01352	0.63021347
ADAMTS9-AS2_AC134312.1	0.948162458
ADAMTS9-AS2_AC091588.3	0.808178995
ADAMTS9-AS2_HLX-AS1	0.69283778
ADAMTS9-AS2_LINC00402	0.034997027
ADAMTS9-AS2_LINC01781	0.39664473
ADAMTS9-AS2_AC007128.1	0.633165528
ADAMTS9-AS2_AC099850.3	0.455563488
ADAMTS9-AS2_AC010655.2	0.646435378
ADAMTS9-AS2_LINC00941	0.580626262
ADAMTS9-AS2_MYOSLID	0.325097457
ADAMTS9-AS2_AL161772.1	0.010622551
ADAMTS9-AS2_AP005432.2	0.01407251
ADAMTS9-AS2_AC099066.2	0.935107054
ADAMTS9-AS2_CDKN2B-AS1	0.019092721
ADAMTS9-AS2_AL449423.1	0.798189403
ADAMTS9-AS2_AL445490.1	0.232323907
ADAMTS9-AS2_AC004988.1	0.797684414
ADAMTS9-AS2_LINC02195	0.887470266
ADAMTS9-AS2_AC090125.1	0.828504318
ADAMTS9-AS2_LINC01048	0.630254559
ADAMTS9-AS2_AC090673.1	0.041155643
ADAMTS9-AS2_AL356417.2	0.086354791
ADAMTS9-AS2_AC007750.1	0.57727731
ADAMTS9-AS2_LINC01561	0.249780929
ADAMTS9-AS2_LINC01833	0.3425317
ADAMTS9-AS2_LINC00460	0.306209991
ADAMTS9-AS2_LINC00536	0.152550595
ADAMTS9-AS2_AP000553.1	0.80823478
ADAMTS9-AS2_AATBC	0.637835938
ADAMTS9-AS2_AL161431.1	0.324461477
ADAMTS9-AS2_PICSAR	0.152233543
ADAMTS9-AS2_LINC01615	0.684180759
ADAMTS9-AS2_MIR133A1HG	0.182218844
ADAMTS9-AS2_AC104984.6	0.301532617
ADAMTS9-AS2_AL023755.1	0.149563504
ADAMTS9-AS2_AL359851.1	0.205173469
ADAMTS9-AS2_AC015923.1	0.07472606
ADAMTS9-AS2_AC005828.1	0.420805752
ADAMTS9-AS2_AC023034.1	0.422263478
ADAMTS9-AS2_AC099811.5	0.278260052
ADAMTS9-AS2_FP325330.3	0.1362966
ADAMTS9-AS2_AL355075.4	0.001710951
ADAMTS9-AS2_RMRP	0.872425045

ADAMTS9-AS2_AC037487.2	0.242094938
ADAMTS9-AS2_AC006141.1	0.220052478
ADAMTS9-AS2_LINC02443	0.335615436
ADAMTS9-AS2_MIR181A1HG	0.973690378
ADAMTS9-AS2_LINC02465	0.639876584
ADAMTS9-AS2_AC091906.1	0.841088876
AP001107.5_AC087521.1	0.275149345
AP001107.5_AC007671.1	0.381310871
AP001107.5_LINC01081	0.288390616
AP001107.5_HAND2-AS1	0.585433903
AP001107.5_AC005180.2	0.402163442
AP001107.5_LINC00702	0.410845513
AP001107.5_MBNL1-AS1	0.029128338
AP001107.5_LINC01614	0.28331758
AP001107.5_MIR100HG	0.667465446
AP001107.5_JAZF1-AS1	0.393288508
AP001107.5_AC022893.1	0.298647479
AP001107.5_ACTA2-AS1	0.042617542
AP001107.5_AP003071.3	0.410752631
AP001107.5_LINC01352	0.197916733
AP001107.5_AC134312.1	0.206256283
AP001107.5_AC091588.3	0.130194274
AP001107.5_HLX-AS1	0.462903885
AP001107.5_LINC00402	0.124706812
AP001107.5_LINC01781	0.117304782
AP001107.5_AC007128.1	0.426713748
AP001107.5_AC099850.3	0.413637046
AP001107.5_AC010655.2	0.136102806
AP001107.5_LINC00941	0.194953009
AP001107.5_MYOSLID	0.564066661
AP001107.5_AL161772.1	0.952051837
AP001107.5_AP005432.2	0.817835506
AP001107.5_AC099066.2	0.532530402
AP001107.5_CDKN2B-AS1	0.163774329
AP001107.5_AL449423.1	0.226274687
AP001107.5_AL445490.1	0.318625848
AP001107.5_U62317.2	0.509906012
AP001107.5_AC004988.1	0.011532424
AP001107.5_LINC02195	0.161649548
AP001107.5_AC090125.1	0.757227166
AP001107.5_LINC01048	0.691415936
AP001107.5_AC090673.1	0.556530879
AP001107.5_AL356417.2	0.416806949
AP001107.5_AC007750.1	0.134343476

AP001107.5_LINC01561	0.301735532
AP001107.5_LINC01833	0.272425244
AP001107.5_LINC00460	0.185653855
AP001107.5_LINC00536	0.954008681
AP001107.5_AP000553.1	0.184596425
AP001107.5_AATBC	0.055543402
AP001107.5_AL161431.1	0.538141844
AP001107.5_PICSAR	0.033935963
AP001107.5_LINC01615	0.375358425
AP001107.5_LINC00641	0.287617379
AP001107.5_MIR133A1HG	0.192673879
AP001107.5_AC104984.6	0.188460448
AP001107.5_AL023755.1	0.026061421
AP001107.5_AL359851.1	0.130944292
AP001107.5_AC015923.1	0.087774559
AP001107.5_AC005828.1	0.105212071
AP001107.5_AC023034.1	0.171354349
AP001107.5_AC099811.5	0.62496653
AP001107.5_FP325330.3	0.007187964
AP001107.5_AL355075.4	0.133633294
AP001107.5_RMRP	0.068405112
AP001107.5_AC037487.2	0.356556783
AP001107.5_AC006141.1	0.155104327
AP001107.5_LINC02443	0.019247164
AP001107.5_MIR181A1HG	0.370098506
AP001107.5_LINC02465	0.271627992
AP001107.5_AC091906.1	0.136246628
AC087521.1_AC007671.1	0.287152274
AC087521.1_LINC01081	0.201701093
AC087521.1_HAND2-AS1	0.387241688
AC087521.1_AC005180.2	0.810631697
AC087521.1_LINC00702	0.652865809
AC087521.1_MBNL1-AS1	0.296945179
AC087521.1_LINC01614	0.112623579
AC087521.1_MIR100HG	0.829805915
AC087521.1_JAZF1-AS1	0.085034959
AC087521.1_AC022893.1	0.183366826
AC087521.1_ACTA2-AS1	0.146037175
AC087521.1_AP003071.3	0.819281425
AC087521.1_LINC01352	0.257514849
AC087521.1_AC134312.1	0.609320312
AC087521.1_AC091588.3	0.466266558
AC087521.1_HLX-AS1	0.681607852
AC087521.1_LINC00402	0.927215287

AC087521.1_LINC01781	0.777473287
AC087521.1_AC007128.1	0.297457336
AC087521.1_AC099850.3	0.69047262
AC087521.1_AC010655.2	0.042989831
AC087521.1_LINC00941	0.834639825
AC087521.1_MYOSLID	0.413923665
AC087521.1_AL161772.1	0.972370056
AC087521.1_AP005432.2	0.258416766
AC087521.1_AC099066.2	0.290611232
AC087521.1_CDKN2B-AS1	0.361634618
AC087521.1_AL449423.1	0.534692615
AC087521.1_AL445490.1	0.28288204
AC087521.1_AC004988.1	0.32262317
AC087521.1_LINC02195	0.092248227
AC087521.1_AC090125.1	0.534033067
AC087521.1_LINC01048	0.110114189
AC087521.1_AC090673.1	0.046964699
AC087521.1_AL356417.2	0.281211732
AC087521.1_AC007750.1	0.688371502
AC087521.1_LINC01561	0.611645624
AC087521.1_LINC01833	0.356160477
AC087521.1_LINC00460	0.517075808
AC087521.1_LINC00536	0.055505801
AC087521.1_AP000553.1	0.542592439
AC087521.1_AATBC	0.000401669
AC087521.1_AL161431.1	0.19633382
AC087521.1_PICSAR	0.409528572
AC087521.1_LINC01615	0.115455214
AC087521.1_LINC00641	0.798612329
AC087521.1_MIR133A1HG	0.434867318
AC087521.1_AC104984.6	0.284357917
AC087521.1_AL023755.1	0.03842862
AC087521.1_AL359851.1	0.930056647
AC087521.1_AC015923.1	0.119563544
AC087521.1_AC005828.1	0.547115046
AC087521.1_AC023034.1	0.775826419
AC087521.1_AC099811.5	0.64645666
AC087521.1_FP325330.3	0.141655606
AC087521.1_AL355075.4	0.901007533
AC087521.1_RMRP	0.119352194
AC087521.1_AC037487.2	0.918318223
AC087521.1_AC006141.1	0.967764449
AC087521.1_LINC02443	0.394295792
AC087521.1_MIR181A1HG	0.517764912

AC087521.1_LINC02465	0.404487391
AC087521.1_AC091906.1	0.699926068
AC007671.1_LINC01081	0.709226203
AC007671.1_HAND2-AS1	0.778859857
AC007671.1_AC005180.2	0.018091289
AC007671.1_LINC00702	0.386404589
AC007671.1_MBNL1-AS1	0.488742872
AC007671.1_LINC01614	0.678539464
AC007671.1_MIR100HG	0.746111226
AC007671.1_JAZF1-AS1	0.90126998
AC007671.1_AC022893.1	0.187765325
AC007671.1_ACTA2-AS1	0.028096069
AC007671.1_AP003071.3	0.447556219
AC007671.1_LINC01352	0.978449218
AC007671.1_AC134312.1	0.496814339
AC007671.1_AC091588.3	0.591423437
AC007671.1_HLX-AS1	0.740820201
AC007671.1_LINC00402	0.570679749
AC007671.1_LINC01781	0.42338373
AC007671.1_AC007128.1	0.288551143
AC007671.1_AC099850.3	0.252496528
AC007671.1_AC010655.2	0.831579469
AC007671.1_LINC00941	0.786874425
AC007671.1_MYOSLID	0.557152429
AC007671.1_AL161772.1	0.159239273
AC007671.1_AP005432.2	0.08492922
AC007671.1_AC099066.2	0.940736654
AC007671.1_CDKN2B-AS1	0.228993576
AC007671.1_AL449423.1	0.951589683
AC007671.1_AL445490.1	0.009600635
AC007671.1_AC004988.1	0.411848315
AC007671.1_LINC02195	0.52454051
AC007671.1_AC090125.1	0.813485716
AC007671.1_LINC01048	0.488478222
AC007671.1_AC090673.1	0.525804309
AC007671.1_AL356417.2	0.406061677
AC007671.1_AC007750.1	0.449718906
AC007671.1_LINC01561	0.511888092
AC007671.1_LINC01833	0.269861378
AC007671.1_LINC00460	0.306368646
AC007671.1_LINC00536	0.772635452
AC007671.1_AP000553.1	0.519390524
AC007671.1_AATBC	0.760140517
AC007671.1_AL161431.1	0.170827941

AC007671.1_PICSAR	0.393719776
AC007671.1_LINC01615	0.235917442
AC007671.1_LINC00641	0.030515299
AC007671.1_MIR133A1HG	0.665015207
AC007671.1_AC104984.6	0.324449186
AC007671.1_AL023755.1	0.6026906
AC007671.1_AL359851.1	0.563340967
AC007671.1_AC015923.1	0.21543533
AC007671.1_AC005828.1	0.308612731
AC007671.1_AC023034.1	0.196807458
AC007671.1_AC099811.5	0.380496393
AC007671.1_FP325330.3	0.293207013
AC007671.1_AL355075.4	0.655837491
AC007671.1_RMRP	0.87784687
AC007671.1_AC037487.2	0.677714534
AC007671.1_AC006141.1	0.760087866
AC007671.1_LINC02443	0.320364228
AC007671.1_MIR181A1HG	0.354000408
AC007671.1_LINC02465	0.745279212
AC007671.1_AC091906.1	0.72188931
LINC01081_HAND2-AS1	0.840929747
LINC01081_AC005180.2	0.615360435
LINC01081_LINC00702	0.54711769
LINC01081_MBNL1-AS1	0.722377359
LINC01081_LINC01614	0.768570155
LINC01081_MIR100HG	0.240657266
LINC01081_JAZF1-AS1	0.908497862
LINC01081_AC022893.1	0.51602388
LINC01081_ACTA2-AS1	0.408825025
LINC01081_AP003071.3	0.624333592
LINC01081_LINC01352	0.436737383
LINC01081_AC134312.1	0.708322891
LINC01081_AC091588.3	0.512690397
LINC01081_HLX-AS1	0.498293387
LINC01081_LINC00402	0.51904086
LINC01081_LINC01781	0.551582382
LINC01081_AC007128.1	0.989395943
LINC01081_AC099850.3	0.252496528
LINC01081_AC010655.2	0.541889261
LINC01081_LINC00941	0.561841978
LINC01081_MYOSLID	0.594574628
LINC01081_AL161772.1	0.921956416
LINC01081_AP005432.2	0.135607318
LINC01081_AC099066.2	0.387876329

LINC01081_CDKN2B-AS1	0.457742983
LINC01081_AL449423.1	0.904538632
LINC01081_AL445490.1	0.964447843
LINC01081_AC004988.1	0.593827345
LINC01081_LINC02195	0.884841164
LINC01081_AC090125.1	0.744758507
LINC01081_LINC01048	0.584591713
LINC01081_AC090673.1	0.62586036
LINC01081_AL356417.2	0.759912998
LINC01081_AC007750.1	0.379441004
LINC01081_LINC01561	0.845041363
LINC01081_LINC01833	0.741780509
LINC01081_LINC00460	0.586020897
LINC01081_LINC00536	0.58490819
LINC01081_AP000553.1	0.873296522
LINC01081_AATBC	0.668113912
LINC01081_AL161431.1	0.048090775
LINC01081_PICSAR	0.324123654
LINC01081_LINC01615	0.286338612
LINC01081_LINC00641	0.546197772
LINC01081_MIR133A1HG	0.733284743
LINC01081_AC104984.6	0.548622384
LINC01081_AL023755.1	0.841826778
LINC01081_AL359851.1	0.932953608
LINC01081_AC015923.1	0.163376366
LINC01081_AC005828.1	0.367501994
LINC01081_AC023034.1	0.994818616
LINC01081_AC099811.5	0.389712824
LINC01081_FP325330.3	0.729158576
LINC01081_AL355075.4	0.425343276
LINC01081_RMRP	0.966059974
LINC01081_AC037487.2	0.618312705
LINC01081_AC006141.1	0.686114683
LINC01081_LINC02443	0.916356473
LINC01081_MIR181A1HG	0.875071906
LINC01081_LINC02465	0.774816606
LINC01081_AC091906.1	0.171168369
HAND2-AS1_AC005180.2	0.709051923
HAND2-AS1_LINC00702	0.5309984
HAND2-AS1_MBNL1-AS1	0.877570439
HAND2-AS1_LINC01614	0.043256196
HAND2-AS1_MIR100HG	0.533888111
HAND2-AS1_JAZF1-AS1	0.749737047
HAND2-AS1_AC022893.1	0.88351325

HAND2-AS1_ACTA2-AS1	0.094892827
HAND2-AS1_AP003071.3	0.441132552
HAND2-AS1_LINC01352	0.603903144
HAND2-AS1_AC134312.1	0.303000764
HAND2-AS1_AC091588.3	0.91939511
HAND2-AS1_HLX-AS1	0.705463033
HAND2-AS1_LINC00402	0.891499519
HAND2-AS1_LINC01781	0.358447877
HAND2-AS1_AC007128.1	0.482859089
HAND2-AS1_AC099850.3	0.389368705
HAND2-AS1_AC010655.2	0.357519678
HAND2-AS1_LINC00941	0.806760336
HAND2-AS1_MYOSLID	0.582982386
HAND2-AS1_AL161772.1	0.358535338
HAND2-AS1_AP005432.2	0.365274394
HAND2-AS1_AC099066.2	0.304288427
HAND2-AS1_CDKN2B-AS1	0.190877858
HAND2-AS1_AL449423.1	0.961875441
HAND2-AS1_AL445490.1	0.18812719
HAND2-AS1_AC004988.1	0.121263092
HAND2-AS1_LINC02195	0.202485948
HAND2-AS1_AC090125.1	0.830792285
HAND2-AS1_LINC01048	0.98049266
HAND2-AS1_AC090673.1	0.040185241
HAND2-AS1_AL356417.2	0.076214986
HAND2-AS1_AC007750.1	0.743145153
HAND2-AS1_LINC01561	0.769781462
HAND2-AS1_LINC01833	0.022014179
HAND2-AS1_LINC00460	0.466402978
HAND2-AS1_LINC00536	0.203322554
HAND2-AS1_AP000553.1	0.948392628
HAND2-AS1_AATBC	0.846611748
HAND2-AS1_AL161431.1	0.007238402
HAND2-AS1_PICSAR	0.059771715
HAND2-AS1_LINC01615	0.908146026
HAND2-AS1_LINC00641	0.038257017
HAND2-AS1_MIR133A1HG	0.06603793
HAND2-AS1_AC104984.6	0.358164099
HAND2-AS1_AL023755.1	0.077772808
HAND2-AS1_AL359851.1	0.169658833
HAND2-AS1_AC015923.1	0.013872889
HAND2-AS1_AC005828.1	0.062774325
HAND2-AS1_AC023034.1	0.523275432
HAND2-AS1_AC099811.5	0.693845296

HAND2-AS1_FP325330.3	0.495269141
HAND2-AS1_AL355075.4	0.375055633
HAND2-AS1_RMRP	0.6770006
HAND2-AS1_AC037487.2	0.318218661
HAND2-AS1_AC006141.1	0.56315844
HAND2-AS1_LINC02443	0.257211072
HAND2-AS1_MIR181A1HG	0.713429332
HAND2-AS1_LINC02465	0.503618366
HAND2-AS1_AC091906.1	0.215046248
AC005180.2_LINC00702	0.151692895
AC005180.2_MBNL1-AS1	0.245837036
AC005180.2_LINC01614	0.399472648
AC005180.2_MIR100HG	0.542181787
AC005180.2_JAZF1-AS1	0.427776142
AC005180.2_AC022893.1	0.868122232
AC005180.2_ACTA2-AS1	0.318963132
AC005180.2_AP003071.3	0.573922366
AC005180.2_LINC01352	0.13445557
AC005180.2_AC134312.1	0.555469472
AC005180.2_AC091588.3	0.334918539
AC005180.2_HLX-AS1	0.316605247
AC005180.2_LINC00402	0.201040218
AC005180.2_LINC01781	0.725079424
AC005180.2_AC007128.1	0.042618983
AC005180.2_AC099850.3	0.607716621
AC005180.2_AC010655.2	0.240485037
AC005180.2_LINC00941	0.428143029
AC005180.2_MYOSLID	0.523345451
AC005180.2_AL161772.1	0.46039063
AC005180.2_AP005432.2	0.40957717
AC005180.2_AC099066.2	0.760265118
AC005180.2_CDKN2B-AS1	0.538681492
AC005180.2_AL449423.1	0.764338109
AC005180.2_AL445490.1	0.29563791
AC005180.2_U62317.2	0.550461815
AC005180.2_AC004988.1	0.93612458
AC005180.2_LINC02195	0.036704743
AC005180.2_AC090125.1	0.73095196
AC005180.2_LINC01048	0.776312381
AC005180.2_AC090673.1	0.298954351
AC005180.2_AL356417.2	0.56358716
AC005180.2_AC007750.1	0.814413783
AC005180.2_LINC01561	0.769598025
AC005180.2_LINC01833	0.013632022

AC005180.2_LINC00460	0.5803047
AC005180.2_LINC00536	0.021811663
AC005180.2_AP000553.1	0.361461764
AC005180.2_AATBC	0.429225226
AC005180.2_AL161431.1	0.061736877
AC005180.2_PICSAR	0.738306979
AC005180.2_LINC01615	0.668866262
AC005180.2_LINC00641	0.395170197
AC005180.2_MIR133A1HG	0.988065985
AC005180.2_AC104984.6	0.971226576
AC005180.2_AL023755.1	0.699175018
AC005180.2_AL359851.1	0.441837051
AC005180.2_AC015923.1	0.569092408
AC005180.2_AC005828.1	0.66262586
AC005180.2_AC023034.1	0.46867914
AC005180.2_AC099811.5	0.451782504
AC005180.2_FP325330.3	0.312470986
AC005180.2_AL355075.4	0.758613026
AC005180.2_RMRP	0.903388216
AC005180.2_AC037487.2	0.875329289
AC005180.2_AC006141.1	0.860147703
AC005180.2_LINC02443	0.952723436
AC005180.2_MIR181A1HG	0.78382767
AC005180.2_LINC02465	0.571258202
AC005180.2_AC091906.1	0.952661472
LINC00702_MBNL1-AS1	0.161473608
LINC00702_LINC01614	0.030706246
LINC00702_MIR100HG	0.042233747
LINC00702_JAZF1-AS1	0.176402637
LINC00702_AC022893.1	0.251840898
LINC00702_ACTA2-AS1	0.029134392
LINC00702_AP003071.3	0.374110442
LINC00702_LINC01352	0.11801801
LINC00702_AC134312.1	0.667577206
LINC00702_AC091588.3	0.660218988
LINC00702_HLX-AS1	0.489351586
LINC00702_LINC00402	0.173622324
LINC00702_LINC01781	0.107363321
LINC00702_AC007128.1	0.108794561
LINC00702_AC099850.3	0.90191644
LINC00702_AC010655.2	0.271709726
LINC00702_LINC00941	0.13816511
LINC00702_MYOSLID	0.65244428
LINC00702_AL161772.1	0.272115522

LINC00702_AP005432.2	0.258475846
LINC00702_AC099066.2	0.789665034
LINC00702_CDKN2B-AS1	0.015614103
LINC00702_AL449423.1	0.95261199
LINC00702_AL445490.1	0.390708175
LINC00702_U62317.2	0.173352419
LINC00702_AC004988.1	0.023682591
LINC00702_LINC02195	0.021015396
LINC00702_AC090125.1	0.446132176
LINC00702_LINC01048	0.828490483
LINC00702_AC090673.1	0.890325098
LINC00702_AL356417.2	0.715031322
LINC00702_AC007750.1	0.230156414
LINC00702_LINC01561	0.767413144
LINC00702_LINC01833	0.0221509
LINC00702_LINC00460	0.938766445
LINC00702_LINC00536	0.000989842
LINC00702_AP000553.1	0.705659003
LINC00702_AATBC	0.340275507
LINC00702_AL161431.1	0.108803515
LINC00702_PICSAR	0.037625995
LINC00702_LINC01615	0.981128075
LINC00702_LINC00641	0.157495885
LINC00702_MIR133A1HG	0.200635875
LINC00702_AC104984.6	0.414038178
LINC00702_AL023755.1	0.051500989
LINC00702_AL359851.1	0.471241782
LINC00702_AC015923.1	0.009036306
LINC00702_AC005828.1	0.015868944
LINC00702_AC023034.1	0.060412914
LINC00702_AC099811.5	0.448417069
LINC00702_FP325330.3	0.017423791
LINC00702_AL355075.4	0.378628197
LINC00702_RMRP	0.535935223
LINC00702_AC037487.2	0.238524763
LINC00702_AC006141.1	0.436673908
LINC00702_LINC02443	0.098356517
LINC00702_MIR181A1HG	0.574677144
LINC00702_LINC02465	0.789618548
LINC00702_AC091906.1	0.167806463
MBNL1-AS1_LINC01614	0.027312249
MBNL1-AS1_MIR100HG	0.054669714
MBNL1-AS1_AC022893.1	0.142354835
MBNL1-AS1_ACTA2-AS1	0.775396308

MBNL1-AS1_AP003071.3	0.543956397
MBNL1-AS1_LINC01352	0.870238051
MBNL1-AS1_AC134312.1	2.01E-10
MBNL1-AS1_AC091588.3	0.036977829
MBNL1-AS1_HLX-AS1	0.19577148
MBNL1-AS1_LINC00402	0.406126076
MBNL1-AS1_LINC01781	0.417626261
MBNL1-AS1_AC007128.1	0.930879822
MBNL1-AS1_AC099850.3	0.946635786
MBNL1-AS1_AC010655.2	0.027479922
MBNL1-AS1_LINC00941	0.241107192
MBNL1-AS1_MYOSLID	0.111486065
MBNL1-AS1_AL161772.1	0.771573746
MBNL1-AS1_AP005432.2	0.992729977
MBNL1-AS1_AC099066.2	0.055765228
MBNL1-AS1_CDKN2B-AS1	0.561311414
MBNL1-AS1_AL449423.1	0.856213016
MBNL1-AS1_AL445490.1	0.325312145
MBNL1-AS1_U62317.2	0.99260774
MBNL1-AS1_AC004988.1	0.305018092
MBNL1-AS1_LINC02195	0.136689156
MBNL1-AS1_AC090125.1	0.782280286
MBNL1-AS1_LINC01048	0.007419067
MBNL1-AS1_AC090673.1	0.72489615
MBNL1-AS1_AL356417.2	0.24006343
MBNL1-AS1_AC007750.1	0.876780488
MBNL1-AS1_LINC01561	0.172651683
MBNL1-AS1_LINC01833	0.101040507
MBNL1-AS1_LINC00460	0.501146619
MBNL1-AS1_LINC00536	0.000110899
MBNL1-AS1_AP000553.1	0.493336268
MBNL1-AS1_AATBC	0.298437172
MBNL1-AS1_AL161431.1	0.146423836
MBNL1-AS1_PICSAR	0.89493676
MBNL1-AS1_LINC01615	0.11922968
MBNL1-AS1_LINC00641	0.227749856
MBNL1-AS1_MIR133A1HG	0.444611284
MBNL1-AS1_AC104984.6	0.095056889
MBNL1-AS1_AL023755.1	0.966304549
MBNL1-AS1_AC015923.1	0.86876594
MBNL1-AS1_AC005828.1	0.089315182
MBNL1-AS1_AC023034.1	0.292819388
MBNL1-AS1_AC099811.5	2.71E-07
MBNL1-AS1_FP325330.3	0.087110242

MBNL1-AS1_AL355075.4	0.039811151
MBNL1-AS1_RMRP	0.004835946
MBNL1-AS1_AC037487.2	0.630532035
MBNL1-AS1_AC006141.1	0.523395204
MBNL1-AS1_LINC02443	0.051806876
MBNL1-AS1_MIR181A1HG	0.558230318
MBNL1-AS1_LINC02465	0.034406881
MBNL1-AS1_AC091906.1	0.592792648
LINC01614_MIR100HG	0.184353129
LINC01614_JAZF1-AS1	0.025145695
LINC01614_AC022893.1	0.20276688
LINC01614_ACTA2-AS1	0.062736868
LINC01614_AP003071.3	0.164766785
LINC01614_LINC01352	0.02358965
LINC01614_AC134312.1	0.019221239
LINC01614_AC091588.3	0.044904013
LINC01614_HLX-AS1	0.050080922
LINC01614_LINC00402	0.079206593
LINC01614_LINC01781	0.024924815
LINC01614_AC007128.1	0.00061948
LINC01614_AC099850.3	0.152809745
LINC01614_AC010655.2	0.044216487
LINC01614_LINC00941	0.097550099
LINC01614_MYOSLID	0.107717835
LINC01614_AL161772.1	0.05014609
LINC01614_AP005432.2	0.009662917
LINC01614_AC099066.2	0.114453823
LINC01614_CDKN2B-AS1	0.122714355
LINC01614_AL449423.1	0.32331803
LINC01614_AL445490.1	0.173738108
LINC01614_U62317.2	0.027106017
LINC01614_AC004988.1	0.047475461
LINC01614_LINC02195	0.001492674
LINC01614_AC090125.1	0.031221433
LINC01614_LINC01048	0.082749817
LINC01614_AC090673.1	0.079709968
LINC01614_AL356417.2	0.079044794
LINC01614_AC007750.1	0.021726264
LINC01614_LINC01561	0.162290023
LINC01614_LINC01833	0.001719399
LINC01614_LINC00460	0.968712808
LINC01614_LINC00536	0.148176946
LINC01614_AP000553.1	0.032378721
LINC01614_AATBC	0.005833998

LINC01614_AL161431.1	0.931466989
LINC01614_PICSAR	0.097730109
LINC01614_LINC01615	0.075399568
LINC01614_LINC00641	0.030490753
LINC01614_MIR133A1HG	0.030338514
LINC01614_AC104984.6	0.041205692
LINC01614_AL023755.1	0.030073301
LINC01614_AL359851.1	0.022684995
LINC01614_AC015923.1	0.040749106
LINC01614_AC005828.1	0.042294333
LINC01614_AC023034.1	0.065775941
LINC01614_AC099811.5	0.144115082
LINC01614_FP325330.3	0.013371999
LINC01614_AL355075.4	0.060115115
LINC01614_RMRP	0.108285601
LINC01614_AC037487.2	0.246688635
LINC01614_AC006141.1	0.037153687
LINC01614_LINC02443	0.024075342
LINC01614_MIR181A1HG	0.020725712
LINC01614_LINC02465	0.064086454
LINC01614_AC091906.1	0.041557888
MIR100HG_JAZF1-AS1	0.359769049
MIR100HG_AC022893.1	0.186172834
MIR100HG_ACTA2-AS1	0.057546143
MIR100HG_AP003071.3	0.26144913
MIR100HG_LINC01352	0.842366404
MIR100HG_AC134312.1	0.535463636
MIR100HG_AC091588.3	0.430446258
MIR100HG_HLX-AS1	0.627624572
MIR100HG_LINC00402	0.040870121
MIR100HG_LINC01781	0.438240704
MIR100HG_AC007128.1	0.043520405
MIR100HG_AC099850.3	0.377342931
MIR100HG_AC010655.2	0.016862908
MIR100HG_LINC00941	0.042081249
MIR100HG_MYOSLID	0.717398807
MIR100HG_AL161772.1	0.408628983
MIR100HG_AP005432.2	0.129833173
MIR100HG_AC099066.2	0.26930468
MIR100HG_CDKN2B-AS1	0.355571932
MIR100HG_AL449423.1	0.384922723
MIR100HG_AL445490.1	0.042768207
MIR100HG_U62317.2	0.057977887
MIR100HG_AC004988.1	0.583541747

MIR100HG_LINC02195	0.028231867
MIR100HG_AC090125.1	0.92611859
MIR100HG_LINC01048	0.643822291
MIR100HG_AC090673.1	0.970706362
MIR100HG_AL356417.2	0.732155492
MIR100HG_AC007750.1	0.441498489
MIR100HG_LINC01561	0.218390981
MIR100HG_LINC01833	0.077337936
MIR100HG_LINC00460	0.199844124
MIR100HG_LINC00536	9.80E-05
MIR100HG_AP000553.1	0.863468755
MIR100HG_AATBC	8.14E-05
MIR100HG_AL161431.1	0.257780023
MIR100HG_PICSAR	0.500496566
MIR100HG_LINC01615	0.481923202
MIR100HG_LINC00641	0.037769675
MIR100HG_MIR133A1HG	0.643757185
MIR100HG_AC104984.6	0.850657871
MIR100HG_AL023755.1	0.09828958
MIR100HG_AL359851.1	0.735303302
MIR100HG_AC015923.1	0.990863463
MIR100HG_AC005828.1	0.131460748
MIR100HG_AC023034.1	0.578233721
MIR100HG_AC099811.5	0.283693943
MIR100HG_FP325330.3	0.403518326
MIR100HG_AL355075.4	0.075507351
MIR100HG_RMRP	0.737425051
MIR100HG_AC037487.2	0.896071471
MIR100HG_AC006141.1	0.352202693
MIR100HG_LINC02443	0.025500284
MIR100HG_MIR181A1HG	0.444611284
MIR100HG_LINC02465	0.726365382
MIR100HG_AC091906.1	0.171529317
JAZF1-AS1_AC022893.1	0.53936763
JAZF1-AS1_ACTA2-AS1	0.053310816
JAZF1-AS1_AP003071.3	0.350293412
JAZF1-AS1_LINC01352	0.636966059
JAZF1-AS1_AC134312.1	0.691037373
JAZF1-AS1_AC091588.3	0.411892486
JAZF1-AS1_HLX-AS1	0.187674877
JAZF1-AS1_LINC00402	0.33914808
JAZF1-AS1_LINC01781	0.632763112
JAZF1-AS1_AC007128.1	0.910614458
JAZF1-AS1_AC099850.3	0.564661434

JAZF1-AS1_AC010655.2	0.26398664
JAZF1-AS1_LINC00941	0.289915668
JAZF1-AS1_MYOSLID	0.039325675
JAZF1-AS1_AL161772.1	0.080096473
JAZF1-AS1_AP005432.2	6.35E-06
JAZF1-AS1_AC099066.2	0.91240399
JAZF1-AS1_CDKN2B-AS1	0.042861964
JAZF1-AS1_AL449423.1	0.735056946
JAZF1-AS1_AL445490.1	0.200505668
JAZF1-AS1_AC004988.1	0.962230583
JAZF1-AS1_LINC02195	0.683595821
JAZF1-AS1_AC090125.1	0.607670515
JAZF1-AS1_LINC01048	0.545294372
JAZF1-AS1_AC090673.1	0.069568838
JAZF1-AS1_AL356417.2	0.156108267
JAZF1-AS1_AC007750.1	0.398939131
JAZF1-AS1_LINC01561	0.926144386
JAZF1-AS1_LINC01833	0.60827402
JAZF1-AS1_LINC00460	0.182032775
JAZF1-AS1_LINC00536	0.066557865
JAZF1-AS1_AP000553.1	0.933231181
JAZF1-AS1_AATBC	0.155895988
JAZF1-AS1_AL161431.1	0.054103894
JAZF1-AS1_PICSAR	0.104732556
JAZF1-AS1_LINC01615	0.635006334
JAZF1-AS1_LINC00641	0.909384629
JAZF1-AS1_MIR133A1HG	0.551681204
JAZF1-AS1_AC104984.6	0.068228609
JAZF1-AS1_AL023755.1	0.102783747
JAZF1-AS1_AL359851.1	0.211450997
JAZF1-AS1_AC015923.1	0.093574193
JAZF1-AS1_AC005828.1	0.46230625
JAZF1-AS1_AC023034.1	0.963391348
JAZF1-AS1_AC099811.5	0.665559357
JAZF1-AS1_FP325330.3	0.12904826
JAZF1-AS1_AL355075.4	0.018084939
JAZF1-AS1_RMRP	0.968603134
JAZF1-AS1_AC037487.2	0.538541146
JAZF1-AS1_AC006141.1	0.409777172
JAZF1-AS1_LINC02443	0.913061077
JAZF1-AS1_MIR181A1HG	0.676985735
JAZF1-AS1_LINC02465	0.473088473
JAZF1-AS1_AC091906.1	0.733822025
AC022893.1_ACTA2-AS1	0.756896389

AC022893.1_AP003071.3	0.97228266
AC022893.1_LINC01352	0.999485525
AC022893.1_AC134312.1	0.013850835
AC022893.1_AC091588.3	0.474826571
AC022893.1_HLX-AS1	0.126768891
AC022893.1_LINC00402	0.536417328
AC022893.1_LINC01781	0.791442547
AC022893.1_AC007128.1	0.096070834
AC022893.1_AC099850.3	0.967820325
AC022893.1_AC010655.2	0.328908421
AC022893.1_LINC00941	0.714544546
AC022893.1_MYOSLID	0.734781852
AC022893.1_AL161772.1	0.637546701
AC022893.1_AP005432.2	0.114083741
AC022893.1_AC099066.2	0.125780952
AC022893.1_CDKN2B-AS1	0.730569285
AC022893.1_AL449423.1	0.926888877
AC022893.1_AL445490.1	0.606152599
AC022893.1_U62317.2	0.212852055
AC022893.1_AC004988.1	0.4331282
AC022893.1_LINC02195	0.04446845
AC022893.1_AC090125.1	0.392642242
AC022893.1_LINC01048	0.18451878
AC022893.1_AC090673.1	0.633760498
AC022893.1_AL356417.2	0.506698143
AC022893.1_AC007750.1	0.367562732
AC022893.1_LINC01561	0.167414079
AC022893.1_LINC01833	0.023757228
AC022893.1_LINC00460	0.346714725
AC022893.1_LINC00536	0.000189656
AC022893.1_AP000553.1	0.497385989
AC022893.1_AATBC	0.114536432
AC022893.1_AL161431.1	0.062287954
AC022893.1_PICSAR	0.170048514
AC022893.1_LINC01615	0.787538519
AC022893.1_LINC00641	0.154028846
AC022893.1_MIR133A1HG	1.78E-10
AC022893.1_AC104984.6	0.802529311
AC022893.1_AL023755.1	0.979947319
AC022893.1_AC015923.1	0.278902822
AC022893.1_AC005828.1	0.196121151
AC022893.1_AC023034.1	0.57905031
AC022893.1_AC099811.5	0.011788966
AC022893.1_FP325330.3	0.125362473

AC022893.1_AL355075.4	0.848280162
AC022893.1_RMRP	0.542784454
AC022893.1_AC037487.2	0.477230973
AC022893.1_AC006141.1	0.128022603
AC022893.1_LINC02443	0.575539356
AC022893.1_MIR181A1HG	0.681024207
AC022893.1_LINC02465	0.034406881
AC022893.1_AC091906.1	0.30470598
ACTA2-AS1_AP003071.3	0.089581479
ACTA2-AS1_LINC01352	0.571310261
ACTA2-AS1_AC134312.1	0.131618004
ACTA2-AS1_AC091588.3	0.307102536
ACTA2-AS1_HLX-AS1	0.422035188
ACTA2-AS1_LINC00402	0.095482351
ACTA2-AS1_LINC01781	0.604250383
ACTA2-AS1_AC007128.1	0.160330064
ACTA2-AS1_AC099850.3	0.46219855
ACTA2-AS1_AC010655.2	0.18410379
ACTA2-AS1_LINC00941	0.982088129
ACTA2-AS1_MYOSLID	0.564306959
ACTA2-AS1_AL161772.1	0.711036983
ACTA2-AS1_AP005432.2	0.166507166
ACTA2-AS1_AC099066.2	0.05389411
ACTA2-AS1_CDKN2B-AS1	0.799923484
ACTA2-AS1_AL449423.1	0.939109198
ACTA2-AS1_AL445490.1	0.774024374
ACTA2-AS1_U62317.2	0.76301116
ACTA2-AS1_AC004988.1	0.795036597
ACTA2-AS1_LINC02195	0.124207253
ACTA2-AS1_AC090125.1	0.718935493
ACTA2-AS1_LINC01048	0.198199507
ACTA2-AS1_AC090673.1	0.56445877
ACTA2-AS1_AL356417.2	0.958804366
ACTA2-AS1_AC007750.1	0.683658834
ACTA2-AS1_LINC01561	0.65215076
ACTA2-AS1_LINC01833	0.212573799
ACTA2-AS1_LINC00460	0.237700845
ACTA2-AS1_LINC00536	1.89E-06
ACTA2-AS1_AP000553.1	0.300921855
ACTA2-AS1_AATBC	0.13231229
ACTA2-AS1_AL161431.1	0.063034912
ACTA2-AS1_PICSAR	0.537282359
ACTA2-AS1_LINC01615	0.272162406
ACTA2-AS1_LINC00641	0.941613088

ACTA2-AS1_MIR133A1HG	0.197629894
ACTA2-AS1_AC104984.6	0.928173052
ACTA2-AS1_AL023755.1	0.219541199
ACTA2-AS1_AL359851.1	0.188928349
ACTA2-AS1_AC015923.1	0.616315353
ACTA2-AS1_AC005828.1	0.262118937
ACTA2-AS1_AC023034.1	0.458044966
ACTA2-AS1_AC099811.5	0.769687087
ACTA2-AS1_FP325330.3	0.658657539
ACTA2-AS1_AL355075.4	0.225990898
ACTA2-AS1_RMRP	0.190753318
ACTA2-AS1_AC037487.2	0.503279045
ACTA2-AS1_AC006141.1	0.570771548
ACTA2-AS1_LINC02443	0.359519188
ACTA2-AS1_MIR181A1HG	0.202363786
ACTA2-AS1_LINC02465	0.504882176
ACTA2-AS1_AC091906.1	0.185900572
AP003071.3_LINC01352	0.755669156
AP003071.3_AC134312.1	0.572876172
AP003071.3_AC091588.3	0.182274502
AP003071.3_HLX-AS1	0.378115788
AP003071.3_LINC00402	0.103258537
AP003071.3_LINC01781	0.273841347
AP003071.3_AC007128.1	0.396391716
AP003071.3_AC099850.3	0.354835519
AP003071.3_AC010655.2	0.668694274
AP003071.3_LINC00941	0.852460633
AP003071.3_MYOSLID	0.565077415
AP003071.3_AL161772.1	0.861897166
AP003071.3_AP005432.2	0.067261312
AP003071.3_AC099066.2	0.269496639
AP003071.3_CDKN2B-AS1	0.047750805
AP003071.3_AL449423.1	0.990504267
AP003071.3_AL445490.1	0.533541873
AP003071.3_AC004988.1	0.010649267
AP003071.3_LINC02195	0.068578991
AP003071.3_AC090125.1	0.370167417
AP003071.3_LINC01048	0.662416745
AP003071.3_AC090673.1	0.179019593
AP003071.3_AL356417.2	0.271383359
AP003071.3_AC007750.1	0.156468477
AP003071.3_LINC01561	0.242607405
AP003071.3_LINC01833	0.22374517
AP003071.3_LINC00460	0.767068541

AP003071.3_LINC00536	0.63295507
AP003071.3_AP000553.1	0.418637305
AP003071.3_AATBC	0.109717467
AP003071.3_AL161431.1	0.121960275
AP003071.3_PICSAR	0.338145174
AP003071.3_LINC01615	0.201939179
AP003071.3_LINC00641	0.791104835
AP003071.3_MIR133A1HG	0.349374233
AP003071.3_AC104984.6	0.304469276
AP003071.3_AL023755.1	0.165117943
AP003071.3_AL359851.1	0.409159701
AP003071.3_AC015923.1	0.130490471
AP003071.3_AC005828.1	0.39072377
AP003071.3_AC023034.1	0.637296917
AP003071.3_AC099811.5	0.529746862
AP003071.3_FP325330.3	0.102767369
AP003071.3_AL355075.4	0.805375095
AP003071.3_RMRP	0.831097726
AP003071.3_AC037487.2	0.058121772
AP003071.3_AC006141.1	0.416236128
AP003071.3_LINC02443	0.150117342
AP003071.3_MIR181A1HG	0.225227959
AP003071.3_LINC02465	0.331774383
AP003071.3_AC091906.1	0.184752832
LINC01352_AC134312.1	0.83964118
LINC01352_AC091588.3	0.067317149
LINC01352_HLX-AS1	0.765314614
LINC01352_LINC00402	0.995053231
LINC01352_LINC01781	0.374470866
LINC01352_AC007128.1	0.739537008
LINC01352_AC099850.3	3.32E-06
LINC01352_AC010655.2	0.076770489
LINC01352_LINC00941	0.463684828
LINC01352_MYOSLID	0.257367633
LINC01352_AL161772.1	0.610944311
LINC01352_AP005432.2	0.298066791
LINC01352_AC099066.2	0.889205878
LINC01352_CDKN2B-AS1	0.487616067
LINC01352_AL449423.1	0.673149936
LINC01352_AL445490.1	0.28996232
LINC01352_AC004988.1	0.339842541
LINC01352_LINC02195	0.681980734
LINC01352_AC090125.1	0.798763084
LINC01352_LINC01048	0.542241327

LINC01352_AC090673.1	0.555553454
LINC01352_AL356417.2	0.06062399
LINC01352_AC007750.1	0.244621113
LINC01352_LINC01561	0.70391657
LINC01352_LINC01833	0.125853204
LINC01352_LINC00460	0.31551752
LINC01352_LINC00536	0.560925456
LINC01352_AP000553.1	0.875056957
LINC01352_AATBC	0.406251296
LINC01352_AL161431.1	0.097014229
LINC01352_PICSAR	0.700417604
LINC01352_LINC01615	0.124939691
LINC01352_LINC00641	0.042667572
LINC01352_MIR133A1HG	0.997678796
LINC01352_AC104984.6	0.03269871
LINC01352_AL023755.1	0.055173019
LINC01352_AL359851.1	0.721414868
LINC01352_AC015923.1	0.081813616
LINC01352_AC005828.1	0.214480236
LINC01352_AC023034.1	0.302521766
LINC01352_AC099811.5	0.322940228
LINC01352_FP325330.3	0.111728256
LINC01352_AL355075.4	0.176293056
LINC01352_RMRP	0.738933727
LINC01352_AC037487.2	0.050703008
LINC01352_AC006141.1	0.411431902
LINC01352_LINC02443	0.121484746
LINC01352_MIR181A1HG	0.819585071
LINC01352_LINC02465	0.782758227
LINC01352_AC091906.1	0.474790979
AC134312.1_AC091588.3	0.497460697
AC134312.1_HLX-AS1	0.242918793
AC134312.1_LINC00402	0.400655408
AC134312.1_LINC01781	0.888488983
AC134312.1_AC007128.1	0.646710138
AC134312.1_AC099850.3	3.32E-06
AC134312.1_AC010655.2	0.290583893
AC134312.1_LINC00941	0.62343254
AC134312.1_MYOSLID	0.315748012
AC134312.1_AL161772.1	0.07570811
AC134312.1_AP005432.2	0.009400911
AC134312.1_AC099066.2	0.159901149
AC134312.1_CDKN2B-AS1	0.568915159
AC134312.1_AL449423.1	0.848061395

AC134312.1_AL445490.1	0.827449671
AC134312.1_AC004988.1	0.875028241
AC134312.1_LINC02195	0.555946756
AC134312.1_AC090125.1	0.924046883
AC134312.1_LINC01048	0.713566643
AC134312.1_AC090673.1	0.163564415
AC134312.1_AL356417.2	0.069831889
AC134312.1_AC007750.1	0.967251345
AC134312.1_LINC01561	0.34790251
AC134312.1_LINC01833	0.534550702
AC134312.1_LINC00460	0.971503595
AC134312.1_LINC00536	0.727007659
AC134312.1_AP000553.1	0.81098604
AC134312.1_AATBC	0.925982393
AC134312.1_AL161431.1	0.065463242
AC134312.1_PICSAR	0.988626589
AC134312.1_LINC01615	0.470735563
AC134312.1_MIR133A1HG	0.498784279
AC134312.1_AC104984.6	0.261336382
AC134312.1_AL023755.1	0.062446655
AC134312.1_AL359851.1	0.709534738
AC134312.1_AC015923.1	0.189921111
AC134312.1_AC005828.1	0.450131599
AC134312.1_AC023034.1	0.446403675
AC134312.1_AC099811.5	0.049232159
AC134312.1_FP325330.3	0.955458891
AC134312.1_AL355075.4	0.145170313
AC134312.1_RMRP	0.540610057
AC134312.1_AC037487.2	0.140161145
AC134312.1_AC006141.1	0.740379257
AC134312.1_LINC02443	0.738497661
AC134312.1_MIR181A1HG	0.305590314
AC134312.1_LINC02465	0.938475336
AC134312.1_AC091906.1	0.831582629
AC091588.3_HLX-AS1	0.814409205
AC091588.3_LINC00402	0.629954858
AC091588.3_LINC01781	0.933801099
AC091588.3_AC007128.1	0.706086274
AC091588.3_AC099850.3	3.32E-06
AC091588.3_AC010655.2	0.932146635
AC091588.3_LINC00941	0.715366531
AC091588.3_MYOSLID	0.619841598
AC091588.3_AL161772.1	0.355085056
AC091588.3_AP005432.2	0.025829445

AC091588.3_AC099066.2	0.596239818
AC091588.3_CDKN2B-AS1	0.122468571
AC091588.3_AL449423.1	0.876611412
AC091588.3_AL445490.1	0.529444266
AC091588.3_AC004988.1	0.356532257
AC091588.3_LINC02195	0.475809514
AC091588.3_AC090125.1	0.562341329
AC091588.3_LINC01048	0.368939407
AC091588.3_AC090673.1	0.634651698
AC091588.3_AL356417.2	0.272043529
AC091588.3_AC007750.1	0.601750439
AC091588.3_LINC01561	0.924034394
AC091588.3_LINC01833	0.134300317
AC091588.3_LINC00460	0.886106116
AC091588.3_LINC00536	0.48794317
AC091588.3_AP000553.1	0.97646423
AC091588.3_AATBC	0.8883032
AC091588.3_AL161431.1	0.145872202
AC091588.3_PICSAR	0.509612388
AC091588.3_LINC01615	0.503271856
AC091588.3_MIR133A1HG	0.862778916
AC091588.3_AC104984.6	0.281642427
AC091588.3_AL023755.1	0.639605353
AC091588.3_AL359851.1	0.42395493
AC091588.3_AC015923.1	0.445449388
AC091588.3_AC005828.1	0.801653503
AC091588.3_AC023034.1	0.700318214
AC091588.3_AC099811.5	0.988645203
AC091588.3_FP325330.3	0.222147371
AC091588.3_AL355075.4	0.572970634
AC091588.3_RMRP	0.625579741
AC091588.3_AC037487.2	0.581762761
AC091588.3_AC006141.1	0.554256457
AC091588.3_LINC02443	0.26054039
AC091588.3_MIR181A1HG	0.957543307
AC091588.3_LINC02465	0.646227791
AC091588.3_AC091906.1	0.883208957
HLX-AS1_LINC00402	0.036263473
HLX-AS1_LINC01781	0.793985213
HLX-AS1_AC007128.1	0.720366957
HLX-AS1_AC099850.3	0.636228414
HLX-AS1_AC010655.2	0.104543126
HLX-AS1_LINC00941	0.456280862
HLX-AS1_MYOSLID	0.880591952

HLX-AS1_AL161772.1	0.263161721
HLX-AS1_AP005432.2	0.052453206
HLX-AS1_AC099066.2	0.243144163
HLX-AS1_CDKN2B-AS1	0.659642603
HLX-AS1_AL449423.1	0.216350855
HLX-AS1_AL445490.1	0.101247652
HLX-AS1_AC004988.1	0.737996417
HLX-AS1_LINC02195	0.360386174
HLX-AS1_AC090125.1	0.928405132
HLX-AS1_LINC01048	0.84744214
HLX-AS1_AC090673.1	0.402472307
HLX-AS1_AL356417.2	0.738609546
HLX-AS1_AC007750.1	0.69299013
HLX-AS1_LINC01561	0.814968809
HLX-AS1_LINC01833	0.228734616
HLX-AS1_LINC00460	0.678940383
HLX-AS1_LINC00536	0.127263549
HLX-AS1_AP000553.1	0.1754192
HLX-AS1_AATBC	0.761042495
HLX-AS1_AL161431.1	0.972974258
HLX-AS1_PICSAR	0.900780288
HLX-AS1_LINC01615	0.281474477
HLX-AS1_LINC00641	0.583434919
HLX-AS1_MIR133A1HG	0.126262136
HLX-AS1_AC104984.6	0.065452557
HLX-AS1_AL023755.1	0.04261382
HLX-AS1_AL359851.1	0.206467651
HLX-AS1_AC015923.1	0.023849399
HLX-AS1_AC005828.1	0.308163671
HLX-AS1_AC023034.1	0.024063812
HLX-AS1_AC099811.5	0.261321383
HLX-AS1_FP325330.3	0.038580479
HLX-AS1_AL355075.4	0.341513956
HLX-AS1_RMRP	0.092063
HLX-AS1_AC037487.2	0.094563902
HLX-AS1_AC006141.1	0.147006422
HLX-AS1_LINC02443	0.06137243
HLX-AS1_MIR181A1HG	0.23801745
HLX-AS1_LINC02465	0.059179593
HLX-AS1_AC091906.1	0.39863524
LINC00402_LINC01781	0.637757166
LINC00402_AC007128.1	0.611136026
LINC00402_AC099850.3	0.42748968
LINC00402_AC010655.2	0.631205347

LINC00402_LINC00941	0.613374355
LINC00402_MYOSLID	0.913709025
LINC00402_AL161772.1	0.940691353
LINC00402_AP005432.2	0.476461679
LINC00402_AC099066.2	0.566962858
LINC00402_CDKN2B-AS1	0.482446592
LINC00402_AL449423.1	0.622963062
LINC00402_AL445490.1	0.844211706
LINC00402_AC004988.1	0.400453467
LINC00402_LINC02195	0.660926795
LINC00402_AC090125.1	0.46805374
LINC00402_LINC01048	0.621836576
LINC00402_AC090673.1	0.463338561
LINC00402_AL356417.2	0.279124153
LINC00402_AC007750.1	0.432633295
LINC00402_LINC01561	0.059921384
LINC00402_LINC01833	0.232659502
LINC00402_LINC00460	0.027667755
LINC00402_LINC00536	0.107878696
LINC00402_AP000553.1	0.855507267
LINC00402_AATBC	0.964976546
LINC00402_AL161431.1	0.204205703
LINC00402_PICSAR	0.377682648
LINC00402_LINC01615	0.954576055
LINC00402_MIR133A1HG	0.155996417
LINC00402_AC104984.6	0.08710378
LINC00402_AL023755.1	0.383936863
LINC00402_AL359851.1	0.247361358
LINC00402_AC015923.1	0.443585987
LINC00402_AC005828.1	0.765897945
LINC00402_AC023034.1	0.541985347
LINC00402_AC099811.5	0.10997407
LINC00402_FP325330.3	0.995962819
LINC00402_AL355075.4	0.845828887
LINC00402_RMRP	0.764912112
LINC00402_AC037487.2	0.581662857
LINC00402_AC006141.1	0.243281296
LINC00402_LINC02443	0.284018019
LINC00402_MIR181A1HG	0.392919319
LINC00402_LINC02465	0.375186262
LINC00402_AC091906.1	0.366905974
LINC01781_AC007128.1	0.728471447
LINC01781_AC099850.3	0.069794187
LINC01781_AC010655.2	0.847493056

LINC01781_LINC00941	0.82009739
LINC01781_MYOSLID	0.910828914
LINC01781_AL161772.1	0.558940393
LINC01781_AP005432.2	0.548631612
LINC01781_AC099066.2	0.412748379
LINC01781_CDKN2B-AS1	0.61444716
LINC01781_AL449423.1	0.606377447
LINC01781_AL445490.1	0.634593072
LINC01781_U62317.2	0.760392192
LINC01781_AC004988.1	0.813448811
LINC01781_LINC02195	0.274758019
LINC01781_AC090125.1	0.491636004
LINC01781_LINC01048	0.681936948
LINC01781_AC090673.1	0.966530762
LINC01781_AL356417.2	0.572792661
LINC01781_AC007750.1	0.928600774
LINC01781_LINC01561	0.819054629
LINC01781_LINC01833	0.338400518
LINC01781_LINC00460	0.617196601
LINC01781_LINC00536	0.979751261
LINC01781_AP000553.1	0.862044783
LINC01781_AATBC	0.48120147
LINC01781_AL161431.1	0.554427694
LINC01781_PICSAR	0.46924208
LINC01781_LINC01615	0.261769206
LINC01781_LINC00641	0.555965325
LINC01781_MIR133A1HG	0.708539877
LINC01781_AC104984.6	0.411747579
LINC01781_AL023755.1	0.739912397
LINC01781_AL359851.1	0.752775739
LINC01781_AC015923.1	0.382539603
LINC01781_AC005828.1	0.665802343
LINC01781_AC023034.1	0.6758654
LINC01781_AC099811.5	0.818295055
LINC01781_FP325330.3	0.525319069
LINC01781_AL355075.4	0.776659604
LINC01781_RMRP	0.773544096
LINC01781_AC037487.2	0.417976987
LINC01781_AC006141.1	0.785782903
LINC01781_LINC02443	0.96429617
LINC01781_MIR181A1HG	0.632323139
LINC01781_LINC02465	0.614992226
LINC01781_AC091906.1	0.613846043
AC007128.1_AC099850.3	0.241558123

AC007128.1_AC010655.2	0.6152564
AC007128.1_LINC00941	0.8941342
AC007128.1_MYOSLID	0.284521788
AC007128.1_AL161772.1	0.663005157
AC007128.1_AP005432.2	0.344914375
AC007128.1_AC099066.2	0.143224979
AC007128.1_CDKN2B-AS1	0.510722426
AC007128.1_AL449423.1	0.414352649
AC007128.1_AL445490.1	0.982865085
AC007128.1_U62317.2	0.346949337
AC007128.1_AC004988.1	0.562149075
AC007128.1_LINC02195	0.343023189
AC007128.1_AC090125.1	0.266304367
AC007128.1_LINC01048	0.761902671
AC007128.1_AC090673.1	0.549692076
AC007128.1_AL356417.2	0.379842948
AC007128.1_AC007750.1	0.672139004
AC007128.1_LINC01561	0.46423615
AC007128.1_LINC01833	0.137248592
AC007128.1_LINC00460	0.912191997
AC007128.1_LINC00536	0.199300916
AC007128.1_AP000553.1	0.859691738
AC007128.1_AATBC	0.001114672
AC007128.1_AL161431.1	0.198055439
AC007128.1_PICSAR	0.292281534
AC007128.1_LINC01615	0.256128869
AC007128.1_LINC00641	0.459025379
AC007128.1_MIR133A1HG	0.602974655
AC007128.1_AC104984.6	0.58175512
AC007128.1_AL023755.1	0.905571425
AC007128.1_AL359851.1	0.618649673
AC007128.1_AC015923.1	0.770021644
AC007128.1_AC005828.1	0.657005984
AC007128.1_AC023034.1	0.798636629
AC007128.1_AC099811.5	0.734047586
AC007128.1_FP325330.3	0.894211658
AC007128.1_AL355075.4	0.395297428
AC007128.1_RMRP	0.400213608
AC007128.1_AC037487.2	0.843270858
AC007128.1_AC006141.1	0.835070283
AC007128.1_LINC02443	0.55277119
AC007128.1_MIR181A1HG	0.640537825
AC007128.1_LINC02465	0.875408276
AC007128.1_AC091906.1	0.776440798

AC099850.3_AC010655.2	0.264096618
AC099850.3_LINC00941	0.861837191
AC099850.3_MYOSLID	0.086894209
AC099850.3_AL161772.1	0.646287161
AC099850.3_AP005432.2	0.754363671
AC099850.3_AC099066.2	0.103719234
AC099850.3_CDKN2B-AS1	0.296232934
AC099850.3_AL449423.1	0.455563488
AC099850.3_AL445490.1	0.429749883
AC099850.3_U62317.2	0.002548052
AC099850.3_AC004988.1	0.455563488
AC099850.3_LINC02195	0.172382593
AC099850.3_AC090125.1	0.723615386
AC099850.3_LINC01048	0.610725824
AC099850.3_AC090673.1	0.455563488
AC099850.3_AL356417.2	0.93273751
AC099850.3_AC007750.1	0.455563488
AC099850.3_LINC01561	0.899576327
AC099850.3_LINC01833	0.005839331
AC099850.3_LINC00460	0.433727296
AC099850.3_LINC00536	0.455563488
AC099850.3_AP000553.1	0.354835519
AC099850.3_AATBC	0.006511008
AC099850.3_AL161431.1	0.446444178
AC099850.3_PICSAR	0.76218127
AC099850.3_LINC01615	0.343709349
AC099850.3_LINC00641	0.143644965
AC099850.3_MIR133A1HG	0.455563488
AC099850.3_AC104984.6	0.455563488
AC099850.3_AL023755.1	0.455563488
AC099850.3_AL359851.1	0.455563488
AC099850.3_AC015923.1	0.455563488
AC099850.3_AC005828.1	0.455563488
AC099850.3_AC023034.1	0.455563488
AC099850.3_AC099811.5	0.455563488
AC099850.3_FP325330.3	0.455563488
AC099850.3_AL355075.4	0.511977047
AC099850.3_RMRP	0.511977047
AC099850.3_AC037487.2	0.455563488
AC099850.3_AC006141.1	0.455563488
AC099850.3_LINC02443	0.455563488
AC099850.3_MIR181A1HG	0.455563488
AC099850.3_LINC02465	0.455563488
AC099850.3_AC091906.1	0.455563488

AC010655.2_LINC00941	0.876456967
AC010655.2_MYOSLID	0.336042802
AC010655.2_AL161772.1	0.645397945
AC010655.2_AP005432.2	0.900091955
AC010655.2_AC099066.2	0.137722381
AC010655.2_CDKN2B-AS1	0.435197827
AC010655.2_AL449423.1	0.256237181
AC010655.2_AL445490.1	0.78872417
AC010655.2_U62317.2	0.384704215
AC010655.2_AC004988.1	0.097187396
AC010655.2_LINC02195	0.405180637
AC010655.2_AC090125.1	0.860090131
AC010655.2_LINC01048	0.467759912
AC010655.2_AC090673.1	0.983494475
AC010655.2_AL356417.2	0.605144817
AC010655.2_AC007750.1	0.776483009
AC010655.2_LINC01561	0.749115967
AC010655.2_LINC01833	0.106568839
AC010655.2_LINC00460	0.545241909
AC010655.2_LINC00536	0.000873924
AC010655.2_AP000553.1	0.521865328
AC010655.2_AATBC	0.012556517
AC010655.2_AL161431.1	0.193563117
AC010655.2_PICSAR	0.529807977
AC010655.2_LINC01615	0.172488345
AC010655.2_LINC00641	0.128047291
AC010655.2_MIR133A1HG	0.798608939
AC010655.2_AC104984.6	0.469023568
AC010655.2_AL023755.1	0.242298702
AC010655.2_AL359851.1	0.555012315
AC010655.2_AC015923.1	0.732713375
AC010655.2_AC005828.1	0.451011743
AC010655.2_AC023034.1	0.103897552
AC010655.2_AC099811.5	0.520833789
AC010655.2_FP325330.3	0.899713401
AC010655.2_AL355075.4	0.665099999
AC010655.2_RMRP	0.754887794
AC010655.2_AC037487.2	0.930690763
AC010655.2_AC006141.1	0.555012315
AC010655.2_LINC02443	0.9447377
AC010655.2_MIR181A1HG	0.638387591
AC010655.2_LINC02465	0.772853797
AC010655.2_AC091906.1	0.777315538
LINC00941_MYOSLID	0.185970816

LINC00941_AL161772.1	0.512685635
LINC00941_AP005432.2	0.477693023
LINC00941_AC099066.2	0.264292025
LINC00941_CDKN2B-AS1	0.35917328
LINC00941_AL449423.1	0.100115114
LINC00941_AL445490.1	0.756844089
LINC00941_U62317.2	0.056918481
LINC00941_AC004988.1	0.778903681
LINC00941_LINC02195	0.014729505
LINC00941_AC090125.1	0.16553518
LINC00941_LINC01048	0.138955452
LINC00941_AC090673.1	0.959231601
LINC00941_AL356417.2	0.163900897
LINC00941_AC007750.1	0.379262175
LINC00941_LINC01561	0.497549133
LINC00941_LINC01833	0.149467515
LINC00941_LINC00460	0.349511936
LINC00941_LINC00536	0.00913369
LINC00941_AP000553.1	0.43449805
LINC00941_AATBC	0.114051486
LINC00941_AL161431.1	0.398613706
LINC00941_PICSAR	0.593182399
LINC00941_LINC01615	0.890251971
LINC00941_LINC00641	0.618894962
LINC00941_MIR133A1HG	0.658312693
LINC00941_AC104984.6	0.696780063
LINC00941_AL023755.1	0.802983678
LINC00941_AL359851.1	0.373561707
LINC00941_AC015923.1	0.716947354
LINC00941_AC005828.1	0.78370196
LINC00941_AC023034.1	0.614630756
LINC00941_AC099811.5	0.907368538
LINC00941_FP325330.3	0.654632197
LINC00941_AL355075.4	0.972239964
LINC00941_RMRP	0.487991524
LINC00941_AC037487.2	0.74375387
LINC00941_AC006141.1	0.666215255
LINC00941_LINC02443	0.454623158
LINC00941_MIR181A1HG	0.507700203
LINC00941_LINC02465	0.551572549
LINC00941_AC091906.1	0.343012979
MYOSLID_AL161772.1	0.740702356
MYOSLID_AP005432.2	0.219768304
MYOSLID_AC099066.2	0.824027086

MYOSLID_CDKN2B-AS1	0.463084134
MYOSLID_AL449423.1	0.144335064
MYOSLID_AL445490.1	0.579727614
MYOSLID_U62317.2	0.009955656
MYOSLID_AC004988.1	0.238437556
MYOSLID_LINC02195	0.079130686
MYOSLID_AC090125.1	0.281071822
MYOSLID_LINC01048	0.971717435
MYOSLID_AC090673.1	0.596571139
MYOSLID_AL356417.2	0.394694897
MYOSLID_AC007750.1	0.825592385
MYOSLID_LINC01561	0.8562212
MYOSLID_LINC01833	0.142272042
MYOSLID_LINC00460	0.557143336
MYOSLID_LINC00536	0.111703724
MYOSLID_AP000553.1	0.370371255
MYOSLID_AATBC	0.168030218
MYOSLID_AL161431.1	0.58403669
MYOSLID_PICSAR	0.230730215
MYOSLID_LINC01615	0.61036045
MYOSLID_LINC00641	0.076040372
MYOSLID_MIR133A1HG	0.808854094
MYOSLID_AC104984.6	0.144449105
MYOSLID_AL023755.1	0.267030785
MYOSLID_AL359851.1	0.954947242
MYOSLID_AC015923.1	0.276885095
MYOSLID_AC005828.1	0.964380478
MYOSLID_AC023034.1	0.846425614
MYOSLID_AC099811.5	0.549416766
MYOSLID_FP325330.3	0.252864945
MYOSLID_AL355075.4	0.504296599
MYOSLID_RMRP	0.077325511
MYOSLID_AC037487.2	0.604806274
MYOSLID_AC006141.1	0.793442058
MYOSLID_LINC02443	0.312527649
MYOSLID_MIR181A1HG	0.49293252
MYOSLID_LINC02465	0.052940658
MYOSLID_AC091906.1	0.202096105
AL161772.1_AP005432.2	0.002733185
AL161772.1_AC099066.2	0.860347239
AL161772.1_CDKN2B-AS1	0.644041895
AL161772.1_AL449423.1	0.879220915
AL161772.1_AL445490.1	0.667294459
AL161772.1_U62317.2	0.413703015

AL161772.1_AC004988.1	0.180915625
AL161772.1_LINC02195	0.034873336
AL161772.1_AC090125.1	0.38742399
AL161772.1_LINC01048	0.076852302
AL161772.1_AC090673.1	0.190089029
AL161772.1_AL356417.2	0.692037697
AL161772.1_AC007750.1	0.239935961
AL161772.1_LINC01561	0.272984151
AL161772.1_LINC01833	0.000857357
AL161772.1_LINC00460	0.846811414
AL161772.1_LINC00536	0.006048864
AL161772.1_AP000553.1	0.306995584
AL161772.1_AATBC	0.003274457
AL161772.1_AL161431.1	0.346904323
AL161772.1_PICSAR	0.907819328
AL161772.1_LINC01615	0.062211424
AL161772.1_LINC00641	0.574645646
AL161772.1_MIR133A1HG	0.84422816
AL161772.1_AC104984.6	0.334311366
AL161772.1_AL023755.1	0.998819698
AL161772.1_AL359851.1	0.77970828
AL161772.1_AC015923.1	0.624775827
AL161772.1_AC005828.1	0.620880683
AL161772.1_AC023034.1	0.744638757
AL161772.1_AC099811.5	0.055956902
AL161772.1_FP325330.3	0.925314112
AL161772.1_AL355075.4	0.294256911
AL161772.1_RMRP	0.30091749
AL161772.1_AC037487.2	0.357008505
AL161772.1_AC006141.1	0.949421938
AL161772.1_LINC02443	0.655732388
AL161772.1_MIR181A1HG	0.649212198
AL161772.1_LINC02465	0.829531824
AL161772.1_AC091906.1	0.892293689
AP005432.2_AC099066.2	0.311126066
AP005432.2_CDKN2B-AS1	0.162231343
AP005432.2_AL449423.1	0.1695025
AP005432.2_AL445490.1	0.96694861
AP005432.2_U62317.2	0.675619986
AP005432.2_AC004988.1	0.708500634
AP005432.2_LINC02195	0.943498956
AP005432.2_AC090125.1	0.085710626
AP005432.2_LINC01048	0.005443796
AP005432.2_AC090673.1	0.303267226

AP005432.2_AL356417.2	0.241258856
AP005432.2_AC007750.1	0.562886206
AP005432.2_LINC01561	0.888442567
AP005432.2_LINC01833	0.257418083
AP005432.2_LINC00460	0.203098487
AP005432.2_LINC00536	0.001431491
AP005432.2_AP000553.1	0.010123986
AP005432.2_AATBC	0.039512697
AP005432.2_AL161431.1	0.272711625
AP005432.2_PICSAR	0.485148649
AP005432.2_LINC01615	0.056260674
AP005432.2_LINC00641	0.393483835
AP005432.2_MIR133A1HG	0.027968837
AP005432.2_AC104984.6	0.02583432
AP005432.2_AL023755.1	0.153769684
AP005432.2_AL359851.1	0.001915391
AP005432.2_AC015923.1	0.00329905
AP005432.2_AC005828.1	0.002428124
AP005432.2_AC023034.1	0.052271005
AP005432.2_AC099811.5	0.002651291
AP005432.2_FP325330.3	0.019855169
AP005432.2_AL355075.4	0.000705024
AP005432.2_RMRP	0.003883034
AP005432.2_AC037487.2	0.011820832
AP005432.2_AC006141.1	0.006524075
AP005432.2_LINC02443	0.026901505
AP005432.2_MIR181A1HG	0.002024467
AP005432.2_LINC02465	0.003645765
AP005432.2_AC091906.1	0.004575902
AC099066.2_CDKN2B-AS1	0.009972378
AC099066.2_AL449423.1	0.802699311
AC099066.2_AL445490.1	0.165187125
AC099066.2_AC004988.1	0.205566039
AC099066.2_LINC02195	0.010143348
AC099066.2_AC090125.1	0.825308228
AC099066.2_LINC01048	0.935726263
AC099066.2_AC090673.1	0.581251621
AC099066.2_AL356417.2	0.702520526
AC099066.2_AC007750.1	0.713076375
AC099066.2_LINC01561	0.870035162
AC099066.2_LINC01833	0.173761263
AC099066.2_LINC00460	0.416306637
AC099066.2_LINC00536	0.006790674
AC099066.2_AP000553.1	0.692578707

AC099066.2_AATBC	0.153608004
AC099066.2_AL161431.1	0.172557095
AC099066.2_PICSAR	0.131744027
AC099066.2_LINC01615	0.623549159
AC099066.2_LINC00641	0.912828969
AC099066.2_MIR133A1HG	0.588862963
AC099066.2_AC104984.6	0.366830232
AC099066.2_AL023755.1	0.565294158
AC099066.2_AL359851.1	0.590864449
AC099066.2_AC015923.1	0.069291852
AC099066.2_AC005828.1	0.395873266
AC099066.2_AC023034.1	0.880200474
AC099066.2_AC099811.5	0.377664522
AC099066.2_FP325330.3	0.564977489
AC099066.2_AL355075.4	0.686480646
AC099066.2_RMRP	0.219393991
AC099066.2_AC037487.2	0.954485945
AC099066.2_AC006141.1	0.899507792
AC099066.2_LINC02443	0.513508324
AC099066.2_MIR181A1HG	0.957247407
AC099066.2_LINC02465	0.356338688
AC099066.2_AC091906.1	0.396254203
CDKN2B-AS1_AL449423.1	0.223158872
CDKN2B-AS1_AL445490.1	0.727028024
CDKN2B-AS1_AC004988.1	0.643383717
CDKN2B-AS1_LINC02195	0.026490152
CDKN2B-AS1_AC090125.1	0.334757156
CDKN2B-AS1_LINC01048	0.086257283
CDKN2B-AS1_AC090673.1	0.022369602
CDKN2B-AS1_AL356417.2	0.541941545
CDKN2B-AS1_AC007750.1	0.031180118
CDKN2B-AS1_LINC01561	0.228586963
CDKN2B-AS1_LINC01833	0.059131547
CDKN2B-AS1_LINC00460	0.331487051
CDKN2B-AS1_LINC00536	0.004917615
CDKN2B-AS1_AP000553.1	0.827015472
CDKN2B-AS1_AATBC	0.03559591
CDKN2B-AS1_AL161431.1	0.006254577
CDKN2B-AS1_PICSAR	0.694752995
CDKN2B-AS1_LINC01615	0.784987642
CDKN2B-AS1_LINC00641	0.96923037
CDKN2B-AS1_MIR133A1HG	0.607680403
CDKN2B-AS1_AC104984.6	0.203349806
CDKN2B-AS1_AL023755.1	0.301081178

CDKN2B-AS1_AL359851.1	0.922894197
CDKN2B-AS1_AC015923.1	0.485282196
CDKN2B-AS1_AC005828.1	0.682764674
CDKN2B-AS1_AC023034.1	0.328134786
CDKN2B-AS1_AC099811.5	0.9469466
CDKN2B-AS1_FP325330.3	0.665880224
CDKN2B-AS1_AL355075.4	0.24109975
CDKN2B-AS1_RMRP	0.006849284
CDKN2B-AS1_AC037487.2	0.658979439
CDKN2B-AS1_AC006141.1	0.054205492
CDKN2B-AS1_LINC02443	0.399937669
CDKN2B-AS1_MIR181A1HG	0.169204878
CDKN2B-AS1_LINC02465	0.744691284
CDKN2B-AS1_AC091906.1	0.632706878
AL449423.1_AL445490.1	0.050440743
AL449423.1_AC004988.1	0.463959694
AL449423.1_LINC02195	0.076035099
AL449423.1_AC090125.1	0.247999417
AL449423.1_LINC01048	0.421906859
AL449423.1_AC090673.1	0.678616395
AL449423.1_AL356417.2	0.739652352
AL449423.1_AC007750.1	0.284615667
AL449423.1_LINC01561	0.568922751
AL449423.1_LINC01833	0.950249716
AL449423.1_LINC00460	0.779033391
AL449423.1_LINC00536	0.492733418
AL449423.1_AP000553.1	0.918476467
AL449423.1_AATBC	0.173223601
AL449423.1_AL161431.1	0.188479482
AL449423.1_PICSAR	0.496371249
AL449423.1_LINC01615	0.483101863
AL449423.1_LINC00641	0.532635386
AL449423.1_MIR133A1HG	0.99268963
AL449423.1_AC104984.6	0.256708154
AL449423.1_AL023755.1	0.494130632
AL449423.1_AL359851.1	0.963419176
AL449423.1_AC015923.1	0.748930878
AL449423.1_AC005828.1	0.602201794
AL449423.1_AC023034.1	0.916085976
AL449423.1_AC099811.5	0.353201301
AL449423.1_FP325330.3	0.906112336
AL449423.1_AL355075.4	0.643877844
AL449423.1_RMRP	0.421117126
AL449423.1_AC037487.2	0.604020433

AL449423.1_AC006141.1	0.928903223
AL449423.1_LINC02443	0.495539953
AL449423.1_MIR181A1HG	0.991772089
AL449423.1_LINC02465	0.856626584
AL449423.1_AC091906.1	0.74075407
AL445490.1_AC004988.1	0.826177268
AL445490.1_LINC02195	0.087654378
AL445490.1_AC090125.1	0.226445521
AL445490.1_LINC01048	0.627121556
AL445490.1_AC090673.1	0.085855001
AL445490.1_AL356417.2	0.307300777
AL445490.1_AC007750.1	0.565934626
AL445490.1_LINC01561	0.470029119
AL445490.1_LINC01833	0.262780076
AL445490.1_LINC00460	0.440039763
AL445490.1_LINC00536	0.341017147
AL445490.1_AP000553.1	0.915565065
AL445490.1_AATBC	0.787494963
AL445490.1_AL161431.1	0.049556263
AL445490.1_PICSAR	0.830703693
AL445490.1_LINC01615	0.446168009
AL445490.1_LINC00641	0.631440116
AL445490.1_MIR133A1HG	0.612023135
AL445490.1_AC104984.6	0.088581714
AL445490.1_AL023755.1	0.622348942
AL445490.1_AL359851.1	0.892547429
AL445490.1_AC015923.1	0.830553656
AL445490.1_AC005828.1	0.75945564
AL445490.1_AC023034.1	0.237280056
AL445490.1_AC099811.5	0.599586875
AL445490.1_FP325330.3	0.471442462
AL445490.1_AL355075.4	0.738176617
AL445490.1_RMRP	0.194882675
AL445490.1_AC037487.2	0.654095518
AL445490.1_AC006141.1	0.944419987
AL445490.1_LINC02443	0.950972648
AL445490.1_MIR181A1HG	0.996178156
AL445490.1_LINC02465	0.683267369
AL445490.1_AC091906.1	0.818445888
U62317.2_LINC02195	0.483109276
U62317.2_AC090125.1	0.160409029
U62317.2_LINC01833	0.18597131
U62317.2_LINC00460	0.256537633
U62317.2_AATBC	0.012792428

U62317.2_AL161431.1	0.308248566
U62317.2_PICSAR	0.639202051
U62317.2_LINC01615	0.742933731
U62317.2_LINC00641	0.190429076
U62317.2_AC104984.6	0.299638174
U62317.2_AL355075.4	0.126254415
U62317.2_RMRP	0.126254415
U62317.2_AC037487.2	0.006842617
AC004988.1_LINC02195	0.879759647
AC004988.1_AC090125.1	0.224817266
AC004988.1_LINC01048	0.124669042
AC004988.1_AC090673.1	0.990289078
AC004988.1_AL356417.2	0.054775443
AC004988.1_AC007750.1	0.620202586
AC004988.1_LINC01561	0.377357367
AC004988.1_LINC01833	0.618826824
AC004988.1_LINC00460	0.71619323
AC004988.1_LINC00536	0.684379361
AC004988.1_AP000553.1	0.157665087
AC004988.1_AATBC	0.425646574
AC004988.1_AL161431.1	0.269022411
AC004988.1_PICSAR	0.119552546
AC004988.1_LINC01615	0.224398355
AC004988.1_LINC00641	0.408062094
AC004988.1_MIR133A1HG	0.203670243
AC004988.1_AC104984.6	0.345461335
AC004988.1_AL023755.1	0.200352758
AC004988.1_AL359851.1	0.122286341
AC004988.1_AC015923.1	0.109016539
AC004988.1_AC005828.1	0.257750674
AC004988.1_AC023034.1	0.198873506
AC004988.1_AC099811.5	0.84366394
AC004988.1_FP325330.3	0.11953489
AC004988.1_AL355075.4	0.629374369
AC004988.1_RMRP	0.374063555
AC004988.1_AC037487.2	0.910382851
AC004988.1_AC006141.1	0.560078312
AC004988.1_LINC02443	0.027367266
AC004988.1_MIR181A1HG	0.674910499
AC004988.1_LINC02465	0.521917273
AC004988.1_AC091906.1	0.326493457
LINC02195_AC090125.1	0.772839613
LINC02195_LINC01048	0.742887372
LINC02195_AC090673.1	0.927466235

LINC02195_AL356417.2	0.059559171
LINC02195_AC007750.1	0.899841369
LINC02195_LINC01561	0.63493154
LINC02195_LINC01833	0.950070033
LINC02195_LINC00460	0.087014026
LINC02195_LINC00536	0.267804802
LINC02195_AP000553.1	0.628809101
LINC02195_AATBC	0.66046283
LINC02195_AL161431.1	0.029940229
LINC02195_PICSAR	0.428911177
LINC02195_LINC01615	0.000585589
LINC02195_LINC00641	0.017389111
LINC02195_MIR133A1HG	0.7843443
LINC02195_AC104984.6	0.855647889
LINC02195_AL023755.1	0.77037512
LINC02195_AL359851.1	0.967931227
LINC02195_AC015923.1	0.522066589
LINC02195_AC005828.1	0.713986711
LINC02195_AC023034.1	0.293010198
LINC02195_AC099811.5	0.77193912
LINC02195_FP325330.3	0.326300016
LINC02195_AL355075.4	0.673744095
LINC02195_RMRP	0.495545104
LINC02195_AC037487.2	0.99397681
LINC02195_AC006141.1	0.929100632
LINC02195_LINC02443	0.718208415
LINC02195_MIR181A1HG	0.67534444
LINC02195_LINC02465	0.85697265
LINC02195_AC091906.1	0.674208242
AC090125.1_LINC01048	0.809984834
AC090125.1_AC090673.1	0.290857215
AC090125.1_AL356417.2	0.879065266
AC090125.1_AC007750.1	0.466942266
AC090125.1_LINC01561	0.494889789
AC090125.1_LINC01833	0.111113675
AC090125.1_LINC00460	0.328417725
AC090125.1_LINC00536	0.82608727
AC090125.1_AP000553.1	0.645889744
AC090125.1_AATBC	0.517355792
AC090125.1_AL161431.1	0.400492721
AC090125.1_PICSAR	0.734359228
AC090125.1_LINC01615	0.796983992
AC090125.1_LINC00641	0.250587915
AC090125.1_MIR133A1HG	0.748050183

AC090125.1_AC104984.6	0.727294145
AC090125.1_AL023755.1	0.523957298
AC090125.1_AL359851.1	0.575509458
AC090125.1_AC015923.1	0.59178359
AC090125.1_AC005828.1	0.876351561
AC090125.1_AC023034.1	0.593752523
AC090125.1_AC099811.5	0.987901691
AC090125.1_FP325330.3	0.391926765
AC090125.1_AL355075.4	0.628051094
AC090125.1_RMRP	0.753192811
AC090125.1_AC037487.2	0.904061968
AC090125.1_AC006141.1	0.62758448
AC090125.1_LINC02443	0.893329772
AC090125.1_MIR181A1HG	0.758589592
AC090125.1_LINC02465	0.618289346
AC090125.1_AC091906.1	0.878895184
LINC01048_AC090673.1	0.634571546
LINC01048_AL356417.2	0.780273935
LINC01048_AC007750.1	0.124765221
LINC01048_LINC01561	0.826830571
LINC01048_LINC01833	0.60699315
LINC01048_LINC00460	0.644476297
LINC01048_LINC00536	0.677082837
LINC01048_AP000553.1	0.257135034
LINC01048_AATBC	0.024402689
LINC01048_AL161431.1	0.488548351
LINC01048_PICSAR	0.592620595
LINC01048_LINC01615	0.342203854
LINC01048_LINC00641	0.048518918
LINC01048_MIR133A1HG	0.383545801
LINC01048_AC104984.6	0.136922728
LINC01048_AL023755.1	0.866376829
LINC01048_AL359851.1	0.360256765
LINC01048_AC015923.1	0.302731967
LINC01048_AC005828.1	0.674700004
LINC01048_AC023034.1	0.464879267
LINC01048_AC099811.5	0.76615277
LINC01048_FP325330.3	0.34469989
LINC01048_AL355075.4	0.671010999
LINC01048_RMRP	0.523930417
LINC01048_AC037487.2	0.255144957
LINC01048_AC006141.1	0.272599312
LINC01048_LINC02443	0.558502254
LINC01048_MIR181A1HG	0.476500106

LINC01048_LINC02465	0.731450802
LINC01048_AC091906.1	0.698997675
AC090673.1_AL356417.2	0.470193211
AC090673.1_AC007750.1	0.008626072
AC090673.1_LINC01561	0.419177583
AC090673.1_LINC01833	0.067295364
AC090673.1_LINC00460	0.408204488
AC090673.1_LINC00536	0.072945361
AC090673.1_AP000553.1	0.01080623
AC090673.1_AATBC	0.62491948
AC090673.1_AL161431.1	0.243709321
AC090673.1_PICSAR	0.012564182
AC090673.1_LINC01615	0.228143076
AC090673.1_LINC00641	0.475967588
AC090673.1_MIR133A1HG	0.009948578
AC090673.1_AC104984.6	0.076490466
AC090673.1_AL023755.1	0.03907635
AC090673.1_AL359851.1	0.017333881
AC090673.1_AC015923.1	0.024983115
AC090673.1_AC005828.1	0.009926333
AC090673.1_AC023034.1	0.204636476
AC090673.1_AC099811.5	0.018945617
AC090673.1_FP325330.3	0.002775777
AC090673.1_AL355075.4	0.064027529
AC090673.1_RMRP	0.05441831
AC090673.1_AC037487.2	0.055842206
AC090673.1_AC006141.1	0.011484992
AC090673.1_LINC02443	0.009871966
AC090673.1_MIR181A1HG	0.01508436
AC090673.1_LINC02465	0.096241947
AC090673.1_AC091906.1	0.022546873
AL356417.2_AC007750.1	0.214900425
AL356417.2_LINC01561	0.028138164
AL356417.2_LINC01833	0.167838968
AL356417.2_LINC00460	0.227537359
AL356417.2_LINC00536	0.38806202
AL356417.2_AP000553.1	0.030212286
AL356417.2_AATBC	0.080945242
AL356417.2_AL161431.1	0.357510287
AL356417.2_PICSAR	0.325929389
AL356417.2_LINC01615	0.082063946
AL356417.2_LINC00641	0.999191252
AL356417.2_MIR133A1HG	0.047703445
AL356417.2_AC104984.6	0.196303405

AL356417.2_AL023755.1	0.042258202
AL356417.2_AL359851.1	0.042012191
AL356417.2_AC015923.1	0.092200202
AL356417.2_AC005828.1	0.092213861
AL356417.2_AC023034.1	0.604141808
AL356417.2_AC099811.5	0.359535905
AL356417.2_FP325330.3	0.020666805
AL356417.2_AL355075.4	0.234013948
AL356417.2_RMRP	0.158714068
AL356417.2_AC037487.2	0.077384767
AL356417.2_AC006141.1	0.063035249
AL356417.2_LINC02443	0.407682161
AL356417.2_MIR181A1HG	0.026807411
AL356417.2_LINC02465	0.023644603
AL356417.2_AC091906.1	0.08934334
AC007750.1_LINC01561	0.647672801
AC007750.1_LINC01833	0.182511305
AC007750.1_LINC00460	0.057864914
AC007750.1_LINC00536	0.060739575
AC007750.1_AP000553.1	0.439402934
AC007750.1_AATBC	0.666915356
AC007750.1_AL161431.1	0.089374072
AC007750.1_PICSAR	0.334404281
AC007750.1_LINC01615	0.390796118
AC007750.1_LINC00641	0.669882731
AC007750.1_MIR133A1HG	0.549857156
AC007750.1_AC104984.6	0.17576474
AC007750.1_AL023755.1	0.445609961
AC007750.1_AL359851.1	0.471162281
AC007750.1_AC015923.1	0.104799324
AC007750.1_AC005828.1	0.020451021
AC007750.1_AC023034.1	0.582488987
AC007750.1_AC099811.5	0.7979846
AC007750.1_FP325330.3	0.069999568
AC007750.1_AL355075.4	0.396437995
AC007750.1_RMRP	0.133211204
AC007750.1_AC037487.2	0.15273215
AC007750.1_AC006141.1	0.14661486
AC007750.1_LINC02443	0.403458352
AC007750.1_MIR181A1HG	0.805996648
AC007750.1_LINC02465	0.456791219
AC007750.1_AC091906.1	0.721412831
LINC01561_LINC01833	0.232232287
LINC01561_LINC00460	0.787879509

LINC01561_LINC00536	0.905211789
LINC01561_AP000553.1	0.145683147
LINC01561_AATBC	0.627367155
LINC01561_AL161431.1	0.652624367
LINC01561_PICSAR	0.172122511
LINC01561_LINC01615	0.42843782
LINC01561_LINC00641	0.989999069
LINC01561_MIR133A1HG	0.107342845
LINC01561_AC104984.6	0.263322403
LINC01561_AL023755.1	0.095786612
LINC01561_AL359851.1	0.173883122
LINC01561_AC015923.1	0.165592917
LINC01561_AC005828.1	0.279080486
LINC01561_AC023034.1	0.234069685
LINC01561_AC099811.5	0.211650685
LINC01561_FP325330.3	0.104292529
LINC01561_AL355075.4	0.188907242
LINC01561_RMRP	0.926021254
LINC01561_AC037487.2	0.058830334
LINC01561_AC006141.1	0.457551652
LINC01561_LINC02443	0.198704673
LINC01561_MIR181A1HG	0.350498895
LINC01561_LINC02465	0.085820907
LINC01561_AC091906.1	0.32788784
LINC01833_LINC00460	0.056996378
LINC01833_LINC00536	0.237988515
LINC01833_AP000553.1	0.433706086
LINC01833_AATBC	0.404154633
LINC01833_AL161431.1	0.035544441
LINC01833_PICSAR	0.435523226
LINC01833_LINC01615	0.011820402
LINC01833_LINC00641	0.001453093
LINC01833_MIR133A1HG	0.509793678
LINC01833_AC104984.6	0.429770699
LINC01833_AL023755.1	0.657764916
LINC01833_AL359851.1	0.851313925
LINC01833_AC015923.1	0.877527211
LINC01833_AC005828.1	0.983961936
LINC01833_AC023034.1	0.216094901
LINC01833_AC099811.5	0.348406311
LINC01833_FP325330.3	0.39050155
LINC01833_AL355075.4	0.360337154
LINC01833_RMRP	0.260653072
LINC01833_AC037487.2	0.531863048

LINC01833_AC006141.1	0.233925211
LINC01833_LINC02443	0.466182287
LINC01833_MIR181A1HG	0.509283987
LINC01833_LINC02465	0.392990771
LINC01833_AC091906.1	0.06000991
LINC00460_LINC00536	0.80452568
LINC00460_AP000553.1	0.261627776
LINC00460_AATBC	0.334876754
LINC00460_AL161431.1	0.47039228
LINC00460_PICSAR	0.001337745
LINC00460_LINC01615	0.002963772
LINC00460_LINC00641	0.283787142
LINC00460_MIR133A1HG	0.240972309
LINC00460_AC104984.6	0.224900736
LINC00460_AL023755.1	0.054789926
LINC00460_AL359851.1	0.284228721
LINC00460_AC015923.1	0.015489491
LINC00460_AC005828.1	0.060052877
LINC00460_AC023034.1	0.187426635
LINC00460_AC099811.5	0.160927126
LINC00460_FP325330.3	0.059592094
LINC00460_AL355075.4	0.45117214
LINC00460_RMRP	0.452924121
LINC00460_AC037487.2	0.47126908
LINC00460_AC006141.1	0.15810447
LINC00460_LINC02443	0.045503797
LINC00460_MIR181A1HG	0.166347786
LINC00460_LINC02465	0.197796384
LINC00460_AC091906.1	0.29405248
LINC00536_AP000553.1	0.37055573
LINC00536_AATBC	0.000110899
LINC00536_AL161431.1	0.577312534
LINC00536_PICSAR	0.030607721
LINC00536_LINC01615	0.001138669
LINC00536_LINC00641	0.000110899
LINC00536_MIR133A1HG	0.079610098
LINC00536_AC104984.6	0.236117353
LINC00536_AL023755.1	0.020866666
LINC00536_AL359851.1	0.026474201
LINC00536_AC015923.1	0.101013827
LINC00536_AC005828.1	0.084205618
LINC00536_AC023034.1	0.197224602
LINC00536_AC099811.5	0.129033008
LINC00536_FP325330.3	0.072636026

LINC00536_AL355075.4	0.07641238
LINC00536_RMRP	0.581586016
LINC00536_AC037487.2	0.051509563
LINC00536_AC006141.1	0.055399322
LINC00536_LINC02443	0.335142698
LINC00536_MIR181A1HG	0.375081216
LINC00536_LINC02465	0.273393814
LINC00536_AC091906.1	0.362917226
AP000553.1_AL161431.1	0.474546767
AP000553.1_PICSAR	0.781492645
AP000553.1_LINC01615	0.752412835
AP000553.1_LINC00641	0.567885877
AP000553.1_MIR133A1HG	0.963449914
AP000553.1_AC104984.6	0.963449914
AP000553.1_AL023755.1	0.963449914
AP000553.1_AL359851.1	0.963449914
AP000553.1_AC015923.1	0.963449914
AP000553.1_AC005828.1	0.963449914
AP000553.1_AC023034.1	0.963449914
AP000553.1_AC099811.5	0.963449914
AP000553.1_FP325330.3	0.963449914
AP000553.1_AL355075.4	0.963449914
AP000553.1_RMRP	0.963449914
AP000553.1_AC037487.2	0.963449914
AP000553.1_AC006141.1	0.963449914
AP000553.1_LINC02443	0.963449914
AP000553.1_MIR181A1HG	0.963449914
AP000553.1_LINC02465	0.963449914
AP000553.1_AC091906.1	0.727872699
AATBC_AL161431.1	0.041139427
AATBC_PICSAR	0.16875668
AATBC_LINC01615	0.016292688
AATBC_LINC00641	0.001281406
AATBC_MIR133A1HG	0.629331086
AATBC_AC104984.6	0.220662645
AATBC_AL023755.1	0.185719619
AATBC_AC015923.1	0.98956805
AATBC_AC005828.1	0.480211135
AATBC_AC023034.1	0.055741569
AATBC_AC099811.5	0.951370039
AATBC_FP325330.3	0.492179308
AATBC_AL355075.4	0.173733574
AATBC_RMRP	0.201439148
AATBC_AC037487.2	0.297374419

AATBC_LINC02443	0.886772154
AATBC_MIR181A1HG	0.558230318
AATBC_LINC02465	0.0004446
AATBC_AC091906.1	0.346507408
AL161431.1_PICSAR	0.000468991
AL161431.1_LINC01615	0.222307148
AL161431.1_LINC00641	0.652867635
AL161431.1_MIR133A1HG	0.01051303
AL161431.1_AC104984.6	0.00027458
AL161431.1_AL023755.1	0.012659191
AL161431.1_AL359851.1	0.031741371
AL161431.1_AC015923.1	0.040641863
AL161431.1_AC005828.1	0.026678376
AL161431.1_AC023034.1	0.066461317
AL161431.1_AC099811.5	0.031272101
AL161431.1_FP325330.3	0.000835711
AL161431.1_AL355075.4	0.000716701
AL161431.1_RMRP	0.04638701
AL161431.1_AC037487.2	0.013510656
AL161431.1_AC006141.1	0.004361499
AL161431.1_LINC02443	0.002965153
AL161431.1_MIR181A1HG	0.01417887
AL161431.1_LINC02465	0.007196596
AL161431.1_AC091906.1	0.001770821
PICSAR_LINC01615	0.48129391
PICSAR_LINC00641	0.512912107
PICSAR_MIR133A1HG	0.232110598
PICSAR_AC104984.6	0.656330013
PICSAR_AL023755.1	0.980718196
PICSAR_AL359851.1	0.340050767
PICSAR_AC015923.1	0.202696889
PICSAR_AC005828.1	0.543612081
PICSAR_AC023034.1	0.704343428
PICSAR_AC099811.5	0.206982235
PICSAR_FP325330.3	0.925949414
PICSAR_AL355075.4	0.686192439
PICSAR_RMRP	0.108306414
PICSAR_AC037487.2	0.384876552
PICSAR_AC006141.1	0.188529763
PICSAR_LINC02443	0.377330856
PICSAR_MIR181A1HG	0.235088927
PICSAR_LINC02465	0.257824068
PICSAR_AC091906.1	0.218974576
LINC01615_LINC00641	0.076370836

LINC01615_MIR133A1HG	0.612979588
LINC01615_AC104984.6	0.380721657
LINC01615_AL023755.1	0.233756018
LINC01615_AL359851.1	0.486360212
LINC01615_AC015923.1	0.825903403
LINC01615_AC005828.1	0.250317492
LINC01615_AC023034.1	0.907576602
LINC01615_AC099811.5	0.625807967
LINC01615_FP325330.3	0.378594586
LINC01615_AL355075.4	0.803115234
LINC01615_RMRP	0.882310307
LINC01615_AC037487.2	0.81782013
LINC01615_AC006141.1	0.510773292
LINC01615_LINC02443	0.237743786
LINC01615_MIR181A1HG	0.972751941
LINC01615_LINC02465	0.505396262
LINC01615_AC091906.1	0.055846096
LINC00641_AC104984.6	0.508645179
LINC00641_AL023755.1	4.13E-07
LINC00641_AC015923.1	0.847241026
LINC00641_AC005828.1	0.609538103
LINC00641_AC023034.1	0.952292503
LINC00641_AC099811.5	0.002126016
LINC00641_AL355075.4	0.092055643
LINC00641_RMRP	0.133945453
LINC00641_AC037487.2	2.71E-07
LINC00641_LINC02443	0.909384629
LINC00641_MIR181A1HG	0.909384629
LINC00641_AC091906.1	0.706567578
MIR133A1HG_AC104984.6	0.589889894
MIR133A1HG_AL023755.1	0.502992249
MIR133A1HG_AL359851.1	0.439070627
MIR133A1HG_AC015923.1	0.537583198
MIR133A1HG_AC005828.1	0.276872164
MIR133A1HG_AC023034.1	0.562820729
MIR133A1HG_AC099811.5	0.134015781
MIR133A1HG_FP325330.3	0.318861019
MIR133A1HG_AL355075.4	0.182233322
MIR133A1HG_RMRP	0.166552884
MIR133A1HG_AC037487.2	0.884601906
MIR133A1HG_AC006141.1	0.997441687
MIR133A1HG_LINC02443	0.777017656
MIR133A1HG_MIR181A1HG	0.327833097
MIR133A1HG_LINC02465	0.556627413

MIR133A1HG_AC091906.1	0.134765503
AC104984.6_AL023755.1	0.031560538
AC104984.6_AL359851.1	0.030137532
AC104984.6_AC015923.1	0.149526104
AC104984.6_AC005828.1	0.134969734
AC104984.6_AC023034.1	0.07740784
AC104984.6_AC099811.5	0.091306807
AC104984.6_FP325330.3	0.196297381
AC104984.6_AL355075.4	0.031365563
AC104984.6_RMRP	0.067018774
AC104984.6_AC037487.2	0.134939598
AC104984.6_AC006141.1	0.158596172
AC104984.6_LINC02443	0.06291827
AC104984.6_MIR181A1HG	0.113115476
AC104984.6_LINC02465	0.057970361
AC104984.6_AC091906.1	0.481256177
AL023755.1_AL359851.1	0.019455952
AL023755.1_AC015923.1	0.386445237
AL023755.1_AC005828.1	0.372541238
AL023755.1_AC023034.1	0.07668532
AL023755.1_AC099811.5	0.233616784
AL023755.1_FP325330.3	0.08913116
AL023755.1_AL355075.4	0.792544762
AL023755.1_RMRP	0.021464435
AL023755.1_AC037487.2	0.073022111
AL023755.1_AC006141.1	0.068690081
AL023755.1_LINC02443	0.158800104
AL023755.1_MIR181A1HG	0.142277779
AL023755.1_LINC02465	0.007964118
AL023755.1_AC091906.1	0.702446506
AL359851.1_AC015923.1	0.166281586
AL359851.1_AC005828.1	0.557386943
AL359851.1_AC023034.1	0.108100802
AL359851.1_AC099811.5	0.105059513
AL359851.1_FP325330.3	0.24814934
AL359851.1_AL355075.4	0.483087516
AL359851.1_RMRP	0.044048856
AL359851.1_AC037487.2	0.367175406
AL359851.1_AC006141.1	0.877351298
AL359851.1_LINC02443	0.162364087
AL359851.1_MIR181A1HG	0.055169371
AL359851.1_LINC02465	0.014699021
AL359851.1_AC091906.1	0.253228553
AC015923.1_AC005828.1	0.158093725

AC015923.1_AC023034.1	0.317038125
AC015923.1_AC099811.5	0.110902504
AC015923.1_FP325330.3	0.424252614
AC015923.1_AL355075.4	0.175990465
AC015923.1_RMRP	0.101281978
AC015923.1_AC037487.2	0.284182338
AC015923.1_AC006141.1	0.153330643
AC015923.1_LINC02443	0.401506676
AC015923.1_MIR181A1HG	0.026390734
AC015923.1_LINC02465	0.068132831
AC015923.1_AC091906.1	0.207491854
AC005828.1_AC023034.1	0.965728304
AC005828.1_AC099811.5	0.352264773
AC005828.1_FP325330.3	0.510167175
AC005828.1_AL355075.4	0.085963014
AC005828.1_RMRP	0.316218039
AC005828.1_AC037487.2	0.590269057
AC005828.1_AC006141.1	0.223963833
AC005828.1_LINC02443	0.615090915
AC005828.1_MIR181A1HG	0.150821336
AC005828.1_LINC02465	0.103113236
AC005828.1_AC091906.1	0.331173227
AC023034.1_AC099811.5	0.764117127
AC023034.1_FP325330.3	0.891756295
AC023034.1_AL355075.4	0.68220102
AC023034.1_RMRP	0.787291409
AC023034.1_AC037487.2	0.460241015
AC023034.1_AC006141.1	0.876949645
AC023034.1_LINC02443	0.651296899
AC023034.1_MIR181A1HG	0.902928126
AC023034.1_LINC02465	0.955288049
AC023034.1_AC091906.1	0.231671406
AC099811.5_FP325330.3	0.753237632
AC099811.5_AL355075.4	0.827567423
AC099811.5_RMRP	0.148347066
AC099811.5_AC037487.2	0.539464141
AC099811.5_AC006141.1	0.872964732
AC099811.5_LINC02443	0.980938009
AC099811.5_MIR181A1HG	0.602882475
AC099811.5_LINC02465	0.235095175
AC099811.5_AC091906.1	0.368630893
FP325330.3_AL355075.4	0.142597158
FP325330.3_RMRP	0.892244872
FP325330.3_AC037487.2	0.250781454

FP325330.3_AC006141.1	0.246795011
FP325330.3_LINC02443	0.489633351
FP325330.3_MIR181A1HG	0.952726907
FP325330.3_LINC02465	0.713833039
FP325330.3_AC091906.1	0.747057364
AL355075.4_RMRP	0.117022312
AL355075.4_AC037487.2	0.867520492
AL355075.4_AC006141.1	0.139254197
AL355075.4_LINC02443	0.341434914
AL355075.4_MIR181A1HG	0.022251961
AL355075.4_LINC02465	0.019335464
AL355075.4_AC091906.1	0.986597319
RMRP_AC037487.2	0.247719782
RMRP_AC006141.1	0.256551045
RMRP_LINC02443	0.205998296
RMRP_MIR181A1HG	0.423134273
RMRP_LINC02465	0.480292012
RMRP_AC091906.1	0.165605525
AC037487.2_AC006141.1	0.498541502
AC037487.2_LINC02443	0.897673789
AC037487.2_MIR181A1HG	0.318505491
AC037487.2_LINC02465	0.289039088
AC037487.2_AC091906.1	0.654483032
AC006141.1_LINC02443	0.551382704
AC006141.1_MIR181A1HG	0.130199617
AC006141.1_LINC02465	0.080517732
AC006141.1_AC091906.1	0.573257846
LINC02443_MIR181A1HG	0.280820901
LINC02443_LINC02465	0.327081604
LINC02443_AC091906.1	0.916181576
MIR181A1HG_LINC02465	0.532838315
MIR181A1HG_AC091906.1	0.864673697
LINC02465_AC091906.1	0.770402146
