# Supplementary material for: Development of a Ferroptosis-Related lncRNA Signature to Predict the Prognosis and Immune Landscape of Bladder Cancer
Source: Dis Markers. 2021 Jun 20;2021:1031906. doi: 10.1155/2021/1031906 (PMC8238626; doi:10.1155/2021/1031906)
Supplement: Supplementary Materials — Supplementary Figure S1: validation of the risk model with resampling. Note: (a) Kaplan-Meier survival analysis of cases in high-risk group and low-risk group (p < 0.001). (b) ROC curves of the risk score in internal validation dataset. Supplementary Figure S2: Kaplan-Meier subgroup survival analysis in TCGA-BLCA patients. Note: (a) age ≤ 64 (p < 0.001). (b) Age > 64 (p < 0.001). (c) Male (p < 0.001). (d) Female (p < 0.001). (e) Pathological T 1-2 stages (p < 0.001). (f) Pathological T 3-4 stages (p < 0.001). (g) N0 stages (p < 0.001). (h) N1-N3 stages (p < 0.001). (i) M0 stage (p < 0.001). (j) M1 stage (p < 0.05). Supplementary Figure S3: functional prediction of AC090825.1 and MAGI2-AS3. Note: (a) the expression difference of AC090825.1 in BCa samples and paracarcinoma tissue via Wilcoxon signed-rank test. (b) The expression difference of MAGI2-AS3 in BCa samples and paracarcinoma tissue via Wilcoxon signed-rank test. (c) The difference values between the expression of AC090825.1 and MAGI2-AS3 in BCa samples and paracarcinoma samples. (d) The cases suffered poorer survival when the expression value of AC090825.1 was higher than that of MAGI2-AS3 (p < 0.01). (e) The expression levels of AC090825.1 and MAGI2-AS3 were positively correlated both in BCa samples and paracarcinoma samples. (f) Network of lncRNAs, the target genes and their possible transcription factors. (g) UpSet diagram showing 6 overlapped transcription factors. (h) The correlation network of lncRNAs, the target genes and transcription factors. BCa: bladder cancer. Supplementary Table S1: 20 ferroptosis regulators were differentially expressed between BCa and paracarinoma samples. Supplementary Table S2: the Pearson correlation analysis of lncRNAs and ferroptosis regulators. Supplementary Table S3: Kaplan-Meier survival analysis of the lncRNA pairs. Supplementary Table S4: enrichment results from LncSEA website. Supplementary Table S5: GSVA results of different clusters based on ferroptosis regulators [file 1031906.f1.zip › Supplementary_Table_S4.PDF]

Supplementary Table S4. Enrichment results from LncSEA website.

Class	Sub_Class	Simpson	P-value	FDR	Bonferroni	Jaccard	Input_number
Disease	LncRNADisease2.0	0.5	3.21E-07	0.000124	0.000124	0.0789	36
Disease	Lnc2Cancer2.0	0.333	9.78E-05	0.0147	0.0148	0.0513	36
Disease	Lnc2Cancer2.0	0.111	0.000195	0.0147	0.0294	0.028	36
Cancer_Hallmark	_	0.0833	0.000431	0.0302	0.0302	0.0189	36
Cancer_Phenotype	Malignant_neoplasm_of_colon_with_rectum	0.111	2.62E-05	0.000393	0.000393	0.0408	36
Cancer_Phenotype	Breast_Carcinoma	0.286	0.000137	0.0101	0.0101	0.05	36
Cancer_Phenotype	Esophageal_carcinoma	0.222	0.000234	0.00515	0.00515	0.0476	36
Cancer_Phenotype	Malignant_neoplasm_of_colon_with_rectum	0.0833	0.000659	0.00494	0.00988	0.0306	36
Cancer_Phenotype	Stomach_Carcinoma	0.125	0.00077	0.0186	0.0293	0.0408	36
Cancer_Phenotype	Stomach_Carcinoma	0.111	0.000979	0.0186	0.0372	0.0392	36
Cancer_Phenotype	Malignant_neoplasm_of_colon_with_rectum	0.139	0.00213	0.00979	0.032	0.013	36
Cancer_Phenotype	Malignant_neoplasm_of_colon_with_rectum	0.139	0.00261	0.00979	0.0392	0.0124	36
Cancer_Phenotype	Carcinoma_of_bladder	0.167	0.00297	0.0115	0.0267	0.0102	36
Cancer_Phenotype	Carcinoma_of_bladder	0.167	0.00321	0.0115	0.0289	0.01	36
Cancer_Phenotype	Carcinoma_of_bladder	0.167	0.00384	0.0115	0.0346	0.00968	36
Cancer_Phenotype	Colorectal_Carcinoma	0.5	0.00519	0.00519	0.00519	0.0278	36
