# Supplementary material for: Development of a Ferroptosis-Related lncRNA Signature to Predict the Prognosis and Immune Landscape of Bladder Cancer
Source: Dis Markers. 2021 Jun 20;2021:1031906. doi: 10.1155/2021/1031906 (PMC8238626; doi:10.1155/2021/1031906)
Supplement: Supplementary Materials — Supplementary Figure S1: validation of the risk model with resampling. Note: (a) Kaplan-Meier survival analysis of cases in high-risk group and low-risk group (p < 0.001). (b) ROC curves of the risk score in internal validation dataset. Supplementary Figure S2: Kaplan-Meier subgroup survival analysis in TCGA-BLCA patients. Note: (a) age ≤ 64 (p < 0.001). (b) Age > 64 (p < 0.001). (c) Male (p < 0.001). (d) Female (p < 0.001). (e) Pathological T 1-2 stages (p < 0.001). (f) Pathological T 3-4 stages (p < 0.001). (g) N0 stages (p < 0.001). (h) N1-N3 stages (p < 0.001). (i) M0 stage (p < 0.001). (j) M1 stage (p < 0.05). Supplementary Figure S3: functional prediction of AC090825.1 and MAGI2-AS3. Note: (a) the expression difference of AC090825.1 in BCa samples and paracarcinoma tissue via Wilcoxon signed-rank test. (b) The expression difference of MAGI2-AS3 in BCa samples and paracarcinoma tissue via Wilcoxon signed-rank test. (c) The difference values between the expression of AC090825.1 and MAGI2-AS3 in BCa samples and paracarcinoma samples. (d) The cases suffered poorer survival when the expression value of AC090825.1 was higher than that of MAGI2-AS3 (p < 0.01). (e) The expression levels of AC090825.1 and MAGI2-AS3 were positively correlated both in BCa samples and paracarcinoma samples. (f) Network of lncRNAs, the target genes and their possible transcription factors. (g) UpSet diagram showing 6 overlapped transcription factors. (h) The correlation network of lncRNAs, the target genes and transcription factors. BCa: bladder cancer. Supplementary Table S1: 20 ferroptosis regulators were differentially expressed between BCa and paracarinoma samples. Supplementary Table S2: the Pearson correlation analysis of lncRNAs and ferroptosis regulators. Supplementary Table S3: Kaplan-Meier survival analysis of the lncRNA pairs. Supplementary Table S4: enrichment results from LncSEA website. Supplementary Table S5: GSVA results of different clusters based on ferroptosis regulators [file 1031906.f1.zip › Supplementary_Table_S5.PDF]

Supplementary Table S5. GSVA results of different clusters based on ferroptosis regulators.

id	logFC	AveExpr	t	P.Value	adj.P.Val	B
GO_IRON_ION_TRANSMEMBRANE_TRANSPORTER_ACTIVITY	-0.151747806	-0.032808502	-7.800374719	5.13E-14	1.23E-12	21.172064833
GO_IRON_IMPORT_INTO_CELL	-0.106390648	-0.002779266	-5.283960457	2.05E-07	4.72E-06	6.392781383
GO_IRON_ION_TRANSMEMBRANE_TRANSPORT	-0.081930211	-0.022079982	-4.899247303	1.39E-06	3.05E-05	4.56042007
GO_IRON_ION_HOMEOSTASIS	-0.049656195	-0.012143087	-4.769266573	2.57E-06	5.14E-05	3.969101129
HALLMARK_HEME_METABOLISM	-0.039378097	-0.018275256	-4.558575094	6.80E-06	0.000129215	3.04091283
GO_CELLULAR_IRON_ION_HOMEOSTASIS	-0.049950849	-0.011248664	-4.521612501	8.04E-06	0.000144652	2.881974671
GO_RESPONSE_TO_IRON_ION	-0.039203977	-0.004511982	-3.157067686	0.001711	0.02908625	-2.143508019
GO_HEME_TRANSPORT	0.052710074	-0.021230434	2.869425083	0.0043241	0.069185796	-2.987049866
GO_IRON_ION_BINDING	0.022738458	0.005525278	2.836307658	0.0047892	0.071838053	-3.079213012
GO_2_IRON_2_SULFUR_CLUSTER_BINDING	-0.055603373	-0.033355534	-2.767408834	0.0059053	0.082674715	-3.267654796
GO_4_IRON_4_SULFUR_CLUSTER_BINDING	-0.042069226	-0.040574758	-2.670021557	0.007885	0.102505355	-3.526394548
HEME_BIOSYNTHETIC_PROCESS	0.043463019	-0.022977801	2.540074423	0.0114496	0.137395516	-3.85768606
GO_IRON_ION_TRANSPORT	-0.025256552	-0.010056797	-2.352629488	0.0191112	0.210223586	-4.307315952

GO_IRON_COORDINATION_ENTITY_TRANSPORT	0.033394454	-0.011294149	2.013575514	0.0447046	0.447045767	-5.035202846
GO_IRON_SULFUR_CLUSTER_ASSEMBLY	-0.041017065	-0.044431968	-1.985641236	0.0477364	0.447045767	-5.090238019
REACTOME_IRON_UPTAKE_AND_TRANSPORT	-0.023482383	-0.018461995	-1.882217381	0.0605118	0.484094687	-5.287429802
MODULE_540	-0.031917229	0.009791014	-1.619659767	0.1060714	0.742499642	-5.741391135
GO_SEQUESTERING_OF_IRON_ION	0.039762743	0.038013927	1.460418292	0.1449381	0.869628731	-5.983971544
GO_PROTOPORPHYRINOGEN_IX_METABOLIC_PROCESS	-0.021987366	-0.029508842	-1.281836852	0.2006215	1	-6.226465203
GO_MULTICELLULAR_ORGANISMAL_IRON_ION_HOMEOSTASIS	-0.011799719	0.011672524	-0.570466566	0.5686726	1	-6.880381035
GO_HEME_METABOLIC_PROCESS	0.005977929	-0.018416186	0.502223313	0.615779	1	-6.916771348
GO_REGULATION_OF_IRON_ION_TRANSPORT	-0.007279251	0.010472505	-0.319118668	0.7497984	1	-6.991552828
GO_CELLULAR_RESPONSE_TO_IRON_ION	-0.00481431	-0.013349973	-0.276271209	0.7824785	1	-7.004240755
