# Supplementary material for: Development of a Ferroptosis-Related lncRNA Signature to Predict the Prognosis and Immune Landscape of Bladder Cancer
Source: Dis Markers. 2021 Jun 20;2021:1031906. doi: 10.1155/2021/1031906 (PMC8238626; doi:10.1155/2021/1031906)
Supplement: Supplementary Materials — Supplementary Figure S1: validation of the risk model with resampling. Note: (a) Kaplan-Meier survival analysis of cases in high-risk group and low-risk group (p < 0.001). (b) ROC curves of the risk score in internal validation dataset. Supplementary Figure S2: Kaplan-Meier subgroup survival analysis in TCGA-BLCA patients. Note: (a) age ≤ 64 (p < 0.001). (b) Age > 64 (p < 0.001). (c) Male (p < 0.001). (d) Female (p < 0.001). (e) Pathological T 1-2 stages (p < 0.001). (f) Pathological T 3-4 stages (p < 0.001). (g) N0 stages (p < 0.001). (h) N1-N3 stages (p < 0.001). (i) M0 stage (p < 0.001). (j) M1 stage (p < 0.05). Supplementary Figure S3: functional prediction of AC090825.1 and MAGI2-AS3. Note: (a) the expression difference of AC090825.1 in BCa samples and paracarcinoma tissue via Wilcoxon signed-rank test. (b) The expression difference of MAGI2-AS3 in BCa samples and paracarcinoma tissue via Wilcoxon signed-rank test. (c) The difference values between the expression of AC090825.1 and MAGI2-AS3 in BCa samples and paracarcinoma samples. (d) The cases suffered poorer survival when the expression value of AC090825.1 was higher than that of MAGI2-AS3 (p < 0.01). (e) The expression levels of AC090825.1 and MAGI2-AS3 were positively correlated both in BCa samples and paracarcinoma samples. (f) Network of lncRNAs, the target genes and their possible transcription factors. (g) UpSet diagram showing 6 overlapped transcription factors. (h) The correlation network of lncRNAs, the target genes and transcription factors. BCa: bladder cancer. Supplementary Table S1: 20 ferroptosis regulators were differentially expressed between BCa and paracarinoma samples. Supplementary Table S2: the Pearson correlation analysis of lncRNAs and ferroptosis regulators. Supplementary Table S3: Kaplan-Meier survival analysis of the lncRNA pairs. Supplementary Table S4: enrichment results from LncSEA website. Supplementary Table S5: GSVA results of different clusters based on ferroptosis regulators [file 1031906.f1.zip › Supplementary_Table_S6.PDF]

Supplementary Table S6. GSVA results of different risk subgroups based on frlncRNA signature.

id	logFC	AveExpr	t	P.Value	adj.P.Val	B
MODULE_540	0.075127249	0.010193773	3.695994365	0.0002496	0.005740794	0.009758712
GO_4_IRON_4_SULFUR_CLUSTER_BINDING	-0.058588567	-0.041018879	-3.543247002	0.000441967	0.009723275	-0.516820374
GO_IRON_SULFUR_CLUSTER_ASSEMBLY	-0.076270067	-0.044337002	-3.541911235	0.000444142	0.009723275	-0.521333478
GO_SEQUESTERING_OF_IRON_ION	0.093402211	0.038053515	3.291465394	0.001085321	0.021706428	-1.339177041
GO_HEME_TRANSPORT	-0.065357992	-0.02227842	-3.278284755	0.001135885	0.021706428	-1.380650903
GO_CELLULAR_IRON_ION_HOMEOSTASIS	0.036807403	-0.011087988	3.22131064 5	0.001380614	0.024851043	-1.558110535
GO_MULTICELLULAR_ORGANISMAL_IRON_ION_HOMEOSTASIS	0.066063519	0.011921837	3.081360939	0.002203319	0.037456416	-1.981469623
GO_IRON_IMPORT_INTO_CELL	0.064246779	-0.002682862	2.939650568	0.00347676	0.055628154	-2.391883631
GO_IRON_ION_HOMEOSTASIS	0.031261069	-0.011953805	2.885889515	0.004114907	0.0617236	-2.542748144
GO_RESPONSE_TO_IRON_ION	0.036578172	-0.004878373	2.820098674	0.0050402	0.070562806	-2.723737518
GO_IRON_ION_BINDING	0.019291184	0.00520826	2.261197476	0.024284398	0.315697178	-4.098465697
GO_HEME_METABOLIC_PROCESS	-0.02658728	-0.019157496	-2.103657558	0.03603324	0.432398874	-4.432808852
HEME_BIOSYNTHETIC_PROCESS	-0.036701962	-0.022044884	-1.969762915	0.049556272	0.545118994	-4.698409857
GO_IRON_ION_TRANSMEMBRANE	0.0422	-0.033	1.9657	0.050	0.545	-4.706

_TRANSPORTER_ACTIVITY	55932	31226	67828	017945	118994	071897
GO_IRON_COORDINATION_ENTITY_TRANSPORT	-0.032279771	-0.011775791	-1.816511016	0.070041638	0.630374738	-4.981381269
HALLMARK_HEME_METABOLISM	0.011017288	-0.017889426	1.241398003	0.215187472	1	-5.841559741
GO_IRON_ION_TRANSPORT	0.013426759	-0.010024673	1.201520012	0.230261669	1	-5.889320919
GO_2_IRON_2_SULFUR_CLUSTER_BINDING	-0.023455992	-0.032958727	-1.111967974	0.26682093	1	-5.990932607
GO_IRON_ION_TRANSMEMBRANE_TRANSPORT	0.01803015	-0.022367967	1.019186425	0.30873155	1	-6.08796148
GO_PROTOPORPHYRINOGEN_IX_METABOLIC_PROCESS	-0.0140828	-0.029711937	-0.779673563	0.436044897	1	-6.299552821
REACTOME_IRON_UPTAKE_AND_TRANSPORT	0.009698765	-0.018593467	0.750878768	0.45316787	1	-6.321209723
GO_REGULATION_OF_IRON_ION_TRANSPORT	0.00914345	0.011222425	0.380979275	0.703421233	1	-6.527066803
GO_CELLULAR_RESPONSE_TO_IRON_ION	0.0023433	-0.012473918	0.12769936	0.89845118	1	-6.59045922
