# Supplementary material for: Development of a Ferroptosis-Related lncRNA Signature to Predict the Prognosis and Immune Landscape of Bladder Cancer
Source: Dis Markers. 2021 Jun 20;2021:1031906. doi: 10.1155/2021/1031906 (PMC8238626; doi:10.1155/2021/1031906)
Supplement: Supplementary Materials — Supplementary Figure S1: validation of the risk model with resampling. Note: (a) Kaplan-Meier survival analysis of cases in high-risk group and low-risk group (p < 0.001). (b) ROC curves of the risk score in internal validation dataset. Supplementary Figure S2: Kaplan-Meier subgroup survival analysis in TCGA-BLCA patients. Note: (a) age ≤ 64 (p < 0.001). (b) Age > 64 (p < 0.001). (c) Male (p < 0.001). (d) Female (p < 0.001). (e) Pathological T 1-2 stages (p < 0.001). (f) Pathological T 3-4 stages (p < 0.001). (g) N0 stages (p < 0.001). (h) N1-N3 stages (p < 0.001). (i) M0 stage (p < 0.001). (j) M1 stage (p < 0.05). Supplementary Figure S3: functional prediction of AC090825.1 and MAGI2-AS3. Note: (a) the expression difference of AC090825.1 in BCa samples and paracarcinoma tissue via Wilcoxon signed-rank test. (b) The expression difference of MAGI2-AS3 in BCa samples and paracarcinoma tissue via Wilcoxon signed-rank test. (c) The difference values between the expression of AC090825.1 and MAGI2-AS3 in BCa samples and paracarcinoma samples. (d) The cases suffered poorer survival when the expression value of AC090825.1 was higher than that of MAGI2-AS3 (p < 0.01). (e) The expression levels of AC090825.1 and MAGI2-AS3 were positively correlated both in BCa samples and paracarcinoma samples. (f) Network of lncRNAs, the target genes and their possible transcription factors. (g) UpSet diagram showing 6 overlapped transcription factors. (h) The correlation network of lncRNAs, the target genes and transcription factors. BCa: bladder cancer. Supplementary Table S1: 20 ferroptosis regulators were differentially expressed between BCa and paracarinoma samples. Supplementary Table S2: the Pearson correlation analysis of lncRNAs and ferroptosis regulators. Supplementary Table S3: Kaplan-Meier survival analysis of the lncRNA pairs. Supplementary Table S4: enrichment results from LncSEA website. Supplementary Table S5: GSVA results of different clusters based on ferroptosis regulators [file 1031906.f1.zip › Supplementary_Table_S7.PDF]

Supplementary Table S7. The prediction of the transcription factors of AC090825.1.

seqname	source	feature	start	end	score	strand	frame	attributes
AC090825.1|NR_135737	TFBS	TFBS	565	570	1	-	.	TF=ZNF354C;class=C2H2 zinc finger factors;sequence=ATCCAC
AC090825.1|NR_135737	TFBS	TFBS	2021	2026	1	-	.	TF=ZNF354C;class=C2H2 zinc finger factors;sequence=ATCCAC
AC090825.1|NR_135737	TFBS	TFBS	1588	1594	1	+	.	TF=NFATC2;class=Rel homology region (RHR) factors;sequence=TTTTCCA
AC090825.1|NR_135737	TFBS	TFBS	1947	1956	1	+	.	TF=KLF5;class=C2H2 zinc finger factors;sequence=GCCCCGCCCC
AC090825.1|NR_135737	TFBS	TFBS	1952	1961	1	+	.	TF=KLF5;class=C2H2 zinc finger factors;sequence=GCCCCGCCCC
AC090825.1|NR_135737	TFBS	TFBS	2261	2268	1	-	.	TF=LBX1;class=Homeo domain factors;sequence=TTAATTAG
AC090825.1|NR_135737	TFBS	TFBS	2260	2269	1	+	.	TF=ALX3;class=Homeo domain factors;sequence=TCTAATTAAA
AC090825.1|NR_135737	TFBS	TFBS	2261	2268	1	+	.	TF=ISX;class=Homeo domain factors;sequence=CTAATTAA
AC090825.1|NR_135737	TFBS	TFBS	2261	2268	1	+	.	TF=NKX6-2;class=Homeo domain factors;sequence=CTAATTAA
AC090825.1|NR_135737	TFBS	TFBS	292	300	1	-	.	TF=PITX3;class=Homeo domain factors;sequence=CTTAATCCC
AC090825.1|N	TFB	TFBS	226	22	1	+	.	TF=PRRX1;class=Homeo

R_135737	S		1	68				domain factors;sequence=CTAATTAA
AC090825.1|NR_135737	TFBS	TFBS	2261	2268	1	+	.	TF=UNCX;class=Homeo domain factors;sequence=CTAATTAA
AC090825.1|NR_135737	TFBS	TFBS	1487	1493	1	+	.	TF=MEIS1;class=Homeo domain factors;sequence=TTGACAG
AC090825.1|NR_135737	TFBS	TFBS	1487	1494	1	+	.	TF=MEIS3;class=Homeo domain factors;sequence=TTGACAGG
AC090825.1|NR_135737	TFBS	TFBS	981	990	1	-	.	TF=RBPJ;class=Rel homology region (RHR) factors;sequence=CCTGGGAAAG
AC090825.1|NR_135737	TFBS	TFBS	301	312	1	+	.	TF=ZNF384;class=C2H2 zinc finger factors;sequence=TTTAAAAAAAAA
AC090825.1|NR_135737	TFBS	TFBS	711	718	1	-	.	TF=VDR;class=Nuclear receptors with C4 zinc fingers;sequence=TGAGTTCA
AC090825.1|NR_135737	TFBS	TFBS	2389	2396	1	-	.	TF=VDR;class=Nuclear receptors with C4 zinc fingers;sequence=TGAGTTCA
AC090825.1|NR_135737	TFBS	TFBS	2260	2269	1	+	.	TF=ARGFX;class=Homeo domain factors;sequence=TCTAATTAAA
AC090825.1|NR_135737	TFBS	TFBS	1952	1962	1	+	.	TF=KLF15;class=C2H2 zinc finger factors;sequence=GCCCCGCCCCC
AC090825.1|NR_135737	TFBS	TFBS	1142	1157	1	+	.	TF=ZNF460;class=C2H2 zinc finger factors;sequence=GCC

								TCAGCCTCCCGAG
AC090825.1|NR_135737	TFBS	TFBS	2261	2268	1	+	.	TF=LHX9;class=Homeo domain factors;sequence=CTAATTAA
AC090825.1|NR_135737	TFBS	TFBS	2261	2268	1	+	.	TF=PRRX2;class=Homeo domain factors;sequence=CTAATTAA
AC090825.1|NR_135737	TFBS	TFBS	292	299	0.999852312	−	.	TF=RHOXF1;class=Homeo domain factors;sequence=TTAATCCC
AC090825.1|NR_135737	TFBS	TFBS	2261	2268	0.999222304	−	.	TF=SHOX;class=Homeo domain factors;sequence=TTAATTAG
AC090825.1|NR_135737	TFBS	TFBS	1300	1306	0.999127731	+	.	TF=MEIS1;class=Homeo domain factors;sequence=ATGACAG
AC090825.1|NR_135737	TFBS	TFBS	1434	1440	0.999127731	+	.	TF=MEIS1;class=Homeo domain factors;sequence=ATGACAG
AC090825.1|NR_135737	TFBS	TFBS	1680	1686	0.999127731	+	.	TF=MEIS1;class=Homeo domain factors;sequence=ATGACAG
AC090825.1|NR_135737	TFBS	TFBS	2261	2268	0.999094201	+	.	TF=RAX2;class=Homeo domain factors;sequence=CTAATTAA
AC090825.1|NR_135737	TFBS	TFBS	2261	2268	0.998897959	+	.	TF=NKX6-1;class=Homeo domain factors;sequence=CTAATTAA
AC090825.1|NR_135737	TFBS	TFBS	2111	2118	0.998798659	−	.	TF=RHOXF1;class=Homeo domain factors;sequence=TTAATCCA
AC090825.1|NR_135737	TFBS	TFBS	2261	2268	0.998712673	+	.	TF=LHX5;class=Homeo domain factors;sequence=CTA

									ATTAA
AC090825.1|NR_135737	TFBS	TFBS	2261	2268	0.998636203	-	.		TF=TLX2;class=Homeo domain factors;sequence=TTAATTAG
AC090825.1|NR_135737	TFBS	TFBS	2260	2269	0.998479883	+	.		TF=MIXL1;class=Homeo domain factors;sequence=TCTAATTAAA
AC090825.1|NR_135737	TFBS	TFBS	1487	1494	0.998331277	+	.		TF=MEIS2;class=Homeo domain factors;sequence=TTGACAGG
AC090825.1|NR_135737	TFBS	TFBS	292	299	0.998227995	-	.		TF=PITX2;class=Homeo domain factors;sequence=TTAATCCC
AC090825.1|NR_135737	TFBS	TFBS	777	786	0.995670475	-	.		TF=MYB;class=Tryptophan cluster factors;sequence=ACCAACTGTA
AC090825.1|NR_135737	TFBS	TFBS	1257	1264	0.995237296	-	.		TF=NR2C2(var.2);class=Nuclear receptors with C4 zinc fingers;sequence=GAGGTCAG
AC090825.1|NR_135737	TFBS	TFBS	1391	1398	0.995237296	-	.		TF=NR2C2(var.2);class=Nuclear receptors with C4 zinc fingers;sequence=GAGGTCAG
AC090825.1|NR_135737	TFBS	TFBS	683	689	0.994989704	+	.		TF=MEIS1;class=Homeo domain factors;sequence=CTGACAG
AC090825.1|NR_135737	TFBS	TFBS	1734	1740	0.994989704	-	.		TF=MEIS1;class=Homeo domain factors;sequence=CTGACAG
AC090825.1|NR_135737	TFBS	TFBS	302	313	0.994914257	+	.		TF=ZNF384;class=C2H2 zinc finger factors;sequence=TTAAAAAAAAAA
AC090825.1|N	TFB	TFBS	125	12	0.9948	-	.		TF=NR2C1;class=Nucle

R_135737	S		7	65	46925			ar receptors with C4 zinc fingers;sequence=CGAGGTCAG
AC090825.1|NR_135737	TFBS	TFBS	1391	1399	0.994846925	-	.	TF=NR2C1;class=Nuclear receptors with C4 zinc fingers;sequence=CGAGGTCAG
AC090825.1|NR_135737	TFBS	TFBS	303	314	0.994832864	+	.	TF=ZNF384;class=C2H2 zinc finger factors;sequence=TAAAAAAAAAA
AC090825.1|NR_135737	TFBS	TFBS	292	299	0.994778353	-	.	TF=PITX1;class=Homeo domain factors;sequence=TTAATCCC
AC090825.1|NR_135737	TFBS	TFBS	1811	1816	0.994048003	+	.	TF=ZNF354C;class=C2H2 zinc finger factors;sequence=CTCCAC
AC090825.1|NR_135737	TFBS	TFBS	823	830	0.993795438	-	.	TF=TEAD3;class=TEA domain factors;sequence=ACATTCCT
AC090825.1|NR_135737	TFBS	TFBS	121	128	0.993704332	+	.	TF=HOXC4;class=Homeo domain factors;sequence=CTCATTAA
AC090825.1|NR_135737	TFBS	TFBS	2261	2268	0.993695647	-	.	TF=DRGX;class=Homeo domain factors;sequence=TTAATTAG
AC090825.1|NR_135737	TFBS	TFBS	2261	2268	0.993480683	+	.	TF=PDX1;class=Homeo domain factors;sequence=CTAATTAA
AC090825.1|NR_135737	TFBS	TFBS	304	315	0.993230986	+	.	TF=ZNF384;class=C2H2 zinc finger factors;sequence=AAAAAAAAAAA
AC090825.1|NR_135737	TFBS	TFBS	305	316	0.993230986	+	.	TF=ZNF384;class=C2H2 zinc finger factors;sequence=AAA

								AAAAAAAAA
AC090825.1|NR_135737	TFBS	TFBS	306	317	0.993230986	+	.	TF=ZNF384;class=C2H2 zinc finger factors;sequence=AAAAAAAAAAAA
AC090825.1|NR_135737	TFBS	TFBS	1032	1043	0.993230986	−	.	TF=ZNF384;class=C2H2 zinc finger factors;sequence=AAAAAAAAAAAA
AC090825.1|NR_135737	TFBS	TFBS	1033	1044	0.993230986	−	.	TF=ZNF384;class=C2H2 zinc finger factors;sequence=AAAAAAAAAAAA
AC090825.1|NR_135737	TFBS	TFBS	1034	1045	0.993230986	−	.	TF=ZNF384;class=C2H2 zinc finger factors;sequence=AAAAAAAAAAAA
AC090825.1|NR_135737	TFBS	TFBS	1035	1046	0.993230986	−	.	TF=ZNF384;class=C2H2 zinc finger factors;sequence=AAAAAAAAAAAA
AC090825.1|NR_135737	TFBS	TFBS	1036	1047	0.993230986	−	.	TF=ZNF384;class=C2H2 zinc finger factors;sequence=AAAAAAAAAAAA
AC090825.1|NR_135737	TFBS	TFBS	1947	1957	0.993184677	+	.	TF=KLF15;class=C2H2 zinc finger factors;sequence=GCCCCGCCCCG
AC090825.1|NR_135737	TFBS	TFBS	2260	2269	0.992952069	−	.	TF=MIXL1;class=Homeo domain factors;sequence=TTTAATTAGA
AC090825.1|NR_135737	TFBS	TFBS	2261	2268	0.99286404	+	.	TF=DRGX;class=Homeo domain factors;sequence=CTAATTAA
AC090825.1|NR_135737	TFBS	TFBS	2261	2268	0.992269432	+	.	TF=GSX2;class=Homeo domain factors;sequence=CTAATTAA
AC090825.1|NR_135737	TFBS	TFBS	2261	2268	0.99206191	+	.	TF=TLX2;class=Homeo domain factors;sequence=CTA

								ATTAA
AC090825.1|NR_135737	TFBS	TFBS	2261	2268	0.99154001	+	.	TF=DLX6;class=Homeo domain factors;sequence=CTAATTAA
AC090825.1|NR_135737	TFBS	TFBS	1587	1596	0.990914365	−	.	TF=NFATC4;class=Rel homology region (RHR) factors;sequence=GATGGAAAAT
AC090825.1|NR_135737	TFBS	TFBS	121	128	0.990638987	+	.	TF=HOXD4;class=Homeo domain factors;sequence=CTCATTAA
AC090825.1|NR_135737	TFBS	TFBS	2260	2269	0.990591174	+	.	TF=MNX1;class=Homeo domain factors;sequence=TCTAATTAAA
AC090825.1|NR_135737	TFBS	TFBS	292	299	0.990531244	−	.	TF=OTX1;class=Homeo domain factors;sequence=TTAATCCC
AC090825.1|NR_135737	TFBS	TFBS	1038	1049	0.990326324	−	.	TF=ZNF384;class=C2H2 zinc finger factors;sequence=TCAAAAAAAAAA
AC090825.1|NR_135737	TFBS	TFBS	2261	2268	0.990178233	−	.	TF=EMX1;class=Homeo domain factors;sequence=TTAATTAG
AC090825.1|NR_135737	TFBS	TFBS	2247	2253	0.990162411	+	.	TF=MEIS1;class=Homeo domain factors;sequence=TTGACAA
