# Supplementary material for: Development of a Ferroptosis-Related lncRNA Signature to Predict the Prognosis and Immune Landscape of Bladder Cancer
Source: Dis Markers. 2021 Jun 20;2021:1031906. doi: 10.1155/2021/1031906 (PMC8238626; doi:10.1155/2021/1031906)
Supplement: Supplementary Materials — Supplementary Figure S1: validation of the risk model with resampling. Note: (a) Kaplan-Meier survival analysis of cases in high-risk group and low-risk group (p < 0.001). (b) ROC curves of the risk score in internal validation dataset. Supplementary Figure S2: Kaplan-Meier subgroup survival analysis in TCGA-BLCA patients. Note: (a) age ≤ 64 (p < 0.001). (b) Age > 64 (p < 0.001). (c) Male (p < 0.001). (d) Female (p < 0.001). (e) Pathological T 1-2 stages (p < 0.001). (f) Pathological T 3-4 stages (p < 0.001). (g) N0 stages (p < 0.001). (h) N1-N3 stages (p < 0.001). (i) M0 stage (p < 0.001). (j) M1 stage (p < 0.05). Supplementary Figure S3: functional prediction of AC090825.1 and MAGI2-AS3. Note: (a) the expression difference of AC090825.1 in BCa samples and paracarcinoma tissue via Wilcoxon signed-rank test. (b) The expression difference of MAGI2-AS3 in BCa samples and paracarcinoma tissue via Wilcoxon signed-rank test. (c) The difference values between the expression of AC090825.1 and MAGI2-AS3 in BCa samples and paracarcinoma samples. (d) The cases suffered poorer survival when the expression value of AC090825.1 was higher than that of MAGI2-AS3 (p < 0.01). (e) The expression levels of AC090825.1 and MAGI2-AS3 were positively correlated both in BCa samples and paracarcinoma samples. (f) Network of lncRNAs, the target genes and their possible transcription factors. (g) UpSet diagram showing 6 overlapped transcription factors. (h) The correlation network of lncRNAs, the target genes and transcription factors. BCa: bladder cancer. Supplementary Table S1: 20 ferroptosis regulators were differentially expressed between BCa and paracarinoma samples. Supplementary Table S2: the Pearson correlation analysis of lncRNAs and ferroptosis regulators. Supplementary Table S3: Kaplan-Meier survival analysis of the lncRNA pairs. Supplementary Table S4: enrichment results from LncSEA website. Supplementary Table S5: GSVA results of different clusters based on ferroptosis regulators [file 1031906.f1.zip › Supplementary_Table_S8.PDF]

Supplementary Table S8. The prediction of the transcription factors of MAGI2-AS3.

seqname	source	feature	start	end	score	strand	frame	attributes
MAGI2-AS3|NR_038345	TFBS	TFBS	1067	1072	1	+	.	TF=ZNF354C;class=C2H2 zinc finger factors;sequence=ATCCAC
MAGI2-AS3|NR_038345	TFBS	TFBS	479	486	1	−	.	TF=GATA3;class=Other C4 zinc finger-type factors;sequence=AGATAAGA
MAGI2-AS3|NR_038345	TFBS	TFBS	1396	1403	1	+	.	TF=TFE3;class=Basic helix-loop-helix factors (bHLH);sequence=CACGTGAC
MAGI2-AS3|NR_038345	TFBS	TFBS	358	365	1	+	.	TF=HOXA4;class=Homeo domain factors;sequence=GTCATTAG
MAGI2-AS3|NR_038346	TFBS	TFBS	1067	1072	1	+	.	TF=ZNF354C;class=C2H2 zinc finger factors;sequence=ATCCAC
MAGI2-AS3|NR_038346	TFBS	TFBS	479	486	1	−	.	TF=GATA3;class=Other C4 zinc finger-type factors;sequence=AGATAAGA
MAGI2-AS3|NR_038346	TFBS	TFBS	1396	1403	1	+	.	TF=TFE3;class=Basic helix-loop-helix factors (bHLH);sequence=CACGTGAC
MAGI2-AS3|NR_038346	TFBS	TFBS	358	365	1	+	.	TF=HOXA4;class=Homeo domain factors;sequence=GTCATTAG
MAGI2-AS3|NR_038344	TFBS	TFBS	64	69	1	+	.	TF=ZNF354C;class=C2H2 zinc finger factors;sequence=ATCCAC

MAGI2-AS3|NR_038344	TFBS	TFBS	1115	1122	1	−	.	TF=ARNT::HIF1A;class=Basic helix-loop-helix factors (bHLH);sequence=GGACGTGC
MAGI2-AS3|NR_038344	TFBS	TFBS	393	400	1	+	.	TF=TFE3;class=Basic helix-loop-helix factors (bHLH);sequence=CACGTGAC
MAGI2-AS3|NR_038344	TFBS	TFBS	1443	1452	1	−	.	TF=PLAGL2;class=C2H2 zinc finger factors;sequence=TGGGCCCCCT
MAGI2-AS3|NR_038343	TFBS	TFBS	64	69	1	+	.	TF=ZNF354C;class=C2H2 zinc finger factors;sequence=ATCCAC
MAGI2-AS3|NR_038343	TFBS	TFBS	1115	1122	1	−	.	TF=ARNT::HIF1A;class=Basic helix-loop-helix factors (bHLH);sequence=GGACGTGC
MAGI2-AS3|NR_038343	TFBS	TFBS	393	400	1	+	.	TF=TFE3;class=Basic helix-loop-helix factors (bHLH);sequence=CACGTGAC
MAGI2-AS3|NR_038343	TFBS	TFBS	1443	1452	1	−	.	TF=PLAGL2;class=C2H2 zinc finger factors;sequence=TGGGCCCCCT
MAGI2-AS3|NR_038345	TFBS	TFBS	358	365	0.9996462	+	.	TF=HOXC4;class=Homeo domain factors;sequence=GTCATTAG
MAGI2-AS3|NR_038346	TFBS	TFBS	358	365	0.9996462	+	.	TF=HOXC4;class=Homeo domain factors;sequence=GTCATTAG
MAGI2-AS3|NR_038345	TFBS	TFBS	46	53	0.999565985	−	.	TF=HOXC4;class=Homeo domain

								factors;sequence=A TCATTAA
MAGI2-AS3|NR _038346	TFB S	TFBS	46	53	0.99956 5985	−	.	TF=HOXC4;class=Hom eo domain factors;sequence=A TCATTAA
MAGI2-AS3|NR _038345	TFB S	TFBS	358	36 5	0.99955 7369	+	.	TF=HOXD4;class=Hom eo domain factors;sequence=G TCATTAG
MAGI2-AS3|NR _038346	TFB S	TFBS	358	36 5	0.99955 7369	+	.	TF=HOXD4;class=Hom eo domain factors;sequence=G TCATTAG
MAGI2-AS3|NR _038345	TFB S	TFBS	139 4	14 03	0.99949 5867	−	.	TF=ARNT2;class=Bas ic helix-loop-helix factors (bHLH);sequence=GT CACGTGCG
MAGI2-AS3|NR _038346	TFB S	TFBS	139 4	14 03	0.99949 5867	−	.	TF=ARNT2;class=Bas ic helix-loop-helix factors (bHLH);sequence=GT CACGTGCG
MAGI2-AS3|NR _038344	TFB S	TFBS	391	40 0	0.99949 5867	−	.	TF=ARNT2;class=Bas ic helix-loop-helix factors (bHLH);sequence=GT CACGTGCG
MAGI2-AS3|NR _038343	TFB S	TFBS	391	40 0	0.99949 5867	−	.	TF=ARNT2;class=Bas ic helix-loop-helix factors (bHLH);sequence=GT CACGTGCG
MAGI2-AS3|NR _038345	TFB S	TFBS	46	53	0.99933 1142	−	.	TF=HOXD4;class=Hom eo domain factors;sequence=A TCATTAA
MAGI2-AS3|NR _038346	TFB S	TFBS	46	53	0.99933 1142	−	.	TF=HOXD4;class=Hom eo domain factors;sequence=A TCATTAA
MAGI2-AS3|NR _038345	TFB S	TFBS	225 9	22 69	0.99925 1668	+	.	TF=FOS;class=Basic leucine zipper

								factors (bZIP);sequence=TCTGACTCATT
MAGI2-AS3|NR_038346	TFBS	TFBS	2259	2269	0.999251668	+	.	TF=FOS;class=Basic leucine zipper factors (bZIP);sequence=TCTGACTCATT
MAGI2-AS3|NR_038344	TFBS	TFBS	2259	2269	0.999251668	+	.	TF=FOS;class=Basic leucine zipper factors (bZIP);sequence=TCTGACTCATT
MAGI2-AS3|NR_038345	TFBS	TFBS	358	365	0.999053421	+	.	TF=HOXB4;class=Homeo domain factors;sequence=GTCATTAG
MAGI2-AS3|NR_038345	TFBS	TFBS	46	53	0.999053421	-	.	TF=HOXB4;class=Homeo domain factors;sequence=ATCATTAA
MAGI2-AS3|NR_038346	TFBS	TFBS	358	365	0.999053421	+	.	TF=HOXB4;class=Homeo domain factors;sequence=GTCATTAG
MAGI2-AS3|NR_038346	TFBS	TFBS	46	53	0.999053421	-	.	TF=HOXB4;class=Homeo domain factors;sequence=ATCATTAA
MAGI2-AS3|NR_038345	TFBS	TFBS	683	689	0.996305873	-	.	TF=FOXL1;class=Fork head / winged helix factors;sequence=ATAAACA
MAGI2-AS3|NR_038346	TFBS	TFBS	683	689	0.996305873	-	.	TF=FOXL1;class=Fork head / winged helix factors;sequence=ATAAACA
MAGI2-AS3|NR_038345	TFBS	TFBS	358	365	0.996213057	+	.	TF=GSX2;class=Homeo domain factors;sequence=GTCATTAG
MAGI2-AS3|NR	TFB	TFBS	358	36	0.99621	+	.	TF=GSX2;class=Home

_038346	S			5	3057			o domain factors;sequence=GTCATTAG
MAGI2-AS3|NR_038345	TFBS	TFBS	1875	1884	0.995691748	+	.	TF=KLF5;class=C2H2 zinc finger factors;sequence=GCCCCTCCCC
MAGI2-AS3|NR_038346	TFBS	TFBS	1875	1884	0.995691748	+	.	TF=KLF5;class=C2H2 zinc finger factors;sequence=GCCCCTCCCC
MAGI2-AS3|NR_038344	TFBS	TFBS	872	881	0.995691748	+	.	TF=KLF5;class=C2H2 zinc finger factors;sequence=GCCCCTCCCC
MAGI2-AS3|NR_038343	TFBS	TFBS	872	881	0.995691748	+	.	TF=KLF5;class=C2H2 zinc finger factors;sequence=GCCCCTCCCC
MAGI2-AS3|NR_038345	TFBS	TFBS	1797	1804	0.995237296	+	.	TF=NR2C2(var.2);class=Nuclear receptors with C4 zinc fingers;sequence=GAGGTCAG
MAGI2-AS3|NR_038346	TFBS	TFBS	1797	1804	0.995237296	+	.	TF=NR2C2(var.2);class=Nuclear receptors with C4 zinc fingers;sequence=GAGGTCAG
MAGI2-AS3|NR_038344	TFBS	TFBS	794	801	0.995237296	+	.	TF=NR2C2(var.2);class=Nuclear receptors with C4 zinc fingers;sequence=GAGGTCAG
MAGI2-AS3|NR_038343	TFBS	TFBS	794	801	0.995237296	+	.	TF=NR2C2(var.2);class=Nuclear receptors with C4 zinc fingers;sequence=GAGGTCAG
MAGI2-AS3|NR	TFB	TFBS	236	23	0.99498	-	.	TF=MEIS1;class=Hom

_038345	S		4	70	9704			eo domain factors;sequence=CTGACAG
MAGI2-AS3|NR_038346	TFBS	TFBS	2364	2370	0.994989704	-	.	TF=MEIS1;class=Homeo domain factors;sequence=CTGACAG
MAGI2-AS3|NR_038344	TFBS	TFBS	2364	2370	0.994989704	-	.	TF=MEIS1;class=Homeo domain factors;sequence=CTGACAG
MAGI2-AS3|NR_038345	TFBS	TFBS	786	793	0.994148567	-	.	TF=HOXA4;class=Homeo domain factors;sequence=GTCATTAC
MAGI2-AS3|NR_038346	TFBS	TFBS	786	793	0.994148567	-	.	TF=HOXA4;class=Homeo domain factors;sequence=GTCATTAC
MAGI2-AS3|NR_038343	TFBS	TFBS	2436	2443	0.994069584	+	.	TF=SOX18;class=High-mobility group (HMG) domain factors;sequence=GACAATGC
MAGI2-AS3|NR_038345	TFBS	TFBS	73	78	0.994048003	+	.	TF=ZNF354C;class=C2H2 zinc finger factors;sequence=CTCCAC
MAGI2-AS3|NR_038346	TFBS	TFBS	73	78	0.994048003	+	.	TF=ZNF354C;class=C2H2 zinc finger factors;sequence=CTCCAC
MAGI2-AS3|NR_038345	TFBS	TFBS	2168	2176	0.993536806	+	.	TF=NKX6-3;class=Homeo domain factors;sequence=CCCATTAAA
MAGI2-AS3|NR_038346	TFBS	TFBS	2168	2176	0.993536806	+	.	TF=NKX6-3;class=Homeo domain factors;sequence=CCCATTAAA
MAGI2-AS3|NR_038344	TFBS	TFBS	2168	2176	0.993536806	+	.	TF=NKX6-3;class=Homeo domain factors;sequence=CCCATTAAA

MAGI2-AS3|NR_038345	TFBS	TFBS	855	863	0.993102439	+	.	TF=POU3F4;class=Homeo domain factors;sequence=TATGCTAAT
MAGI2-AS3|NR_038346	TFBS	TFBS	855	863	0.993102439	+	.	TF=POU3F4;class=Homeo domain factors;sequence=TATGCTAAT
MAGI2-AS3|NR_038344	TFBS	TFBS	1813	1822	0.993033649	−	.	TF=BHLHE22(var.2);class=Basic helix-loop-helix factors (bHLH);sequence=TGCAGCTGCC
MAGI2-AS3|NR_038343	TFBS	TFBS	1813	1822	0.993033649	−	.	TF=BHLHE22(var.2);class=Basic helix-loop-helix factors (bHLH);sequence=TGCAGCTGCC
MAGI2-AS3|NR_038344	TFBS	TFBS	1813	1822	0.993020439	+	.	TF=BHLHE22(var.2);class=Basic helix-loop-helix factors (bHLH);sequence=GGCAGCTGCA
MAGI2-AS3|NR_038343	TFBS	TFBS	1813	1822	0.993020439	+	.	TF=BHLHE22(var.2);class=Basic helix-loop-helix factors (bHLH);sequence=GGCAGCTGCA
MAGI2-AS3|NR_038345	TFBS	TFBS	1874	1884	0.992746735	+	.	TF=MAZ;class=C2H2 zinc finger factors;sequence=TGCCCCTCCCC
MAGI2-AS3|NR_038346	TFBS	TFBS	1874	1884	0.992746735	+	.	TF=MAZ;class=C2H2 zinc finger factors;sequence=TGCCCCTCCCC
MAGI2-AS3|NR_038344	TFBS	TFBS	871	881	0.992746735	+	.	TF=MAZ;class=C2H2 zinc finger factors;sequence=TGCCCCTCCCC

MAGI2-AS3|NR_038343	TFBS	TFBS	871	881	0.992746735	+	.	TF=MAZ;class=C2H2 zinc finger factors;sequence=TGCCCCTCCCC
MAGI2-AS3|NR_038345	TFBS	TFBS	1435	1445	0.992484811	-	.	TF=MAZ;class=C2H2 zinc finger factors;sequence=TCCCCCTCCCC
MAGI2-AS3|NR_038346	TFBS	TFBS	1435	1445	0.992484811	-	.	TF=MAZ;class=C2H2 zinc finger factors;sequence=TCCCCCTCCCC
MAGI2-AS3|NR_038344	TFBS	TFBS	432	442	0.992484811	-	.	TF=MAZ;class=C2H2 zinc finger factors;sequence=TCCCCCTCCCC
MAGI2-AS3|NR_038343	TFBS	TFBS	432	442	0.992484811	-	.	TF=MAZ;class=C2H2 zinc finger factors;sequence=TCCCCCTCCCC
MAGI2-AS3|NR_038345	TFBS	TFBS	786	793	0.992250779	-	.	TF=HOXC4;class=Homeo domain factors;sequence=GTCATTAC
MAGI2-AS3|NR_038346	TFBS	TFBS	786	793	0.992250779	-	.	TF=HOXC4;class=Homeo domain factors;sequence=GTCATTAC
MAGI2-AS3|NR_038345	TFBS	TFBS	1394	1403	0.992039415	+	.	TF=BHLHE40;class=Basic helix-loop-helix factors (bHLH);sequence=CGCACGTGAC
MAGI2-AS3|NR_038346	TFBS	TFBS	1394	1403	0.992039415	+	.	TF=BHLHE40;class=Basic helix-loop-helix factors (bHLH);sequence=CGCACGTGAC
MAGI2-AS3|NR_038344	TFBS	TFBS	391	400	0.992039415	+	.	TF=BHLHE40;class=Basic helix-loop-helix factors

								(bHLH);sequence=CGCACGTGAC
MAGI2-AS3|NR_038343	TFBS	TFBS	391	400	0.992039415	+	.	TF=BHLHE40;class=Basic helix-loop-helix factors (bHLH);sequence=CGCACGTGAC
MAGI2-AS3|NR_038343	TFBS	TFBS	2473	2482	0.991904082	+	.	TF=HAND2;class=Basic helix-loop-helix factors (bHLH);sequence=AACAGATGCC
MAGI2-AS3|NR_038345	TFBS	TFBS	2389	2396	0.991814605	+	.	TF=BARX1;class=Homeo domain factors;sequence=ACAATTAA
MAGI2-AS3|NR_038346	TFBS	TFBS	2389	2396	0.991814605	+	.	TF=BARX1;class=Homeo domain factors;sequence=ACAATTAA
MAGI2-AS3|NR_038344	TFBS	TFBS	2389	2396	0.991814605	+	.	TF=BARX1;class=Homeo domain factors;sequence=ACAATTAA
MAGI2-AS3|NR_038345	TFBS	TFBS	46	53	0.991528411	−	.	TF=HOXA4;class=Homeo domain factors;sequence=ATCATTAA
MAGI2-AS3|NR_038346	TFBS	TFBS	46	53	0.991528411	−	.	TF=HOXA4;class=Homeo domain factors;sequence=ATCATTAA
MAGI2-AS3|NR_038345	TFBS	TFBS	786	793	0.99131454	−	.	TF=HOXA7;class=Homeo domain factors;sequence=GTCATTAC
MAGI2-AS3|NR_038346	TFBS	TFBS	786	793	0.99131454	−	.	TF=HOXA7;class=Homeo domain factors;sequence=GTCATTAC
MAGI2-AS3|NR_038345	TFBS	TFBS	786	793	0.990847565	−	.	TF=HOXD8;class=Homeo domain factors;sequence=G

								TCATTAC
MAGI2-AS3|NR_038346	TFBS	TFBS	786	793	0.990847565	-	.	TF=HOXD8;class=Homeo domain factors;sequence=GTCATTAC
MAGI2-AS3|NR_038345	TFBS	TFBS	786	793	0.99075971	-	.	TF=HOXB8;class=Homeo domain factors;sequence=GTCATTAC
MAGI2-AS3|NR_038346	TFBS	TFBS	786	793	0.99075971	-	.	TF=HOXB8;class=Homeo domain factors;sequence=GTCATTAC
MAGI2-AS3|NR_038345	TFBS	TFBS	1394	1404	0.990521718	+	.	TF=TFEC;class=Basic helix-loop-helix factors (bHLH);sequence=CGCACGTGACG
MAGI2-AS3|NR_038346	TFBS	TFBS	1394	1404	0.990521718	+	.	TF=TFEC;class=Basic helix-loop-helix factors (bHLH);sequence=CGCACGTGACG
MAGI2-AS3|NR_038344	TFBS	TFBS	391	401	0.990521718	+	.	TF=TFEC;class=Basic helix-loop-helix factors (bHLH);sequence=CGCACGTGACG
MAGI2-AS3|NR_038343	TFBS	TFBS	391	401	0.990521718	+	.	TF=TFEC;class=Basic helix-loop-helix factors (bHLH);sequence=CGCACGTGACG
MAGI2-AS3|NR_038345	TFBS	TFBS	682	689	0.990192875	-	.	TF=FOXG1;class=Fork head / winged helix factors;sequence=ATAAACAA
MAGI2-AS3|NR_038346	TFBS	TFBS	682	689	0.990192875	-	.	TF=FOXG1;class=Fork head / winged helix factors;sequence=ATAAACAA
