# Supplementary material for: Development of a Ferroptosis-Related lncRNA Signature to Predict the Prognosis and Immune Landscape of Bladder Cancer
Source: Dis Markers. 2021 Jun 20;2021:1031906. doi: 10.1155/2021/1031906 (PMC8238626; doi:10.1155/2021/1031906)
Supplement: Supplementary Materials — Supplementary Figure S1: validation of the risk model with resampling. Note: (a) Kaplan-Meier survival analysis of cases in high-risk group and low-risk group (p < 0.001). (b) ROC curves of the risk score in internal validation dataset. Supplementary Figure S2: Kaplan-Meier subgroup survival analysis in TCGA-BLCA patients. Note: (a) age ≤ 64 (p < 0.001). (b) Age > 64 (p < 0.001). (c) Male (p < 0.001). (d) Female (p < 0.001). (e) Pathological T 1-2 stages (p < 0.001). (f) Pathological T 3-4 stages (p < 0.001). (g) N0 stages (p < 0.001). (h) N1-N3 stages (p < 0.001). (i) M0 stage (p < 0.001). (j) M1 stage (p < 0.05). Supplementary Figure S3: functional prediction of AC090825.1 and MAGI2-AS3. Note: (a) the expression difference of AC090825.1 in BCa samples and paracarcinoma tissue via Wilcoxon signed-rank test. (b) The expression difference of MAGI2-AS3 in BCa samples and paracarcinoma tissue via Wilcoxon signed-rank test. (c) The difference values between the expression of AC090825.1 and MAGI2-AS3 in BCa samples and paracarcinoma samples. (d) The cases suffered poorer survival when the expression value of AC090825.1 was higher than that of MAGI2-AS3 (p < 0.01). (e) The expression levels of AC090825.1 and MAGI2-AS3 were positively correlated both in BCa samples and paracarcinoma samples. (f) Network of lncRNAs, the target genes and their possible transcription factors. (g) UpSet diagram showing 6 overlapped transcription factors. (h) The correlation network of lncRNAs, the target genes and transcription factors. BCa: bladder cancer. Supplementary Table S1: 20 ferroptosis regulators were differentially expressed between BCa and paracarinoma samples. Supplementary Table S2: the Pearson correlation analysis of lncRNAs and ferroptosis regulators. Supplementary Table S3: Kaplan-Meier survival analysis of the lncRNA pairs. Supplementary Table S4: enrichment results from LncSEA website. Supplementary Table S5: GSVA results of different clusters based on ferroptosis regulators [file 1031906.f1.zip › Supplementary_Table_S9.PDF]

Supplementary Table S9. The prediction of the transcription factors of NOX4.

seqname	source	feature	start	end	score	strand	frame	attributes
NOX4|NM_001300995	TFBS	TFBS	1055	1060	1	+	.	TF=ZNF354C;class=C2H2 zinc finger factors;sequence=ATCCAC
NOX4|NM_001300995	TFBS	TFBS	221	226	1	-	.	TF=ZNF354C;class=C2H2 zinc finger factors;sequence=ATCCAC
NOX4|NM_001300995	TFBS	TFBS	746	753	1	+	.	TF=SHOX;class=Homeo domain factors;sequence=TTAATTGG
NOX4|NM_001300995	TFBS	TFBS	1718	1725	1	+	.	TF=SHOX;class=Homeo domain factors;sequence=TTAATTGG
NOX4|NM_001300995	TFBS	TFBS	746	753	1	-	.	TF=MSX2;class=Homeo domain factors;sequence=CCAATTAA
NOX4|NM_001300995	TFBS	TFBS	1718	1725	1	-	.	TF=MSX2;class=Homeo domain factors;sequence=CCAATTAA
NOX4|NM_001300995	TFBS	TFBS	746	753	1	-	.	TF=RAX2;class=Homeo domain factors;sequence=CCAATTAA
NOX4|NM_001300995	TFBS	TFBS	1718	1725	1	-	.	TF=RAX2;class=Homeo domain factors;sequence=CCAATTAA
NOX4|NM_001300995	TFBS	TFBS	2138	2145	1	-	.	TF=MGA;class=T-Box factors;sequence=AGGTGTGA
NOX4|NM_001300995	TFBS	TFBS	2138	2145	1	-	.	TF=TBX15;class=T-Box factors;sequence=AGGTGTGA
NOX4|NM_001300995	TFBS	TFBS	2138	2145	1	-	.	TF=TBX1;class=T-Box factors;sequence=AGGTGTGA

NOX4|NM_001300995	TFBS	TFBS	2138	2145	1	−	.	TF=TBX4;class=T-Box factors;sequence=AGGTGTGA
NOX4|NM_001300995	TFBS	TFBS	2138	2145	1	−	.	TF=TBX5;class=T-Box factors;sequence=AGGTGTGA
NOX4|NM_001300995	TFBS	TFBS	746	753	1	−	.	TF=BSX;class=Homeo domain factors;sequence=CCAATTAA
NOX4|NM_001300995	TFBS	TFBS	1718	1725	1	−	.	TF=BSX;class=Homeo domain factors;sequence=CCAATTAA
NOX4|NM_001300995	TFBS	TFBS	147	154	1	+	.	TF=HOXC4;class=Homeo domain factors;sequence=GTCATTAA
NOX4|NM_001300995	TFBS	TFBS	2294	2309	1	−	.	TF=ZNF460;class=C2H2 zinc finger factors;sequence=GCCTCAGCCTCCCGAG
NOX4|NM_001143837	TFBS	TFBS	198	203	1	+	.	TF=ZNF354C;class=C2H2 zinc finger factors;sequence=ATCCAC
NOX4|NM_001143837	TFBS	TFBS	416	421	1	−	.	TF=ZNF354C;class=C2H2 zinc finger factors;sequence=ATCCAC
NOX4|NM_001143837	TFBS	TFBS	1157	1162	1	−	.	TF=ZNF354C;class=C2H2 zinc finger factors;sequence=ATCCAC
NOX4|NM_001143837	TFBS	TFBS	1173	1179	1	+	.	TF=NFATC2;class=Rel homology region (RHR) factors;sequence=TTTTCCA
NOX4|NM_001143837	TFBS	TFBS	1174	1184	1	+	.	TF=STAT1;class=STAT domain factors;sequence=TTTCCAGGAAA
NOX4|NM_001143837	TFBS	TFBS	509	517	1	+	.	TF=PITX3;class=Homeo domain

								factors;sequence=CTTAATCCC
NOX4|NM_001143837	TFBS	TFBS	1316	1323	1	-	.	TF=VSX1;class=Homeo domain factors;sequence=CTAATTAT
NOX4|NM_001143837	TFBS	TFBS	344	350	1	+	.	TF=MEIS1;class=Homeo domain factors;sequence=TTGACAG
NOX4|NM_001143837	TFBS	TFBS	2138	2145	1	-	.	TF=MGA;class=T-Box factors;sequence=AGGTGTGA
NOX4|NM_001143837	TFBS	TFBS	2138	2145	1	-	.	TF=TBX15;class=T-Box factors;sequence=AGGTGTGA
NOX4|NM_001143837	TFBS	TFBS	2138	2145	1	-	.	TF=TBX1;class=T-Box factors;sequence=AGGTGTGA
NOX4|NM_001143837	TFBS	TFBS	2138	2145	1	-	.	TF=TBX4;class=T-Box factors;sequence=AGGTGTGA
NOX4|NM_001143837	TFBS	TFBS	2138	2145	1	-	.	TF=TBX5;class=T-Box factors;sequence=AGGTGTGA
NOX4|NM_001143837	TFBS	TFBS	736	747	1	+	.	TF=ZNF384;class=C2H2 zinc finger factors;sequence=TTTAAAAAAAAA
NOX4|NM_001143837	TFBS	TFBS	716	724	1	+	.	TF=NKX6-3;class=Homeo domain factors;sequence=CTCATTAAA
NOX4|NM_001143837	TFBS	TFBS	2294	2309	1	-	.	TF=ZNF460;class=C2H2 zinc finger factors;sequence=GCCTCAGCCTCCCGAG
NOX4|NM_001143837	TFBS	TFBS	383	393	1	-	.	TF=PRDM1;class=C2H2 zinc finger factors;sequence=TTCTTTCTCTT
NOX4|NM_001291929	TFBS	TFBS	259	266	1	+	.	TF=SHOX;class=Homeo domain factors;sequence=TTA

								ATTGG
NOX4|NM_001291929	TFBS	TFBS	259	266	1	-	.	TF=MSX2;class=Homeo domain factors;sequence=CCAATTAA
NOX4|NM_001291929	TFBS	TFBS	259	266	1	-	.	TF=RAX2;class=Homeo domain factors;sequence=CCAATTAA
NOX4|NM_001291929	TFBS	TFBS	2138	2145	1	-	.	TF=MGA;class=T-Box factors;sequence=AGGTGTGA
NOX4|NM_001291929	TFBS	TFBS	2138	2145	1	-	.	TF=TBX15;class=T-Box factors;sequence=AGGTGTGA
NOX4|NM_001291929	TFBS	TFBS	2138	2145	1	-	.	TF=TBX1;class=T-Box factors;sequence=AGGTGTGA
NOX4|NM_001291929	TFBS	TFBS	2138	2145	1	-	.	TF=TBX4;class=T-Box factors;sequence=AGGTGTGA
NOX4|NM_001291929	TFBS	TFBS	2138	2145	1	-	.	TF=TBX5;class=T-Box factors;sequence=AGGTGTGA
NOX4|NM_001291929	TFBS	TFBS	259	266	1	-	.	TF=BSX;class=Homeo domain factors;sequence=CCAATTAA
NOX4|NM_001291929	TFBS	TFBS	1919	1926	1	+	.	TF=HOXC4;class=Homeo domain factors;sequence=GTCATTAA
NOX4|NM_001291929	TFBS	TFBS	946	954	1	+	.	TF=NR1I3;class=Nuclear receptors with C4 zinc fingers;sequence=ATGAACTTT
NOX4|NM_001291929	TFBS	TFBS	1816	1823	1	+	.	TF=NR2C2(var.2);class=Nuclear receptors with C4 zinc fingers;sequence=GAGGTCAT
NOX4|NM_001291929	TFBS	TFBS	2294	2309	1	-	.	TF=ZNF460;class=C2H2 zinc finger

								factors;sequence=GCCTCAGCCTCCCGAG
NOX4|NM_001291929	TFBS	TFBS	1870	1879	1	-	.	TF=SOX4;class=High-mobility group (HMG) domain factors;sequence=GAACAAAGGG
NOX4|NM_001291927	TFBS	TFBS	477	484	1	+	.	TF=SHOX;class=Homeo domain factors;sequence=TTAATTGG
NOX4|NM_001291927	TFBS	TFBS	477	484	1	-	.	TF=MSX2;class=Homeo domain factors;sequence=CCAATTAA
NOX4|NM_001291927	TFBS	TFBS	477	484	1	-	.	TF=RAX2;class=Homeo domain factors;sequence=CCAATTAA
NOX4|NM_001291927	TFBS	TFBS	2138	2145	1	-	.	TF=MGA;class=T-Box factors;sequence=AGGTGTGA
NOX4|NM_001291927	TFBS	TFBS	2138	2145	1	-	.	TF=TBX15;class=T-Box factors;sequence=AGGTGTGA
NOX4|NM_001291927	TFBS	TFBS	2138	2145	1	-	.	TF=TBX1;class=T-Box factors;sequence=AGGTGTGA
NOX4|NM_001291927	TFBS	TFBS	2138	2145	1	-	.	TF=TBX4;class=T-Box factors;sequence=AGGTGTGA
NOX4|NM_001291927	TFBS	TFBS	2138	2145	1	-	.	TF=TBX5;class=T-Box factors;sequence=AGGTGTGA
NOX4|NM_001291927	TFBS	TFBS	477	484	1	-	.	TF=BSX;class=Homeo domain factors;sequence=CCAATTAA
NOX4|NM_001291927	TFBS	TFBS	1164	1172	1	+	.	TF=NR1I3;class=Nuclear receptors with C4 zinc fingers;sequence=ATGAACTTT
NOX4|NM_00	TFB	TFBS	229	23	1	-	.	TF=ZNF460;class=C2H2

1291927	S		4	09				zinc finger factors;sequence=GCCTCAGCCTCCCGAG
NOX4|NM_001291926	TFBS	TFBS	211	216	1	+	.	TF=ZNF354C;class=C2H2 zinc finger factors;sequence=ATCCAC
NOX4|NM_001291926	TFBS	TFBS	874	881	1	+	.	TF=SHOX;class=Homeo domain factors;sequence=TTAATTGG
NOX4|NM_001291926	TFBS	TFBS	874	881	1	−	.	TF=MSX2;class=Homeo domain factors;sequence=CCAATTAA
NOX4|NM_001291926	TFBS	TFBS	874	881	1	−	.	TF=RAX2;class=Homeo domain factors;sequence=CCAATTAA
NOX4|NM_001291926	TFBS	TFBS	2138	2145	1	−	.	TF=MGA;class=T-Box factors;sequence=AGGTGTGA
NOX4|NM_001291926	TFBS	TFBS	2138	2145	1	−	.	TF=TBX15;class=T-Box factors;sequence=AGGTGTGA
NOX4|NM_001291926	TFBS	TFBS	2138	2145	1	−	.	TF=TBX1;class=T-Box factors;sequence=AGGTGTGA
NOX4|NM_001291926	TFBS	TFBS	2138	2145	1	−	.	TF=TBX4;class=T-Box factors;sequence=AGGTGTGA
NOX4|NM_001291926	TFBS	TFBS	2138	2145	1	−	.	TF=TBX5;class=T-Box factors;sequence=AGGTGTGA
NOX4|NM_001291926	TFBS	TFBS	874	881	1	−	.	TF=BSX;class=Homeo domain factors;sequence=CCAATTAA
NOX4|NM_001291926	TFBS	TFBS	1561	1569	1	+	.	TF=NR1I3;class=Nuclear receptors with C4 zinc fingers;sequence=ATGAACTTT
NOX4|NM_00	TFB	TFBS	229	23	1	−	.	TF=ZNF460;class=C2H2

1291926	S		4	09				zinc finger factors;sequence=GCCTCAGCCTCCCGAG
NOX4|NM_016931	TFBS	TFBS	211	216	1	+	.	TF=ZNF354C;class=C2H2 zinc finger factors;sequence=ATCCAC
NOX4|NM_016931	TFBS	TFBS	874	881	1	+	.	TF=SHOX;class=Homeo domain factors;sequence=TTAATTGG
NOX4|NM_016931	TFBS	TFBS	874	881	1	-	.	TF=MSX2;class=Homeo domain factors;sequence=CCAATTAA
NOX4|NM_016931	TFBS	TFBS	874	881	1	-	.	TF=RAX2;class=Homeo domain factors;sequence=CCAATTAA
NOX4|NM_016931	TFBS	TFBS	2138	2145	1	-	.	TF=MGA;class=T-Box factors;sequence=AGGTGTGA
NOX4|NM_016931	TFBS	TFBS	2138	2145	1	-	.	TF=TBX15;class=T-Box factors;sequence=AGGTGTGA
NOX4|NM_016931	TFBS	TFBS	2138	2145	1	-	.	TF=TBX1;class=T-Box factors;sequence=AGGTGTGA
NOX4|NM_016931	TFBS	TFBS	2138	2145	1	-	.	TF=TBX4;class=T-Box factors;sequence=AGGTGTGA
NOX4|NM_016931	TFBS	TFBS	2138	2145	1	-	.	TF=TBX5;class=T-Box factors;sequence=AGGTGTGA
NOX4|NM_016931	TFBS	TFBS	874	881	1	-	.	TF=BSX;class=Homeo domain factors;sequence=CCAATTAA
NOX4|NM_016931	TFBS	TFBS	1561	1569	1	+	.	TF=NR1I3;class=Nuclear receptors with C4 zinc fingers;sequence=ATGAACTTT
NOX4|NM_01	TFB	TFBS	229	23	1	-	.	TF=ZNF460;class=C2H2

6931	S		4	09				zinc finger factors;sequence=GCCTCAGCCTCCCGAG
NOX4|NM_001143836	TFBS	TFBS	211	216	1	+	.	TF=ZNF354C;class=C2H2 zinc finger factors;sequence=ATCCAC
NOX4|NM_001143836	TFBS	TFBS	874	881	1	+	.	TF=SHOX;class=Homeo domain factors;sequence=TTAATTGG
NOX4|NM_001143836	TFBS	TFBS	874	881	1	−	.	TF=MSX2;class=Homeo domain factors;sequence=CCAATTAA
NOX4|NM_001143836	TFBS	TFBS	874	881	1	−	.	TF=RAX2;class=Homeo domain factors;sequence=CCAATTAA
NOX4|NM_001143836	TFBS	TFBS	2138	2145	1	−	.	TF=MGA;class=T-Box factors;sequence=AGGTGTGA
NOX4|NM_001143836	TFBS	TFBS	2138	2145	1	−	.	TF=TBX15;class=T-Box factors;sequence=AGGTGTGA
NOX4|NM_001143836	TFBS	TFBS	2138	2145	1	−	.	TF=TBX1;class=T-Box factors;sequence=AGGTGTGA
NOX4|NM_001143836	TFBS	TFBS	2138	2145	1	−	.	TF=TBX4;class=T-Box factors;sequence=AGGTGTGA
NOX4|NM_001143836	TFBS	TFBS	2138	2145	1	−	.	TF=TBX5;class=T-Box factors;sequence=AGGTGTGA
NOX4|NM_001143836	TFBS	TFBS	874	881	1	−	.	TF=BSX;class=Homeo domain factors;sequence=CCAATTAA
NOX4|NM_001143836	TFBS	TFBS	1561	1569	1	+	.	TF=NR1I3;class=Nuclear receptors with C4 zinc fingers;sequence=ATGAACTTT
NOX4|NM_00	TFB	TFBS	229	23	1	−	.	TF=ZNF460;class=C2H2

1143836	S		4	09				zinc finger factors;sequence=GCCTCAGCCTCCCGAG
NOX4|NR_120406	TFBS	TFBS	211	216	1	+	.	TF=ZNF354C;class=C2H2 zinc finger factors;sequence=ATCCAC
NOX4|NR_120406	TFBS	TFBS	2437	2443	1	−	.	TF=NFATC2;class=Rel homology region (RHR) factors;sequence=TTTTCCA
NOX4|NR_120406	TFBS	TFBS	874	881	1	+	.	TF=SHOX;class=Homeo domain factors;sequence=TTAATTGG
NOX4|NR_120406	TFBS	TFBS	874	881	1	−	.	TF=MSX2;class=Homeo domain factors;sequence=CCAATTAA
NOX4|NR_120406	TFBS	TFBS	874	881	1	−	.	TF=RAX2;class=Homeo domain factors;sequence=CCAATTAA
NOX4|NR_120406	TFBS	TFBS	874	881	1	−	.	TF=BSX;class=Homeo domain factors;sequence=CCAATTAA
NOX4|NR_120406	TFBS	TFBS	1561	1569	1	+	.	TF=NR1I3;class=Nuclear receptors with C4 zinc fingers;sequence=ATGAACTTT
NOX4|NM_001300995	TFBS	TFBS	147	155	0.999954273	+	.	TF=NKX6-3;class=Homeo domain factors;sequence=GTCATTAAA
NOX4|NM_001143837	TFBS	TFBS	510	517	0.999852312	+	.	TF=RHOXF1;class=Homeo domain factors;sequence=TTAATCCC
NOX4|NM_001300995	TFBS	TFBS	1697	1704	0.999565985	+	.	TF=HOXC4;class=Homeo domain factors;sequence=ATCATTAA

NOX4|NM_001291929	TFBS	TFBS	238	245	0.999565985	+	.	TF=HOXC4;class=Homeo domain factors;sequence=ATCATTAA
NOX4|NM_001291927	TFBS	TFBS	456	463	0.999565985	+	.	TF=HOXC4;class=Homeo domain factors;sequence=ATCATTAA
NOX4|NM_001291926	TFBS	TFBS	853	860	0.999565985	+	.	TF=HOXC4;class=Homeo domain factors;sequence=ATCATTAA
NOX4|NM_016931	TFBS	TFBS	853	860	0.999565985	+	.	TF=HOXC4;class=Homeo domain factors;sequence=ATCATTAA
NOX4|NM_001143836	TFBS	TFBS	853	860	0.999565985	+	.	TF=HOXC4;class=Homeo domain factors;sequence=ATCATTAA
NOX4|NR_120406	TFBS	TFBS	853	860	0.999565985	+	.	TF=HOXC4;class=Homeo domain factors;sequence=ATCATTAA
NOX4|NM_001300995	TFBS	TFBS	1697	1704	0.999331142	+	.	TF=HOXD4;class=Homeo domain factors;sequence=ATCATTAA
NOX4|NM_001291929	TFBS	TFBS	238	245	0.999331142	+	.	TF=HOXD4;class=Homeo domain factors;sequence=ATCATTAA
NOX4|NM_001291927	TFBS	TFBS	456	463	0.999331142	+	.	TF=HOXD4;class=Homeo domain factors;sequence=ATCATTAA
NOX4|NM_001291926	TFBS	TFBS	853	860	0.999331142	+	.	TF=HOXD4;class=Homeo domain factors;sequence=ATCATTAA
NOX4|NM_016931	TFBS	TFBS	853	860	0.999331142	+	.	TF=HOXD4;class=Homeo domain factors;sequence=ATCATTAA

NOX4|NM_001143836	TFBS	TFBS	853	860	0.999331142	+	.	TF=HOXD4;class=Homeo domain factors;sequence=ATCATTAA
NOX4|NR_120406	TFBS	TFBS	853	860	0.999331142	+	.	TF=HOXD4;class=Homeo domain factors;sequence=ATCATTAA
NOX4|NM_001300995	TFBS	TFBS	1	7	0.999127731	-	.	TF=MEIS1;class=Homeo domain factors;sequence=ATGACAG
NOX4|NM_001300995	TFBS	TFBS	1697	1704	0.999053421	+	.	TF=HOXB4;class=Homeo domain factors;sequence=ATCATTAA
NOX4|NM_001291929	TFBS	TFBS	238	245	0.999053421	+	.	TF=HOXB4;class=Homeo domain factors;sequence=ATCATTAA
NOX4|NM_001291927	TFBS	TFBS	456	463	0.999053421	+	.	TF=HOXB4;class=Homeo domain factors;sequence=ATCATTAA
NOX4|NM_001291926	TFBS	TFBS	853	860	0.999053421	+	.	TF=HOXB4;class=Homeo domain factors;sequence=ATCATTAA
NOX4|NM_016931	TFBS	TFBS	853	860	0.999053421	+	.	TF=HOXB4;class=Homeo domain factors;sequence=ATCATTAA
NOX4|NM_001143836	TFBS	TFBS	853	860	0.999053421	+	.	TF=HOXB4;class=Homeo domain factors;sequence=ATCATTAA
NOX4|NR_120406	TFBS	TFBS	853	860	0.999053421	+	.	TF=HOXB4;class=Homeo domain factors;sequence=ATCATTAA
NOX4|NM_001300995	TFBS	TFBS	746	753	0.998950001	-	.	TF=ISX;class=Homeo domain factors;sequence=CCAATTAA

NOX4|NM_001300995	TFBS	TFBS	1718	1725	0.998950001	-	.	TF=ISX;class=Homeo domain factors;sequence=CCAATTAA
NOX4|NM_001291929	TFBS	TFBS	259	266	0.998950001	-	.	TF=ISX;class=Homeo domain factors;sequence=CCAATTAA
NOX4|NM_001291927	TFBS	TFBS	477	484	0.998950001	-	.	TF=ISX;class=Homeo domain factors;sequence=CCAATTAA
NOX4|NM_001291926	TFBS	TFBS	874	881	0.998950001	-	.	TF=ISX;class=Homeo domain factors;sequence=CCAATTAA
NOX4|NM_016931	TFBS	TFBS	874	881	0.998950001	-	.	TF=ISX;class=Homeo domain factors;sequence=CCAATTAA
NOX4|NM_001143836	TFBS	TFBS	874	881	0.998950001	-	.	TF=ISX;class=Homeo domain factors;sequence=CCAATTAA
NOX4|NR_120406	TFBS	TFBS	874	881	0.998950001	-	.	TF=ISX;class=Homeo domain factors;sequence=CCAATTAA
NOX4|NM_001143837	TFBS	TFBS	1158	1165	0.998946347	-	.	TF=RHOXF1;class=Homeo domain factors;sequence=ATAATCCA
NOX4|NM_001143837	TFBS	TFBS	1350	1357	0.998931566	+	.	TF=NR2C2(var.2);class=Nuclear receptors with C4 zinc fingers;sequence=GAGGTCAC
NOX4|NM_001300995	TFBS	TFBS	147	154	0.998888511	+	.	TF=HOXD4;class=Homeo domain factors;sequence=GTCATTAA
NOX4|NM_001291929	TFBS	TFBS	1919	1926	0.998888511	+	.	TF=HOXD4;class=Homeo domain factors;sequence=GTC

								ATTAA
NOX4|NM_001143837	TFBS	TFBS	510	517	0.998227995	+	.	TF=PITX2;class=Homeo domain factors;sequence=TTAATCCC
NOX4|NM_001143837	TFBS	TFBS	1349	1357	0.998188349	+	.	TF=NR2C1;class=Nuclear receptors with C4 zinc fingers;sequence=AGAGGTCAC
NOX4|NM_001300995	TFBS	TFBS	147	154	0.998106843	+	.	TF=HOXB4;class=Homeo domain factors;sequence=GTCATTAA
NOX4|NM_001291929	TFBS	TFBS	1919	1926	0.998106843	+	.	TF=HOXB4;class=Homeo domain factors;sequence=GTCATTAA
NOX4|NM_001143837	TFBS	TFBS	1852	1859	0.997853614	+	.	TF=TLX2;class=Homeo domain factors;sequence=TCAATTAG
NOX4|NM_001300995	TFBS	TFBS	746	753	0.99735684	−	.	TF=MSX1;class=Homeo domain factors;sequence=CCAATTAA
NOX4|NM_001300995	TFBS	TFBS	1718	1725	0.99735684	−	.	TF=MSX1;class=Homeo domain factors;sequence=CCAATTAA
NOX4|NM_001291929	TFBS	TFBS	259	266	0.99735684	−	.	TF=MSX1;class=Homeo domain factors;sequence=CCAATTAA
NOX4|NM_001291927	TFBS	TFBS	477	484	0.99735684	−	.	TF=MSX1;class=Homeo domain factors;sequence=CCAATTAA
NOX4|NM_001291926	TFBS	TFBS	874	881	0.99735684	−	.	TF=MSX1;class=Homeo domain factors;sequence=CCAATTAA
NOX4|NM_016931	TFBS	TFBS	874	881	0.99735684	−	.	TF=MSX1;class=Homeo domain

								factors;sequence=CCAATTAA
NOX4|NM_001143836	TFBS	TFBS	874	881	0.99735684	–	.	TF=MSX1;class=Homeo domain factors;sequence=CCAATTAA
NOX4|NR_120406	TFBS	TFBS	874	881	0.99735684	–	.	TF=MSX1;class=Homeo domain factors;sequence=CCAATTAA
NOX4|NM_001143837	TFBS	TFBS	1490	1499	0.997278284	+	.	TF=RBPJ;class=Rel homology region (RHR) factors;sequence=CCTGGGAACG
NOX4|NM_001143837	TFBS	TFBS	1316	1323	0.996848455	+	.	TF=HOXD4;class=Homeo domain factors;sequence=ATAATTAG
NOX4|NM_001291929	TFBS	TFBS	1395	1402	0.996558046	–	.	TF=NR2C2(var.2);class=Nuclear receptors with C4 zinc fingers;sequence=AGGGTCAA
NOX4|NM_001291927	TFBS	TFBS	1613	1620	0.996558046	–	.	TF=NR2C2(var.2);class=Nuclear receptors with C4 zinc fingers;sequence=AGGGTCAA
NOX4|NM_001291929	TFBS	TFBS	1912	1924	0.99648563	+	.	TF=CREB1;class=Basic leucine zipper factors (bZIP);sequence=TGGTGATGTCATT
NOX4|NM_001291929	TFBS	TFBS	1815	1823	0.996393595	+	.	TF=NR2C1;class=Nuclear receptors with C4 zinc fingers;sequence=AGAGGTCAT
NOX4|NM_001300995	TFBS	TFBS	620	626	0.996305873	+	.	TF=FOXL1;class=Fork head / winged helix factors;sequence=ATAAACA
NOX4|NM_001300995	TFBS	TFBS	1543	1549	0.996305873	+	.	TF=FOXL1;class=Fork head / winged helix

								factors;sequence=ATAAACA
NOX4|NM_001143837	TFBS	TFBS	289	295	0.996305873	–	.	TF=FOXL1;class=Fork head / winged helix factors;sequence=ATAAACA
NOX4|NM_001291929	TFBS	TFBS	84	90	0.996305873	+	.	TF=FOXL1;class=Fork head / winged helix factors;sequence=ATAAACA
NOX4|NM_001291927	TFBS	TFBS	302	308	0.996305873	+	.	TF=FOXL1;class=Fork head / winged helix factors;sequence=ATAAACA
NOX4|NM_001291926	TFBS	TFBS	699	705	0.996305873	+	.	TF=FOXL1;class=Fork head / winged helix factors;sequence=ATAAACA
NOX4|NM_016931	TFBS	TFBS	699	705	0.996305873	+	.	TF=FOXL1;class=Fork head / winged helix factors;sequence=ATAAACA
NOX4|NM_001143836	TFBS	TFBS	699	705	0.996305873	+	.	TF=FOXL1;class=Fork head / winged helix factors;sequence=ATAAACA
NOX4|NR_120406	TFBS	TFBS	699	705	0.996305873	+	.	TF=FOXL1;class=Fork head / winged helix factors;sequence=ATAAACA
NOX4|NM_001300995	TFBS	TFBS	1778	1785	0.996179597	–	.	TF=HOXD4;class=Homeo domain factors;sequence=ATAATTAA
NOX4|NM_001143837	TFBS	TFBS	1828	1835	0.996179597	–	.	TF=HOXD4;class=Homeo domain factors;sequence=ATAATTAA
NOX4|NM_001291929	TFBS	TFBS	319	326	0.996179597	–	.	TF=HOXD4;class=Homeo domain factors;sequence=ATAATTAA
NOX4|NM_001291927	TFBS	TFBS	537	544	0.996179597	–	.	TF=HOXD4;class=Homeo domain

								factors;sequence=ATAATTAA
NOX4|NM_001291926	TFBS	TFBS	934	941	0.996179597	-	.	TF=HOXD4;class=Homeo domain factors;sequence=ATAATTAA
NOX4|NM_016931	TFBS	TFBS	934	941	0.996179597	-	.	TF=HOXD4;class=Homeo domain factors;sequence=ATAATTAA
NOX4|NM_001143836	TFBS	TFBS	934	941	0.996179597	-	.	TF=HOXD4;class=Homeo domain factors;sequence=ATAATTAA
NOX4|NR_120406	TFBS	TFBS	934	941	0.996179597	-	.	TF=HOXD4;class=Homeo domain factors;sequence=ATAATTAA
NOX4|NM_001300995	TFBS	TFBS	746	753	0.99571388	-	.	TF=PRRX1;class=Homeo domain factors;sequence=CCAATTAA
NOX4|NM_001300995	TFBS	TFBS	1718	1725	0.99571388	-	.	TF=PRRX1;class=Homeo domain factors;sequence=CCAATTAA
NOX4|NM_001291929	TFBS	TFBS	259	266	0.99571388	-	.	TF=PRRX1;class=Homeo domain factors;sequence=CCAATTAA
NOX4|NM_001291927	TFBS	TFBS	477	484	0.99571388	-	.	TF=PRRX1;class=Homeo domain factors;sequence=CCAATTAA
NOX4|NM_001291926	TFBS	TFBS	874	881	0.99571388	-	.	TF=PRRX1;class=Homeo domain factors;sequence=CCAATTAA
NOX4|NM_016931	TFBS	TFBS	874	881	0.99571388	-	.	TF=PRRX1;class=Homeo domain factors;sequence=CCAATTAA
NOX4|NM_001143836	TFBS	TFBS	874	881	0.99571388	-	.	TF=PRRX1;class=Homeo domain

								factors;sequence=CCAATTAA
NOX4|NR_120406	TFBS	TFBS	874	881	0.99571388	-	.	TF=PRRX1;class=Homeo domain factors;sequence=CCAATTAA
NOX4|NM_001300995	TFBS	TFBS	746	753	0.995684226	-	.	TF=DLX6;class=Homeo domain factors;sequence=CCAATTAA
NOX4|NM_001300995	TFBS	TFBS	1718	1725	0.995684226	-	.	TF=DLX6;class=Homeo domain factors;sequence=CCAATTAA
NOX4|NM_001291929	TFBS	TFBS	259	266	0.995684226	-	.	TF=DLX6;class=Homeo domain factors;sequence=CCAATTAA
NOX4|NM_001291927	TFBS	TFBS	477	484	0.995684226	-	.	TF=DLX6;class=Homeo domain factors;sequence=CCAATTAA
NOX4|NM_001291926	TFBS	TFBS	874	881	0.995684226	-	.	TF=DLX6;class=Homeo domain factors;sequence=CCAATTAA
NOX4|NM_016931	TFBS	TFBS	874	881	0.995684226	-	.	TF=DLX6;class=Homeo domain factors;sequence=CCAATTAA
NOX4|NM_001143836	TFBS	TFBS	874	881	0.995684226	-	.	TF=DLX6;class=Homeo domain factors;sequence=CCAATTAA
NOX4|NR_120406	TFBS	TFBS	874	881	0.995684226	-	.	TF=DLX6;class=Homeo domain factors;sequence=CCAATTAA
NOX4|NM_001143837	TFBS	TFBS	1327	1336	0.995411894	+	.	TF=POU6F1(var.2);class=Homeo domain factors;sequence=ATAATGAGGA
NOX4|NM_001300995	TFBS	TFBS	147	154	0.9953249	+	.	TF=HOXA4;class=Homeo domain

								factors;sequence=GTCATTAA
NOX4|NM_001291929	TFBS	TFBS	1919	1926	0.9953249	+	.	TF=HOXA4;class=Homeo domain factors;sequence=GTCATTAA
NOX4|NM_001300995	TFBS	TFBS	692	699	0.995311515	+	.	TF=TEAD3;class=TEA domain factors;sequence=GCATTCCA
NOX4|NM_001300995	TFBS	TFBS	232	239	0.995237296	+	.	TF=NR2C2(var.2);class=Nuclear receptors with C4 zinc fingers;sequence=GAGGTCAG
NOX4|NM_001300995	TFBS	TFBS	2188	2195	0.995237296	+	.	TF=NR2C2(var.2);class=Nuclear receptors with C4 zinc fingers;sequence=GAGGTCAG
NOX4|NM_001143837	TFBS	TFBS	2188	2195	0.995237296	+	.	TF=NR2C2(var.2);class=Nuclear receptors with C4 zinc fingers;sequence=GAGGTCAG
NOX4|NM_001291929	TFBS	TFBS	2188	2195	0.995237296	+	.	TF=NR2C2(var.2);class=Nuclear receptors with C4 zinc fingers;sequence=GAGGTCAG
NOX4|NM_001291927	TFBS	TFBS	2188	2195	0.995237296	+	.	TF=NR2C2(var.2);class=Nuclear receptors with C4 zinc fingers;sequence=GAGGTCAG
NOX4|NM_001291926	TFBS	TFBS	2188	2195	0.995237296	+	.	TF=NR2C2(var.2);class=Nuclear receptors with C4 zinc fingers;sequence=GAGGTCAG
NOX4|NM_016931	TFBS	TFBS	2188	2195	0.995237296	+	.	TF=NR2C2(var.2);class=Nuclear receptors with C4 zinc fingers;sequence=GAG

								GTCAG
NOX4|NM_001143836	TFBS	TFBS	2188	2195	0.995237296	+	.	TF=NR2C2(var.2);class=Nuclear receptors with C4 zinc fingers;sequence=GAGGTCAG
NOX4|NM_001300995	TFBS	TFBS	311	319	0.995113409	-	.	TF=NFIX;class=SMAD/NF-1 DNA-binding domain factors;sequence=TGTGCCAAC
NOX4|NM_001143837	TFBS	TFBS	1566	1572	0.994989704	+	.	TF=MEIS1;class=Homeo domain factors;sequence=CTGACAG
NOX4|NM_001291929	TFBS	TFBS	828	834	0.994989704	-	.	TF=MEIS1;class=Homeo domain factors;sequence=CTGACAG
NOX4|NM_001291927	TFBS	TFBS	1046	1052	0.994989704	-	.	TF=MEIS1;class=Homeo domain factors;sequence=CTGACAG
NOX4|NM_001291926	TFBS	TFBS	1443	1449	0.994989704	-	.	TF=MEIS1;class=Homeo domain factors;sequence=CTGACAG
NOX4|NM_016931	TFBS	TFBS	1443	1449	0.994989704	-	.	TF=MEIS1;class=Homeo domain factors;sequence=CTGACAG
NOX4|NM_001143836	TFBS	TFBS	1443	1449	0.994989704	-	.	TF=MEIS1;class=Homeo domain factors;sequence=CTGACAG
NOX4|NR_120406	TFBS	TFBS	1443	1449	0.994989704	-	.	TF=MEIS1;class=Homeo domain factors;sequence=CTGACAG
NOX4|NM_001143837	TFBS	TFBS	737	748	0.994914257	+	.	TF=ZNF384;class=C2H2 zinc finger factors;sequence=TTAAAAAAAAAA
NOX4|NM_00	TFB	TFBS	738	74	0.99483	+	.	TF=ZNF384;class=C2H2

1143837	S			9	2864			zinc finger factors;sequence=TAAAAAAAAAAA
NOX4|NM_001143837	TFBS	TFBS	485	496	0.994832864	-	.	TF=ZNF384;class=C2H2 zinc finger factors;sequence=TAAAAAAAAAAA
NOX4|NM_001143837	TFBS	TFBS	586	597	0.994832864	-	.	TF=ZNF384;class=C2H2 zinc finger factors;sequence=TAAAAAAAAAAA
NOX4|NM_001143837	TFBS	TFBS	510	517	0.994778353	+	.	TF=PITX1;class=Homeo domain factors;sequence=TTAATCCC
NOX4|NM_001300995	TFBS	TFBS	1778	1785	0.994071307	-	.	TF=HOXC4;class=Homeo domain factors;sequence=ATAATTAA
NOX4|NM_001143837	TFBS	TFBS	1828	1835	0.994071307	-	.	TF=HOXC4;class=Homeo domain factors;sequence=ATAATTAA
NOX4|NM_001291929	TFBS	TFBS	319	326	0.994071307	-	.	TF=HOXC4;class=Homeo domain factors;sequence=ATAATTAA
NOX4|NM_001291927	TFBS	TFBS	537	544	0.994071307	-	.	TF=HOXC4;class=Homeo domain factors;sequence=ATAATTAA
NOX4|NM_001291926	TFBS	TFBS	934	941	0.994071307	-	.	TF=HOXC4;class=Homeo domain factors;sequence=ATAATTAA
NOX4|NM_016931	TFBS	TFBS	934	941	0.994071307	-	.	TF=HOXC4;class=Homeo domain factors;sequence=ATAATTAA
NOX4|NM_001143836	TFBS	TFBS	934	941	0.994071307	-	.	TF=HOXC4;class=Homeo domain factors;sequence=ATAATTAA
NOX4|NR_12	TFB	TFBS	934	94	0.99407	-	.	TF=HOXC4;class=Homeo

<table>
<tr><td>0406</td><td>S</td><td></td><td></td><td>1</td><td>1307</td><td></td><td></td><td>domain factors;sequence=ATAATTAA</td></tr>
<tr><td>NOX4|NM_001300995</td><td>TFBS</td><td>TFBS</td><td>383</td><td>388</td><td>0.994048003</td><td>-</td><td>.</td><td>TF=ZNF354C;class=C2H2 zinc finger factors;sequence=CTCCAC</td></tr>
<tr><td>NOX4|NM_001300995</td><td>TFBS</td><td>TFBS</td><td>1460</td><td>1465</td><td>0.994048003</td><td>-</td><td>.</td><td>TF=ZNF354C;class=C2H2 zinc finger factors;sequence=CTCCAC</td></tr>
<tr><td>NOX4|NM_001291929</td><td>TFBS</td><td>TFBS</td><td>565</td><td>570</td><td>0.994048003</td><td>+</td><td>.</td><td>TF=ZNF354C;class=C2H2 zinc finger factors;sequence=CTCCAC</td></tr>
<tr><td>NOX4|NM_001291929</td><td>TFBS</td><td>TFBS</td><td>1</td><td>6</td><td>0.994048003</td><td>-</td><td>.</td><td>TF=ZNF354C;class=C2H2 zinc finger factors;sequence=CTCCAC</td></tr>
<tr><td>NOX4|NM_001291929</td><td>TFBS</td><td>TFBS</td><td>1800</td><td>1805</td><td>0.994048003</td><td>-</td><td>.</td><td>TF=ZNF354C;class=C2H2 zinc finger factors;sequence=CTCCAC</td></tr>
<tr><td>NOX4|NM_001291927</td><td>TFBS</td><td>TFBS</td><td>783</td><td>788</td><td>0.994048003</td><td>+</td><td>.</td><td>TF=ZNF354C;class=C2H2 zinc finger factors;sequence=CTCCAC</td></tr>
<tr><td>NOX4|NM_001291927</td><td>TFBS</td><td>TFBS</td><td>219</td><td>224</td><td>0.994048003</td><td>-</td><td>.</td><td>TF=ZNF354C;class=C2H2 zinc finger factors;sequence=CTCCAC</td></tr>
<tr><td>NOX4|NM_001291926</td><td>TFBS</td><td>TFBS</td><td>1180</td><td>1185</td><td>0.994048003</td><td>+</td><td>.</td><td>TF=ZNF354C;class=C2H2 zinc finger factors;sequence=CTCCAC</td></tr>
<tr><td>NOX4|NM_001291926</td><td>TFBS</td><td>TFBS</td><td>616</td><td>621</td><td>0.994048003</td><td>-</td><td>.</td><td>TF=ZNF354C;class=C2H2 zinc finger factors;sequence=CTCCAC</td></tr>
<tr><td>NOX4|NM_016931</td><td>TFBS</td><td>TFBS</td><td>1180</td><td>1185</td><td>0.994048003</td><td>+</td><td>.</td><td>TF=ZNF354C;class=C2H2 zinc finger factors;sequence=CTCCAC</td></tr>
<tr><td>NOX4|NM_01</td><td>TFB</td><td>TFBS</td><td>616</td><td>62</td><td>0.99404</td><td>-</td><td>.</td><td>TF=ZNF354C;class=C2H</td></tr>
</table>

6931	S			1	8003			2 zinc finger factors;sequence=CTCCAC
NOX4|NM_001143836	TFBS	TFBS	1180	1185	0.994048003	+	.	TF=ZNF354C;class=C2H2 zinc finger factors;sequence=CTCCAC
NOX4|NM_001143836	TFBS	TFBS	616	621	0.994048003	-	.	TF=ZNF354C;class=C2H2 zinc finger factors;sequence=CTCCAC
NOX4|NR_120406	TFBS	TFBS	1180	1185	0.994048003	+	.	TF=ZNF354C;class=C2H2 zinc finger factors;sequence=CTCCAC
NOX4|NR_120406	TFBS	TFBS	616	621	0.994048003	-	.	TF=ZNF354C;class=C2H2 zinc finger factors;sequence=CTCCAC
NOX4|NM_001300995	TFBS	TFBS	314	323	0.993938771	+	.	TF=MXI1;class=Basic helix-loop-helix factors (bHLH);sequence=GGCACATGCC
NOX4|NM_001143837	TFBS	TFBS	1657	1666	0.993938771	+	.	TF=MXI1;class=Basic helix-loop-helix factors (bHLH);sequence=GGCACATGCC
NOX4|NM_001143837	TFBS	TFBS	1316	1323	0.993833599	+	.	TF=HOXB4;class=Homeo domain factors;sequence=ATAATTAG
NOX4|NM_001143837	TFBS	TFBS	588	599	0.993810188	-	.	TF=ZNF384;class=C2H2 zinc finger factors;sequence=ACTAAAAAAAAA
NOX4|NM_001300995	TFBS	TFBS	190	197	0.993718358	+	.	TF=PITX2;class=Homeo domain factors;sequence=GTAATCCC
NOX4|NM_001300995	TFBS	TFBS	2146	2153	0.993718358	+	.	TF=PITX2;class=Homeo domain factors;sequence=GTA

								ATCCC
NOX4|NM_001300995	TFBS	TFBS	1084	1091	0.993718358	−	.	TF=PITX2;class=Homeo domain factors;sequence=GTAATCCC
NOX4|NM_001143837	TFBS	TFBS	2146	2153	0.993718358	+	.	TF=PITX2;class=Homeo domain factors;sequence=GTAATCCC
NOX4|NM_001291929	TFBS	TFBS	2146	2153	0.993718358	+	.	TF=PITX2;class=Homeo domain factors;sequence=GTAATCCC
NOX4|NM_001291927	TFBS	TFBS	2146	2153	0.993718358	+	.	TF=PITX2;class=Homeo domain factors;sequence=GTAATCCC
NOX4|NM_001291926	TFBS	TFBS	2146	2153	0.993718358	+	.	TF=PITX2;class=Homeo domain factors;sequence=GTAATCCC
NOX4|NM_001291926	TFBS	TFBS	240	247	0.993718358	−	.	TF=PITX2;class=Homeo domain factors;sequence=GTAATCCC
NOX4|NM_016931	TFBS	TFBS	2146	2153	0.993718358	+	.	TF=PITX2;class=Homeo domain factors;sequence=GTAATCCC
NOX4|NM_016931	TFBS	TFBS	240	247	0.993718358	−	.	TF=PITX2;class=Homeo domain factors;sequence=GTAATCCC
NOX4|NM_001143836	TFBS	TFBS	2146	2153	0.993718358	+	.	TF=PITX2;class=Homeo domain factors;sequence=GTAATCCC
NOX4|NM_001143836	TFBS	TFBS	240	247	0.993718358	−	.	TF=PITX2;class=Homeo domain factors;sequence=GTAATCCC
NOX4|NR_120406	TFBS	TFBS	240	247	0.993718358	−	.	TF=PITX2;class=Homeo domain factors;sequence=GTA

								ATCCC
NOX4|NM_001143837	TFBS	TFBS	1316	1323	0.993717508	+	.	TF=HOXC4;class=Homeodomain factors;sequence=ATAATTAG
NOX4|NM_001300995	TFBS	TFBS	536	543	0.993704332	+	.	TF=HOXC4;class=Homeodomain factors;sequence=CTCATTAA
NOX4|NM_001300995	TFBS	TFBS	540	547	0.993704332	−	.	TF=HOXC4;class=Homeodomain factors;sequence=CTCATTAA
NOX4|NM_001143837	TFBS	TFBS	716	723	0.993704332	+	.	TF=HOXC4;class=Homeodomain factors;sequence=CTCATTAA
NOX4|NR_120406	TFBS	TFBS	2325	2332	0.993704332	+	.	TF=HOXC4;class=Homeodomain factors;sequence=CTCATTAA
NOX4|NM_001300995	TFBS	TFBS	1871	1878	0.993350533	+	.	TF=HOXC4;class=Homeodomain factors;sequence=CTCATTAG
NOX4|NM_001291929	TFBS	TFBS	412	419	0.993350533	+	.	TF=HOXC4;class=Homeodomain factors;sequence=CTCATTAG
NOX4|NM_001291927	TFBS	TFBS	630	637	0.993350533	+	.	TF=HOXC4;class=Homeodomain factors;sequence=CTCATTAG
NOX4|NM_001291926	TFBS	TFBS	1027	1034	0.993350533	+	.	TF=HOXC4;class=Homeodomain factors;sequence=CTCATTAG
NOX4|NM_016931	TFBS	TFBS	1027	1034	0.993350533	+	.	TF=HOXC4;class=Homeodomain factors;sequence=CTCATTAG
NOX4|NM_001143836	TFBS	TFBS	1027	1034	0.993350533	+	.	TF=HOXC4;class=Homeodomain factors;sequence=CTC

								ATTAG
NOX4|NR_120406	TFBS	TFBS	1027	1034	0.993350533	+	.	TF=HOXC4;class=Homeo domain factors;sequence=CTCATTAG
NOX4|NM_001300995	TFBS	TFBS	151	159	0.993242305	+	.	TF=SRY;class=High-mobility group (HMG) domain factors;sequence=TTAAACAAT
NOX4|NM_001300995	TFBS	TFBS	2414	2425	0.993230986	+	.	TF=ZNF384;class=C2H2 zinc finger factors;sequence=AAAAAAAAAAAA
NOX4|NM_001300995	TFBS	TFBS	2415	2426	0.993230986	+	.	TF=ZNF384;class=C2H2 zinc finger factors;sequence=AAAAAAAAAAAA
NOX4|NM_001300995	TFBS	TFBS	2416	2427	0.993230986	+	.	TF=ZNF384;class=C2H2 zinc finger factors;sequence=AAAAAAAAAAAA
NOX4|NM_001300995	TFBS	TFBS	2417	2428	0.993230986	+	.	TF=ZNF384;class=C2H2 zinc finger factors;sequence=AAAAAAAAAAAA
NOX4|NM_001300995	TFBS	TFBS	2418	2429	0.993230986	+	.	TF=ZNF384;class=C2H2 zinc finger factors;sequence=AAAAAAAAAAAA
NOX4|NM_001300995	TFBS	TFBS	2419	2430	0.993230986	+	.	TF=ZNF384;class=C2H2 zinc finger factors;sequence=AAAAAAAAAAAA
NOX4|NM_001300995	TFBS	TFBS	2420	2431	0.993230986	+	.	TF=ZNF384;class=C2H2 zinc finger factors;sequence=AAAAAAAAAAAA
NOX4|NM_001300995	TFBS	TFBS	2421	2432	0.993230986	+	.	TF=ZNF384;class=C2H2 zinc finger factors;sequence=AAAAAAAAAAAA
NOX4|NM_001300995	TFBS	TFBS	2422	2433	0.993230986	+	.	TF=ZNF384;class=C2H2 zinc finger

								factors;sequence=AAAAAAAAAAAA
NOX4|NM_001300995	TFBS	TFBS	2423	2434	0.993230986	+	.	TF=ZNF384;class=C2H2 zinc finger factors;sequence=AAAAAAAAAAAA
NOX4|NM_001300995	TFBS	TFBS	2424	2435	0.993230986	+	.	TF=ZNF384;class=C2H2 zinc finger factors;sequence=AAAAAAAAAAAA
NOX4|NM_001300995	TFBS	TFBS	2425	2436	0.993230986	+	.	TF=ZNF384;class=C2H2 zinc finger factors;sequence=AAAAAAAAAAAA
NOX4|NM_001300995	TFBS	TFBS	2426	2437	0.993230986	+	.	TF=ZNF384;class=C2H2 zinc finger factors;sequence=AAAAAAAAAAAA
NOX4|NM_001300995	TFBS	TFBS	2427	2438	0.993230986	+	.	TF=ZNF384;class=C2H2 zinc finger factors;sequence=AAAAAAAAAAAA
NOX4|NM_001300995	TFBS	TFBS	820	831	0.993230986	−	.	TF=ZNF384;class=C2H2 zinc finger factors;sequence=AAAAAAAAAAAA
NOX4|NM_001143837	TFBS	TFBS	739	750	0.993230986	+	.	TF=ZNF384;class=C2H2 zinc finger factors;sequence=AAAAAAAAAAAA
NOX4|NM_001143837	TFBS	TFBS	2414	2425	0.993230986	+	.	TF=ZNF384;class=C2H2 zinc finger factors;sequence=AAAAAAAAAAAA
NOX4|NM_001143837	TFBS	TFBS	2415	2426	0.993230986	+	.	TF=ZNF384;class=C2H2 zinc finger factors;sequence=AAAAAAAAAAAA
NOX4|NM_001143837	TFBS	TFBS	2416	2427	0.993230986	+	.	TF=ZNF384;class=C2H2 zinc finger factors;sequence=AAAAAAAAAAAA
NOX4|NM_001143837	TFBS	TFBS	2417	2428	0.993230986	+	.	TF=ZNF384;class=C2H2 zinc finger

								factors;sequence=AAAAAAAAAAAA
NOX4|NM_001143837	TFBS	TFBS	2418	2429	0.993230986	+	.	TF=ZNF384;class=C2H2 zinc finger factors;sequence=AAAAAAAAAAAA
NOX4|NM_001143837	TFBS	TFBS	2419	2430	0.993230986	+	.	TF=ZNF384;class=C2H2 zinc finger factors;sequence=AAAAAAAAAAAA
NOX4|NM_001143837	TFBS	TFBS	2420	2431	0.993230986	+	.	TF=ZNF384;class=C2H2 zinc finger factors;sequence=AAAAAAAAAAAA
NOX4|NM_001143837	TFBS	TFBS	2421	2432	0.993230986	+	.	TF=ZNF384;class=C2H2 zinc finger factors;sequence=AAAAAAAAAAAA
NOX4|NM_001143837	TFBS	TFBS	2422	2433	0.993230986	+	.	TF=ZNF384;class=C2H2 zinc finger factors;sequence=AAAAAAAAAAAA
NOX4|NM_001143837	TFBS	TFBS	2423	2434	0.993230986	+	.	TF=ZNF384;class=C2H2 zinc finger factors;sequence=AAAAAAAAAAAA
NOX4|NM_001143837	TFBS	TFBS	2424	2435	0.993230986	+	.	TF=ZNF384;class=C2H2 zinc finger factors;sequence=AAAAAAAAAAAA
NOX4|NM_001143837	TFBS	TFBS	2425	2436	0.993230986	+	.	TF=ZNF384;class=C2H2 zinc finger factors;sequence=AAAAAAAAAAAA
NOX4|NM_001143837	TFBS	TFBS	2426	2437	0.993230986	+	.	TF=ZNF384;class=C2H2 zinc finger factors;sequence=AAAAAAAAAAAA
NOX4|NM_001143837	TFBS	TFBS	2427	2438	0.993230986	+	.	TF=ZNF384;class=C2H2 zinc finger factors;sequence=AAAAAAAAAAAA
NOX4|NM_001143837	TFBS	TFBS	484	495	0.993230986	-	.	TF=ZNF384;class=C2H2 zinc finger

								factors;sequence=AAAAAAAAAAAA
NOX4|NM_001143837	TFBS	TFBS	575	586	0.993230986	–	.	TF=ZNF384;class=C2H2 zinc finger factors;sequence=AAAAAAAAAAAA
NOX4|NM_001143837	TFBS	TFBS	576	587	0.993230986	–	.	TF=ZNF384;class=C2H2 zinc finger factors;sequence=AAAAAAAAAAAA
NOX4|NM_001143837	TFBS	TFBS	577	588	0.993230986	–	.	TF=ZNF384;class=C2H2 zinc finger factors;sequence=AAAAAAAAAAAA
NOX4|NM_001143837	TFBS	TFBS	578	589	0.993230986	–	.	TF=ZNF384;class=C2H2 zinc finger factors;sequence=AAAAAAAAAAAA
NOX4|NM_001143837	TFBS	TFBS	579	590	0.993230986	–	.	TF=ZNF384;class=C2H2 zinc finger factors;sequence=AAAAAAAAAAAA
NOX4|NM_001143837	TFBS	TFBS	580	591	0.993230986	–	.	TF=ZNF384;class=C2H2 zinc finger factors;sequence=AAAAAAAAAAAA
NOX4|NM_001143837	TFBS	TFBS	581	592	0.993230986	–	.	TF=ZNF384;class=C2H2 zinc finger factors;sequence=AAAAAAAAAAAA
NOX4|NM_001143837	TFBS	TFBS	582	593	0.993230986	–	.	TF=ZNF384;class=C2H2 zinc finger factors;sequence=AAAAAAAAAAAA
NOX4|NM_001143837	TFBS	TFBS	583	594	0.993230986	–	.	TF=ZNF384;class=C2H2 zinc finger factors;sequence=AAAAAAAAAAAA
NOX4|NM_001143837	TFBS	TFBS	584	595	0.993230986	–	.	TF=ZNF384;class=C2H2 zinc finger factors;sequence=AAAAAAAAAAAA
NOX4|NM_001143837	TFBS	TFBS	585	596	0.993230986	–	.	TF=ZNF384;class=C2H2 zinc finger

								factors;sequence=AAAAAAAAAAAA
NOX4|NM_001291929	TFBS	TFBS	2414	2425	0.993230986	+	.	TF=ZNF384;class=C2H2 zinc finger factors;sequence=AAAAAAAAAAAA
NOX4|NM_001291929	TFBS	TFBS	2415	2426	0.993230986	+	.	TF=ZNF384;class=C2H2 zinc finger factors;sequence=AAAAAAAAAAAA
NOX4|NM_001291929	TFBS	TFBS	2416	2427	0.993230986	+	.	TF=ZNF384;class=C2H2 zinc finger factors;sequence=AAAAAAAAAAAA
NOX4|NM_001291929	TFBS	TFBS	2417	2428	0.993230986	+	.	TF=ZNF384;class=C2H2 zinc finger factors;sequence=AAAAAAAAAAAA
NOX4|NM_001291929	TFBS	TFBS	2418	2429	0.993230986	+	.	TF=ZNF384;class=C2H2 zinc finger factors;sequence=AAAAAAAAAAAA
NOX4|NM_001291929	TFBS	TFBS	2419	2430	0.993230986	+	.	TF=ZNF384;class=C2H2 zinc finger factors;sequence=AAAAAAAAAAAA
NOX4|NM_001291929	TFBS	TFBS	2420	2431	0.993230986	+	.	TF=ZNF384;class=C2H2 zinc finger factors;sequence=AAAAAAAAAAAA
NOX4|NM_001291929	TFBS	TFBS	2421	2432	0.993230986	+	.	TF=ZNF384;class=C2H2 zinc finger factors;sequence=AAAAAAAAAAAA
NOX4|NM_001291929	TFBS	TFBS	2422	2433	0.993230986	+	.	TF=ZNF384;class=C2H2 zinc finger factors;sequence=AAAAAAAAAAAA
NOX4|NM_001291929	TFBS	TFBS	2423	2434	0.993230986	+	.	TF=ZNF384;class=C2H2 zinc finger factors;sequence=AAAAAAAAAAAA
NOX4|NM_001291929	TFBS	TFBS	2424	2435	0.993230986	+	.	TF=ZNF384;class=C2H2 zinc finger

								factors;sequence=AAAAAAAAAAAA
NOX4|NM_001291929	TFBS	TFBS	2425	2436	0.993230986	+	.	TF=ZNF384;class=C2H2 zinc finger factors;sequence=AAAAAAAAAAAA
NOX4|NM_001291929	TFBS	TFBS	2426	2437	0.993230986	+	.	TF=ZNF384;class=C2H2 zinc finger factors;sequence=AAAAAAAAAAAA
NOX4|NM_001291929	TFBS	TFBS	2427	2438	0.993230986	+	.	TF=ZNF384;class=C2H2 zinc finger factors;sequence=AAAAAAAAAAAA
NOX4|NM_001291927	TFBS	TFBS	2414	2425	0.993230986	+	.	TF=ZNF384;class=C2H2 zinc finger factors;sequence=AAAAAAAAAAAA
NOX4|NM_001291927	TFBS	TFBS	2415	2426	0.993230986	+	.	TF=ZNF384;class=C2H2 zinc finger factors;sequence=AAAAAAAAAAAA
NOX4|NM_001291927	TFBS	TFBS	2416	2427	0.993230986	+	.	TF=ZNF384;class=C2H2 zinc finger factors;sequence=AAAAAAAAAAAA
NOX4|NM_001291927	TFBS	TFBS	2417	2428	0.993230986	+	.	TF=ZNF384;class=C2H2 zinc finger factors;sequence=AAAAAAAAAAAA
NOX4|NM_001291927	TFBS	TFBS	2418	2429	0.993230986	+	.	TF=ZNF384;class=C2H2 zinc finger factors;sequence=AAAAAAAAAAAA
NOX4|NM_001291927	TFBS	TFBS	2419	2430	0.993230986	+	.	TF=ZNF384;class=C2H2 zinc finger factors;sequence=AAAAAAAAAAAA
NOX4|NM_001291927	TFBS	TFBS	2420	2431	0.993230986	+	.	TF=ZNF384;class=C2H2 zinc finger factors;sequence=AAAAAAAAAAAA
NOX4|NM_001291927	TFBS	TFBS	2421	2432	0.993230986	+	.	TF=ZNF384;class=C2H2 zinc finger

								factors;sequence=AAAAAAAAAAAA
NOX4|NM_001291927	TFBS	TFBS	2422	2433	0.993230986	+	.	TF=ZNF384;class=C2H2 zinc finger factors;sequence=AAAAAAAAAAAA
NOX4|NM_001291927	TFBS	TFBS	2423	2434	0.993230986	+	.	TF=ZNF384;class=C2H2 zinc finger factors;sequence=AAAAAAAAAAAA
NOX4|NM_001291927	TFBS	TFBS	2424	2435	0.993230986	+	.	TF=ZNF384;class=C2H2 zinc finger factors;sequence=AAAAAAAAAAAA
NOX4|NM_001291927	TFBS	TFBS	2425	2436	0.993230986	+	.	TF=ZNF384;class=C2H2 zinc finger factors;sequence=AAAAAAAAAAAA
NOX4|NM_001291927	TFBS	TFBS	2426	2437	0.993230986	+	.	TF=ZNF384;class=C2H2 zinc finger factors;sequence=AAAAAAAAAAAA
NOX4|NM_001291927	TFBS	TFBS	2427	2438	0.993230986	+	.	TF=ZNF384;class=C2H2 zinc finger factors;sequence=AAAAAAAAAAAA
NOX4|NM_001291926	TFBS	TFBS	2414	2425	0.993230986	+	.	TF=ZNF384;class=C2H2 zinc finger factors;sequence=AAAAAAAAAAAA
NOX4|NM_001291926	TFBS	TFBS	2415	2426	0.993230986	+	.	TF=ZNF384;class=C2H2 zinc finger factors;sequence=AAAAAAAAAAAA
NOX4|NM_001291926	TFBS	TFBS	2416	2427	0.993230986	+	.	TF=ZNF384;class=C2H2 zinc finger factors;sequence=AAAAAAAAAAAA
NOX4|NM_001291926	TFBS	TFBS	2417	2428	0.993230986	+	.	TF=ZNF384;class=C2H2 zinc finger factors;sequence=AAAAAAAAAAAA
NOX4|NM_001291926	TFBS	TFBS	2418	2429	0.993230986	+	.	TF=ZNF384;class=C2H2 zinc finger

								factors;sequence=AAAAAAAAAAAA
NOX4|NM_001291926	TFBS	TFBS	2419	2430	0.993230986	+	.	TF=ZNF384;class=C2H2 zinc finger factors;sequence=AAAAAAAAAAAA
NOX4|NM_001291926	TFBS	TFBS	2420	2431	0.993230986	+	.	TF=ZNF384;class=C2H2 zinc finger factors;sequence=AAAAAAAAAAAA
NOX4|NM_001291926	TFBS	TFBS	2421	2432	0.993230986	+	.	TF=ZNF384;class=C2H2 zinc finger factors;sequence=AAAAAAAAAAAA
NOX4|NM_001291926	TFBS	TFBS	2422	2433	0.993230986	+	.	TF=ZNF384;class=C2H2 zinc finger factors;sequence=AAAAAAAAAAAA
NOX4|NM_001291926	TFBS	TFBS	2423	2434	0.993230986	+	.	TF=ZNF384;class=C2H2 zinc finger factors;sequence=AAAAAAAAAAAA
NOX4|NM_001291926	TFBS	TFBS	2424	2435	0.993230986	+	.	TF=ZNF384;class=C2H2 zinc finger factors;sequence=AAAAAAAAAAAA
NOX4|NM_001291926	TFBS	TFBS	2425	2436	0.993230986	+	.	TF=ZNF384;class=C2H2 zinc finger factors;sequence=AAAAAAAAAAAA
NOX4|NM_001291926	TFBS	TFBS	2426	2437	0.993230986	+	.	TF=ZNF384;class=C2H2 zinc finger factors;sequence=AAAAAAAAAAAA
NOX4|NM_001291926	TFBS	TFBS	2427	2438	0.993230986	+	.	TF=ZNF384;class=C2H2 zinc finger factors;sequence=AAAAAAAAAAAA
NOX4|NM_016931	TFBS	TFBS	2414	2425	0.993230986	+	.	TF=ZNF384;class=C2H2 zinc finger factors;sequence=AAAAAAAAAAAA
NOX4|NM_016931	TFBS	TFBS	2415	2426	0.993230986	+	.	TF=ZNF384;class=C2H2 zinc finger

								factors;sequence=AAAAAAAAAAAA
NOX4|NM_016931	TFBS	TFBS	2416	2427	0.993230986	+	.	TF=ZNF384;class=C2H2 zinc finger factors;sequence=AAAAAAAAAAAA
NOX4|NM_016931	TFBS	TFBS	2417	2428	0.993230986	+	.	TF=ZNF384;class=C2H2 zinc finger factors;sequence=AAAAAAAAAAAA
NOX4|NM_016931	TFBS	TFBS	2418	2429	0.993230986	+	.	TF=ZNF384;class=C2H2 zinc finger factors;sequence=AAAAAAAAAAAA
NOX4|NM_016931	TFBS	TFBS	2419	2430	0.993230986	+	.	TF=ZNF384;class=C2H2 zinc finger factors;sequence=AAAAAAAAAAAA
NOX4|NM_016931	TFBS	TFBS	2420	2431	0.993230986	+	.	TF=ZNF384;class=C2H2 zinc finger factors;sequence=AAAAAAAAAAAA
NOX4|NM_016931	TFBS	TFBS	2421	2432	0.993230986	+	.	TF=ZNF384;class=C2H2 zinc finger factors;sequence=AAAAAAAAAAAA
NOX4|NM_016931	TFBS	TFBS	2422	2433	0.993230986	+	.	TF=ZNF384;class=C2H2 zinc finger factors;sequence=AAAAAAAAAAAA
NOX4|NM_016931	TFBS	TFBS	2423	2434	0.993230986	+	.	TF=ZNF384;class=C2H2 zinc finger factors;sequence=AAAAAAAAAAAA
NOX4|NM_016931	TFBS	TFBS	2424	2435	0.993230986	+	.	TF=ZNF384;class=C2H2 zinc finger factors;sequence=AAAAAAAAAAAA
NOX4|NM_016931	TFBS	TFBS	2425	2436	0.993230986	+	.	TF=ZNF384;class=C2H2 zinc finger factors;sequence=AAAAAAAAAAAA
NOX4|NM_016931	TFBS	TFBS	2426	2437	0.993230986	+	.	TF=ZNF384;class=C2H2 zinc finger

								factors;sequence=AAAAAAAAAAAA
NOX4|NM_016931	TFBS	TFBS	2427	2438	0.993230986	+	.	TF=ZNF384;class=C2H2 zinc finger factors;sequence=AAAAAAAAAAAA
NOX4|NM_001143836	TFBS	TFBS	2414	2425	0.993230986	+	.	TF=ZNF384;class=C2H2 zinc finger factors;sequence=AAAAAAAAAAAA
NOX4|NM_001143836	TFBS	TFBS	2415	2426	0.993230986	+	.	TF=ZNF384;class=C2H2 zinc finger factors;sequence=AAAAAAAAAAAA
NOX4|NM_001143836	TFBS	TFBS	2416	2427	0.993230986	+	.	TF=ZNF384;class=C2H2 zinc finger factors;sequence=AAAAAAAAAAAA
NOX4|NM_001143836	TFBS	TFBS	2417	2428	0.993230986	+	.	TF=ZNF384;class=C2H2 zinc finger factors;sequence=AAAAAAAAAAAA
NOX4|NM_001143836	TFBS	TFBS	2418	2429	0.993230986	+	.	TF=ZNF384;class=C2H2 zinc finger factors;sequence=AAAAAAAAAAAA
NOX4|NM_001143836	TFBS	TFBS	2419	2430	0.993230986	+	.	TF=ZNF384;class=C2H2 zinc finger factors;sequence=AAAAAAAAAAAA
NOX4|NM_001143836	TFBS	TFBS	2420	2431	0.993230986	+	.	TF=ZNF384;class=C2H2 zinc finger factors;sequence=AAAAAAAAAAAA
NOX4|NM_001143836	TFBS	TFBS	2421	2432	0.993230986	+	.	TF=ZNF384;class=C2H2 zinc finger factors;sequence=AAAAAAAAAAAA
NOX4|NM_001143836	TFBS	TFBS	2422	2433	0.993230986	+	.	TF=ZNF384;class=C2H2 zinc finger factors;sequence=AAAAAAAAAAAA
NOX4|NM_001143836	TFBS	TFBS	2423	2434	0.993230986	+	.	TF=ZNF384;class=C2H2 zinc finger

								factors;sequence=AAAAAAAAAAAA
NOX4|NM_001143836	TFBS	TFBS	2424	2435	0.993230986	+	.	TF=ZNF384;class=C2H2 zinc finger factors;sequence=AAAAAAAAAAAA
NOX4|NM_001143836	TFBS	TFBS	2425	2436	0.993230986	+	.	TF=ZNF384;class=C2H2 zinc finger factors;sequence=AAAAAAAAAAAA
NOX4|NM_001143836	TFBS	TFBS	2426	2437	0.993230986	+	.	TF=ZNF384;class=C2H2 zinc finger factors;sequence=AAAAAAAAAAAA
NOX4|NM_001143836	TFBS	TFBS	2427	2438	0.993230986	+	.	TF=ZNF384;class=C2H2 zinc finger factors;sequence=AAAAAAAAAAAA
NOX4|NM_001300995	TFBS	TFBS	1871	1878	0.993171445	+	.	TF=GSX2;class=Homeo domain factors;sequence=CTCATTAG
NOX4|NM_001291929	TFBS	TFBS	412	419	0.993171445	+	.	TF=GSX2;class=Homeo domain factors;sequence=CTCATTAG
NOX4|NM_001291927	TFBS	TFBS	630	637	0.993171445	+	.	TF=GSX2;class=Homeo domain factors;sequence=CTCATTAG
NOX4|NM_001291926	TFBS	TFBS	1027	1034	0.993171445	+	.	TF=GSX2;class=Homeo domain factors;sequence=CTCATTAG
NOX4|NM_016931	TFBS	TFBS	1027	1034	0.993171445	+	.	TF=GSX2;class=Homeo domain factors;sequence=CTCATTAG
NOX4|NM_001143836	TFBS	TFBS	1027	1034	0.993171445	+	.	TF=GSX2;class=Homeo domain factors;sequence=CTCATTAG
NOX4|NR_120406	TFBS	TFBS	1027	1034	0.993171445	+	.	TF=GSX2;class=Homeo domain

								factors;sequence=CTCATTAG
NOX4|NM_001300995	TFBS	TFBS	147	154	0.992897745	-	.	TF=HOXB6;class=Homeo domain factors;sequence=TTAATGAC
NOX4|NM_001291929	TFBS	TFBS	1919	1926	0.992897745	-	.	TF=HOXB6;class=Homeo domain factors;sequence=TTAATGAC
NOX4|NM_001300995	TFBS	TFBS	1778	1785	0.992887021	-	.	TF=HOXB4;class=Homeo domain factors;sequence=ATAATTAA
NOX4|NM_001143837	TFBS	TFBS	1828	1835	0.992887021	-	.	TF=HOXB4;class=Homeo domain factors;sequence=ATAATTAA
NOX4|NM_001291929	TFBS	TFBS	319	326	0.992887021	-	.	TF=HOXB4;class=Homeo domain factors;sequence=ATAATTAA
NOX4|NM_001291927	TFBS	TFBS	537	544	0.992887021	-	.	TF=HOXB4;class=Homeo domain factors;sequence=ATAATTAA
NOX4|NM_001291926	TFBS	TFBS	934	941	0.992887021	-	.	TF=HOXB4;class=Homeo domain factors;sequence=ATAATTAA
NOX4|NM_016931	TFBS	TFBS	934	941	0.992887021	-	.	TF=HOXB4;class=Homeo domain factors;sequence=ATAATTAA
NOX4|NM_001143836	TFBS	TFBS	934	941	0.992887021	-	.	TF=HOXB4;class=Homeo domain factors;sequence=ATAATTAA
NOX4|NR_120406	TFBS	TFBS	934	941	0.992887021	-	.	TF=HOXB4;class=Homeo domain factors;sequence=ATAATTAA
NOX4|NM_001143837	TFBS	TFBS	1316	1323	0.992795264	-	.	TF=DRGX;class=Homeo domain

								factors;sequence=CTAATTAT
NOX4|NM_001300995	TFBS	TFBS	2441	2450	0.992781701	-	.	TF=NFIA;class=SMAD/NF-1 DNA-binding domain factors;sequence=AGTGCCAATT
NOX4|NM_001143837	TFBS	TFBS	2441	2450	0.992781701	-	.	TF=NFIA;class=SMAD/NF-1 DNA-binding domain factors;sequence=AGTGCCAATT
NOX4|NM_001291929	TFBS	TFBS	2441	2450	0.992781701	-	.	TF=NFIA;class=SMAD/NF-1 DNA-binding domain factors;sequence=AGTGCCAATT
NOX4|NM_001291927	TFBS	TFBS	2441	2450	0.992781701	-	.	TF=NFIA;class=SMAD/NF-1 DNA-binding domain factors;sequence=AGTGCCAATT
NOX4|NM_001291926	TFBS	TFBS	2441	2450	0.992781701	-	.	TF=NFIA;class=SMAD/NF-1 DNA-binding domain factors;sequence=AGTGCCAATT
NOX4|NM_016931	TFBS	TFBS	2441	2450	0.992781701	-	.	TF=NFIA;class=SMAD/NF-1 DNA-binding domain factors;sequence=AGTGCCAATT
NOX4|NM_001143836	TFBS	TFBS	2441	2450	0.992781701	-	.	TF=NFIA;class=SMAD/NF-1 DNA-binding domain factors;sequence=AGTGCCAATT
NOX4|NM_001300995	TFBS	TFBS	746	753	0.99252567	-	.	TF=UNCX;class=Homeo domain factors;sequence=CCAATTAA
NOX4|NM_001300995	TFBS	TFBS	1718	1725	0.99252567	-	.	TF=UNCX;class=Homeo domain factors;sequence=CCA

								ATTAA
NOX4|NM_001291929	TFBS	TFBS	259	266	0.99252567	-	.	TF=UNCX;class=Homeo domain factors;sequence=CCAATTAA
NOX4|NM_001291927	TFBS	TFBS	477	484	0.99252567	-	.	TF=UNCX;class=Homeo domain factors;sequence=CCAATTAA
NOX4|NM_001291926	TFBS	TFBS	874	881	0.99252567	-	.	TF=UNCX;class=Homeo domain factors;sequence=CCAATTAA
NOX4|NM_016931	TFBS	TFBS	874	881	0.99252567	-	.	TF=UNCX;class=Homeo domain factors;sequence=CCAATTAA
NOX4|NM_001143836	TFBS	TFBS	874	881	0.99252567	-	.	TF=UNCX;class=Homeo domain factors;sequence=CCAATTAA
NOX4|NR_120406	TFBS	TFBS	874	881	0.99252567	-	.	TF=UNCX;class=Homeo domain factors;sequence=CCAATTAA
NOX4|NM_001143837	TFBS	TFBS	509	518	0.991907541	+	.	TF=GSC2;class=Homeo domain factors;sequence=CTTAATCCCC
NOX4|NM_001291929	TFBS	TFBS	1073	1080	0.991863024	-	.	TF=SOX18;class=High-mobility group (HMG) domain factors;sequence=AACAATGC
NOX4|NM_001291927	TFBS	TFBS	1291	1298	0.991863024	-	.	TF=SOX18;class=High-mobility group (HMG) domain factors;sequence=AACAATGC
NOX4|NM_001291926	TFBS	TFBS	1688	1695	0.991863024	-	.	TF=SOX18;class=High-mobility group (HMG) domain factors;sequence=AACAATGC

NOX4|NM_016931	TFBS	TFBS	1688	1695	0.991863024	-	.	TF=SOX18;class=High-mobility group (HMG) domain factors;sequence=AACAATGC
NOX4|NM_001143836	TFBS	TFBS	1688	1695	0.991863024	-	.	TF=SOX18;class=High-mobility group (HMG) domain factors;sequence=AACAATGC
NOX4|NR_120406	TFBS	TFBS	1688	1695	0.991863024	-	.	TF=SOX18;class=High-mobility group (HMG) domain factors;sequence=AACAATGC
NOX4|NM_001143837	TFBS	TFBS	1316	1323	0.991701267	-	.	TF=VSX2;class=Homeo domain factors;sequence=CTAATTAT
NOX4|NM_001300995	TFBS	TFBS	190	197	0.991551227	+	.	TF=RHOXF1;class=Homeo domain factors;sequence=GTAATCCC
NOX4|NM_001300995	TFBS	TFBS	2146	2153	0.991551227	+	.	TF=RHOXF1;class=Homeo domain factors;sequence=GTAATCCC
NOX4|NM_001300995	TFBS	TFBS	1084	1091	0.991551227	-	.	TF=RHOXF1;class=Homeo domain factors;sequence=GTAATCCC
NOX4|NM_001143837	TFBS	TFBS	2146	2153	0.991551227	+	.	TF=RHOXF1;class=Homeo domain factors;sequence=GTAATCCC
NOX4|NM_001291929	TFBS	TFBS	2146	2153	0.991551227	+	.	TF=RHOXF1;class=Homeo domain factors;sequence=GTAATCCC
NOX4|NM_001291927	TFBS	TFBS	2146	2153	0.991551227	+	.	TF=RHOXF1;class=Homeo domain factors;sequence=GTAATCCC
NOX4|NM_00	TFB	TFBS	214	21	0.99155	+	.	TF=RHOXF1;class=Home

1291926	S		6	53	1227			o domain factors;sequence=GTAATCCC
NOX4|NM_001291926	TFBS	TFBS	240	247	0.991551227	-	.	TF=RHOXF1;class=Homeo domain factors;sequence=GTAATCCC
NOX4|NM_016931	TFBS	TFBS	2146	2153	0.991551227	+	.	TF=RHOXF1;class=Homeo domain factors;sequence=GTAATCCC
NOX4|NM_016931	TFBS	TFBS	240	247	0.991551227	-	.	TF=RHOXF1;class=Homeo domain factors;sequence=GTAATCCC
NOX4|NM_001143836	TFBS	TFBS	2146	2153	0.991551227	+	.	TF=RHOXF1;class=Homeo domain factors;sequence=GTAATCCC
NOX4|NM_001143836	TFBS	TFBS	240	247	0.991551227	-	.	TF=RHOXF1;class=Homeo domain factors;sequence=GTAATCCC
NOX4|NR_120406	TFBS	TFBS	240	247	0.991551227	-	.	TF=RHOXF1;class=Homeo domain factors;sequence=GTAATCCC
NOX4|NM_001300995	TFBS	TFBS	1697	1704	0.991528411	+	.	TF=HOXA4;class=Homeo domain factors;sequence=ATCATTAA
NOX4|NM_001291929	TFBS	TFBS	238	245	0.991528411	+	.	TF=HOXA4;class=Homeo domain factors;sequence=ATCATTAA
NOX4|NM_001291927	TFBS	TFBS	456	463	0.991528411	+	.	TF=HOXA4;class=Homeo domain factors;sequence=ATCATTAA
NOX4|NM_001291926	TFBS	TFBS	853	860	0.991528411	+	.	TF=HOXA4;class=Homeo domain factors;sequence=ATCATTAA
NOX4|NM_01	TFB	TFBS	853	86	0.99152	+	.	TF=HOXA4;class=Homeo

6931	S			0	8411			domain factors;sequence=ATCATTAA
NOX4|NM_001143836	TFBS	TFBS	853	860	0.991528411	+	.	TF=HOXA4;class=Homeo domain factors;sequence=ATCATTAA
NOX4|NR_120406	TFBS	TFBS	853	860	0.991528411	+	.	TF=HOXA4;class=Homeo domain factors;sequence=ATCATTAA
NOX4|NM_001300995	TFBS	TFBS	147	154	0.991524102	+	.	TF=GSX2;class=Homeo domain factors;sequence=GTCATTAA
NOX4|NM_001291929	TFBS	TFBS	1919	1926	0.991524102	+	.	TF=GSX2;class=Homeo domain factors;sequence=GTCATTAA
NOX4|NM_001143837	TFBS	TFBS	1498	1507	0.991479147	-	.	TF=BHLHE22(var.2);class=Basic helix-loop-helix factors (bHLH);sequence=GCCAGCTGCG
NOX4|NM_001143837	TFBS	TFBS	1498	1507	0.991470498	+	.	TF=BHLHE22(var.2);class=Basic helix-loop-helix factors (bHLH);sequence=CGCAGCTGGC
NOX4|NM_001143837	TFBS	TFBS	1316	1323	0.991441186	+	.	TF=PDX1;class=Homeo domain factors;sequence=ATAATTAG
NOX4|NM_001143837	TFBS	TFBS	1641	1650	0.991437632	-	.	TF=MYB;class=Tryptophan cluster factors;sequence=CCCAACTGCT
NOX4|NM_001300995	TFBS	TFBS	1871	1878	0.991307845	+	.	TF=HOXD4;class=Homeo domain factors;sequence=CTCATTAG
NOX4|NM_00	TFB	TFBS	412	41	0.99130	+	.	TF=HOXD4;class=Homeo

<table>
<tr><td></td><td></td><td></td><td></td><td></td><td></td><td></td><td></td><td></td></tr>
<tr><td>1291929</td><td>S</td><td></td><td></td><td>9</td><td>7845</td><td></td><td></td><td>domain factors;sequence=CTCATTAG</td></tr>
<tr><td>NOX4|NM_001291927</td><td>TFBS</td><td>TFBS</td><td>630</td><td>637</td><td>0.991307845</td><td>+</td><td>.</td><td>TF=HOXD4;class=Homeo domain factors;sequence=CTCATTAG</td></tr>
<tr><td>NOX4|NM_001291926</td><td>TFBS</td><td>TFBS</td><td>1027</td><td>1034</td><td>0.991307845</td><td>+</td><td>.</td><td>TF=HOXD4;class=Homeo domain factors;sequence=CTCATTAG</td></tr>
<tr><td>NOX4|NM_016931</td><td>TFBS</td><td>TFBS</td><td>1027</td><td>1034</td><td>0.991307845</td><td>+</td><td>.</td><td>TF=HOXD4;class=Homeo domain factors;sequence=CTCATTAG</td></tr>
<tr><td>NOX4|NM_001143836</td><td>TFBS</td><td>TFBS</td><td>1027</td><td>1034</td><td>0.991307845</td><td>+</td><td>.</td><td>TF=HOXD4;class=Homeo domain factors;sequence=CTCATTAG</td></tr>
<tr><td>NOX4|NR_120406</td><td>TFBS</td><td>TFBS</td><td>1027</td><td>1034</td><td>0.991307845</td><td>+</td><td>.</td><td>TF=HOXD4;class=Homeo domain factors;sequence=CTCATTAG</td></tr>
<tr><td>NOX4|NM_001300995</td><td>TFBS</td><td>TFBS</td><td>746</td><td>753</td><td>0.991279322</td><td>-</td><td>.</td><td>TF=TLX2;class=Homeo domain factors;sequence=CCAATTAA</td></tr>
<tr><td>NOX4|NM_001300995</td><td>TFBS</td><td>TFBS</td><td>1718</td><td>1725</td><td>0.991279322</td><td>-</td><td>.</td><td>TF=TLX2;class=Homeo domain factors;sequence=CCAATTAA</td></tr>
<tr><td>NOX4|NM_001291929</td><td>TFBS</td><td>TFBS</td><td>259</td><td>266</td><td>0.991279322</td><td>-</td><td>.</td><td>TF=TLX2;class=Homeo domain factors;sequence=CCAATTAA</td></tr>
<tr><td>NOX4|NM_001291927</td><td>TFBS</td><td>TFBS</td><td>477</td><td>484</td><td>0.991279322</td><td>-</td><td>.</td><td>TF=TLX2;class=Homeo domain factors;sequence=CCAATTAA</td></tr>
<tr><td>NOX4|NM_001291926</td><td>TFBS</td><td>TFBS</td><td>874</td><td>881</td><td>0.991279322</td><td>-</td><td>.</td><td>TF=TLX2;class=Homeo domain factors;sequence=CCAATTAA</td></tr>
<tr><td>NOX4|NM_01</td><td>TFB</td><td>TFBS</td><td>874</td><td>88</td><td>0.99127</td><td>-</td><td>.</td><td>TF=TLX2;class=Homeo</td></tr>
</table>

6931	S			1	9322			domain factors;sequence=CCAATTAA
NOX4|NM_001143836	TFBS	TFBS	874	881	0.991279322	-	.	TF=TLX2;class=Homeo domain factors;sequence=CCAATTAA
NOX4|NR_120406	TFBS	TFBS	874	881	0.991279322	-	.	TF=TLX2;class=Homeo domain factors;sequence=CCAATTAA
NOX4|NM_001300995	TFBS	TFBS	746	753	0.991264087	-	.	TF=BARX1;class=Homeo domain factors;sequence=CCAATTAA
NOX4|NM_001300995	TFBS	TFBS	1718	1725	0.991264087	-	.	TF=BARX1;class=Homeo domain factors;sequence=CCAATTAA
NOX4|NM_001291929	TFBS	TFBS	259	266	0.991264087	-	.	TF=BARX1;class=Homeo domain factors;sequence=CCAATTAA
NOX4|NM_001291927	TFBS	TFBS	477	484	0.991264087	-	.	TF=BARX1;class=Homeo domain factors;sequence=CCAATTAA
NOX4|NM_001291926	TFBS	TFBS	874	881	0.991264087	-	.	TF=BARX1;class=Homeo domain factors;sequence=CCAATTAA
NOX4|NM_016931	TFBS	TFBS	874	881	0.991264087	-	.	TF=BARX1;class=Homeo domain factors;sequence=CCAATTAA
NOX4|NM_001143836	TFBS	TFBS	874	881	0.991264087	-	.	TF=BARX1;class=Homeo domain factors;sequence=CCAATTAA
NOX4|NR_120406	TFBS	TFBS	874	881	0.991264087	-	.	TF=BARX1;class=Homeo domain factors;sequence=CCAATTAA
NOX4|NM_00	TFB	TFBS	182	18	0.99122	-	.	TF=MNX1;class=Homeo

1143837	S		7	36	8646			domain factors;sequence=GATAATTAAA
NOX4|NM_001300995	TFBS	TFBS	325	332	0.991075076	+	.	TF=RHOXF1;class=Homeo domain factors;sequence=GTGATCCC
NOX4|NM_001300995	TFBS	TFBS	231	239	0.990771625	+	.	TF=NR2C1;class=Nuclear receptors with C4 zinc fingers;sequence=TGAGGTCAG
NOX4|NM_001300995	TFBS	TFBS	2187	2195	0.990771625	+	.	TF=NR2C1;class=Nuclear receptors with C4 zinc fingers;sequence=TGAGGTCAG
NOX4|NM_001143837	TFBS	TFBS	2187	2195	0.990771625	+	.	TF=NR2C1;class=Nuclear receptors with C4 zinc fingers;sequence=TGAGGTCAG
NOX4|NM_001291929	TFBS	TFBS	2187	2195	0.990771625	+	.	TF=NR2C1;class=Nuclear receptors with C4 zinc fingers;sequence=TGAGGTCAG
NOX4|NM_001291927	TFBS	TFBS	2187	2195	0.990771625	+	.	TF=NR2C1;class=Nuclear receptors with C4 zinc fingers;sequence=TGAGGTCAG
NOX4|NM_001291926	TFBS	TFBS	2187	2195	0.990771625	+	.	TF=NR2C1;class=Nuclear receptors with C4 zinc fingers;sequence=TGAGGTCAG
NOX4|NM_016931	TFBS	TFBS	2187	2195	0.990771625	+	.	TF=NR2C1;class=Nuclear receptors with C4 zinc fingers;sequence=TGAGGTCAG
NOX4|NM_001143836	TFBS	TFBS	2187	2195	0.990771625	+	.	TF=NR2C1;class=Nuclear receptors with C4

								zinc fingers;sequence=TGAGGTCAG
NOX4|NM_001300995	TFBS	TFBS	536	543	0.990638987	+	.	TF=HOXD4;class=Homeo domain factors;sequence=CTCATTAA
NOX4|NM_001300995	TFBS	TFBS	540	547	0.990638987	−	.	TF=HOXD4;class=Homeo domain factors;sequence=CTCATTAA
NOX4|NM_001143837	TFBS	TFBS	716	723	0.990638987	+	.	TF=HOXD4;class=Homeo domain factors;sequence=CTCATTAA
NOX4|NR_120406	TFBS	TFBS	2325	2332	0.990638987	+	.	TF=HOXD4;class=Homeo domain factors;sequence=CTCATTAA
NOX4|NM_001143837	TFBS	TFBS	510	517	0.990531244	+	.	TF=OTX1;class=Homeo domain factors;sequence=TTAATCCC
NOX4|NM_001143837	TFBS	TFBS	1787	1794	0.990497575	−	.	TF=RHOXF1;class=Homeo domain factors;sequence=GTAATCCA
NOX4|NM_001300995	TFBS	TFBS	2412	2423	0.990326324	+	.	TF=ZNF384;class=C2H2 zinc finger factors;sequence=TCAAAAAAAAA
NOX4|NM_001300995	TFBS	TFBS	822	833	0.990326324	−	.	TF=ZNF384;class=C2H2 zinc finger factors;sequence=TCAAAAAAAAA
NOX4|NM_001143837	TFBS	TFBS	2412	2423	0.990326324	+	.	TF=ZNF384;class=C2H2 zinc finger factors;sequence=TCAAAAAAAAA
NOX4|NM_001291929	TFBS	TFBS	2412	2423	0.990326324	+	.	TF=ZNF384;class=C2H2 zinc finger factors;sequence=TCAAAAAAAAA
NOX4|NM_00	TFB	TFBS	241	24	0.99032	+	.	TF=ZNF384;class=C2H2

1291927	S		2	23	6324		.	zinc finger factors;sequence=TCAAAAAAAAA
NOX4|NM_001291926	TFBS	TFBS	2412	2423	0.990326324	+	.	TF=ZNF384;class=C2H2 zinc finger factors;sequence=TCAAAAAAAAA
NOX4|NM_016931	TFBS	TFBS	2412	2423	0.990326324	+	.	TF=ZNF384;class=C2H2 zinc finger factors;sequence=TCAAAAAAAAA
NOX4|NM_001143836	TFBS	TFBS	2412	2423	0.990326324	+	.	TF=ZNF384;class=C2H2 zinc finger factors;sequence=TCAAAAAAAAA
NOX4|NM_001291929	TFBS	TFBS	1716	1723	0.990325926	+	.	TF=GATA3;class=Other C4 zinc finger-type factors;sequence=AGATAACA
NOX4|NM_001291927	TFBS	TFBS	1934	1941	0.990325926	+	.	TF=GATA3;class=Other C4 zinc finger-type factors;sequence=AGATAACA
NOX4|NM_001143837	TFBS	TFBS	1316	1323	0.990250825	−	.	TF=PRRX2;class=Homeo domain factors;sequence=CTAATTAT
NOX4|NM_001300995	TFBS	TFBS	1543	1550	0.990192875	+	.	TF=FOXG1;class=Fork head / winged helix factors;sequence=ATAAACAA
NOX4|NM_001143837	TFBS	TFBS	288	295	0.990192875	−	.	TF=FOXG1;class=Fork head / winged helix factors;sequence=ATAAACAA
NOX4|NM_001291929	TFBS	TFBS	84	91	0.990192875	+	.	TF=FOXG1;class=Fork head / winged helix factors;sequence=ATAAACAA
NOX4|NM_001291927	TFBS	TFBS	302	309	0.990192875	+	.	TF=FOXG1;class=Fork head / winged helix factors;sequence=ATAAACAA
NOX4|NM_00	TFB	TFBS	699	70	0.99019	+	.	TF=FOXG1;class=Fork

1291926	S			6	2875			head / winged helix factors;sequence=ATAAACAA
NOX4|NM_016931	TFBS	TFBS	699	706	0.990192875	+	.	TF=FOXG1;class=Fork head / winged helix factors;sequence=ATAAACAA
NOX4|NM_001143836	TFBS	TFBS	699	706	0.990192875	+	.	TF=FOXG1;class=Fork head / winged helix factors;sequence=ATAAACAA
NOX4|NR_120406	TFBS	TFBS	699	706	0.990192875	+	.	TF=FOXG1;class=Fork head / winged helix factors;sequence=ATAAACAA
NOX4|NM_001300995	TFBS	TFBS	746	753	0.990135478	–	.	TF=LHX9;class=Homeo domain factors;sequence=CCAATTAA
NOX4|NM_001300995	TFBS	TFBS	1718	1725	0.990135478	–	.	TF=LHX9;class=Homeo domain factors;sequence=CCAATTAA
NOX4|NM_001291929	TFBS	TFBS	259	266	0.990135478	–	.	TF=LHX9;class=Homeo domain factors;sequence=CCAATTAA
NOX4|NM_001291927	TFBS	TFBS	477	484	0.990135478	–	.	TF=LHX9;class=Homeo domain factors;sequence=CCAATTAA
NOX4|NM_001291926	TFBS	TFBS	874	881	0.990135478	–	.	TF=LHX9;class=Homeo domain factors;sequence=CCAATTAA
NOX4|NM_016931	TFBS	TFBS	874	881	0.990135478	–	.	TF=LHX9;class=Homeo domain factors;sequence=CCAATTAA
NOX4|NM_001143836	TFBS	TFBS	874	881	0.990135478	–	.	TF=LHX9;class=Homeo domain factors;sequence=CCAATTAA
NOX4|NR_12	TFB	TFBS	874	88	0.99013	–	.	TF=LHX9;class=Homeo

<table>
<tr><td>0406</td><td>S</td><td></td><td></td><td>1</td><td>5478</td><td></td><td></td><td>domain factors;sequence=CCAATTAA</td></tr>
<tr><td>NOX4|NM_001300995</td><td>TFBS</td><td>TFBS</td><td>1052</td><td>1059</td><td>0.990021423</td><td>+</td><td>.</td><td>TF=RHOXF1;class=Homeo domain factors;sequence=GTGATCCA</td></tr>
<tr><td>NOX4|NM_001300995</td><td>TFBS</td><td>TFBS</td><td>222</td><td>229</td><td>0.990021423</td><td>−</td><td>.</td><td>TF=RHOXF1;class=Homeo domain factors;sequence=GTGATCCA</td></tr>
<tr><td>NOX4|NM_001291926</td><td>TFBS</td><td>TFBS</td><td>208</td><td>215</td><td>0.990021423</td><td>+</td><td>.</td><td>TF=RHOXF1;class=Homeo domain factors;sequence=GTGATCCA</td></tr>
<tr><td>NOX4|NM_016931</td><td>TFBS</td><td>TFBS</td><td>208</td><td>215</td><td>0.990021423</td><td>+</td><td>.</td><td>TF=RHOXF1;class=Homeo domain factors;sequence=GTGATCCA</td></tr>
<tr><td>NOX4|NM_001143836</td><td>TFBS</td><td>TFBS</td><td>208</td><td>215</td><td>0.990021423</td><td>+</td><td>.</td><td>TF=RHOXF1;class=Homeo domain factors;sequence=GTGATCCA</td></tr>
<tr><td>NOX4|NR_120406</td><td>TFBS</td><td>TFBS</td><td>208</td><td>215</td><td>0.990021423</td><td>+</td><td>.</td><td>TF=RHOXF1;class=Homeo domain factors;sequence=GTGATCCA</td></tr>
<tr><td>NOX4|NR_120406</td><td>TFBS</td><td>TFBS</td><td>2166</td><td>2173</td><td>0.990021423</td><td>−</td><td>.</td><td>TF=RHOXF1;class=Homeo domain factors;sequence=GTGATCCA</td></tr>
</table>
