# Supplementary figures and images for: Development of a Ferroptosis-Related lncRNA Signature to Predict the Prognosis and Immune Landscape of Bladder Cancer
Source: Dis Markers. 2021 Jun 20;2021:1031906. doi: 10.1155/2021/1031906 (PMC8238626; doi:10.1155/2021/1031906)

a

Survival curve ( $p=1.216e-11$ )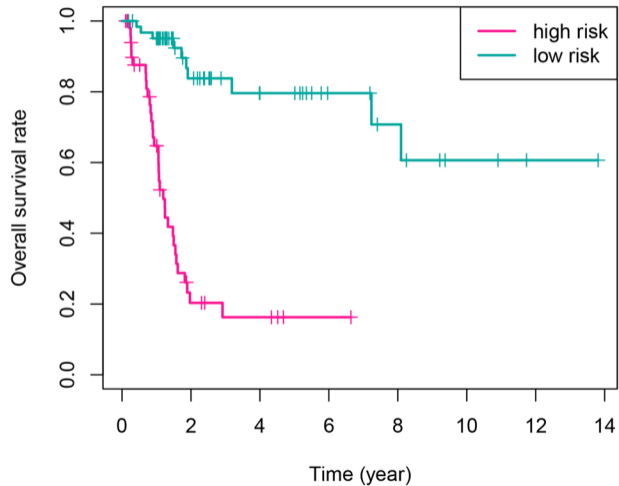

b

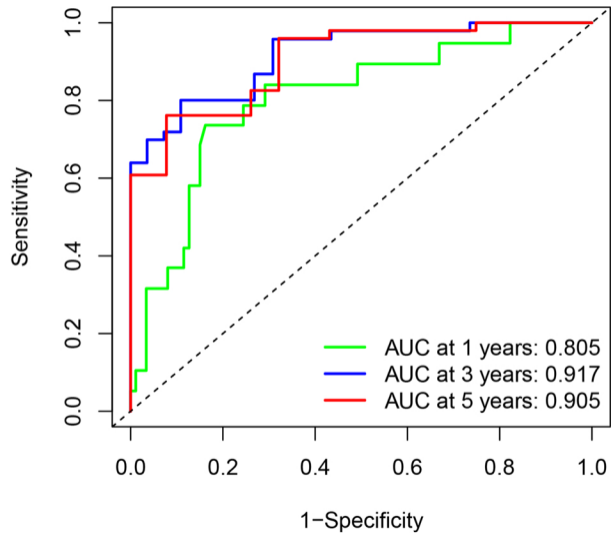

Supplement: Supplementary Materials — Supplementary Figure S1: validation of the risk model with resampling. Note: (a) Kaplan-Meier survival analysis of cases in high-risk group and low-risk group (p < 0.001). (b) ROC curves of the risk score in internal validation dataset. Supplementary Figure S2: Kaplan-Meier subgroup survival analysis in TCGA-BLCA patients. Note: (a) age ≤ 64 (p < 0.001). (b) Age > 64 (p < 0.001). (c) Male (p < 0.001). (d) Female (p < 0.001). (e) Pathological T 1-2 stages (p < 0.001). (f) Pathological T 3-4 stages (p < 0.001). (g) N0 stages (p < 0.001). (h) N1-N3 stages (p < 0.001). (i) M0 stage (p < 0.001). (j) M1 stage (p < 0.05). Supplementary Figure S3: functional prediction of AC090825.1 and MAGI2-AS3. Note: (a) the expression difference of AC090825.1 in BCa samples and paracarcinoma tissue via Wilcoxon signed-rank test. (b) The expression difference of MAGI2-AS3 in BCa samples and paracarcinoma tissue via Wilcoxon signed-rank test. (c) The difference values between the expression of AC090825.1 and MAGI2-AS3 in BCa samples and paracarcinoma samples. (d) The cases suffered poorer survival when the expression value of AC090825.1 was higher than that of MAGI2-AS3 (p < 0.01). (e) The expression levels of AC090825.1 and MAGI2-AS3 were positively correlated both in BCa samples and paracarcinoma samples. (f) Network of lncRNAs, the target genes and their possible transcription factors. (g) UpSet diagram showing 6 overlapped transcription factors. (h) The correlation network of lncRNAs, the target genes and transcription factors. BCa: bladder cancer. Supplementary Table S1: 20 ferroptosis regulators were differentially expressed between BCa and paracarinoma samples. Supplementary Table S2: the Pearson correlation analysis of lncRNAs and ferroptosis regulators. Supplementary Table S3: Kaplan-Meier survival analysis of the lncRNA pairs. Supplementary Table S4: enrichment results from LncSEA website. Supplementary Table S5: GSVA results of different clusters based on ferroptosis regulators [file 1031906.f1.zip › Supplementary_Figure_S1 (1).PDF]

**a****Age $\leq$ 64 ( $p=4.375e-04$ )**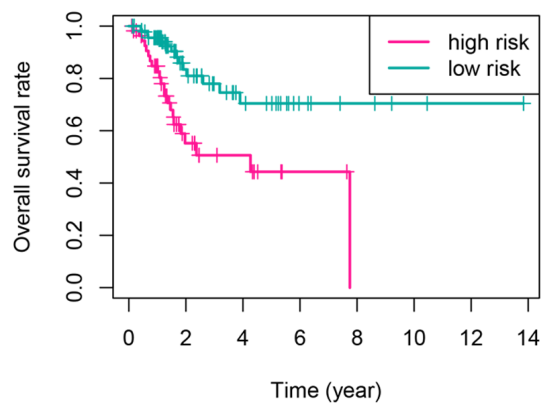**b****Age $>$ 64 ( $p=1.665e-15$ )**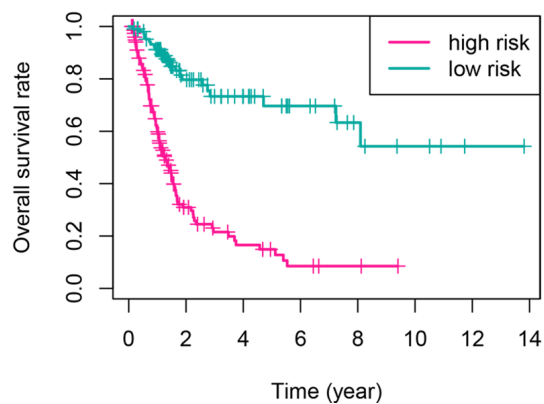**c****Male ( $p=0e+00$ )**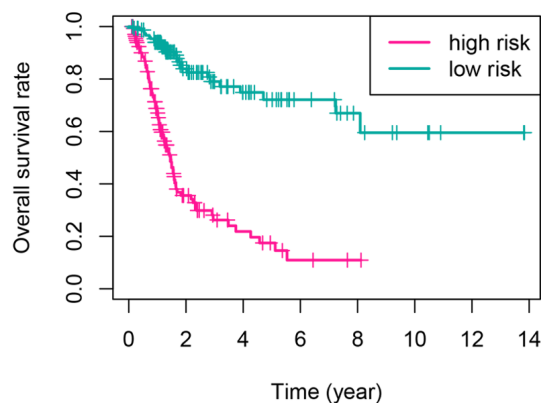**d****Female ( $p=5.7e-04$ )**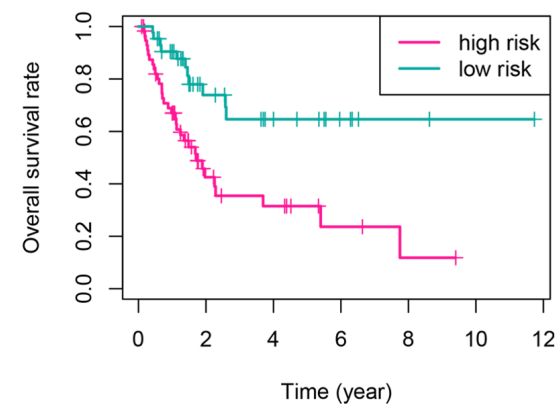**e****T 1-2 ( $p=4.534e-05$ )**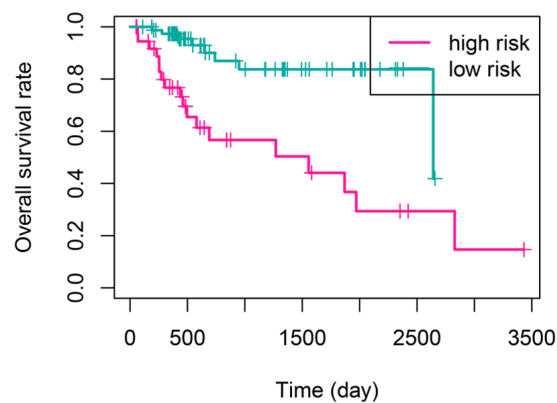**f****T 3-4 ( $p=6.558e-11$ )**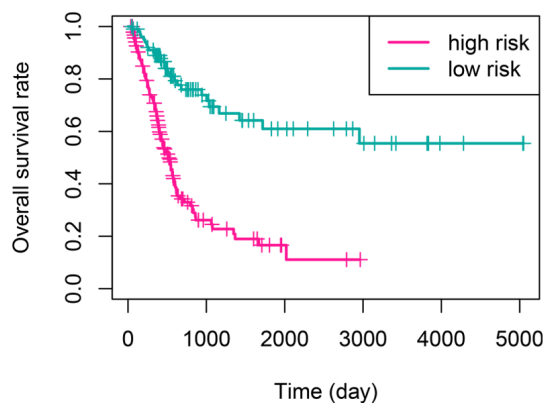**g****N0 ( $p=6.804e-11$ )**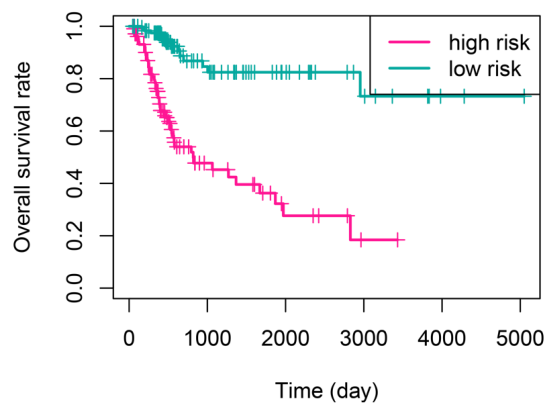**h****N1-3 ( $p=2.906e-07$ )**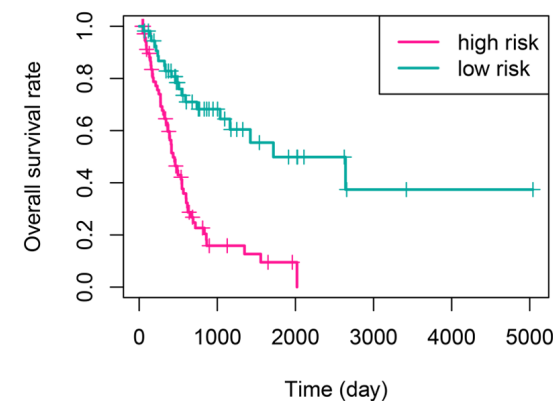**i****M0 stage ( $p=2.461e-12$ )**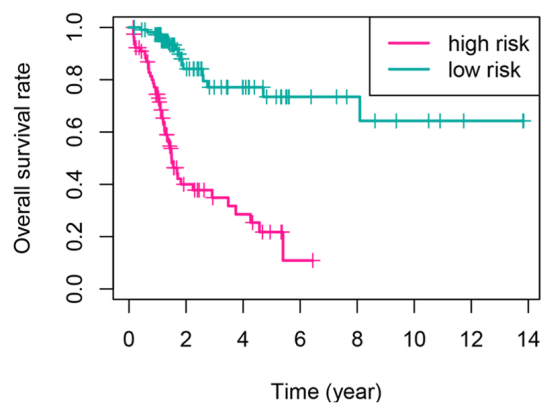**j****M1 stage ( $p=4.025e-02$ )**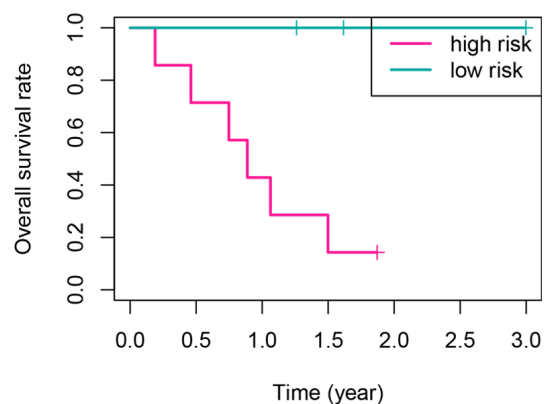

Supplement: Supplementary Materials — Supplementary Figure S1: validation of the risk model with resampling. Note: (a) Kaplan-Meier survival analysis of cases in high-risk group and low-risk group (p < 0.001). (b) ROC curves of the risk score in internal validation dataset. Supplementary Figure S2: Kaplan-Meier subgroup survival analysis in TCGA-BLCA patients. Note: (a) age ≤ 64 (p < 0.001). (b) Age > 64 (p < 0.001). (c) Male (p < 0.001). (d) Female (p < 0.001). (e) Pathological T 1-2 stages (p < 0.001). (f) Pathological T 3-4 stages (p < 0.001). (g) N0 stages (p < 0.001). (h) N1-N3 stages (p < 0.001). (i) M0 stage (p < 0.001). (j) M1 stage (p < 0.05). Supplementary Figure S3: functional prediction of AC090825.1 and MAGI2-AS3. Note: (a) the expression difference of AC090825.1 in BCa samples and paracarcinoma tissue via Wilcoxon signed-rank test. (b) The expression difference of MAGI2-AS3 in BCa samples and paracarcinoma tissue via Wilcoxon signed-rank test. (c) The difference values between the expression of AC090825.1 and MAGI2-AS3 in BCa samples and paracarcinoma samples. (d) The cases suffered poorer survival when the expression value of AC090825.1 was higher than that of MAGI2-AS3 (p < 0.01). (e) The expression levels of AC090825.1 and MAGI2-AS3 were positively correlated both in BCa samples and paracarcinoma samples. (f) Network of lncRNAs, the target genes and their possible transcription factors. (g) UpSet diagram showing 6 overlapped transcription factors. (h) The correlation network of lncRNAs, the target genes and transcription factors. BCa: bladder cancer. Supplementary Table S1: 20 ferroptosis regulators were differentially expressed between BCa and paracarinoma samples. Supplementary Table S2: the Pearson correlation analysis of lncRNAs and ferroptosis regulators. Supplementary Table S3: Kaplan-Meier survival analysis of the lncRNA pairs. Supplementary Table S4: enrichment results from LncSEA website. Supplementary Table S5: GSVA results of different clusters based on ferroptosis regulators [file 1031906.f1.zip › Supplementary_Figure_S2 (3).pdf]

**a**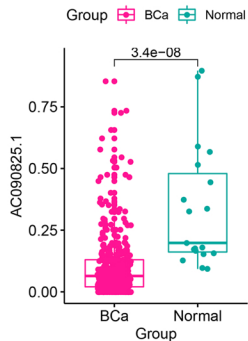**b**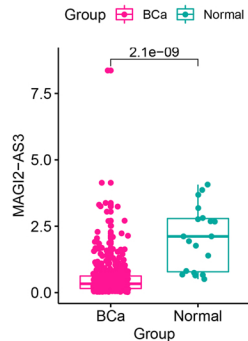**c**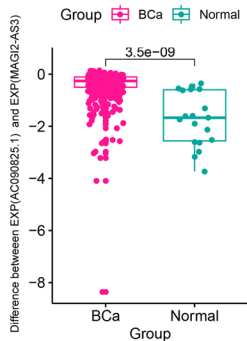**d**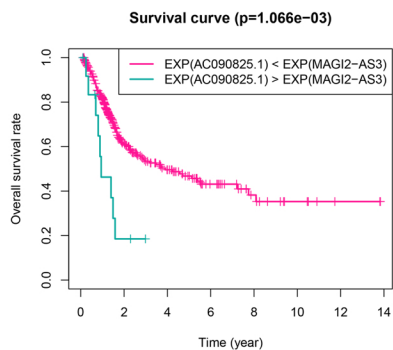**e**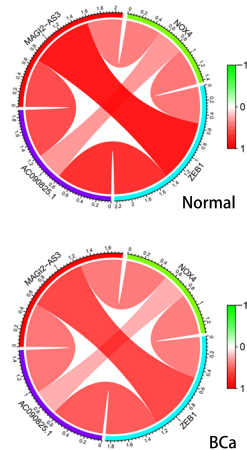**f**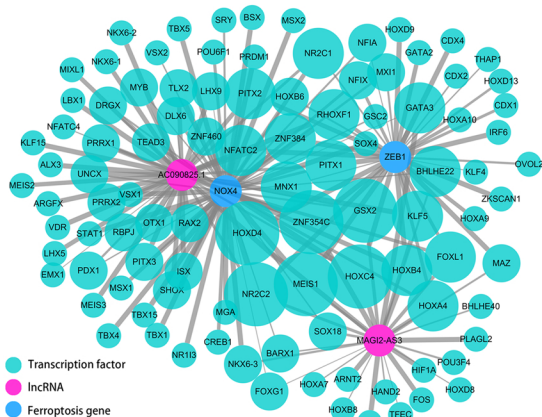**g**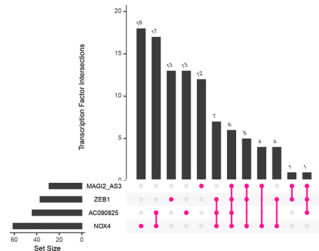**h**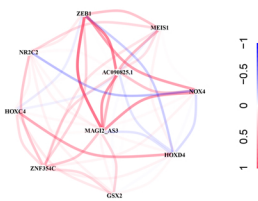

Supplement: Supplementary Materials — Supplementary Figure S1: validation of the risk model with resampling. Note: (a) Kaplan-Meier survival analysis of cases in high-risk group and low-risk group (p < 0.001). (b) ROC curves of the risk score in internal validation dataset. Supplementary Figure S2: Kaplan-Meier subgroup survival analysis in TCGA-BLCA patients. Note: (a) age ≤ 64 (p < 0.001). (b) Age > 64 (p < 0.001). (c) Male (p < 0.001). (d) Female (p < 0.001). (e) Pathological T 1-2 stages (p < 0.001). (f) Pathological T 3-4 stages (p < 0.001). (g) N0 stages (p < 0.001). (h) N1-N3 stages (p < 0.001). (i) M0 stage (p < 0.001). (j) M1 stage (p < 0.05). Supplementary Figure S3: functional prediction of AC090825.1 and MAGI2-AS3. Note: (a) the expression difference of AC090825.1 in BCa samples and paracarcinoma tissue via Wilcoxon signed-rank test. (b) The expression difference of MAGI2-AS3 in BCa samples and paracarcinoma tissue via Wilcoxon signed-rank test. (c) The difference values between the expression of AC090825.1 and MAGI2-AS3 in BCa samples and paracarcinoma samples. (d) The cases suffered poorer survival when the expression value of AC090825.1 was higher than that of MAGI2-AS3 (p < 0.01). (e) The expression levels of AC090825.1 and MAGI2-AS3 were positively correlated both in BCa samples and paracarcinoma samples. (f) Network of lncRNAs, the target genes and their possible transcription factors. (g) UpSet diagram showing 6 overlapped transcription factors. (h) The correlation network of lncRNAs, the target genes and transcription factors. BCa: bladder cancer. Supplementary Table S1: 20 ferroptosis regulators were differentially expressed between BCa and paracarinoma samples. Supplementary Table S2: the Pearson correlation analysis of lncRNAs and ferroptosis regulators. Supplementary Table S3: Kaplan-Meier survival analysis of the lncRNA pairs. Supplementary Table S4: enrichment results from LncSEA website. Supplementary Table S5: GSVA results of different clusters based on ferroptosis regulators [file 1031906.f1.zip › Supplementary_Figure_S3 (1).PDF]
